# Association of Concomitant Use of Cholinesterase Inhibitors or Memantine With Cognitive Decline in Alzheimer Clinical Trials: A Meta-analysis

**DOI:** 10.1001/jamanetworkopen.2018.4080

**Published:** 2018-11-02

**Authors:** Richard E. Kennedy, Gary R. Cutter, Mackenzie E. Fowler, Lon S. Schneider

**Affiliations:** 1 University of Alabama at Birmingham, Birmingham; 2 Keck School of Medicine, University of Southern California, Los Angeles

## Abstract

**Importance:**

Clinical trials in Alzheimer disease (AD) generally allow participants to continue receiving concomitant medications, including cholinesterase inhibitors (ChEIs) and memantine, if the dose is stable. Previous analysis of observational studies indicates such individuals experience greater rate of decline on cognitive testing than those not receiving such medications.

**Objective:**

To investigate whether concomitant use of ChEIs or memantine is associated with cognitive outcomes in AD clinical trials.

**Data Sources:**

Meta-database of 18 studies from the Alzheimer Disease Cooperative Study and Alzheimer Disease Neuroimaging Initiative.

**Study Selection:**

All studies with data on ChEI and memantine use that included assessment of specified outcome measures.

**Data Extraction and Synthesis:**

The analysis estimated annual rate of decline on the Alzheimer Disease Assessment Scale–cognitive subscale (ADAS-cog) using linear mixed-effects models, and compared rates for participants receiving ChEIs and memantine, alone and combined, with participants not receiving either medication using random-effects meta-analysis.

**Main Outcomes and Measures:**

Annual rate of change on the ADAS-cog.

**Results:**

Across 10 studies, of 2714 participants, the mean (SD) age was 75.0 (8.2) years, 58% were female, and 9% were racial/ethnic minorities. There were 906 participants (33.4%) receiving ChEIs, 143 (5.3%) receiving memantine, 923 (34.0%) receiving both, and 742 (27.3%) receiving neither. Meta-analysis showed those receiving ChEIs or memantine were associated with significantly greater annual rate of decline on the ADAS-cog than those receiving neither medication (1.4 points/y; 95% CI, 0.1-2.7).

**Conclusions and Relevance:**

Similar to observational studies, many participants in AD clinical trials receiving ChEIs or memantine experience greater cognitive decline. This difference is nearly as large as the hypothesized effect sizes of the treatments investigated in the trials. Concomitant use of ChEIs or memantine may be confounded with outcomes on the ADAS-cog and should be considered in design of clinical trials of potential therapeutic agents for AD. Post hoc analyses stratifying by ChEIs or memantine must be interpreted cautiously given the potential for confounding.

## Key Points

**Question:**

Are cholinesterase inhibitors or memantine associated with cognitive outcomes in clinical trials for Alzheimer disease?

**Findings:**

In this meta-analysis, participants receiving cholinesterase inhibitors or memantine had 1.4 points per year difference on the Alzheimer Disease Assessment Scale–cognitive subscale compared with those receiving neither medication, a significant difference that is roughly the same size as the expected effect of new therapeutic drugs being investigated in the clinical trials.

**Meaning:**

Differences in the use of cholinesterase inhibitors and memantine between treatment and placebo groups of clinical trials may lead to the conclusion that a treatment is effective when it is not, or vice versa.

## Teaser

This meta-analysis investigates whether concomitant use of cholinesterase inhibitors or memantine affect cognitive outcomes in Alzheimer disease (AD) clinical trials.

## Introduction

Cholinesterase inhibitors (ChEIs) and memantine are currently approved by the US Food and Drug Administration for the treatment of dementia due to Alzheimer disease (AD). The former are approved for all stages of dementia, but not for mild cognitive impairment (MCI) due to AD. Memantine is only approved for moderate to severe dementia due to AD. Both ChEIs and memantine are, nevertheless, often prescribed earlier in the disease course than indicated by US Food and Drug Administration labeling.^1^

Clinical trials of new therapies for AD typically allow participants to continue receiving ChEIs and memantine during the trial if the dose remains stable. Thus, it is critical to know whether participants receiving these concomitant medications differ significantly from those not receiving these medications, particularly if such differences may affect trial outcome. Schneider and colleagues^1^ examined this issue in the observational Alzheimer Neuroimaging Initiative (ADNI)^2^ database. Among the 402 participants with MCI due to AD, 44.0% were receiving ChEIs and 11.4% were receiving memantine at baseline; among the 188 participants with dementia due to AD, 84.6% were receiving ChEIs and 45.7% were receiving memantine at baseline. Those in the former group had greater impairment on the Alzheimer Disease Assessment Scale–cognitive subscale (ADAS-cog)^3^ and the Clinical Dementia Rating (CDR)^4^ scale at baseline; more rapid cognitive decline on the ADAS-cog, Mini-Mental State Examination,^5^ and the CDR-sum of boxes over time; and shorter time to progress to dementia than participants with MCI due to AD who were not receiving ChEIs. Participants receiving both ChEIs and memantine had worse performance than those receiving only ChEIs. Participants with dementia due to AD receiving both ChEIs and memantine showed greater impairment on the CDR at baseline; and more rapid decline on the Mini-Mental State Examination and CDR, but not on the ADAS-cog, over time than participants receiving only ChEIs.

It is unclear, however, whether these differences among participants enrolling in observational studies are also present in participants enrolling in clinical trials for AD. The magnitude of these differences is roughly equal to the expected drug placebo difference for a successful therapeutic agent for AD. Thus, imbalances in the usage of ChEIs and memantine among participants in clinical trials could easily obscure the effects of investigational treatments for AD. Differences among participants receiving and not receiving concomitant medications would be particularly problematic for the increasing number of post hoc analyses of clinical trial data to identify potentially responsive subgroups, where imbalances in usage of ChEIs and memantine can easily occur. We sought to examine this issue further using data from a meta-database of AD clinical trials and observational studies.

## Methods

All procedures for the original studies were approved by local institutional review boards. The analyses for this study were exempted from informed consent requirements by the institutional review boards. We followed the Preferred Reporting Items for Systematic Reviews and Meta-analyses (PRISMA) reporting guideline as applicable.

### Study Overview and Participants

Participants were drawn from a meta-database^6^ consisting of 18 studies from the Alzheimer Disease Cooperative Study^7^ and ADNI,^2^ representing both clinical trials and observational studies in dementia due to AD, MCI due to AD, and normal participants (eTable 1 in the Supplement). This meta-database contains all published studies from the Alzheimer Disease Cooperative Study and ADNI through 2011. All diagnoses of dementia due to AD were based on the National Institute of Neurological Disorders and Stroke–Alzheimer’s Disease and Related Disorders Association criteria,^8^ with the additional requirement of minimal severity based on clinical ratings. Only studies of dementia due to AD were used in analyses. We did not include participants with diagnoses of MCI due to the small number of MCI studies available. Clinical assessments were done at 6-month intervals over the first 2 years.

### Outcomes

#### Cognition

Cognitive assessments were performed using the ADAS-cog,^3^ which evaluates memory, reasoning, orientation, praxis, language, and word finding difficulty, and is scored from 0 to 70 errors, so that higher scores represent more impairment. A 2-point difference on the ADAS-cog is often used to define a significant therapeutic effect in AD clinical trials.^9^ Analyses were restricted to 10 studies (9 randomized clinical trials and 1 observational ADNI study) of dementia due to AD with the ADAS-cog as primary outcome and duration of at least 6 months,^2,10,11,12,13,14,15,16,17,18^ with a total of 2714 participants.

### Statistical Analysis

For each individual study, rates of cognitive decline were estimated using mixed-effects linear models^19^ to test for differences in slopes (ie, rates of change in points/y) of the ADAS-cog between medication groups (ChEIs, memantine, both, and neither). We compared rates for individuals receiving ChEIs, memantine, or both to rates for individuals receiving neither medication as the primary analysis. Additional analyses compared rates for individuals receiving ChEIs to rates for individuals receiving neither, and rates for individuals receiving memantine or both to rates for individuals receiving ChEIs or neither. The mixed-effects model was performed as it used data from all participants (rather than just completers), minimizes bias, and better controls for type I error in the presence of missing data.^20^ The model was constructed with group and time effects and group × time interactions, with age and education as covariates. An unstructured covariance matrix was used to model independence of the slope and intercept parameters. The group × time interaction was evaluated using a likelihood ratio test to determine group differences in slopes. Additional details on the statistical analysis are provided in eAppendix 1 in the Supplement. Mixed-effects models were performed using the R statistical environment, version 3.5.0 (R Foundation) with the *lme4* package. Source code for statistical analyses is provided in the eAppendix 2 in the Supplement. Effect sizes (Cohen *d*) were calculated from the mixed model *t* statistics using the *EMAtools* package for R, version 0.1.3 (R Foundation).

Estimated rates of decline and effect sizes were combined across the 10 individual studies using random-effects meta-analysis to determine the overall effect of concomitant medications on cognitive decline. The random-effects model was used as it is preferred over the fixed-effects model when significant heterogeneity is present.^21^ Weighting by sample size was used to avoid excessive influence of smaller studies. Funnel plots^22^ and Fail Safe N^23^ were used to assess for evidence of systematic bias. We also examined study year and percentage of participants receiving concomitant medications as moderators of the effect on rates of cognitive decline. Meta-analysis was performed using the R statistical software (package metafor, R Foundation).^24^

## Results

### Patient Characteristics

Across 10 studies, consisting of 2714 participants (mean [SD] age was 75.0 [8.2] years; 58% female; 9% racial/ethnic minorities), 906 participants (33.4%) were receiving ChEIs, 143 (5.3%) were receiving memantine, 923 (34.0%) were receiving both, and 742 (27.3%) were receiving neither. Participants receiving ChEIs or memantine at baseline tended to have more education, were more likely to be male, and were more likely to be married than participants not receiving either medication (Table). Individuals receiving memantine were older than those not receiving memantine, and those receiving memantine (with or without ChEIs) were more likely to be white. Interestingly, those receiving ChEIs or memantine were more likely to be in the placebo arm of trials. Participant characteristics for each of the 10 studies are shown in eTables 2-11 in the Supplement.

**Table.** Participant Characteristics by Concomitant Medication Group

Variable	No.	Both ChEI and Memantine (n = 923)	ChEI (n = 906)	Memantine (n = 143)	Neither (n = 742)	*P* Value
Age, mean (SD), y	2714	74.5 (8.8)	75.5 (7.8)	78.1 (7.2)	74.5 (8.2)	<.001
Education, No. (%)	2714					<.001
Less than high school		72 (7.8)	126 (13.9)	20 (14.0)	169 (22.8)	
High school graduate		437 (47.3)	447 (49.3)	69 (48.3)	383 (51.6)	
College graduate		414 (44.9)	333 (36.8)	54 (37.8)	190 (25.6)	
White race, No. (%)	2714	851 (92.2)	821 (90.6)	134 (93.7)	651 (87.7)	.01
Hispanic ethnicity, No. (%)	2714	32 (3.47)	45 (4.97)	9 (6.29)	38 (5.12)	.22
Female, No. (%)	2714	478 (51.8)	529 (58.4)	94 (65.7)	477 (64.3)	<.001
Married, No. (%)	2714	714 (77.4)	637 (70.3)	101 (70.6)	505 (68.1)	<.001
Treatment arm/placebo, No. (%)	2714	490 (53.1)	385 (42.5)	68 (47.6)	246 (33.2)	<.001
ADAS-cog, mean (SD), mo^a^						
Baseline	2714	25.7 (9.5)	22.5 (8.8)	28.4 (11.0)	29.3 (13.2)	<.001
6	2240	27.5 (10.2)	23.7 (9.6)	27.2 (10.4)	29.9 (14.3)	<.001
12	1944	29.8 (11.3)	25.0 (10.3)	28.2 (10.8)	31.5 (15.4)	<.001
18	1182	31.9 (12.1)	25.6 (10.7)	28.1 (10.4)	34.8 (16.4)	<.001
24	329	32.5 (12.2)	28.3 (11.2)	33.8 (10.0)	44.7 (13.1)	<.001

Abbreviations: ADAS-cog, Alzheimer Disease Assessment Scale–cognitive subscale; ChEI, cholinesterase inhibitor.

^a^ ADAS-cog scores range from 0 to 70 errors with higher scores representing worse performance.

**Figure 1.**
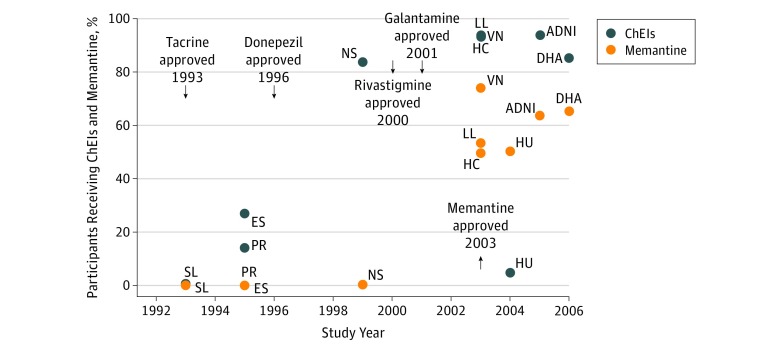
Percentage of Study Participants Receiving ChEIs (With or Without Memantine) and Memantine (With or Without ChEIs), Grouped by Year of Study Initiation ADNI indicates Alzheimer Neuroimaging Initiative; ChEIs, cholinesterase inhibitors; DHA, docosahexaenoic acid; ES, estrogen; HC, homocysteine; HU, huperzine; LL, lipid lowering; NS, nonsteroidal; PR, prednisone; SL, selegiline; and VN, valproate neuroprotection.

Concomitant use of ChEIs in AD clinical trials has steadily increased since the introduction of donepezil in 1996 (Figure 1). In recent trials, approximately 90% of enrolled participants were receiving a ChEI, unless specifically prohibited by the study protocol (as was the case for huperzine, a plant-derived ChEI). Of the 1829 participants who were receiving ChEIs, 1428 participants (78.1%) used donepezil, 308 participants (16.8%) used galantamine, 183 participants (10.0%) used rivastigmine, and 1 participant (0.5%) used tacrine. Notably, an experimental ChEI, metrifonate, was used by 1 (0.5%). The percentages add up to greater than 100% due to the small number (87 [4.5%]) receiving more than 1 ChEI. The use of memantine has also grown since its introduction in 2003, with approximately 50% to 65% of enrolled participants receiving this as a concomitant medication in recent trials (Figure 1).

### Quality of Studies

A funnel plot showed considerable heterogeneity in the annual rate of decline on the ADAS-cog among the studies analyzed (eFigure 1 in the Supplement). There was a smaller rate of decline observed in later studies, but no clear time trend for precision (measurement error). The funnel plot was consistent with our analysis of several large studies with good precision and negative outcomes. The Fail Safe N indicated that 15 nonsignificant, unpublished studies would have to be added to change the results of the meta-analysis from significant to nonsignificant.

### Outcomes

**Figure 2.**
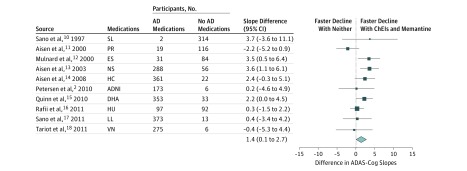
Rates of Decline for Participants Receiving ChEIs, Memantine, or Both Compared With Rates of Decline for Participants Receiving Neither Medication Rates of decline for individual studies were combined using random-effects meta-analysis. Vertical reference line indicates no difference between participants receiving medication and participants not receiving medication; size of squares is proportional to the weight of the study in the analysis. AD indicates Alzheimer disease; ADAS-cog, Alzheimer Disease Assessment Scale–cognitive subscale; ADNI, Alzheimer Neuroimaging Initiative; ChEIs, cholinesterase inhibitors; DHA, docosahexaenoic acid; ES, estrogen; HC, homocysteine; HU, huperzine; LL, lipid lowering; NS, nonsteroidal; PR, prednisone; SL, selegiline; and VN, valproate neuroprotection.

Participants receiving ChEIs or memantine had lower scores on the ADAS-cog at baseline, indicating better cognitive performance (Table). However, mixed-effects modeling showed that participants receiving these concomitant medications had more rapid cognitive decline over time than those not receiving these medications. Meta-analysis of mixed-effects models comparing those receiving ChEIs or memantine or both, vs those receiving neither medication, showed a significantly faster rate of decline in the former of 1.4 points per year (95% CI, 0.1-2.7) (Figure 2). Excluding the observational ADNI study and restricting the analysis to randomized clinical trials yielded a slightly faster rate of decline of 1.5 points per year (95% CI, 0.1-2.8) (eFigure 2 in the Supplement). Effect sizes for the rates of decline are shown in eFigure 3 in the Supplement. Subanalyses comparing those receiving ChEIs only vs those receiving neither ChEIs nor memantine, showed a faster rate of decline in the former of 0.9 points per year, although this was not statistically significant (95% CI, −0.6 to 2.3) (eFigure 4 in the Supplement). In contrast, subanalyses of participants receiving memantine with or without ChEIs vs those receiving ChEIs alone or neither, showed a significantly faster rate of decline in the former of 2.0 points per year (95% CI, 1.3-2.7) (eFigure 5 in the Supplement).

There was no clear trend for the rate of decline on the ADAS-cog to vary over time. Inclusion of the year of study initiation as a covariate in the meta-analysis did not show significant moderating effects for the rate of decline with ChEIs (χ^2^_1_ = 1.240; *P* = .26) or memantine (χ^2^_1_ = 0.90; *P* = .34). Similarly, inclusion of the percentage of participants receiving ChEIs and memantine (which increased over time) as a covariate did not show significant moderating effects for ChEIs (χ^2^_1_ = 0.055; *P* = .80) or memantine (χ^2^_1_ = 0.10; *P* = .25).

## Discussion

This study represents the first broad analysis of the use of concomitant medications (ChEIs and memantine) among participants in AD clinical trials, and the potential implications for concomitant medication use on trial outcomes. This trial found that the use of ChEIs and memantine by AD trial participants is common; in later studies, almost all participants were receiving ChEIs at study entry, and the majority were receiving memantine. These concomitant medications were also being prescribed earlier in the course of the disease, as shown by the lower ADAS-cog scores among participants receiving these medications vs those not receiving them. It is not clear if these lower scores represent an effect of concomitant medications on the course of the disease, or simply prescribing practices that begin medications earlier instead of later in the disease course.

This study also extends the previous results in observational studies to show that participants receiving concomitant medications for AD in clinical trials have a faster rate of decline on the ADAS-cog than those not receiving these medications. The rate of decline for those receiving ChEIs and/or memantine was faster than those not receiving either medication, which was statistically significant with or without inclusion of the observational ADNI study. The rate of decline for those receiving memantine, with or without ChEIs, was faster than those receiving ChEIs only or receiving neither medication, which was statistically significant.

While some may argue that these results demonstrate that ChEIs and memantine are ineffective in long-term treatment for dementia due to AD, it must be borne in mind that there are other possible explanations for our findings. In particular, the use of concomitant medications may represent confounding by indication, in which patients who are perceived as doing worse by their treating physician are the patients who were started on concomitant medications. This confounding by indication does not appear to be due to worse cognitive status, as individuals receiving concomitant medications had lower scores on the ADAS-cog at baseline, but may be related to other factors (such as participants’ subjective sense of worsening cognition) that predict more rapid cognitive decline. Given that participants receiving these concomitant medications had higher levels of education (a proxy for cognitive reserve) at baseline, we could envision that these individuals begin to decline more rapidly as they exhaust their reserve, and are started on these medications based on perceptions that these compensatory mechanisms are failing. In this meta-analysis, the findings for the faster rate of decline among those receiving concomitant medications was not moderated by year of study initiation or by the percentage of trial participants receiving concomitant medications. Thus, the faster rate of decline does not appear to be due to the increased usage of concomitant medications in later trials being confounded with changes in disease severity among enrolled participants in later trials. Although the rapid adoption of concomitant medications among trial participants does complicate the examination of temporal trends in the rate of decline, approximately 10% of participants in later trials were not receiving ChEIs or memantine, which provide a sufficient pool of these individuals in later trials for analysis.

These findings are perhaps more concerning for clinical trials than for observational studies, as the rates of decline for receiving ChEIs, memantine, or both (1.41 ADAS-cog points/y for either medication compared with receiving neither, or 1.97 points/y when receiving or not receiving memantine) could offset any effect of a successful therapeutic intervention. Conversely, these rates of decline could lead to erroneous conclusions that a therapeutic intervention is effective, depending on the distribution of concomitant medication use among groups. The mean treatment effects for ChEIs measured by the ADAS-cog in placebo-controlled trials generally range between 2 and 3 points.^25^ As examples, recent trials of 5-hydroxytryptamine (5-HT_6_) antagonists with negative outcomes that required the use of concomitant ChEIs planned for 2-point differences on the ADAS-cog between drug and placebo.^26^ Notably, trials of the amyloid-β antibody solanezumab, intended for disease modification, were planned to detect 1.8-point differences between drug and placebo on the ADAS-cog.^27^

With randomization, it is expected that there would not be any imbalance in concomitant medication use between treatment and placebo groups in large trials, but this may not necessarily hold for smaller trials. Indeed, our analysis showed that those receiving concomitant medications were more likely to be in the placebo arms of the parent studies. While this likely represents chance findings owing to the small size of many of the included trials, this imbalance would remain a concern for future studies unless specifically considered in the design phase. Imbalance would be an even greater concern when conducting post hoc analyses of completed clinical trials, where unequal distribution of concomitant medications between groups may occur despite randomization at the beginning of the trial. In addition, post hoc analyses based on use of concomitant medications would have inherent biases for cognitive outcomes. Results from trials conducted exclusively among participants receiving ChEIs and/or memantine would likely not extend to patients receiving neither medication, and vice versa. One recent example with this potential bias compared participants receiving concomitant ChEI assigned to the experimental drug with placebo participants not receiving ChEIs or memantine.^28^ Finally, sample size estimates based on previous studies may overestimate the expected effect for future trials if the rates of concomitant medication use differ significantly between the past and future trials.

### Limitations

Our study has several notable strengths, particularly the large sample size collected over a period of nearly 15 years, incorporating changes in prescribing practices over time. We also have a diverse set of studies for meta-analysis, which would make our results more generalizable to future trials. However, there are some limitations that must be acknowledged. Most importantly, we were not able to determine the reasons why participants were or were not started on concomitant medications. Thus, we are unable to fully address the potential issue of confounding by indication discussed previously. Also, although our use of mixed-effects models reflects common practice in the analysis of AD clinical trials, mixed-effects modeling can be affected by the selective drop out of more cognitively impaired participants with AD, so that estimates are only approximately correct. Additionally, we could not rule out overlap of participants between studies, although the number of such occurrences should be low. Our analysis was also based on studies largely conducted at academic medical centers, ie, steering committee members of the Alzheimer Disease Cooperative Study, and may not necessarily reflect the experience of patients outside of this setting. As has been observed for clinical trials in dementia due to AD, our sample was generally highly educated with only a small percentage of racial/ethnic minorities. Further studies are needed to determine if these results extend to other patient populations.

## Conclusions

We have shown that participants receiving concomitant medications for dementia due to AD (particularly memantine added to ChEIs) show faster rate of decline that can exceed the effect of trial interventions. The use of concomitant medications must specifically be accounted for in the design and analysis of trial data to prevent erroneous conclusions that could result from imbalances in the rates of these medications among trial participants.

## Notes

Supplement. **eAppendix 1.** Statistical Analysis **eTable 1.** Placebo-Controlled and Observational Studies Included in the Analyses **eTable 2.** Participant Characteristics by Concomitant Medication Group for the DHA Study Included in Analyses **eTable 3.** Participant Characteristics by Concomitant Medication Group for the ES Study Included in Analyses **eTable 4.** Participant Characteristics by Concomitant Medication Group for the HC Study Included in Analyses **eTable 5.** Participant Characteristics by Concomitant Medication Group for the HU Study Included in Analyses **eTable 6.** Participant Characteristics by Concomitant Medication Group for the LL Study Included in Analyses **eTable 7.** Participant Characteristics by Concomitant Medication Group for the NS Study Included in Analyses **eTable 8.** Participant Characteristics by Concomitant Medication Group for the PR Study Included in Analyses **eTable 9.** Participant Characteristics by Concomitant Medication Group for the SL Study Included in Analyses **eTable 10.** Participant Characteristics by Concomitant Medication Group for the VN Study Included in Analyses **eTable 11.** Participant Characteristics by Concomitant Medication Group for the ADNI Study Included in Analyses **eFigure 1.** Funnel Plot of Estimates (estimated annual rates of decline) vs Precision (standard error) Across All Studies **eFigure 2.** Rates of Decline for Participants Taking ChEIs, Memantine, or Both Compared to Rates of Decline for Participants Taking Neither Medication, Excluding the Observational ADNI Study **eFigure 3.** Effect Sizes for Rates of Decline of Participants Taking ChEIs, Memantine, or Both Compared to Rates of Decline for Participants Taking Neither Medication, Excluding the Observational ADNI Study **eFigure 4.** Rates of Decline for Participants Taking ChEIs Only Compared to Rates of Decline for Participants Taking Neither ChEIs Nor Memantine **eFigure 5.** Rates of Decline for Participants Taking Memantine or Both Memantine and ChEIs Compared to Rates of Decline for Participants Taking ChEIs or Neither **eReferences** **eAppendix 2.** Association of Concomitant Use of Cholinesterase Inhibitors or Memantine With Cognitive Decline in Alzheimer Clinical Trials: Source Code and Output
