# Supplementary material for: Association of Concomitant Use of Cholinesterase Inhibitors or Memantine With Cognitive Decline in Alzheimer Clinical Trials: A Meta-analysis
Source: JAMA Netw Open. 2018 Nov 2;1(7):e184080. doi: 10.1001/jamanetworkopen.2018.4080 (PMC6324361; doi:10.1001/jamanetworkopen.2018.4080)
Supplement: Supplement. — eAppendix 1. Statistical Analysis eTable 1. Placebo-Controlled and Observational Studies Included in the Analyses eTable 2. Participant Characteristics by Concomitant Medication Group for the DHA Study Included in Analyses eTable 3. Participant Characteristics by Concomitant Medication Group for the ES Study Included in Analyses eTable 4. Participant Characteristics by Concomitant Medication Group for the HC Study Included in Analyses eTable 5. Participant Characteristics by Concomitant Medication Group for the HU Study Included in Analyses eTable 6. Participant Characteristics by Concomitant Medication Group for the LL Study Included in Analyses eTable 7. Participant Characteristics by Concomitant Medication Group for the NS Study Included in Analyses eTable 8. Participant Characteristics by Concomitant Medication Group for the PR Study Included in Analyses eTable 9. Participant Characteristics by Concomitant Medication Group for the SL Study Included in Analyses eTable 10. Participant Characteristics by Concomitant Medication Group for the VN Study Included in Analyses eTable 11. Participant Characteristics by Concomitant Medication Group for the ADNI Study Included in Analyses eFigure 1. Funnel Plot of Estimates (estimated annual rates of decline) vs Precision (standard error) Across All Studies eFigure 2. Rates of Decline for Participants Taking ChEIs, Memantine, or Both Compared to Rates of Decline for Participants Taking Neither Medication, Excluding the Observational ADNI Study eFigure 3. Effect Sizes for Rates of Decline of Participants Taking ChEIs, Memantine, or Both Compared to Rates of Decline for Participants Taking Neither Medication, Excluding the Observational ADNI Study eFigure 4. Rates of Decline for Participants Taking ChEIs Only Compared to Rates of Decline for Participants Taking Neither ChEIs Nor Memantine eFigure 5. Rates of Decline for Participants Taking Memantine or Both Memantine and ChEIs Compared to Rates of Decline for Participants Taki [file jamanetwopen-1-e184080-s001.pdf]

Supplementary Online Content

Kennedy RE, Cutter GR, Fowler ME, Schneider LS. Association of concomitant use of cholinesterase inhibitors or memantine with cognitive decline in Alzheimer clinical trials: a meta-analysis. *JAMA Netw Open*. 2018;1(7):e184080. doi:10.1001/jamanetworkopen.2018.4080

eAppendix 1. Statistical Analysis

eTable 1. Placebo-Controlled and Observational Studies Included in the Analyses

eTable 2. Participant Characteristics by Concomitant Medication Group for the DHA Study Included in Analyses

eTable 3. Participant Characteristics by Concomitant Medication Group for the ES Study Included in Analyses

eTable 4. Participant Characteristics by Concomitant Medication Group for the HC Study Included in Analyses

eTable 5. Participant Characteristics by Concomitant Medication Group for the HU Study Included in Analyses

eTable 6. Participant Characteristics by Concomitant Medication Group for the LL Study Included in Analyses

eTable 7. Participant Characteristics by Concomitant Medication Group for the NS Study Included in Analyses

eTable 8. Participant Characteristics by Concomitant Medication Group for the PR Study Included in Analyses

eTable 9. Participant Characteristics by Concomitant Medication Group for the SL Study Included in Analyses

eTable 10. Participant Characteristics by Concomitant Medication Group for the VN Study Included in Analyses

eTable 11. Participant Characteristics by Concomitant Medication Group for the ADNI Study Included in Analyses

eFigure 1. Funnel Plot of Estimates (estimated annual rates of decline) vs Precision (standard error) Across All Studies

© 2018 Kennedy RE et al. *JAMA Network Open*.

eFigure 2. Rates of Decline for Participants Taking ChEIs, Memantine, or Both Compared to Rates of Decline for Participants Taking Neither Medication, Excluding the Observational ADNI Study

eFigure 3. Effect Sizes for Rates of Decline of Participants Taking ChEIs, Memantine, or Both Compared to Rates of Decline for Participants Taking Neither Medication, Excluding the Observational ADNI Study

eFigure 4. Rates of Decline for Participants Taking ChEIs Only Compared to Rates of Decline for Participants Taking Neither ChEIs Nor Memantine

eFigure 5. Rates of Decline for Participants Taking Memantine or Both Memantine and ChEIs Compared to Rates of Decline for Participants Taking ChEIs or Neither

eReferences

eAppendix 2. Association of Concomitant Use of Cholinesterase Inhibitors or Memantine With Cognitive Decline in Alzheimer Clinical Trials: Source Code and Output

This supplementary material has been provided by the authors to give readers additional information about their work.

© 2018 Kennedy RE et al. *JAMA Network Open*.

eAppendix 1. Statistical Analysis

Mixed effects linear models[1] were used to estimate the slopes (i.e., rates of change in points per year) of the ADAS-cog between medication groups (ChEIs, memantine, both, and neither) for each study individually. The group taking neither medication was used as the reference group in the primary comparisons. Additional analyses compared rates for individuals taking ChEIs to rates for individuals taking neither, and rates for individuals taking memantine or both to rates for individuals taking ChEIs or neither. The full model was constructed with medication group and time effects and group by time interactions, with age and education as covariates, and the reduced model with time, group, and age and education effects. Thus, for participant $i = 1,2,\ldots,n$ at time $j = 1,2,\ldots,n_i$, the full model was

$$ADAS_{i,j} = age_i + education_i + group_i + time_{i,j} + group_i \cdot time_{i,j} + \varepsilon_{i,j}$$

and the reduced model was

$$ADAS_{i,j} = age_i + education_i + group_i + time_{i,j} + \varepsilon_{i,j}$$

where the model includes both fixed effects of time at the group level and random effects of time at the individual level. An unstructured covariance matrix was used to model independence of the slope and intercept parameters. Parameters were estimated using maximum likelihood. P-values for the group by time interaction were found using -2 times the difference in the log likelihood of the full and reduced model, which follows a chi-squared distribution. Mixed effects models were performed using the *lme4* package[2] in the R statistical environment version 3.5.0.[3] Effect sizes (Cohen's *d*) were calculated from the mixed model *t* statistics using the *EMAtools* package for R.

© 2018 Kennedy RE et al. *JAMA Network Open*.

eTable 1. Placebo-Controlled and Observational Studies Included in the Analyses

**Study (code), dates**	**Design**	**Intervention**	**N**	**Duration (months)**	**Minimal Severity**
English Instruments (EIN) 1993-1995[4]	Observational, AD, NL	None	306	12	CDR ≥ 1 (AD)<br>CDR 0 (NL)
Selegiline, vitamin E (SL) 1993-1996[5]	RCT, moderate to severe AD	Vitamin E, selegiline	341	24	CDR ≥ 2
Agitation (AP) 1994-1996[6]	RCT, AD	Haloperidol, Trazodone, Behavior Techniques	149	24	None
Prednisone (PR) 1995-1998[7]	RCT, mild to moderate AD	Prednisone	138	16	MMSE 13-26
Conjugated estrogens (ES) 1995-1999[8]	RCT, mild to moderate AD	Conjugated estrogens	120	15	MMSE 12-28
Melatonin (ML) 1997-2000[9]	RCT, mild to moderate AD	Melatonin	157	2	MMSE 0-26
Non-steroidal anti-inflammatory (NS) 1999-2001[10]	RCT, mild to moderate AD	Rofecoxib, naproxen	351	12	MMSE 13-26
Memory impairment study (MIS), donepezil, vitamin E, 1999-2004[11]	RCT, MCI	Donepezil, vitamin E	769	36	MMSE 24-30<br>CDR 0.5
Normal Control (NL) 1999-2004; not yet published	Observational, NL	None	100	36	MMSE 24-30
Valproate (VP) 2000-2002[12]	RCT, AD	Divalproex	153	3	MMSE 4-24
Prevention Instruments (PI) 2002-2006[13]	Observational, MCI, NL	None	662	48	MMSE 26-30

© 2018 Kennedy RE et al. *JAMA Network Open*.

Simvastatin (LL) 2003-2008[14]	RCT, mild to moderate AD	Simvastatin	406	18	MMSE 12-26
Valproate Neuroprotection (VN) 2003-2009[15]	RCT, moderate AD	Divalproex	313	24	MMSE 12-20
Vitamins B (HC) 2003-2007[16]	RCT, mild to moderate AD	B vitamins	409	18	MMSE 14-26
Huperzine (HU) 2004-2007[17]	RCT, mild to moderate AD	Huperzine A	210	6	MMSE 10-24
Docosahexaenoic acid (DHA) 2006-2009[18]	RCT, mild to moderate AD	Docosahexaenoic acid	402	18	MMSE 14-26
Alzheimer's Disease Neuroimaging Initiative (ADNI) 2005-2010[19]	Observational, AD, MCI, normal	None	800 (192 AD, 398 MCI, 229 normal)	36 (AD)	MMSE 20-26 (AD)<br>24-30 (MCI)<br>CDR 0 (NL)<br>0.5 (MCI)<br>0.5-1 (AD)

Studies were drawn from the Alzheimer's Disease Cooperative Study (ADCS; http://www.adcs.org) and the Alzheimer's Disease Neuroimaging Initiative (ADNI; http://adni.loni.ucla.edu) and included those with baseline demographic data and information on comorbid medical disorders. RCT=randomized controlled trial; NL=normal control (who were not included in analyses).

© 2018 Kennedy RE et al. *JAMA Network Open*.

eTable 2. Participant Characteristics by Concomitant Medication Group for the DHA Study Included in Analyses

	N	Both N=228	ChEI N=101	Memantine N=24	Neither N=33	P Value
Age, Years	386	75.1 (8.84)	76.6 (8.24)	78.5 (5.64)	78.4 (8.71)	0.052
Education	386					0.162
Less Than HS		14 (6.14%)	12 (11.9%)	5 (20.8%)	3 (9.09%)	
HS Graduate		112 (49.1%)	53 (52.5%)	9 (37.5%)	16 (48.5%)	
College Graduate		102 (44.7%)	36 (35.6%)	10 (41.7%)	14 (42.4%)	
Race, White	386	211 (92.5%)	92 (91.1%)	22 (91.7%)	28 (84.8%)	0.471
Ethnicity, Hispanic	386	5 (2.19%)	3 (2.97%)	2 (8.33%)	4 (12.1%)	0.022
Sex, Female	386	122 (53.5%)	56 (55.4%)	13 (54.2%)	12 (36.4%)	0.271
Married	386	169 (74.1%)	68 (67.3%)	17 (70.8%)	22 (66.7%)	0.569
Treatment Arm, Placebo	386	102 (44.7%)	31 (30.7%)	12 (50.0%)	14 (42.4%)	0.087
ADAS-Cog, Baseline	386	25.3 (8.76)	21.2 (6.97)	25.0 (9.81)	17.8 (8.30)	<0.001
ADAS-Cog, 6 months	340	27.9 (9.89)	22.3 (8.24)	29.1 (9.38)	18.1 (11.4)	<0.001
ADAS-Cog, 12 months	300	29.6 (11.2)	23.5 (9.31)	28.1 (10.1)	17.0 (12.3)	<0.001
ADAS-Cog, 18 months	264	31.0 (12.0)	26.8 (11.8)	27.4 (11.6)	20.4 (14.0)	<0.001

© 2018 Kennedy RE et al. *JAMA Network Open*.

eTable 3. Participant Characteristics by Concomitant Medication Group for the ES Study Included in Analyses

	N	ChEI N=31	Neither N=84	P Value
Age, Years	115	74.3 (7.84)	75.6 (6.43)	0.419
Education	115			0.127
Less Than HS		4 (12.9%)	26 (31.0%)	
HS Graduate		21 (67.7%)	42 (50.0%)	
College Graduate		6 (19.4%)	16 (19.0%)	
Race, White	115	30 (96.8%)	72 (85.7%)	0.181
Ethnicity, Hispanic	115	0 (0.00%)	4 (4.76%)	0.573
Sex, Female	115	31 (100%)	84 (100%)	.
Married	115	16 (51.6%)	36 (42.9%)	0.531
Treatment Arm, Placebo	115	7 (22.6%)	30 (35.7%)	0.266
ADAS-Cog, Baseline	115	24.1 (9.08)	24.3 (9.22)	0.923
ADAS-Cog, 6 months	97	27.8 (10.1)	27.1 (9.40)	0.756
ADAS-Cog, 12 months	90	32.4 (14.2)	27.4 (10.5)	0.118

© 2018 Kennedy RE et al. *JAMA Network Open*.

eTable 4. Participant Characteristics by Concomitant Medication Group for the HC Study Included in Analyses

	**N**	**Both N=186**	**ChEI N=171**	**Memantine N=4**	**Neither N=22**	**P Value**
Age, Years	383	75.7 (8.01)	77.4 (7.29)	82.0 (8.91)	73.6 (8.42)	0.024
Education	383					0.238
Less Than HS		17 (9.14%)	26 (15.2%)	0 (0.00%)	6 (27.3%)	
HS Graduate		95 (51.1%)	81 (47.4%)	2 (50.0%)	10 (45.5%)	
College Graduate		74 (39.8%)	64 (37.4%)	2 (50.0%)	6 (27.3%)	
Race, White	383	171 (91.9%)	147 (86.0%)	4 (100%)	9 (40.9%)	<0.001
Ethnicity, Hispanic	383	7 (3.76%)	14 (8.19%)	0 (0.00%)	0 (0.00%)	0.244
Sex, Female	383	98 (52.7%)	97 (56.7%)	3 (75.0%)	16 (72.7%)	0.277
Married	383	141 (75.8%)	106 (62.0%)	3 (75.0%)	11 (50.0%)	0.006
Treatment Arm, Placebo	383	78 (41.9%)	68 (39.8%)	2 (50.0%)	7 (31.8%)	0.781
ADAS-Cog, Baseline	383	24.6 (8.93)	21.0 (8.05)	20.5 (3.16)	15.0 (7.73)	<0.001
ADAS-Cog, 6 months	346	26.4 (9.99)	21.5 (7.61)	22.3 (2.09)	14.6 (8.21)	<0.001
ADAS-Cog, 12 months	324	29.2 (10.3)	22.3 (8.25)	24.8 (5.53)	17.1 (8.28)	<0.001
ADAS-Cog, 18 months	299	31.4 (12.3)	23.6 (10.1)	26.9 (8.47)	16.3 (8.41)	<0.001

© 2018 Kennedy RE et al. *JAMA Network Open*.

eTable 5. Participant Characteristics by Concomitant Medication Group for the HU Study Included in Analyses

	N	Both N=7	ChEI N=2	Memantine N=88	Neither N=92	P Value
Age, Years	189	77.9 (6.36)	78.0 (2.83)	77.8 (7.54)	79.0 (8.21)	0.762
Education	189					0.310
Less Than HS		2 (28.6%)	0 (0.00%)	11 (12.5%)	15 (16.3%)	
HS Graduate		3 (42.9%)	1 (50.0%)	46 (52.3%)	57 (62.0%)	
College Graduate		2 (28.6%)	1 (50.0%)	31 (35.2%)	20 (21.7%)	
Race, White	189	7 (100%)	1 (50.0%)	82 (93.2%)	86 (93.5%)	0.261
Ethnicity, Hispanic	189	0 (0.00%)	0 (0.00%)	5 (5.68%)	6 (6.52%)	1.000

© 2018 Kennedy RE et al. *JAMA Network Open*.

Sex, Female	189	3 (42.9%)	1 (50.0%)	57 (64.8%)	55 (59.8%)	0.598
Married	189	7 (100%)	1 (50.0%)	63 (71.6%)	62 (67.4%)	0.238
Treatment Arm, Placebo	189	3 (42.9%)	0 (0.00%)	35 (39.8%)	28 (30.4%)	0.406
ADAS-Cog, Baseline	189	30.2 (7.96)	23.7 (0.00)	29.9 (11.7)	23.2 (10.0)	0.001
ADAS-Cog, 6 months	141	26.2 (7.45)	24.3 (0.94)	26.2 (11.0)	20.6 (9.34)	0.015
ADAS-Cog, 12 months	111	26.5 (4.13)	22.0 (.)	27.8 (11.4)	21.2 (11.2)	0.028
ADAS-Cog, 18 months	29	27.2 (2.04)	30.7 (.)	25.9 (8.57)	21.5 (10.8)	0.554

© 2018 Kennedy RE et al. *JAMA Network Open*.

eTable 6. Participant Characteristics by Concomitant Medication Group for the LL Study Included in Analyses

	N	Both N=195	ChEI N=167	Memantine N=11	Neither N=13	P Value
Age, Years	386	72.6 (9.85)	75.5 (8.31)	79.8 (8.13)	73.2 (9.33)	0.004
Education	386					0.084
Less Than HS		12 (6.15%)	24 (14.4%)	1 (9.09%)	1 (7.69%)	
HS Graduate		89 (45.6%)	83 (49.7%)	4 (36.4%)	6 (46.2%)	
College Graduate		94 (48.2%)	60 (35.9%)	6 (54.5%)	6 (46.2%)	
Race, White	386	179 (91.8%)	153 (91.6%)	10 (90.9%)	10 (76.9%)	0.275
Ethnicity, Hispanic	386	12 (6.15%)	12 (7.19%)	0 (0.00%)	0 (0.00%)	0.966
Sex, Female	386	104 (53.3%)	108 (64.7%)	10 (90.9%)	6 (46.2%)	0.014
Married	386	151 (77.4%)	112 (67.1%)	6 (54.5%)	7 (53.8%)	0.027
Treatment Arm, Placebo	386	94 (48.2%)	86 (51.5%)	8 (72.7%)	5 (38.5%)	0.339
ADAS-Cog, Baseline	386	26.1 (10.5)	21.6 (8.93)	28.2 (9.81)	20.8 (6.72)	<0.001
ADAS-Cog, 6 months	326	28.5 (10.9)	21.7 (9.55)	29.4 (11.8)	22.1 (6.73)	<0.001
ADAS-Cog, 12 months	293	31.0 (12.6)	22.3 (8.54)	34.5 (11.1)	26.3 (10.4)	<0.001
ADAS-Cog, 18 months	252	31.7 (12.8)	24.9 (9.37)	35.0 (19.6)	27.7 (9.40)	<0.001

© 2018 Kennedy RE et al. *JAMA Network Open*.

eTable 7. Participant Characteristics by Concomitant Medication Group for the NS Study Included in Analyses

	N	Both N=1	ChEI N=287	Neither N=56	P Value
Age, Years	344	55.0 (.)	73.5 (7.60)	76.3 (7.50)	0.002
Education	344				0.198
Less Than HS		0 (0.00%)	34 (11.8%)	12 (21.4%)	
HS Graduate		0 (0.00%)	141 (49.1%)	25 (44.6%)	
College Graduate		1 (100%)	112 (39.0%)	19 (33.9%)	
Race, White	344	1 (100%)	262 (91.3%)	48 (85.7%)	0.290
Ethnicity, Hispanic	344	0 (0.00%)	13 (4.53%)	6 (10.7%)	0.150
Sex, Female	344	1 (100%)	148 (51.6%)	34 (60.7%)	0.243
Married	344	0 (0.00%)	229 (79.8%)	35 (62.5%)	0.003
Treatment Arm, Placebo	344	1 (100%)	92 (32.1%)	17 (30.4%)	0.425
ADAS-Cog, Baseline	344	45.3 (.)	24.2 (9.55)	22.3 (9.64)	0.034
ADAS-Cog, 6 months	269	. (.)	26.3 (10.8)	20.8 (11.7)	0.005
ADAS-Cog, 12 months	231	. (.)	27.6 (10.6)	21.5 (10.9)	0.002

© 2018 Kennedy RE et al. *JAMA Network Open*.

eTable 8. Participant Characteristics by Concomitant Medication Group for the PR Study Included in Analyses

	N	ChEI N=19	Neither N=116	P Value
Age, Years	135	72.6 (5.13)	72.3 (7.94)	0.838
Education	135			0.510
Less Than HS		1 (5.26%)	14 (12.1%)	
HS Graduate		13 (68.4%)	61 (52.6%)	
College Graduate		5 (26.3%)	41 (35.3%)	
Race, White	135	19 (100%)	110 (94.8%)	0.594
Ethnicity, Hispanic	135	0 (0.00%)	5 (4.31%)	1.000
Sex, Female	135	12 (63.2%)	56 (48.3%)	0.339
Married	135	16 (84.2%)	97 (83.6%)	1.000
Treatment Arm, Placebo	135	9 (47.4%)	58 (50.0%)	1.000
ADAS-Cog, Baseline	135	21.3 (8.05)	21.9 (10.2)	0.756
ADAS-Cog, 6 months	117	24.1 (11.2)	24.1 (11.6)	0.994
ADAS-Cog, 12 months	99	27.1 (12.9)	27.6 (12.9)	0.872
ADAS-Cog, 18 months	87	25.6 (12.6)	27.7 (13.7)	0.540

© 2018 Kennedy RE et al. *JAMA Network Open*.

eTable 9. Participant Characteristics by Concomitant Medication Group for the SL Study Included in Analyses

	N	ChEI N=2	Neither N=314	P Value
Age, Years	316	61.0 (0.00)	73.0 (8.01)	<0.001
Education	316			0.042
Less Than HS		0 (0.00%)	90 (28.7%)	
HS Graduate		0 (0.00%)	161 (51.3%)	
College Graduate		2 (100%)	63 (20.1%)	
Race, White	316	1 (50.0%)	277 (88.2%)	0.226
Ethnicity, Hispanic	316	1 (50.0%)	12 (3.82%)	0.081
Sex, Female	316	1 (50.0%)	205 (65.3%)	1.000
Married	316	2 (100%)	228 (72.6%)	1.000
Treatment Arm, Placebo	316	1 (50.0%)	77 (24.5%)	0.433
ADAS-Cog, Baseline	316	28.8 (3.06)	39.1 (10.8)	0.111
ADAS-Cog, 6 months	238	29.5 (1.18)	40.4 (12.0)	0.001
ADAS-Cog, 12 months	180	44.0 (3.30)	43.5 (12.5)	0.862
ADAS-Cog, 18 months	129	50.0 (.)	44.6 (12.8)	.
ADAS-Cog, 24 months	98	41.0 (.)	45.5 (12.9)	.

© 2018 Kennedy RE et al. *JAMA Network Open*.

eTable 10. Participant Characteristics by Concomitant Medication Group for the VN Study Included in Analyses

	N	Both N=197	ChEI N=67	Memantine N=11	Neither N=6	P Value
Age, Years	281	74.8 (8.36)	77.9 (6.55)	76.2 (5.98)	79.2 (5.78)	0.026
Education	281					0.251
Less Than HS		22 (11.2%)	16 (23.9%)	1 (9.09%)	0 (0.00%)	
HS Graduate		95 (48.2%)	30 (44.8%)	6 (54.5%)	3 (50.0%)	
College Graduate		80 (40.6%)	21 (31.3%)	4 (36.4%)	3 (50.0%)	
Race, White	281	180 (91.4%)	60 (89.6%)	11 (100%)	6 (100%)	0.798
Ethnicity, Hispanic	281	6 (3.05%)	2 (2.99%)	2 (18.2%)	0 (0.00%)	0.137
Sex, Female	281	108 (54.8%)	44 (65.7%)	6 (54.5%)	4 (66.7%)	0.439
Married	281	148 (75.1%)	42 (62.7%)	10 (90.9%)	5 (83.3%)	0.114
Treatment Arm, Placebo	281	103 (52.3%)	32 (47.8%)	6 (54.5%)	4 (66.7%)	0.823
ADAS-Cog, Baseline	281	30.1 (9.18)	26.4 (8.62)	30.8 (8.05)	30.6 (9.48)	0.030
ADAS-Cog, 6 months	197	31.9 (9.13)	27.8 (8.62)	33.3 (7.46)	30.5 (4.76)	0.042
ADAS-Cog, 12 months	166	35.0 (10.2)	30.0 (11.7)	33.5 (10.4)	34.1 (1.85)	0.087
ADAS-Cog, 18 months	122	35.3 (10.5)	32.3 (9.96)	31.2 (3.57)	40.2 (2.59)	0.300
ADAS-Cog, 24 months	104	36.8 (10.7)	30.2 (10.6)	38.0 (8.82)	35.2 (9.67)	0.048

© 2018 Kennedy RE et al. *JAMA Network Open*.

eTable 11. Participant Characteristics by Concomitant Medication Group for the ADNI Study Included in Analyses

	N	Both N=109	ChEI N=59	Memantine N=5	Neither N=6	P Value
Age, Years	179	74.1 (8.15)	76.9 (6.23)	78.0 (9.43)	77.3 (5.09)	0.080
Education	179					0.015
Less Than HS		5 (4.59%)	9 (15.3%)	2 (40.0%)	2 (33.3%)	
HS Graduate		43 (39.4%)	24 (40.7%)	2 (40.0%)	2 (33.3%)	
College Graduate		61 (56.0%)	26 (44.1%)	1 (20.0%)	2 (33.3%)	
Race, White	179	102 (93.6%)	56 (94.9%)	5 (100%)	5 (83.3%)	0.608
Ethnicity, Hispanic	179	2 (1.83%)	0 (0.00%)	0 (0.00%)	1 (16.7%)	0.168
Sex, Female	179	42 (38.5%)	31 (52.5%)	5 (100%)	5 (83.3%)	0.003
Married	179	98 (89.9%)	45 (76.3%)	2 (40.0%)	2 (33.3%)	<0.001
Treatment Arm, Placebo	179	109 (100%)	59 (100%)	5 (100%)	6 (100%)	.
ADAS-Cog, Baseline	179	19.2 (6.29)	17.8 (5.85)	20.7 (9.29)	17.9 (9.95)	0.499
ADAS-Cog, 6 months	169	21.5 (7.89)	19.7 (7.30)	20.7 (7.94)	20.2 (12.6)	0.607
ADAS-Cog, 12 months	150	23.0 (8.98)	21.7 (8.74)	21.2 (7.11)	23.2 (15.2)	0.856
ADAS-Cog, 24 months	127	28.9 (12.3)	26.7 (11.5)	26.5 (8.17)	30.2 (13.2)	0.795

© 2018 Kennedy RE et al. *JAMA Network Open*.

eFigure 1. Funnel Plot of Estimates (estimated annual rates of decline) vs Precision (standard error) Across All Studies

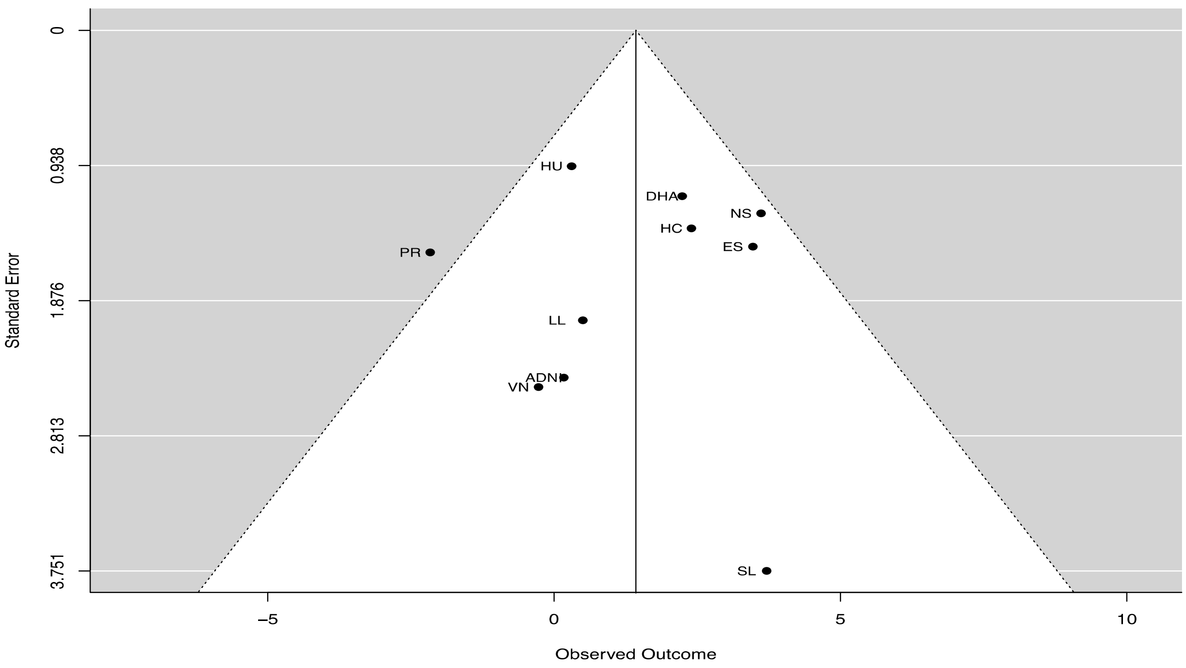

One study (PR) was identified as an outlier but was not excluded from analysis. Although there was considerable heterogeneity among studies, no evidence of low precision studies favoring one group being excluded from publication was seen for rates of progression.

© 2018 Kennedy RE et al. *JAMA Network Open*.

eFigure 2. Rates of Decline for Participants Taking ChEIs, Memantine, or Both Compared to Rates of Decline for Participants Taking Neither Medication, Excluding the Observational ADNI Study

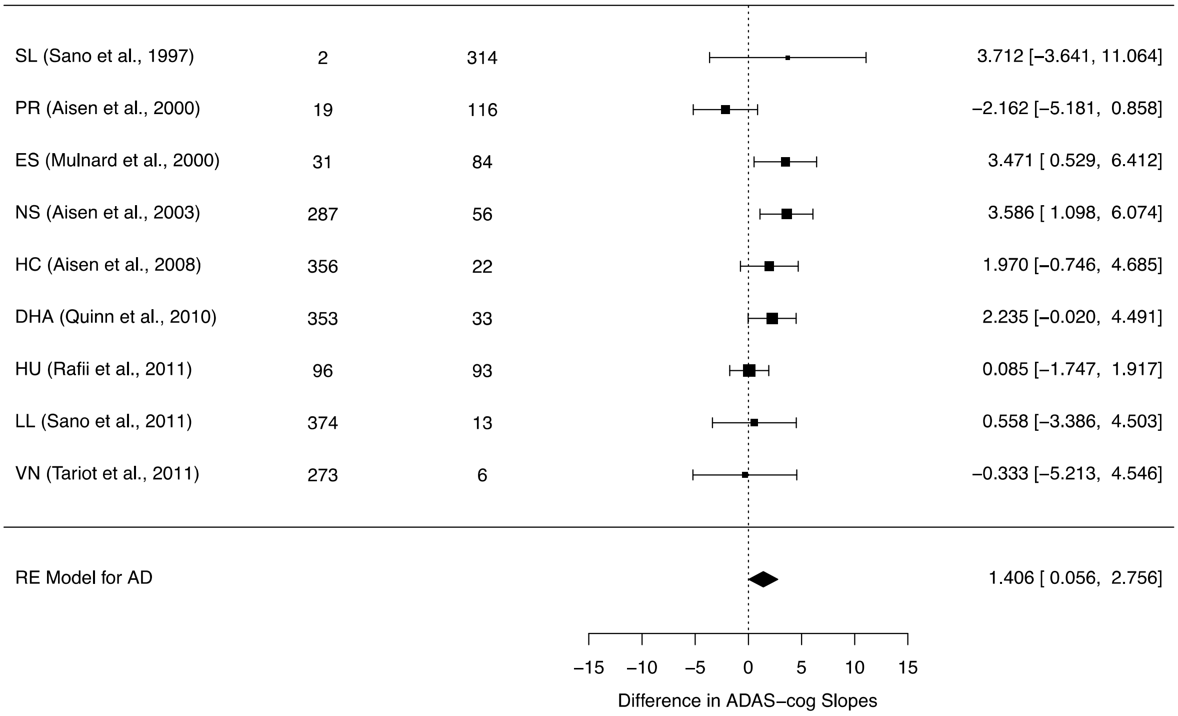

Rates of decline for individual studies were combined using random effects meta-analysis. Vertical reference line indicates no difference between participants taking medication and participants not taking medication; size of squares is proportional to the weight of the study in the analysis. AD=Alzheimer’s disease; ADNI=Alzheimer’s Disease Neuroimaging Initiative; CI=confidence interval.

© 2018 Kennedy RE et al. *JAMA Network Open*.

eFigure 3. Effect Sizes for Rates of Decline of Participants Taking ChEIs, Memantine, or Both Compared to Rates of Decline for Participants Taking Neither Medication, Excluding the Observational ADNI Study

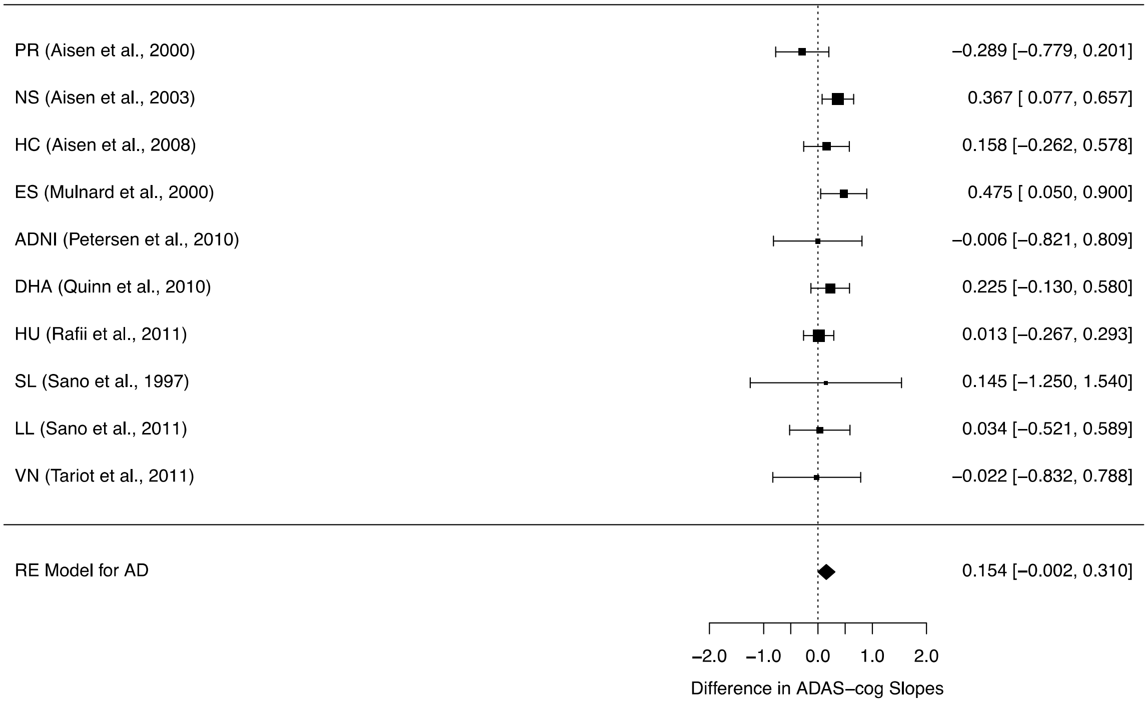

Effect sizes for individual studies were combined using random effects meta-analysis. Vertical reference line indicates no difference between participants taking medication and participants not taking medication; size of squares is proportional to the weight of the study in the analysis. AD=Alzheimer's disease; ADNI=Alzheimer's Disease Neuroimaging Initiative; CI=confidence interval.

© 2018 Kennedy RE et al. *JAMA Network Open*.

eFigure 4. Rates of Decline for Participants Taking ChEIs Only Compared to Rates of Decline for Participants Taking Neither ChEIs Nor Memantine

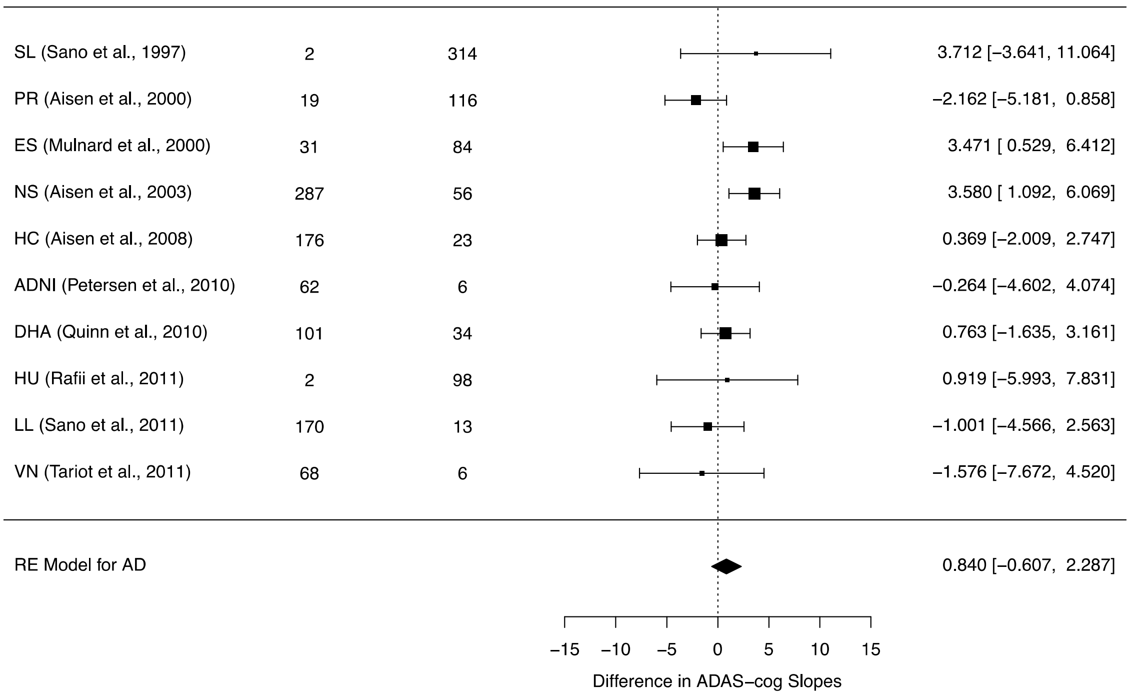

Rates of decline for individual studies were combined using random effects meta-analysis. Vertical reference line indicates no difference between participants taking medication and participants not taking medication; size of squares is proportional to the weight of the study in the analysis. AD=Alzheimer's disease; CI=confidence interval.

© 2018 Kennedy RE et al. *JAMA Network Open*.

eFigure 5. Rates of Decline for Participants Taking Memantine or Both Memantine and ChEIs Compared to Rates of Decline for Participants Taking ChEIs or Neither

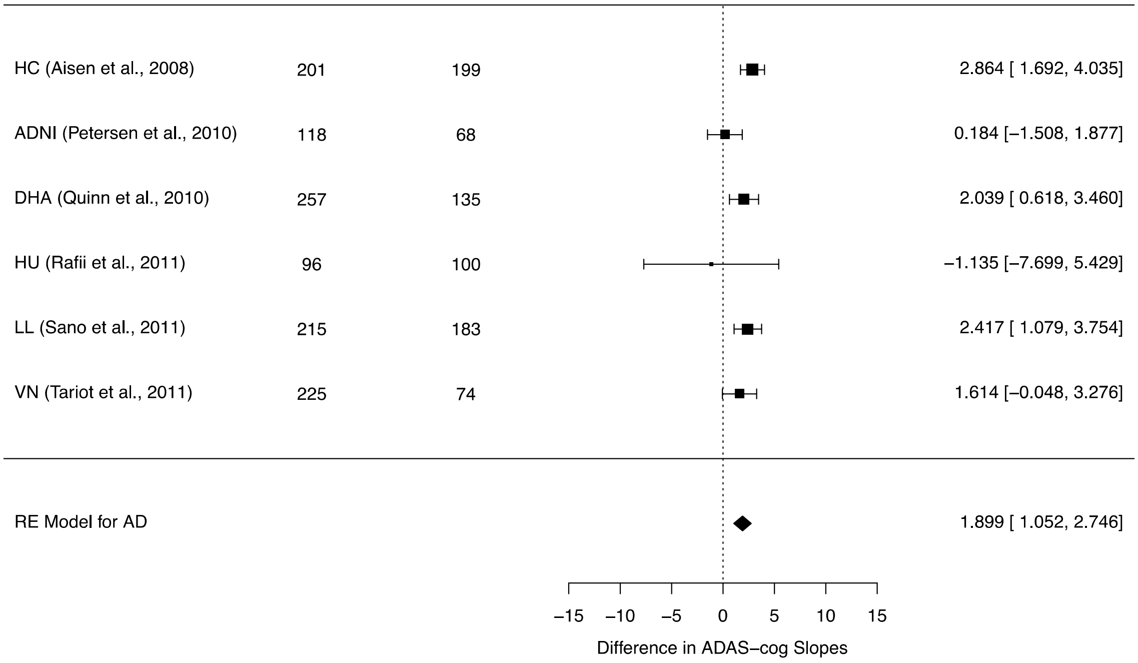

Rates of decline for individual studies were combined using random effects meta-analysis. Vertical reference line indicates no difference between participants taking medication and participants not taking medication; size of squares is proportional to the weight of the study in the analysis. AD=Alzheimer's disease; CI=confidence interval.

© 2018 Kennedy RE et al. *JAMA Network Open*.

eReferences

1. Brown H, Prescott R. *Applied Mixed Models in Medicine.* 2nd ed. Chichester: Wiley; 2006.
2. Bates D, Mächler M, Bolker B, Walker S. Fitting Linear Mixed-Effects Models Using lme4. *J Stat Softw.* 2015;67(1):1-48.
3. *R: A Language and Environment for Statistical Computing* [computer program]. Vienna, Austria: R Foundation for Statistical Computing; 2016.
4. Ferris SH, Mackell JA, Mohs R, et al. A multicenter evaluation of new treatment efficacy instruments for Alzheimer's disease clinical trials: overview and general results. *Alzheimer Dis Assoc Disord.* 1997;11 Suppl 2:S1-12.
5. Sano M, Ernesto C, Thomas RG, et al. A controlled trial of selegiline, alpha-tocopherol, or both as treatment for Alzheimer's disease. *N Engl J Med.* 1997;336(17):1216-1222.
6. Teri L, Logsdon RG, Peskind E, et al. Treatment of agitation in AD: a randomized, placebo-controlled clinical trial. *Neurology.* 2000;55(9):1271-1278.
7. Aisen PS, Davis KL, Berg JD, et al. A randomized controlled trial of prednisone in Alzheimer's disease. *Neurology.* 2000;54(3):588-593.
8. Mulnard RA, Cotman CW, Kawas C, et al. Estrogen replacement therapy for treatment of mild to moderate Alzheimer disease: a randomized controlled trial. *JAMA.* 2000;283(8):1007-1015.
9. Singer C, Tractenberg RE, Kaye J, et al. A multicenter, placebo-controlled trial of melatonin for sleep disturbance in Alzheimer's disease. *Sleep.* 2003;26(7):893-901.

© 2018 Kennedy RE et al. *JAMA Network Open*.

10. Aisen PS, Schafer KA, Grundman M, et al. Effects of rofecoxib or naproxen vs placebo on Alzheimer disease progression: a randomized controlled trial. *JAMA.* 2003;289(21):2819-2826.
11. Petersen RC, Thomas RG, Grundman M, et al. Vitamin E and donepezil for the treatment of mild cognitive impairment. *N Engl J Med.* 2005;352(23):2379-2388.
12. Tariot PN, Raman R, Jakimovich L, et al. Divalproex sodium in nursing home residents with possible or probable Alzheimer disease complicated by agitation: a randomized, controlled trial. *Am J Geriatr Psychiatry.* 2005;13(11):942-949.
13. Ferris SH, Aisen PS, Cummings J, et al. ADCS Prevention Instrument Project: overview and initial results. *Alzheimer Dis Assoc Disord.* 2006;20(4 Suppl 3):S109-123.
14. Sano M, Bell KL, Galasko D, et al. A randomized, double-blind, placebo-controlled trial of simvastatin to treat Alzheimer disease. *Neurology.* 2011;77(6):556-563.
15. Tariot PN, Schneider LS, Cummings J, et al. Chronic divalproex sodium to attenuate agitation and clinical progression of Alzheimer disease. *Arch Gen Psychiatry.* 2011;68(8):853-861.
16. Aisen PS, Schneider LS, Sano M, et al. High-dose B vitamin supplementation and cognitive decline in Alzheimer disease: a randomized controlled trial. *JAMA.* 2008;300(15):1774-1783.
17. Rafii MS, Walsh S, Little JT, et al. A phase II trial of huperzine A in mild to moderate Alzheimer disease. *Neurology.* 2011;76(16):1389-1394.

18. Quinn JF, Raman R, Thomas RG, et al. Docosahexaenoic acid supplementation and cognitive decline in Alzheimer disease: a randomized trial. *JAMA.* 2010;304(17):1903-1911.

19. Petersen RC, Aisen PS, Beckett LA, et al. Alzheimer's Disease Neuroimaging Initiative (ADNI): clinical characterization. *Neurology.* 2010;74(3):201-209.

© 2018 Kennedy RE et al. *JAMA Network Open*.

eAppendix 2. Association of Concomitant Use of Cholinesterase Inhibitors or Memantine With Cognitive Decline in Alzheimer Clinical Trials: Source Code and Output

BIOSTATISTICIAN: RICHARD KENNEDY

AUGUST 14, 2018

Rates of Decline Across Studies

Mixed Effects Model

```
## Study Name
## [1] "ADNI"
## Full Mixed Effects Model
## Linear mixed model fit by maximum likelihood  ['lmerMod']
## Formula: ADAS ~ AGE + EDUCATION + group + month + group:month + (month |
##     UID)
##    Data: mixed.data
##
##      AIC      BIC   logLik deviance df.resid
##   3946.4   3990.8  -1963.2   3926.4      615
##
## Scaled residuals:
##      Min       1Q   Median       3Q      Max
## -2.28808 -0.43372 -0.03786  0.41513  2.54026
##
## Random effects:
##  Groups   Name        Variance Std.Dev. Corr
##  UID      (Intercept) 32.43    5.695
##           month       19.39    4.403    0.46
##  Residual             10.82    3.290
## Number of obs: 625, groups:  UID, 179
##
## Fixed effects:
##              Estimate Std. Error t value
## (Intercept)  12.91213    5.71935   2.258
## AGE           0.05498    0.06101   0.901
## EDUCATION     0.05426    0.14712   0.369
## group1        0.69923    2.65425   0.263
## month         4.87580    2.37431   2.054
## group1:month  0.16899    2.40962   0.070
##
## Correlation of Fixed Effects:
##             (Intr) AGE    EDUCAT group1 month
## AGE         -0.836
## EDUCATION   -0.340  0.032
## group1      -0.433  0.047 -0.142
```

© 2018 Kennedy RE et al. *JAMA Network Open*.

```
## month        0.061  0.007  0.039 -0.175
## group1:mnth -0.059 -0.007 -0.040  0.178 -0.985
## P Value for Full vs. Reduced Model
## [1] 0.9441329
```

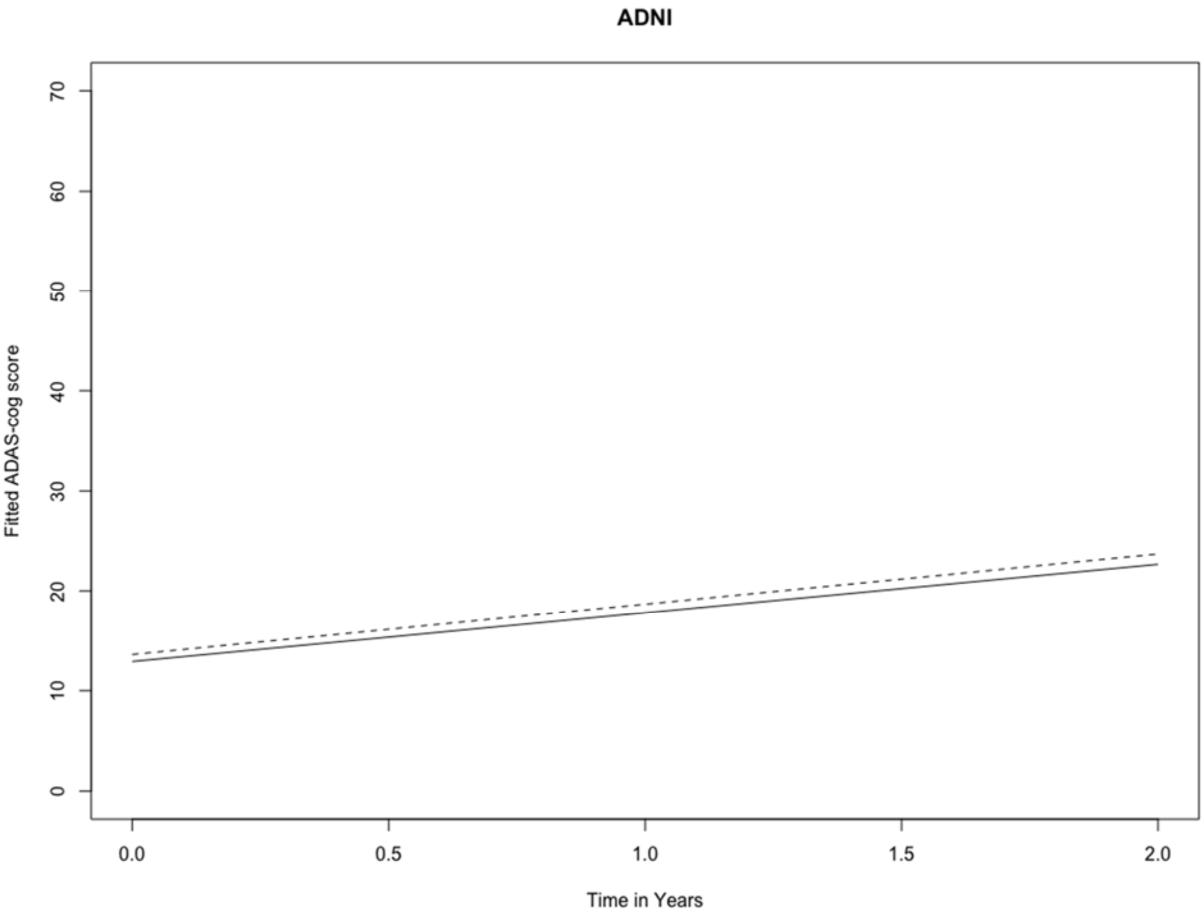


```
## Study Name
## [1] "DHA"
## Full Mixed Effects Model
## Linear mixed model fit by maximum likelihood  ['lmerMod']
## Formula: ADAS ~ AGE + EDUCATION + group + month + group:month + (month |
##     UID)
##    Data: mixed.data
##
##      AIC      BIC   logLik deviance df.resid
##   8300.2   8351.8  -4140.1   8280.2     1280
##
## Scaled residuals:
##      Min       1Q   Median       3Q      Max
## -2.54915 -0.48624 -0.01167  0.47009  2.70408
##
```

© 2018 Kennedy RE et al. *JAMA Network Open*.

```
## Random effects:
##  Groups   Name        Variance Std.Dev. Corr
##  UID      (Intercept) 61.39    7.835
##           month       22.69    4.763    0.63
##  Residual             11.51    3.393
## Number of obs: 1290, groups:  UID, 386
##
## Fixed effects:
##              Estimate Std. Error t value
## (Intercept)   9.16954    4.81603   1.904
## AGE           0.13973    0.04805   2.908
## EDUCATION    -0.19013    0.14829  -1.282
## group1        7.00299    1.52905   4.580
## month         3.20138    1.10153   2.906
## group1:month  2.23547    1.15064   1.943
##
## Correlation of Fixed Effects:
##             (Intr) AGE    EDUCAT group1 month
## AGE         -0.848
## EDUCATION   -0.557  0.149
## group1      -0.359  0.082  0.013
## month        0.095 -0.005  0.004 -0.294
## group1:mnth -0.085 -0.001 -0.009  0.306 -0.957
## P Value for Full vs. Reduced Model
## [1] 0.0529316
```

© 2018 Kennedy RE et al. *JAMA Network Open*.

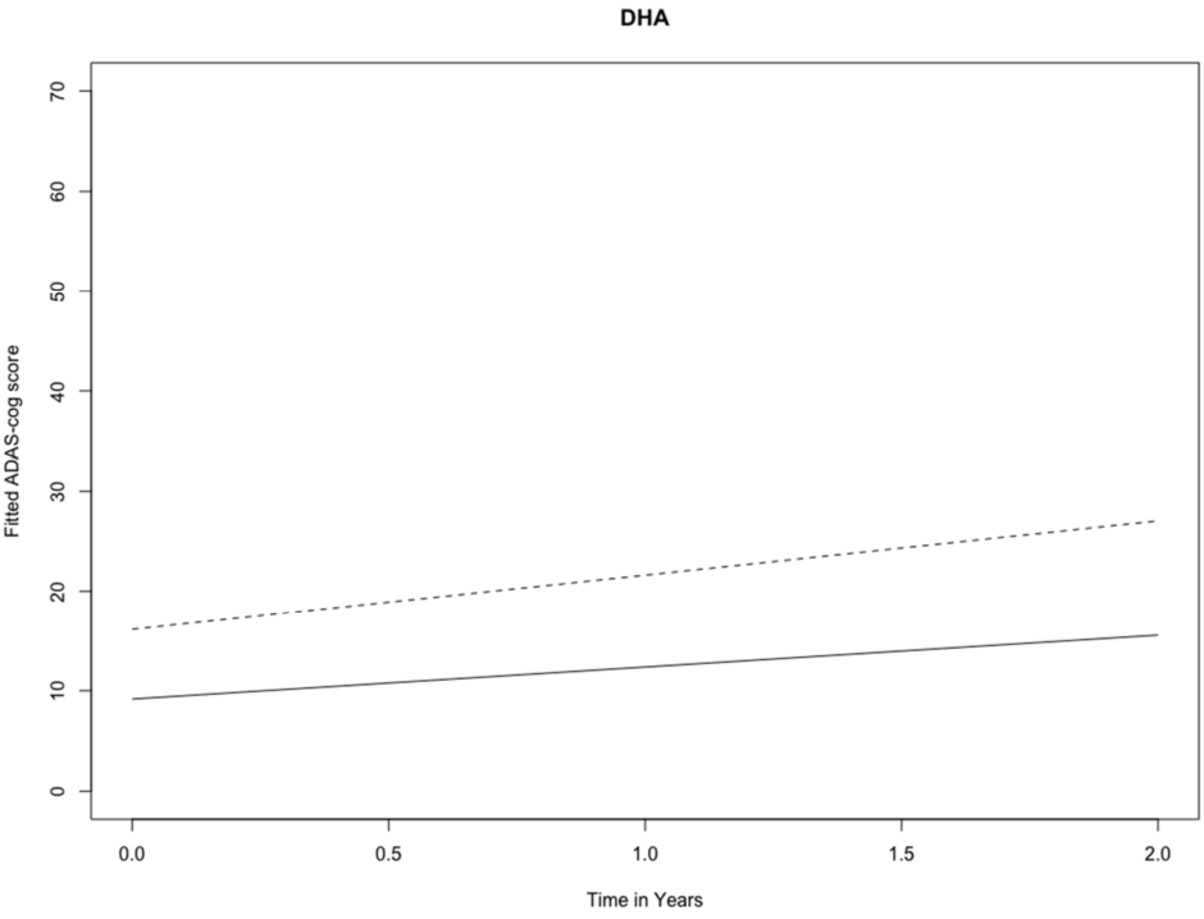


```
## Study Name
## [1] "ES"
## Full Mixed Effects Model
## Linear mixed model fit by maximum likelihood  ['lmerMod']
## Formula: ADAS ~ AGE + EDUCATION + group + month + group:month + (month |
##     UID)
##    Data: mixed.data
##
##      AIC      BIC   logLik deviance df.resid
##   2004.6   2041.7   -992.3   1984.6      292
##
## Scaled residuals:
##      Min       1Q   Median       3Q      Max
## -2.47336 -0.52156 -0.01569  0.48441  2.62754
##
## Random effects:
##  Groups   Name        Variance Std.Dev. Corr
##  UID      (Intercept) 62.40    7.899
##           month       15.68    3.959    0.87
##  Residual             13.66    3.696
```

© 2018 Kennedy RE et al. *JAMA Network Open*.

```
## Number of obs: 302, groups:  UID, 115
##
## Fixed effects:
##              Estimate Std. Error t value
## (Intercept)   14.9766     9.8512   1.520
## AGE            0.1429     0.1177   1.215
## EDUCATION     -0.1100     0.2496  -0.441
## group1        -0.1170     1.8339  -0.064
## month          4.8566     0.7858   6.181
## group1:month   3.4706     1.5006   2.313
##
## Correlation of Fixed Effects:
##             (Intr) AGE    EDUCAT group1 month
## AGE         -0.950
## EDUCATION   -0.442  0.155
## group1      -0.061  0.059 -0.135
## month        0.023 -0.002 -0.007 -0.106
## group1:mnth -0.018  0.004  0.012  0.208 -0.524
## P Value for Full vs. Reduced Model
## [1] 0.02347949
```

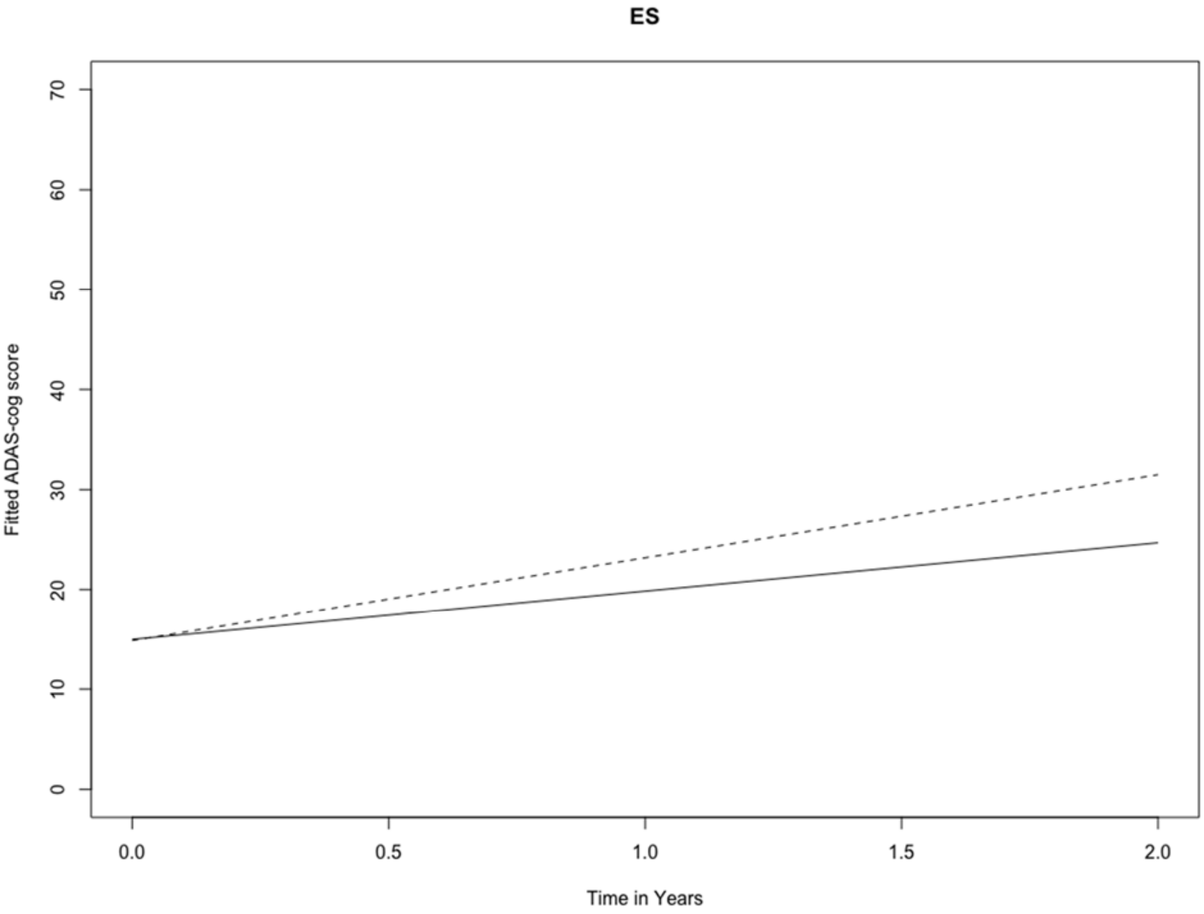

© 2018 Kennedy RE et al. *JAMA Network Open*.

```
## Study Name
## [1] "HC"
## Full Mixed Effects Model
## Linear mixed model fit by maximum likelihood  ['lmerMod']
## Formula: ADAS ~ AGE + EDUCATION + group + month + group:month + (month |
##     UID)
##    Data: mixed.data
##
##      AIC      BIC   logLik deviance df.resid
##   8739.9   8792.0  -4360.0   8719.9     1342
##
## Scaled residuals:
##     Min      1Q  Median      3Q     Max
## -3.2002 -0.4998 -0.0318  0.4747  4.1500
##
## Random effects:
##  Groups   Name        Variance Std.Dev. Corr
##  UID      (Intercept) 57.55    7.586
##           month       17.18    4.145    0.51
##  Residual             13.38    3.657
## Number of obs: 1352, groups:  UID, 383
##
## Fixed effects:
##              Estimate Std. Error t value
## (Intercept)   8.46425    4.83373   1.751
## AGE           0.16653    0.05377   3.097
## EDUCATION    -0.43377    0.13749  -3.155
## group1        7.46975    1.82102   4.102
## month         2.07515    1.34071   1.548
## group1:month  2.39751    1.37374   1.745
##
## Correlation of Fixed Effects:
##             (Intr) AGE    EDUCAT group1 month
## AGE         -0.855
## EDUCATION   -0.454  0.100
## group1      -0.243 -0.096 -0.077
## month        0.056 -0.011  0.004 -0.129
## group1:mnth -0.053  0.008 -0.004  0.134 -0.976
## P Value for Full vs. Reduced Model
## [1] 0.08138587
```

© 2018 Kennedy RE et al. *JAMA Network Open*.

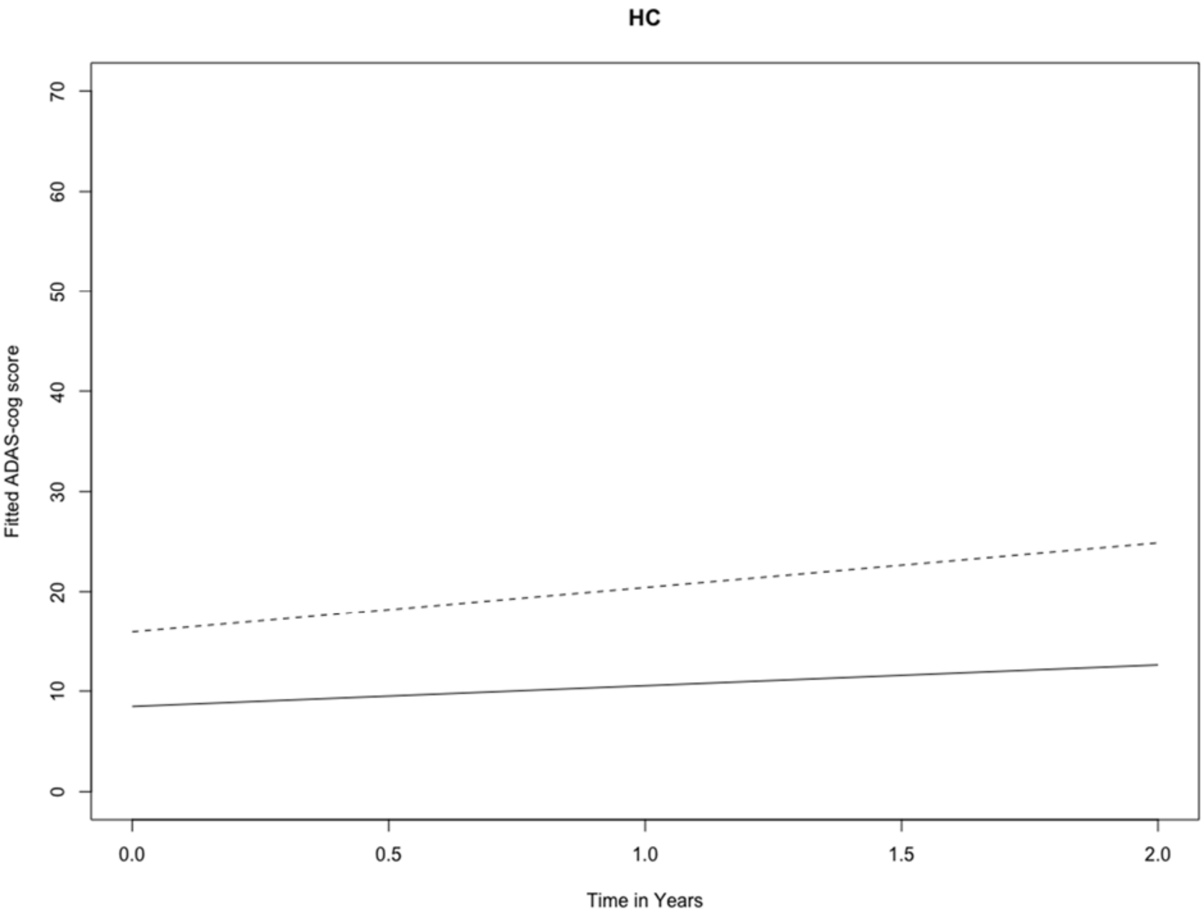


```
## Study Name
## [1] "HU"
## Full Mixed Effects Model
## Linear mixed model fit by maximum likelihood  ['lmerMod']
## Formula: ADAS ~ AGE + EDUCATION + group + month + group:month + (month |
##     UID)
##    Data: mixed.data
##
##      AIC      BIC   logLik deviance df.resid
##   3184.0   3225.5  -1582.0   3164.0      460
##
## Scaled residuals:
##     Min      1Q  Median      3Q     Max
## -2.1772 -0.4950 -0.0242  0.4914  2.7602
##
## Random effects:
##  Groups   Name        Variance Std.Dev. Corr
##  UID      (Intercept) 96.145   9.805
##           month        2.761   1.662    1.00
##  Residual             15.926   3.991
```

© 2018 Kennedy RE et al. *JAMA Network Open*.

```
## Number of obs: 470, groups:  UID, 189
##
## Fixed effects:
##             Estimate Std. Error t value
## (Intercept)  30.50647    8.52768   3.577
## AGE         -0.04086    0.09842  -0.415
## EDUCATION    -0.35447    0.23224  -1.526
## group1        6.69647    1.53576   4.360
## month         1.52377    0.69837   2.182
## group1:month  0.30563    0.94194   0.324
##
## Correlation of Fixed Effects:
##            (Intr) AGE    EDUCAT group1 month
## AGE        -0.926
## EDUCATION   -0.393  0.042
## group1      -0.120  0.072 -0.103
## month        0.014 -0.011 -0.004 -0.016
## group1:mnth -0.012  0.005  0.013  0.031 -0.741
## P Value for Full vs. Reduced Model
## [1] 0.7457864
```

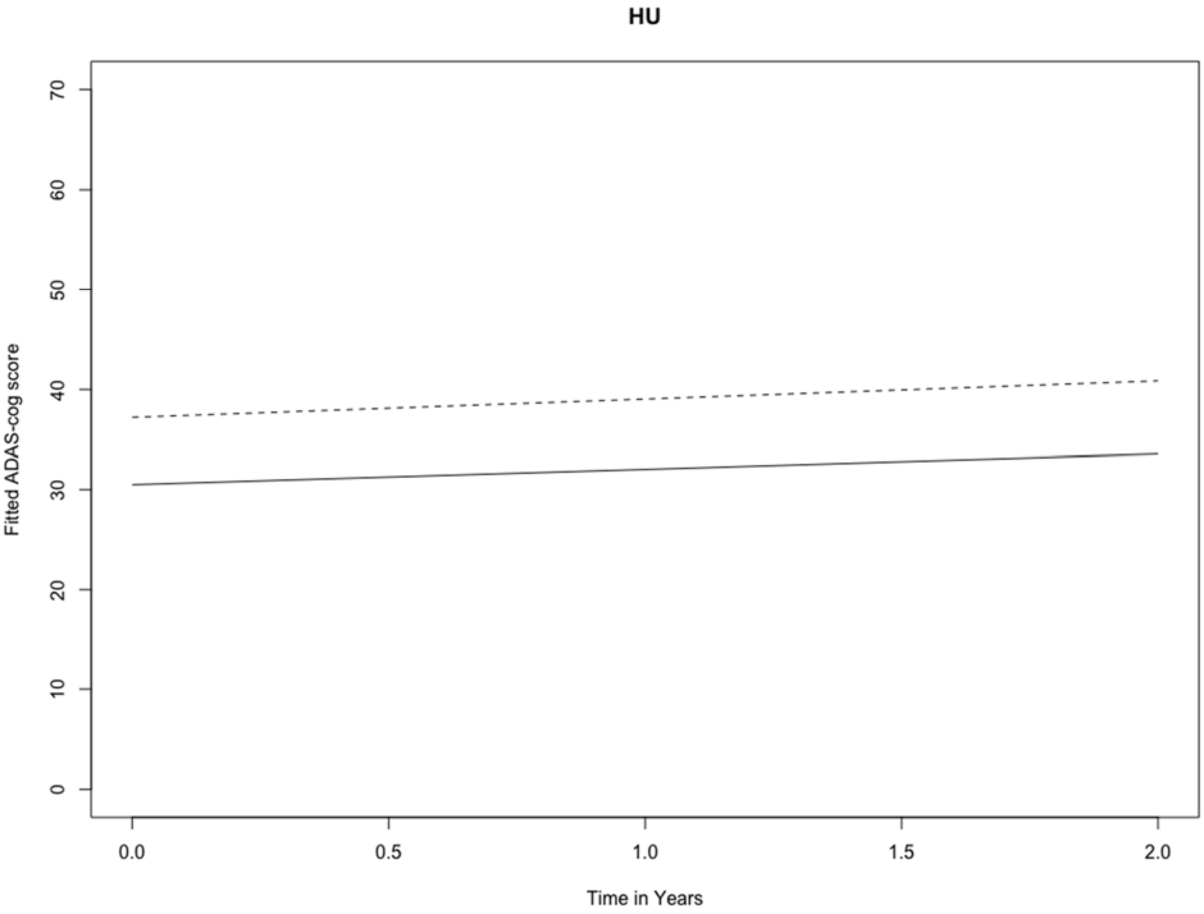

© 2018 Kennedy RE et al. *JAMA Network Open*.

```
## Study Name
## [1] "LL"
## Full Mixed Effects Model
## Linear mixed model fit by maximum likelihood  ['lmerMod']
## Formula: ADAS ~ AGE + EDUCATION + group + month + group:month + (month |
##     UID)
##    Data: mixed.data
##
##      AIC      BIC   logLik deviance df.resid
##   8335.5   8386.9  -4157.7   8315.5     1247
##
## Scaled residuals:
##      Min       1Q   Median       3Q      Max
## -2.44423 -0.48247 -0.00702  0.46690  2.90282
##
## Random effects:
##  Groups   Name        Variance Std.Dev. Corr
##  UID      (Intercept) 86.68    9.310
##           month       19.76    4.445    0.40
##  Residual             13.64    3.693
## Number of obs: 1257, groups:  UID, 386
##
## Fixed effects:
##               Estimate Std. Error t value
## (Intercept)  19.639525   5.512140   3.563
## AGE          -0.006826   0.054198  -0.126
## EDUCATION     0.078098   0.158249   0.494
## group1        3.692815   2.788784   1.324
## month         5.048043   1.914305   2.637
## group1:month  0.376432   1.942334   0.194
##
## Correlation of Fixed Effects:
##             (Intr) AGE    EDUCAT group1 month
## AGE         -0.759
## EDUCATION   -0.490  0.095
## group1      -0.485 -0.017  0.021
## month        0.056 -0.003  0.002 -0.108
## group1:mnth -0.054  0.002 -0.003  0.110 -0.986
## P Value for Full vs. Reduced Model
## [1] 0.8464143
```

© 2018 Kennedy RE et al. *JAMA Network Open*.

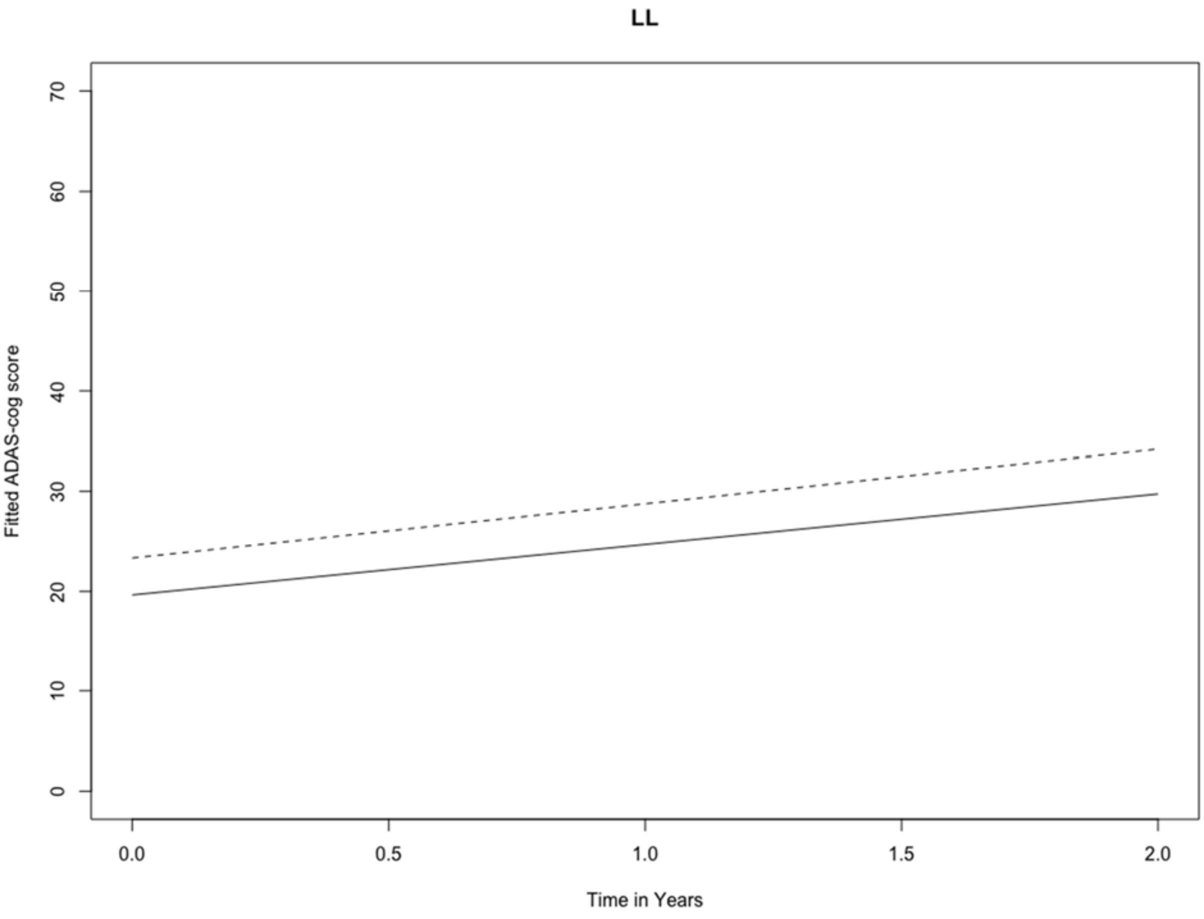


```
## Study Name
## [1] "NS"
## Full Mixed Effects Model
## Linear mixed model fit by maximum likelihood  ['lmerMod']
## Formula: ADAS ~ AGE + EDUCATION + group + month + group:month + (month |
##     UID)
##    Data: mixed.data
##
##      AIC      BIC   logLik deviance df.resid
##   5716.7   5764.1  -2848.4   5696.7      834
##
## Scaled residuals:
##     Min      1Q  Median      3Q     Max
## -2.5777 -0.4560 -0.0277  0.4424  2.6088
##
## Random effects:
##  Groups   Name        Variance Std.Dev. Corr
##  UID      (Intercept) 80.04    8.946
##           month       22.24    4.715    0.47
##  Residual             14.06    3.749
```

© 2018 Kennedy RE et al. *JAMA Network Open*.

```
## Number of obs: 844, groups:  UID, 344
##
## Fixed effects:
##              Estimate Std. Error t value
## (Intercept)  32.72073    6.02192   5.434
## AGE          -0.12793    0.06842  -1.870
## EDUCATION    -0.07395    0.16062  -0.460
## group1        1.91781    1.42228   1.348
## month         2.20356    1.17262   1.879
## group1:month  3.61425    1.27001   2.846
##
## Correlation of Fixed Effects:
##             (Intr) AGE    EDUCAT group1 month
## AGE         -0.911
## EDUCATION   -0.462  0.124
## group1      -0.282  0.131 -0.069
## month        0.009  0.004  0.003 -0.056
## group1:mnth -0.007 -0.005 -0.005  0.062 -0.923
## P Value for Full vs. Reduced Model
## [1] 0.004782265
```

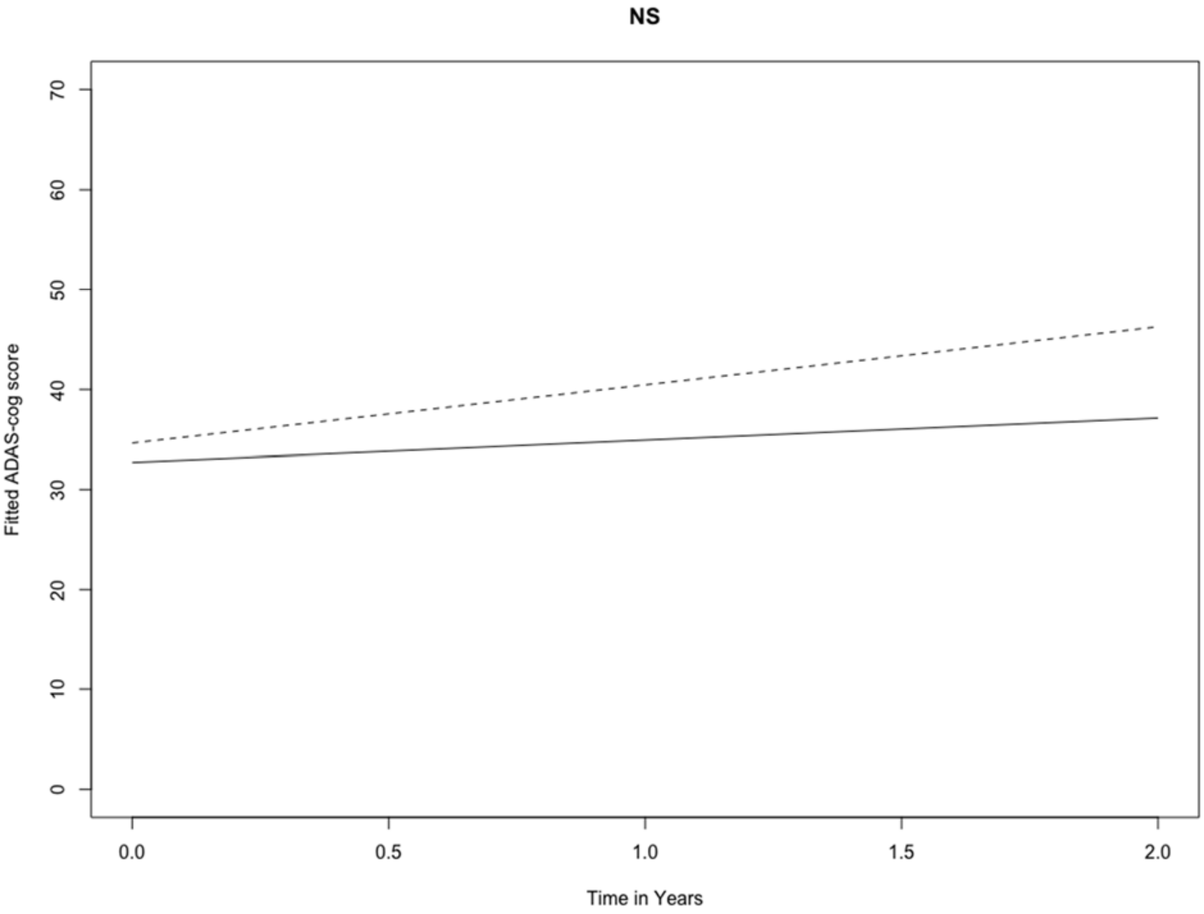

© 2018 Kennedy RE et al. *JAMA Network Open*.

```
## Study Name
## [1] "PR"
## Full Mixed Effects Model
## Linear mixed model fit by maximum likelihood  ['lmerMod']
## Formula: ADAS ~ AGE + EDUCATION + group + month + group:month + (month |
##     UID)
##    Data: mixed.data
##
##      AIC      BIC   logLik deviance df.resid
##   2958.4   2999.2  -1469.2   2938.4      428
##
## Scaled residuals:
##      Min       1Q   Median       3Q      Max
## -2.76980 -0.50680 -0.02234  0.47104  2.93902
##
## Random effects:
##  Groups   Name        Variance Std.Dev. Corr
##  UID      (Intercept) 81.11    9.006
##           month       22.10    4.701    0.76
##  Residual             16.67    4.083
## Number of obs: 438, groups:  UID, 135
##
## Fixed effects:
##             Estimate Std. Error t value
## (Intercept)  32.78688    9.01135   3.638
## AGE          -0.06482    0.10664  -0.608
## EDUCATION    -0.43416    0.25763  -1.685
## group1       -0.12742    2.39055  -0.053
## month         5.99618    0.63927   9.380
## group1:month -2.16187    1.54065  -1.403
##
## Correlation of Fixed Effects:
##             (Intr) AGE    EDUCAT group1 month
## AGE         -0.911
## EDUCATION   -0.521  0.137
## group1      -0.051 -0.008  0.049
## month        0.053 -0.014 -0.026 -0.117
## group1:mnth -0.032  0.014  0.017  0.356 -0.415
## P Value for Full vs. Reduced Model
## [1] 0.1620023
```

© 2018 Kennedy RE et al. *JAMA Network Open*.

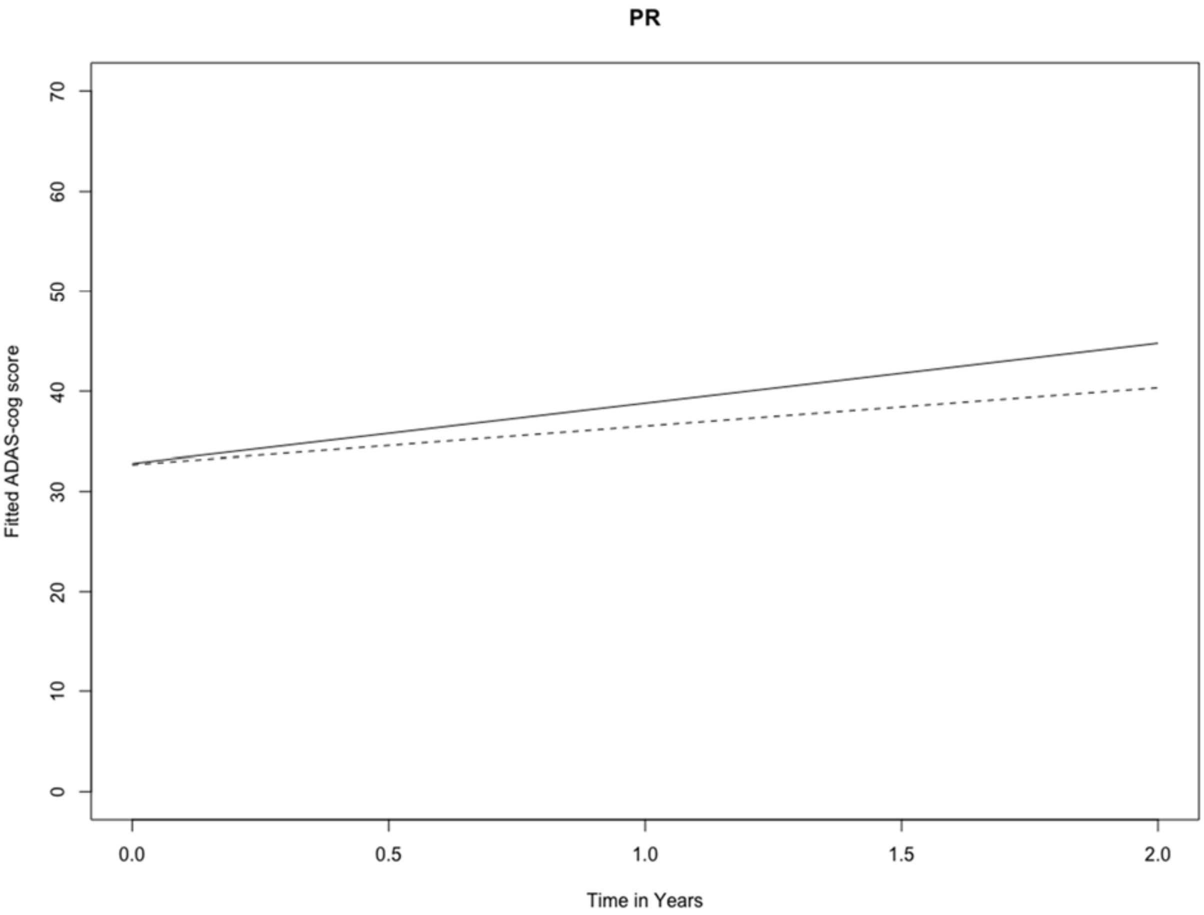


```
## Study Name
## [1] "SL"
## Full Mixed Effects Model
## Linear mixed model fit by maximum likelihood  ['lmerMod']
## Formula: ADAS ~ AGE + EDUCATION + group + month + group:month + (month |
##     UID)
##    Data: mixed.data
##
##      AIC      BIC   logLik deviance df.resid
##   6467.8   6516.5  -3223.9   6447.8      951
##
## Scaled residuals:
##     Min      1Q  Median      3Q     Max
## -2.9713 -0.4627  0.0355  0.4802  3.1977
##
## Random effects:
##  Groups   Name        Variance Std.Dev. Corr
##  UID      (Intercept) 105.53   10.273
##           month        14.80    3.847   0.34
##  Residual              14.68    3.831
```

© 2018 Kennedy RE et al. *JAMA Network Open*.

```
## Number of obs: 961, groups:  UID, 316
##
## Fixed effects:
##               Estimate Std. Error t value
## (Intercept)   51.372173   6.398754   8.028
## AGE           -0.172115   0.076926  -2.237
## EDUCATION      0.006988   0.187404   0.037
## group1       -12.686658   7.818356  -1.623
## month          7.769544   0.356626  21.786
## group1:month   3.711581   3.751239   0.989
##
## Correlation of Fixed Effects:
##             (Intr) AGE    EDUCAT group1 month
## AGE         -0.929
## EDUCATION   -0.488  0.145
## group1      -0.015  0.087 -0.191
## month        0.002  0.001  0.010 -0.007
## group1:mnth  0.001  0.000 -0.003  0.086 -0.095
## P Value for Full vs. Reduced Model
## [1] 0.3234868
```

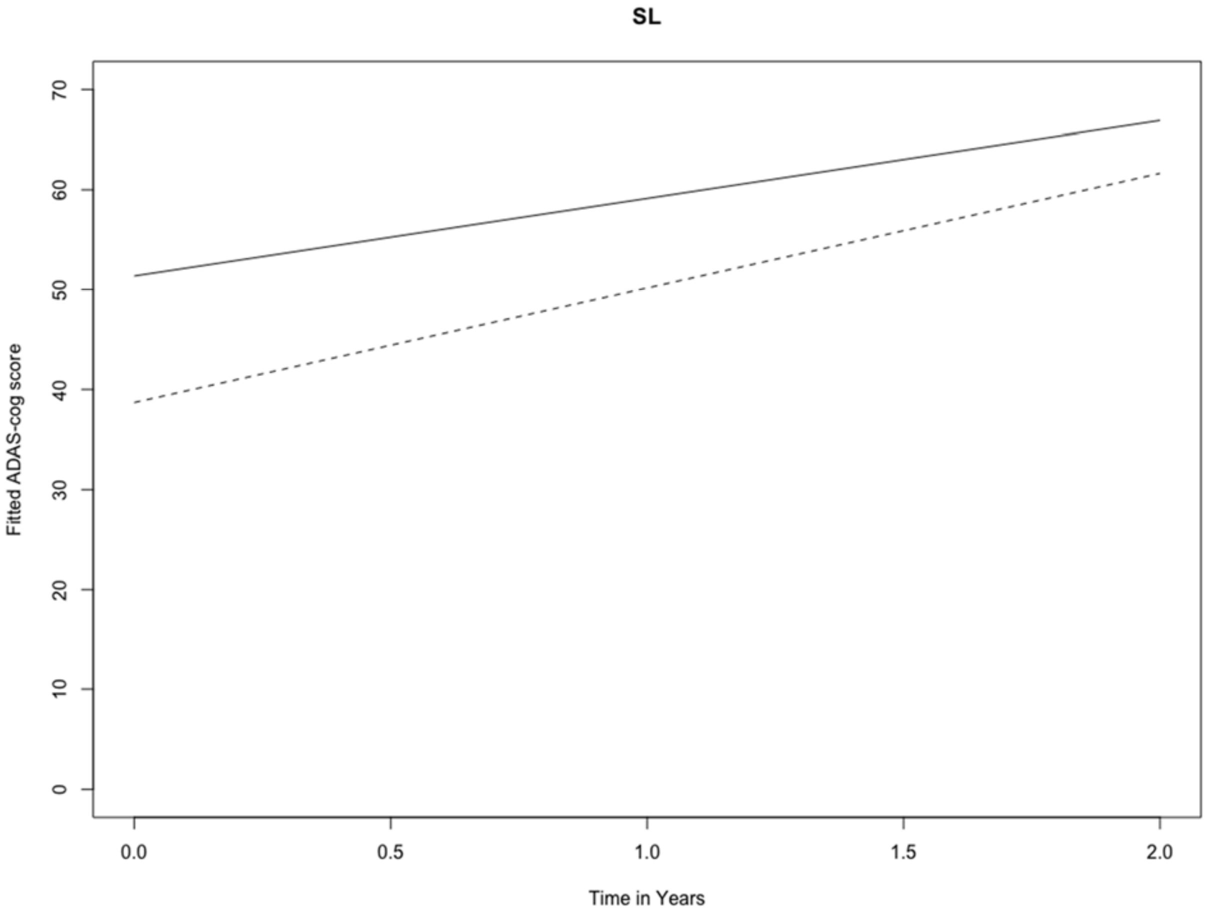

© 2018 Kennedy RE et al. *JAMA Network Open*.

```
## Study Name
## [1] "VN"
## Full Mixed Effects Model
## Linear mixed model fit by maximum likelihood  ['lmerMod']
## Formula: ADAS ~ AGE + EDUCATION + group + month + group:month + (month |
##     UID)
##    Data: mixed.data
##
##      AIC      BIC   logLik deviance df.resid
##   5767.6   5815.3  -2873.8   5747.6      860
##
## Scaled residuals:
##     Min      1Q  Median      3Q     Max
## -3.0375 -0.4897 -0.0031  0.4356  4.0284
##
## Random effects:
##  Groups   Name        Variance Std.Dev. Corr
##  UID      (Intercept) 65.40    8.087
##           month       13.96    3.736    0.31
##  Residual             15.89    3.986
## Number of obs: 870, groups:  UID, 281
##
## Fixed effects:
##              Estimate Std. Error t value
## (Intercept)  41.58966    7.06175   5.889
## AGE          -0.12357    0.06702  -1.844
## EDUCATION    -0.07391    0.15826  -0.467
## group1       -2.03790    3.63689  -0.560
## month         6.58004    2.45421   2.681
## group1:month -0.44030    2.48236  -0.177
##
## Correlation of Fixed Effects:
##             (Intr) AGE    EDUCAT group1 month
## AGE         -0.793
## EDUCATION   -0.434  0.125
## group1      -0.577  0.072  0.069
## month        0.004 -0.006 -0.003  0.001
## group1:mnth -0.005  0.006  0.003 -0.001 -0.989
## P Value for Full vs. Reduced Model
## [1] 0.8592453
```

© 2018 Kennedy RE et al. *JAMA Network Open*.

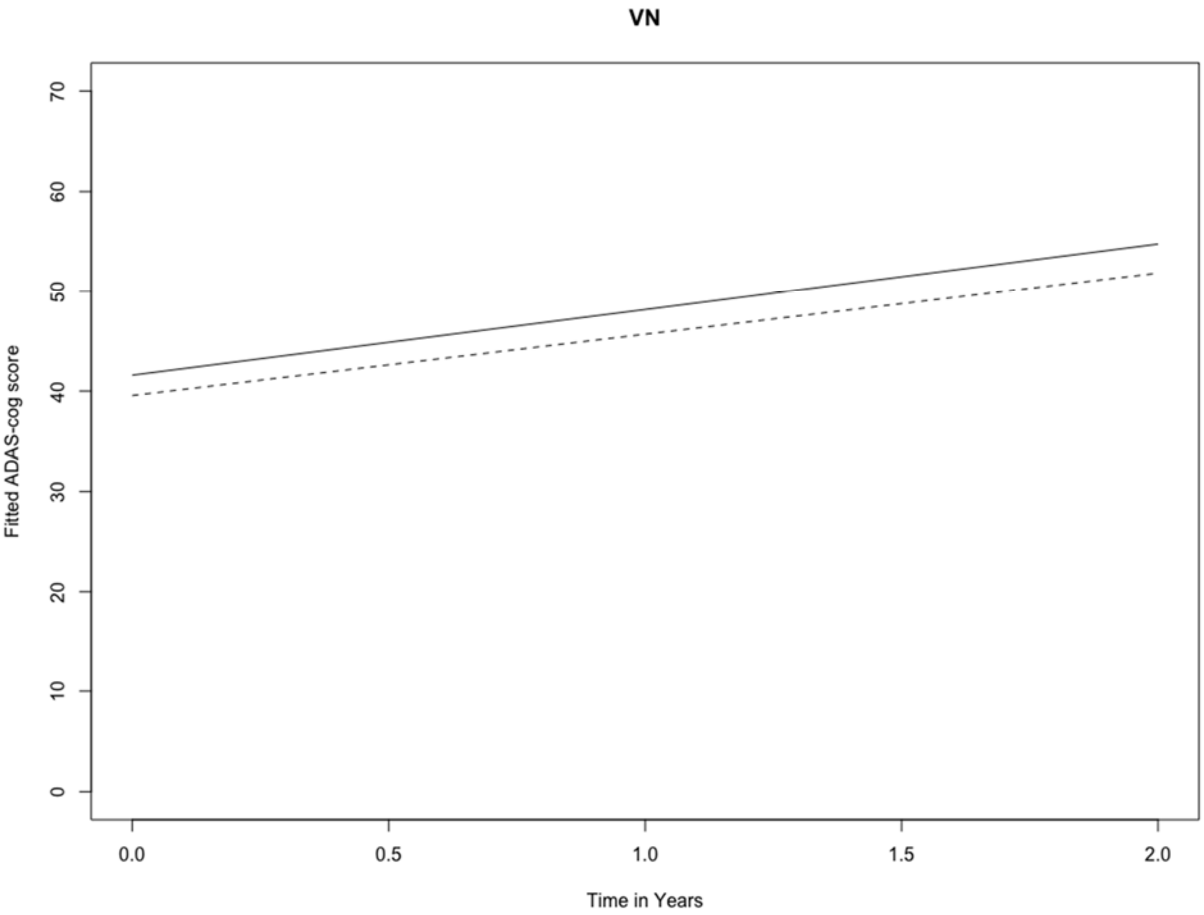

Meta-Analysis with metafor

```
##
## Random-Effects Model (k = 10; tau^2 estimator: REML)
##
## tau^2 (estimated amount of total heterogeneity): 1.5630 (SE = 1.8553)
## tau (square root of estimated tau^2 value):      1.2502
## I^2 (total heterogeneity / total variability):   40.44%
## H^2 (total variability / sampling variability):  1.68
##
## Test for Heterogeneity:
## Q(df = 9) = 14.1588, p-val = 0.1168
##
## Model Results:
##
## estimate      se    zval    pval   ci.lb   ci.ub
##   1.4074  0.6466  2.1767  0.0295  0.1401  2.6747  *
##
## ---
## Signif. codes:  0 '***' 0.001 '**' 0.01 '*' 0.05 '.' 0.1 ' ' 1
```

© 2018 Kennedy RE et al. *JAMA Network Open*.

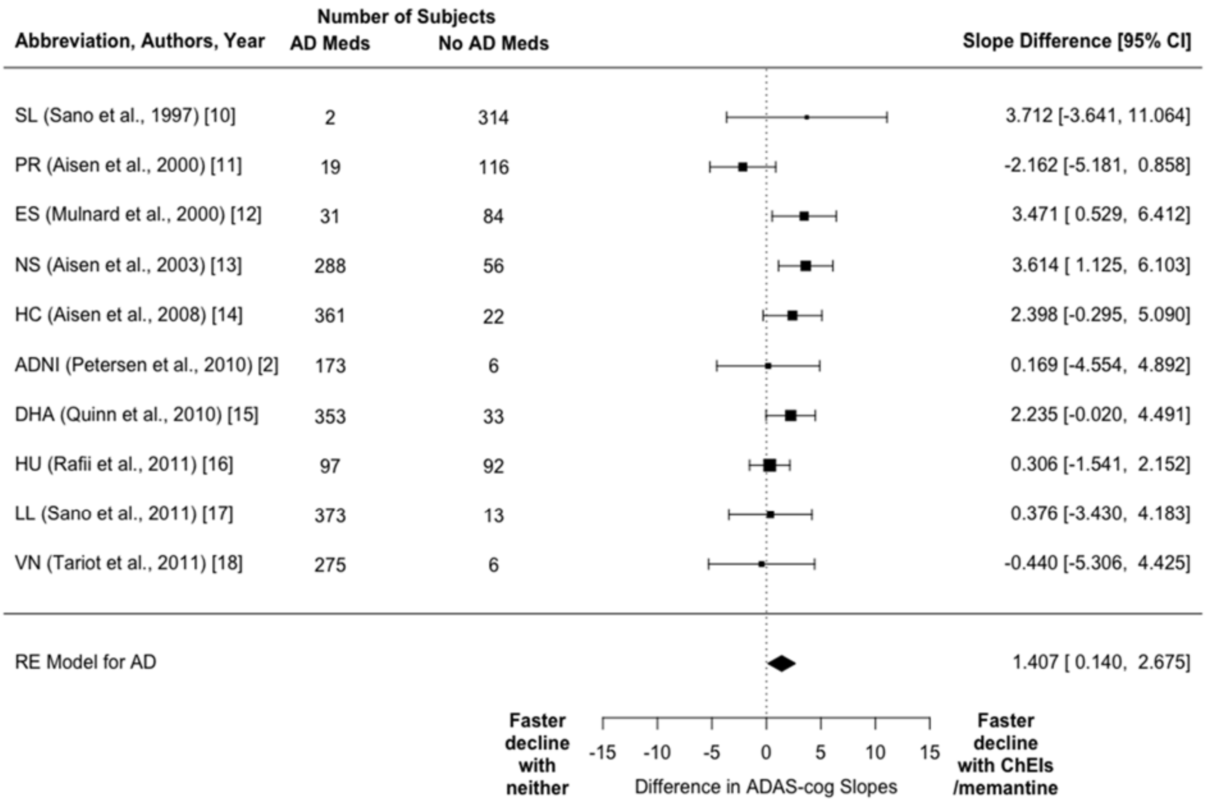

© 2018 Kennedy RE et al. *JAMA Network Open*.

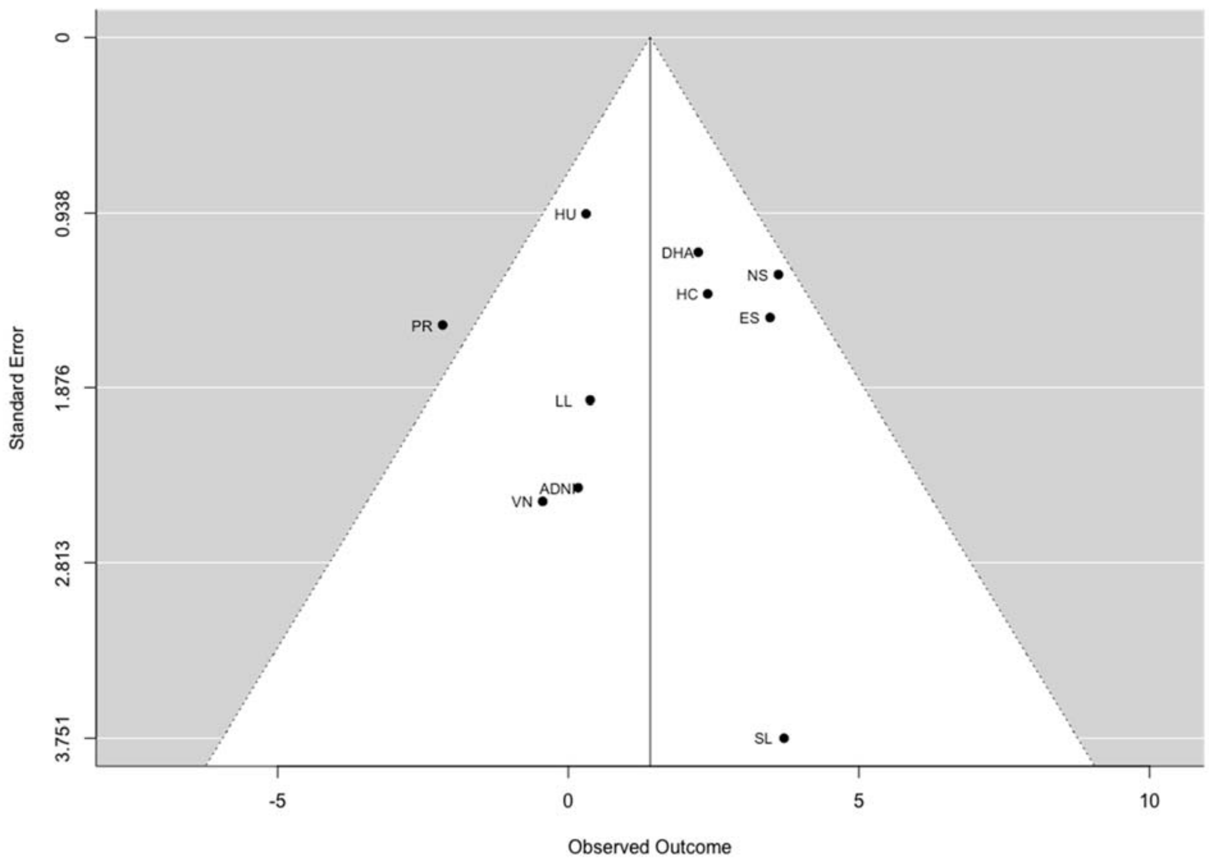

Fail Safe N

```
##
## Fail-safe N Calculation Using the Rosenberg Approach
##
## Average Effect Size:         1.4219
## Observed Significance Level: 0.0024
## Target Significance Level:   0.05
##
## Fail-safe N: 15
```

© 2018 Kennedy RE et al. *JAMA Network Open*.

Participant Characteristics

Baseline Characteristics for All Studies

	Both N=923	ChEI N=906	Memantine N=143	Neither N=742	P Value	N
AGE	74.5 (8.78)	75.5 (7.75)	78.1 (7.24)	74.5 (8.16)	<0.001	2714
EDCAT:					<0.001	2714
Less than HS	72 (7.80%)	126 (13.9%)	20 (14.0%)	169 (22.8%)		
HS graduate	437 (47.3%)	447 (49.3%)	69 (48.3%)	383 (51.6%)		
College graduate	414 (44.9%)	333 (36.8%)	54 (37.8%)	190 (25.6%)		
RACE: White	851 (92.2%)	821 (90.6%)	134 (93.7%)	651 (87.7%)	0.010	2714
ETHNIC: Hispanic	32 (3.47%)	45 (4.97%)	9 (6.29%)	38 (5.12%)	0.222	2714
SEX: Female	478 (51.8%)	529 (58.4%)	94 (65.7%)	477 (64.3%)	<0.001	2714
MARITAL: Married	714 (77.4%)	637 (70.3%)	101 (70.6%)	505 (68.1%)	<0.001	2714
TX: Placebo	490 (53.1%)	385 (42.5%)	68 (47.6%)	246 (33.2%)	<0.001	2714
ADAS_bl	25.7 (9.54)	22.5 (8.78)	28.4 (11.0)	29.3 (13.2)	<0.001	2714
ADAS_m06	27.5 (10.2)	23.7 (9.60)	27.2 (10.4)	29.9 (14.3)	<0.001	2240
ADAS_m12	29.8 (11.3)	25.0 (10.3)	28.2 (10.8)	31.5 (15.4)	<0.001	1944
ADAS_m18	31.9 (12.1)	25.6 (10.7)	28.1 (10.4)	34.8 (16.4)	<0.001	1182
ADAS_m24	32.5 (12.2)	28.3 (11.2)	33.8 (10.0)	44.7 (13.1)	<0.001	329

Baseline Characteristics for DHA

	Both N=228	ChEI N=101	Memantine N=24	Neither N=33	P Value	N
AGE	75.1 (8.84)	76.6 (8.24)	78.5 (5.64)	78.4 (8.71)	0.052	386
EDCAT:					0.162	386

© 2018 Kennedy RE et al. *JAMA Network Open*.

Less than HS	14 (6.14%)	12 (11.9%)	5 (20.8%)	3 (9.09%)		
HS graduate	112 (49.1%)	53 (52.5%)	9 (37.5%)	16 (48.5%)		
College graduate	102 (44.7%)	36 (35.6%)	10 (41.7%)	14 (42.4%)		
RACE: White	211 (92.5%)	92 (91.1%)	22 (91.7%)	28 (84.8%)	0.471	386
ETHNIC: Hispanic	5 (2.19%)	3 (2.97%)	2 (8.33%)	4 (12.1%)	0.022	386
SEX: Female	122 (53.5%)	56 (55.4%)	13 (54.2%)	12 (36.4%)	0.271	386
MARITAL: Married	169 (74.1%)	68 (67.3%)	17 (70.8%)	22 (66.7%)	0.569	386
TX: Placebo	102 (44.7%)	31 (30.7%)	12 (50.0%)	14 (42.4%)	0.087	386
ADAS_bl	25.3 (8.76)	21.2 (6.97)	25.0 (9.81)	17.8 (8.30)	<0.001	386
ADAS_m06	27.9 (9.89)	22.3 (8.24)	29.1 (9.38)	18.1 (11.4)	<0.001	340
ADAS_m12	29.6 (11.2)	23.5 (9.31)	28.1 (10.1)	17.0 (12.3)	<0.001	300
ADAS_m18	31.0 (12.0)	26.8 (11.8)	27.4 (11.6)	20.4 (14.0)	<0.001	264

Baseline Characteristics for ES

	ChEI N=31	Neither N=84	P Value	N
AGE	74.3 (7.84)	75.6 (6.43)	0.419	115
EDCAT:			0.127	115
Less than HS	4 (12.9%)	26 (31.0%)		
HS graduate	21 (67.7%)	42 (50.0%)		
College graduate	6 (19.4%)	16 (19.0%)		
RACE: White	30 (96.8%)	72 (85.7%)	0.181	115
ETHNIC: Hispanic	0 (0.00%)	4 (4.76%)	0.573	115
SEX: Female	31 (100%)	84 (100%)	.	115
MARITAL: Married	16 (51.6%)	36 (42.9%)	0.531	115
TX: Placebo	7 (22.6%)	30 (35.7%)	0.266	115
ADAS_bl	24.1 (9.08)	24.3 (9.22)	0.923	115
ADAS_m06	27.8 (10.1)	27.1 (9.40)	0.756	97

© 2018 Kennedy RE et al. *JAMA Network Open*.

ADAS_m12	32.4 (14.2)	27.4 (10.5)	0.118	90

Baseline Characteristics for HC

	Both N=186	ChEI N=171	Memantine N=4	Neither N=22	P Value	N
AGE	75.7 (8.01)	77.4 (7.29)	82.0 (8.91)	73.6 (8.42)	0.024	383
EDCAT:					0.238	383
Less than HS	17 (9.14%)	26 (15.2%)	0 (0.00%)	6 (27.3%)		
HS graduate	95 (51.1%)	81 (47.4%)	2 (50.0%)	10 (45.5%)		
College graduate	74 (39.8%)	64 (37.4%)	2 (50.0%)	6 (27.3%)		
RACE: White	171 (91.9%)	147 (86.0%)	4 (100%)	9 (40.9%)	<0.001	383
ETHNIC: Hispanic	7 (3.76%)	14 (8.19%)	0 (0.00%)	0 (0.00%)	0.244	383
SEX: Female	98 (52.7%)	97 (56.7%)	3 (75.0%)	16 (72.7%)	0.277	383
MARITAL: Married	141 (75.8%)	106 (62.0%)	3 (75.0%)	11 (50.0%)	0.006	383
TX: Placebo	78 (41.9%)	68 (39.8%)	2 (50.0%)	7 (31.8%)	0.781	383
ADAS_bl	24.6 (8.93)	21.0 (8.05)	20.5 (3.16)	15.0 (7.73)	<0.001	383
ADAS_m06	26.4 (9.99)	21.5 (7.61)	22.3 (2.09)	14.6 (8.21)	<0.001	346
ADAS_m12	29.2 (10.3)	22.3 (8.25)	24.8 (5.53)	17.1 (8.28)	<0.001	324
ADAS_m18	31.4 (12.3)	23.6 (10.1)	26.9 (8.47)	16.3 (8.41)	<0.001	299

Baseline Characteristics for Study HU

	Both N=7	ChEI N=2	Memantine N=88	Neither N=92	P Value	N
AGE	77.9 (6.36)	78.0 (2.83)	77.8 (7.54)	79.0 (8.21)	0.762	189
EDCAT:					0.310	189
Less than HS	2 (28.6%)	0 (0.00%)	11 (12.5%)	15 (16.3%)		

© 2018 Kennedy RE et al. *JAMA Network Open*.

HS graduate	3 (42.9%)	1 (50.0%)	46 (52.3%)	57 (62.0%)		
College graduate	2 (28.6%)	1 (50.0%)	31 (35.2%)	20 (21.7%)		
RACE: White	7 (100%)	1 (50.0%)	82 (93.2%)	86 (93.5%)	0.261	189
ETHNIC: Hispanic	0 (0.00%)	0 (0.00%)	5 (5.68%)	6 (6.52%)	1.000	189
SEX: Female	3 (42.9%)	1 (50.0%)	57 (64.8%)	55 (59.8%)	0.598	189
MARITAL: Married	7 (100%)	1 (50.0%)	63 (71.6%)	62 (67.4%)	0.238	189
TX: Placebo	3 (42.9%)	0 (0.00%)	35 (39.8%)	28 (30.4%)	0.406	189
ADAS_bl	30.2 (7.96)	23.7 (0.00)	29.9 (11.7)	23.2 (10.0)	0.001	189
ADAS_m06	26.2 (7.45)	24.3 (0.94)	26.2 (11.0)	20.6 (9.34)	0.015	141
ADAS_m12	26.5 (4.13)	22.0 (.)	27.8 (11.4)	21.2 (11.2)	0.028	111
ADAS_m18	27.2 (2.04)	30.7 (.)	25.9 (8.57)	21.5 (10.8)	0.554	29

Baseline Characteristics for LL

	Both N=195	ChEI N=167	Memantine N=11	Neither N=13	P Value	N
AGE	72.6 (9.85)	75.5 (8.31)	79.8 (8.13)	73.2 (9.33)	0.004	386
EDCAT:					0.084	386
Less than HS	12 (6.15%)	24 (14.4%)	1 (9.09%)	1 (7.69%)		
HS graduate	89 (45.6%)	83 (49.7%)	4 (36.4%)	6 (46.2%)		
College graduate	94 (48.2%)	60 (35.9%)	6 (54.5%)	6 (46.2%)		
RACE: White	179 (91.8%)	153 (91.6%)	10 (90.9%)	10 (76.9%)	0.275	386
ETHNIC: Hispanic	12 (6.15%)	12 (7.19%)	0 (0.00%)	0 (0.00%)	0.966	386
SEX: Female	104 (53.3%)	108 (64.7%)	10 (90.9%)	6 (46.2%)	0.014	386

© 2018 Kennedy RE et al. *JAMA Network Open*.

MARITAL: Married	151 (77.4%)	112 (67.1%)	6 (54.5%)	7 (53.8%)	0.027	386
TX: Placebo	94 (48.2%)	86 (51.5%)	8 (72.7%)	5 (38.5%)	0.339	386
ADAS_bl	26.1 (10.5)	21.6 (8.93)	28.2 (9.81)	20.8 (6.72)	<0.001	386
ADAS_m06	28.5 (10.9)	21.7 (9.55)	29.4 (11.8)	22.1 (6.73)	<0.001	326
ADAS_m12	31.0 (12.6)	22.3 (8.54)	34.5 (11.1)	26.3 (10.4)	<0.001	293
ADAS_m18	31.7 (12.8)	24.9 (9.37)	35.0 (19.6)	27.7 (9.40)	<0.001	252

Baseline Characteristics for NS

	Both N=1	ChEI N=287	Neither N=56	P Value	N
AGE	55.0 (.)	73.5 (7.60)	76.3 (7.50)	0.002	344
EDCAT:				0.198	344
Less than HS	0 (0.00%)	34 (11.8%)	12 (21.4%)		
HS graduate	0 (0.00%)	141 (49.1%)	25 (44.6%)		
College graduate	1 (100%)	112 (39.0%)	19 (33.9%)		
RACE: White	1 (100%)	262 (91.3%)	48 (85.7%)	0.290	344
ETHNIC: Hispanic	0 (0.00%)	13 (4.53%)	6 (10.7%)	0.150	344
SEX: Female	1 (100%)	148 (51.6%)	34 (60.7%)	0.243	344
MARITAL: Married	0 (0.00%)	229 (79.8%)	35 (62.5%)	0.003	344
TX: Placebo	1 (100%)	92 (32.1%)	17 (30.4%)	0.425	344
ADAS_bl	45.3 (.)	24.2 (9.55)	22.3 (9.64)	0.034	344
ADAS_m06	. (.)	26.3 (10.8)	20.8 (11.7)	0.005	269
ADAS_m12	. (.)	27.6 (10.6)	21.5 (10.9)	0.002	231

Baseline Characteristics for PR

	ChEI N=19	Neither N=116	P Value	N
AGE	72.6 (5.13)	72.3 (7.94)	0.838	135
EDCAT:			0.510	135
Less than HS	1 (5.26%)	14 (12.1%)		
HS graduate	13 (68.4%)	61 (52.6%)		
College graduate	5 (26.3%)	41 (35.3%)		
RACE: White	19 (100%)	110 (94.8%)	0.594	135
ETHNIC: Hispanic	0 (0.00%)	5 (4.31%)	1.000	135

© 2018 Kennedy RE et al. *JAMA Network Open*.

SEX: Female	12 (63.2%)	56 (48.3%)	0.339	135
MARITAL: Married	16 (84.2%)	97 (83.6%)	1.000	135
TX: Placebo	9 (47.4%)	58 (50.0%)	1.000	135
ADAS_bl	21.3 (8.05)	21.9 (10.2)	0.756	135
ADAS_m06	24.1 (11.2)	24.1 (11.6)	0.994	117
ADAS_m12	27.1 (12.9)	27.6 (12.9)	0.872	99
ADAS_m18	25.6 (12.6)	27.7 (13.7)	0.540	87

Baseline Characteristics for SL

	ChEI N=2	Neither N=314	P Value	N
AGE	61.0 (0.00)	73.0 (8.01)	<0.001	316
EDCAT:			0.042	316
Less than HS	0 (0.00%)	90 (28.7%)		
HS graduate	0 (0.00%)	161 (51.3%)		
College graduate	2 (100%)	63 (20.1%)		
RACE: White	1 (50.0%)	277 (88.2%)	0.226	316
ETHNIC: Hispanic	1 (50.0%)	12 (3.82%)	0.081	316
SEX: Female	1 (50.0%)	205 (65.3%)	1.000	316
MARITAL: Married	2 (100%)	228 (72.6%)	1.000	316
TX: Placebo	1 (50.0%)	77 (24.5%)	0.433	316
ADAS_bl	28.8 (3.06)	39.1 (10.8)	0.111	316
ADAS_m06	29.5 (1.18)	40.4 (12.0)	0.001	238
ADAS_m12	44.0 (3.30)	43.5 (12.5)	0.862	180
ADAS_m18	50.0 (.)	44.6 (12.8)	.	129
ADAS_m24	41.0 (.)	45.5 (12.9)	.	98

Baseline Characteristics for VN

	Both N=197	ChEI N=67	Memantine N=11	Neither N=6	P Value	N
AGE	74.8 (8.36)	77.9 (6.55)	76.2 (5.98)	79.2 (5.78)	0.026	281
EDCAT:					0.251	281
Less than HS	22 (11.2%)	16 (23.9%)	1 (9.09%)	0 (0.00%)		
HS graduate	95 (48.2%)	30 (44.8%)	6 (54.5%)	3 (50.0%)		
College graduate	80 (40.6%)	21 (31.3%)	4 (36.4%)	3 (50.0%)		

© 2018 Kennedy RE et al. *JAMA Network Open*.

RACE: White	180 (91.4%)	60 (89.6%)	11 (100%)	6 (100%)	0.798	281
ETHNIC: Hispanic	6 (3.05%)	2 (2.99%)	2 (18.2%)	0 (0.00%)	0.137	281
SEX: Female	108 (54.8%)	44 (65.7%)	6 (54.5%)	4 (66.7%)	0.439	281
MARITAL: Married	148 (75.1%)	42 (62.7%)	10 (90.9%)	5 (83.3%)	0.114	281
TX: Placebo	103 (52.3%)	32 (47.8%)	6 (54.5%)	4 (66.7%)	0.823	281
ADAS_bl	30.1 (9.18)	26.4 (8.62)	30.8 (8.05)	30.6 (9.48)	0.030	281
ADAS_m06	31.9 (9.13)	27.8 (8.62)	33.3 (7.46)	30.5 (4.76)	0.042	197
ADAS_m12	35.0 (10.2)	30.0 (11.7)	33.5 (10.4)	34.1 (1.85)	0.087	166
ADAS_m18	35.3 (10.5)	32.3 (9.96)	31.2 (3.57)	40.2 (2.59)	0.300	122
ADAS_m24	36.8 (10.7)	30.2 (10.6)	38.0 (8.82)	35.2 (9.67)	0.048	104

Baseline Characteristics for ADNI

	Both N=109	ChEI N=59	Memantine N=5	Neither N=6	P Value	N
AGE	74.1 (8.15)	76.9 (6.23)	78.0 (9.43)	77.3 (5.09)	0.080	179
EDCAT:					0.015	179
Less than HS	5 (4.59%)	9 (15.3%)	2 (40.0%)	2 (33.3%)		
HS graduate	43 (39.4%)	24 (40.7%)	2 (40.0%)	2 (33.3%)		
College graduate	61 (56.0%)	26 (44.1%)	1 (20.0%)	2 (33.3%)		
RACE: White	102 (93.6%)	56 (94.9%)	5 (100%)	5 (83.3%)	0.608	179
ETHNIC: Hispanic	2 (1.83%)	0 (0.00%)	0 (0.00%)	1 (16.7%)	0.168	179
SEX: Female	42 (38.5%)	31 (52.5%)	5 (100%)	5 (83.3%)	0.003	179
MARITAL: Married	98 (89.9%)	45 (76.3%)	2 (40.0%)	2 (33.3%)	<0.001	179

© 2018 Kennedy RE et al. *JAMA Network Open*.

TX: Placebo	109 (100%)	59 (100%)	5 (100%)	6 (100%)	.	179
ADAS_bl	19.2 (6.29)	17.8 (5.85)	20.7 (9.29)	17.9 (9.95)	0.499	179
ADAS_m06	21.5 (7.89)	19.7 (7.30)	20.7 (7.94)	20.2 (12.6)	0.607	169
ADAS_m12	23.0 (8.98)	21.7 (8.74)	21.2 (7.11)	23.2 (15.2)	0.856	150
ADAS_m24	28.9 (12.3)	26.7 (11.5)	26.5 (8.17)	30.2 (13.2)	0.795	127

© 2018 Kennedy RE et al. *JAMA Network Open*.

© 2018 Kennedy RE et al. *JAMA Network Open*.

Percent taking ChEIs by year

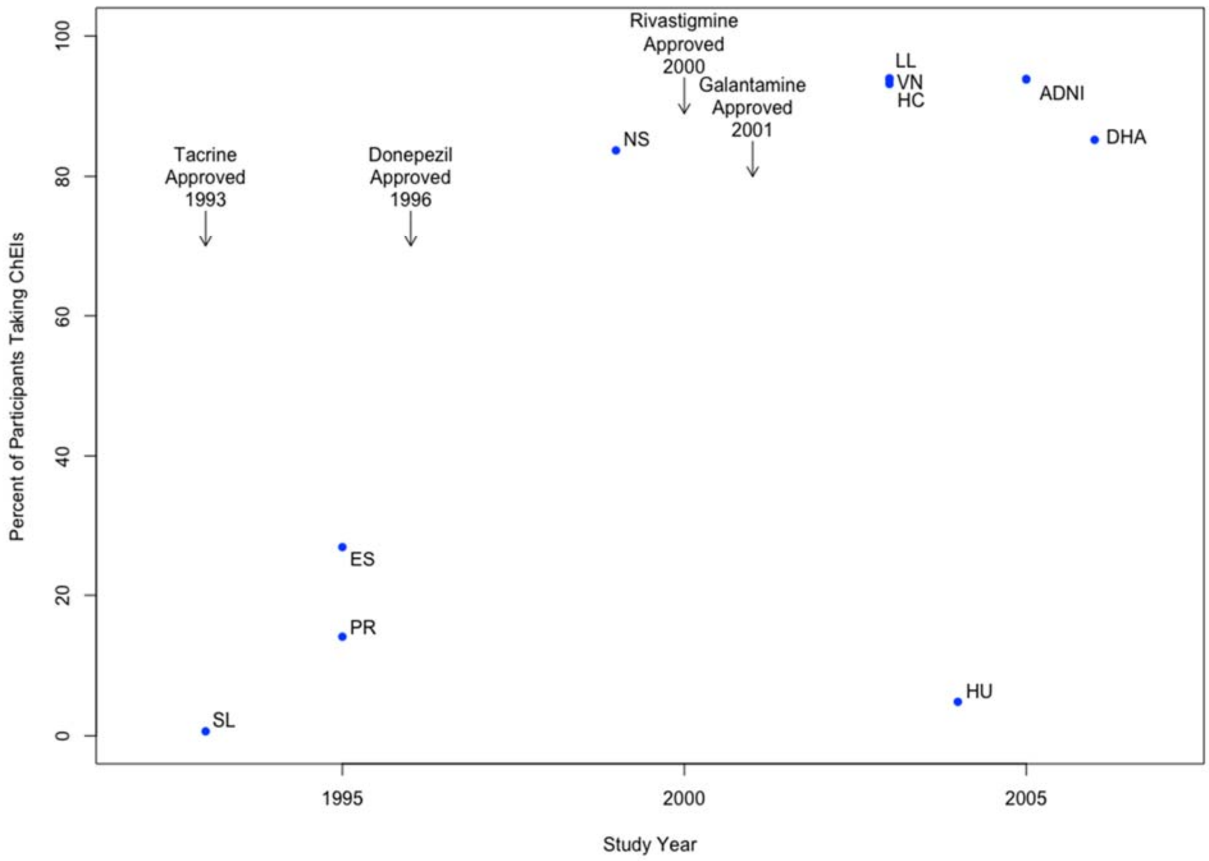

© 2018 Kennedy RE et al. *JAMA Network Open*.

© 2018 Kennedy RE et al. *JAMA Network Open*.

Percent taking each ChEI

```
## [1] 1829

##              [,1]
## donepezil    1428
## galantamine   308
## rivastigmine  183
## metrifonate     1
## tacrine         1

##                    [,1]
## donepezil    78.07545107
## galantamine  16.83980317
## rivastigmine 10.00546747
## metrifonate   0.05467469
## tacrine       0.05467469

## each.chei.count
##    1    2    3
## 1742   82    5

## each.chei.count
##           1           2           3
## 0.952433024 0.044833242 0.002733734
```

© 2018 Kennedy RE et al. *JAMA Network Open*.

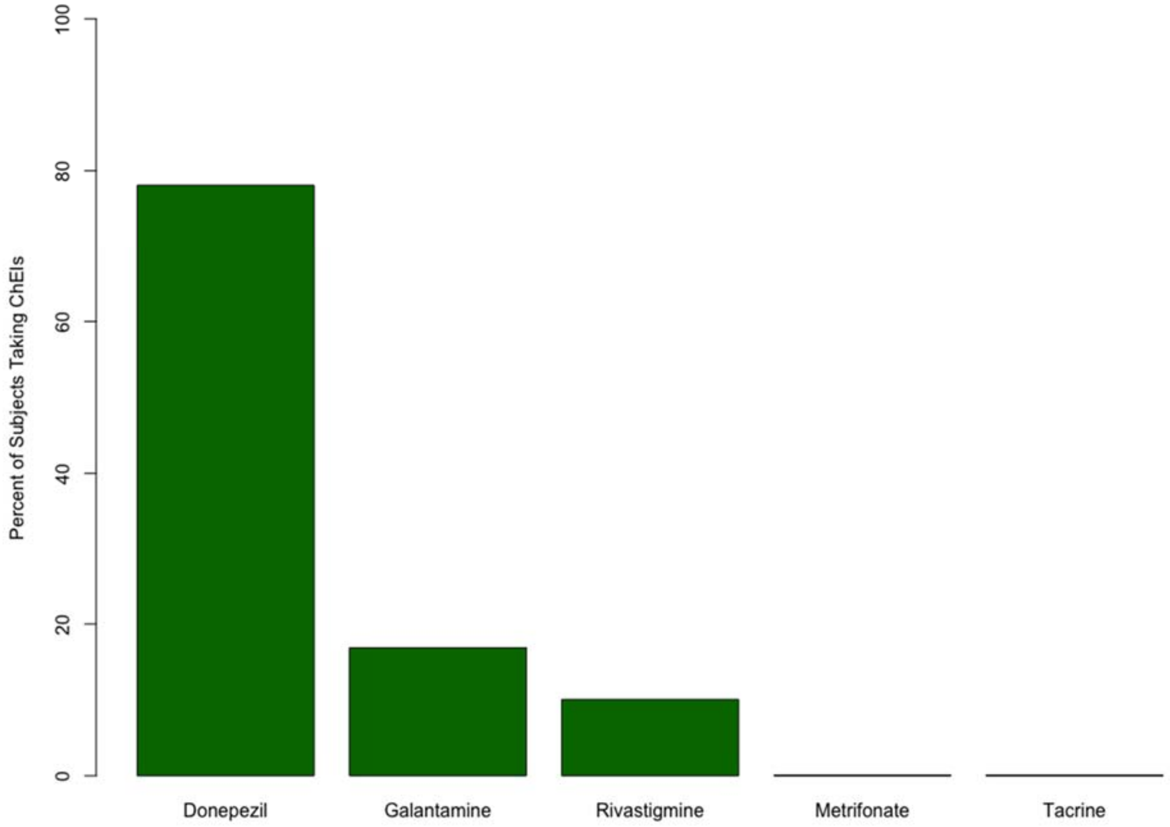

© 2018 Kennedy RE et al. *JAMA Network Open*.

© 2018 Kennedy RE et al. *JAMA Network Open*.

Percent taking memantine by year

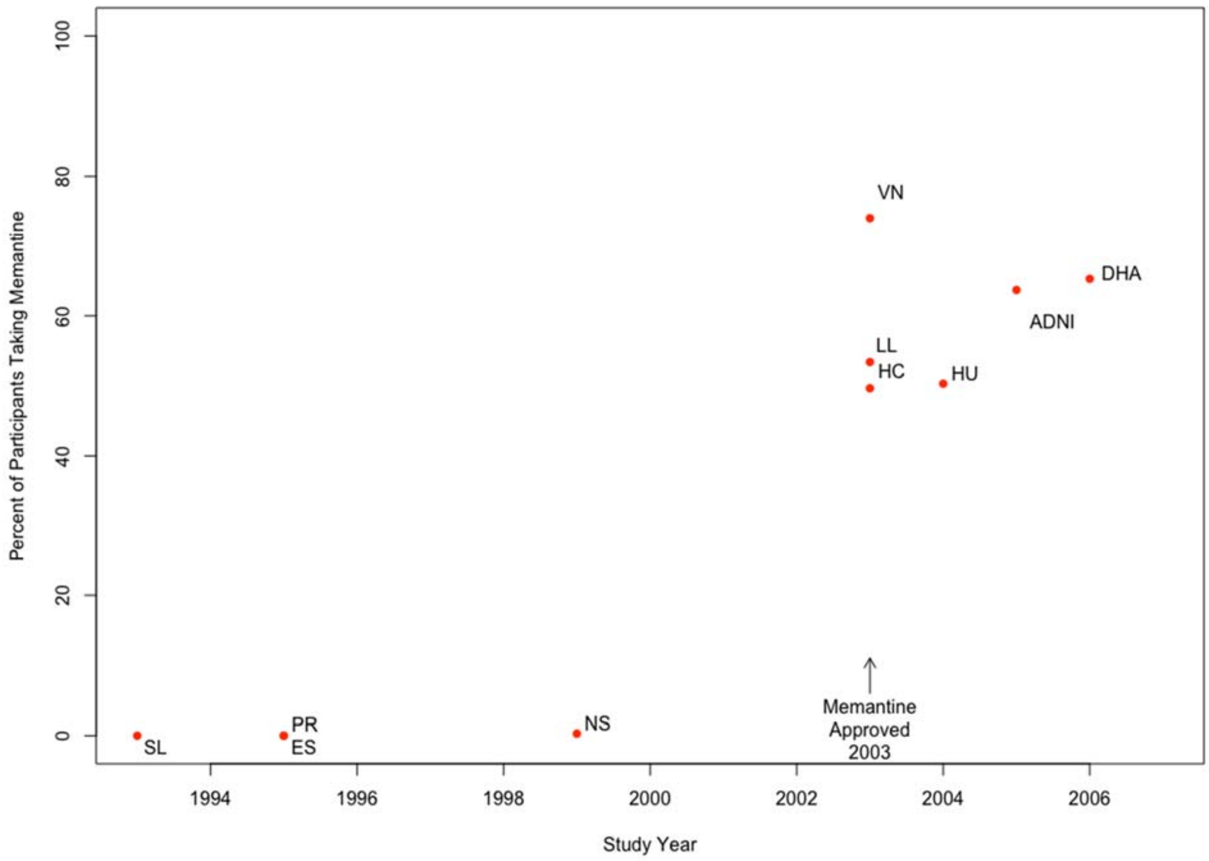

© 2018 Kennedy RE et al. *JAMA Network Open*.

Percent taking ChEIs and memantine by year

```
##
##      Both      ChEI Memantine   Neither
##       923       906       143       742

##
##      Both      ChEI Memantine   Neither
## 34.008843 33.382461  5.268976 27.339720
```

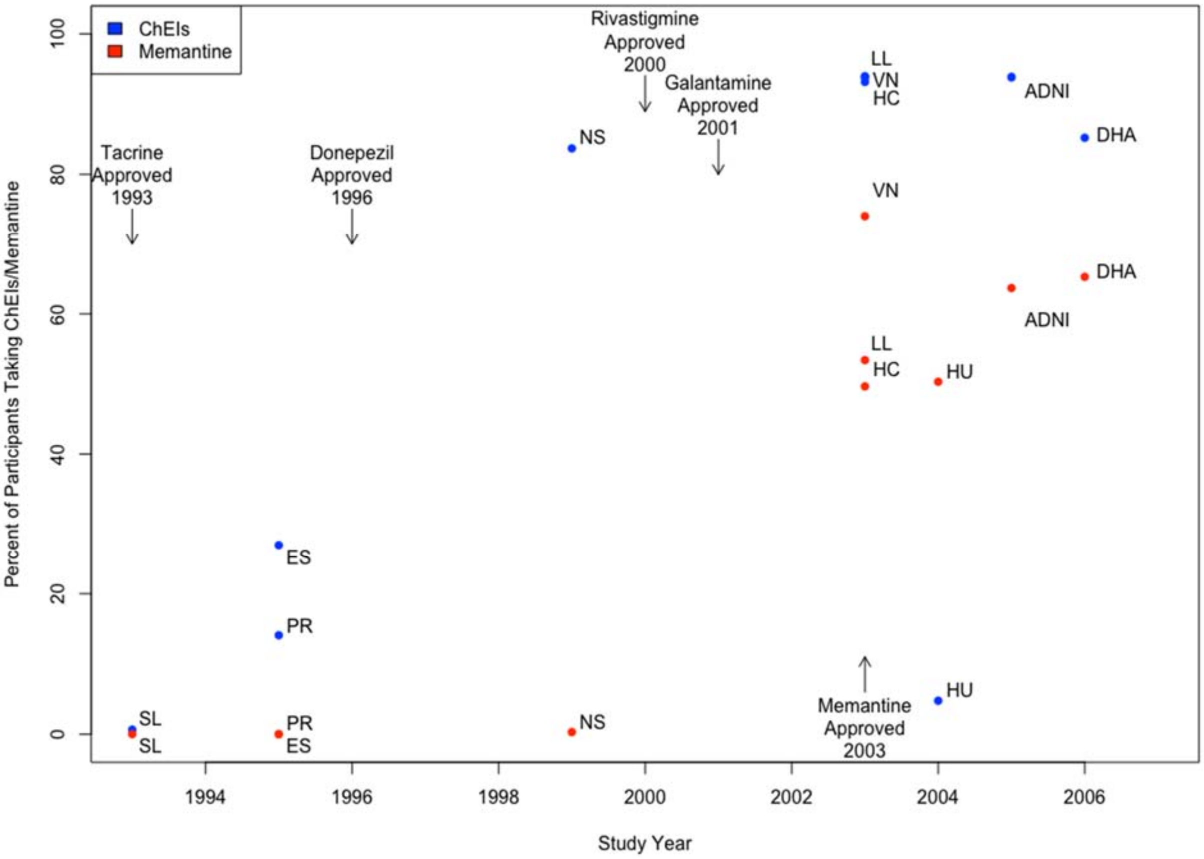

© 2018 Kennedy RE et al. *JAMA Network Open*.

Rates of decline across studies (excluding ADNI)

Meta-Analysis

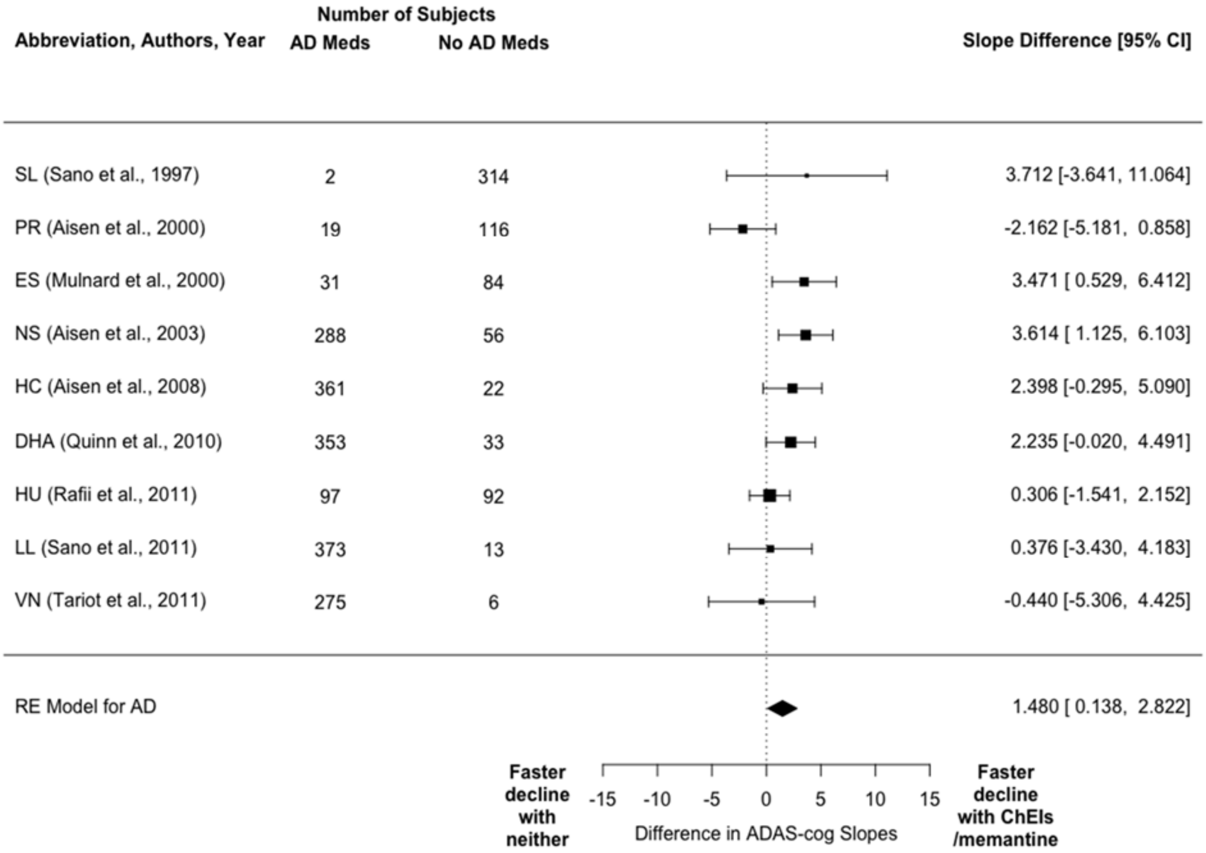

© 2018 Kennedy RE et al. *JAMA Network Open*.

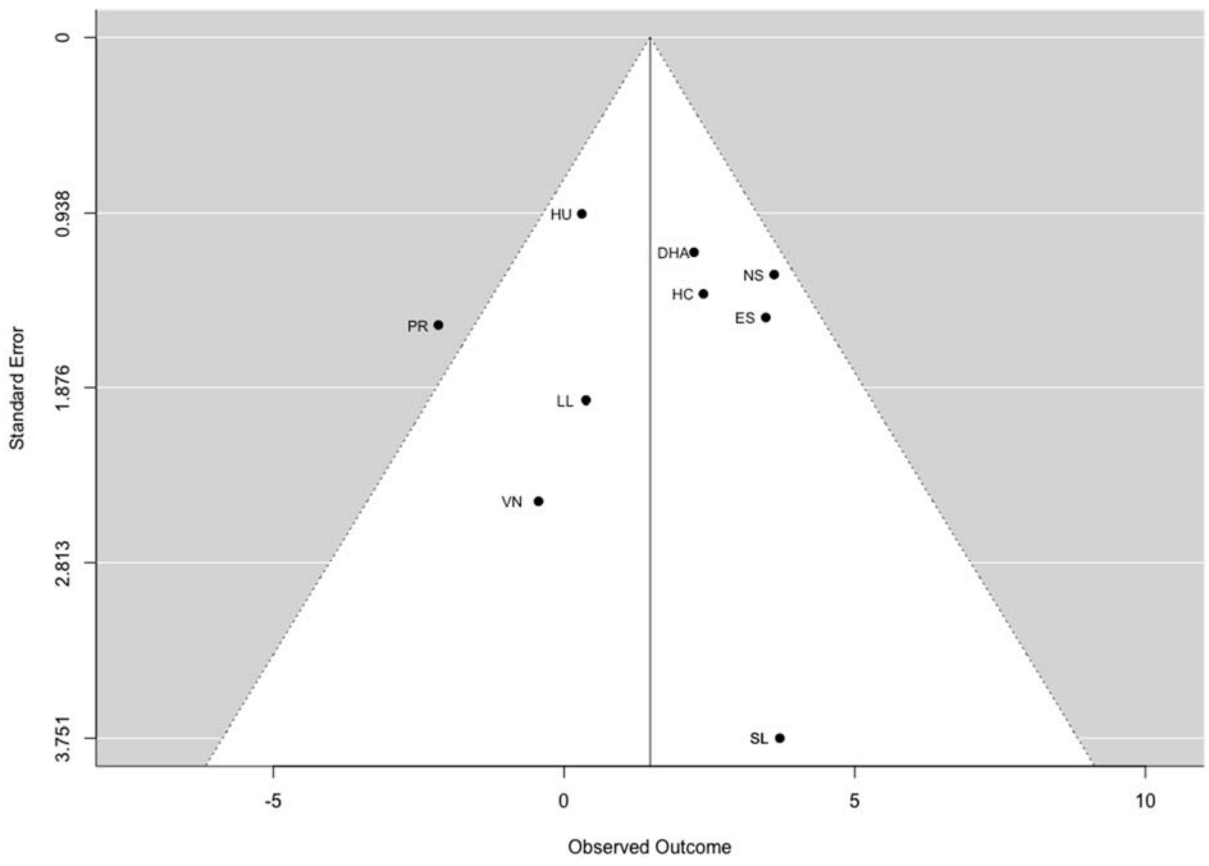

© 2018 Kennedy RE et al. *JAMA Network Open*.

Rates of decline across studies (presented as effect sizes)

Mixed Effects Models

```
## Study Name
## [1] "ADNI"
## Effect Sizes
##                      t       df          d
## AGE          0.88560642 171.0664 0.13542175
## EDUCATION    0.36548967 170.1638 0.05603656
## group1       0.26061972 175.8092 0.03931122
## month        2.04172270 139.8705 0.34527386
## group1:month 0.06693093 140.0256 0.01131236
## Study Name
## [1] "DHA"
## Effect Sizes
##                     t       df          d
## AGE           2.890042 359.0926  0.3050218
## EDUCATION    -1.273045 366.5684 -0.1329831
## group1        4.556713 377.4969  0.4690563
## month         2.895999 294.0238  0.3377826
## group1:month  1.937505 295.3313  0.2254852
## Study Name
## [1] "ES"
## Effect Sizes
##                       t        df           d
## AGE           1.19443908 109.04326  0.22876763
## EDUCATION    -0.43378813 105.68020 -0.08439386
## group1       -0.06235949 110.79660 -0.01184866
## month         6.11482550  94.51447  1.25795443
## group1:month  2.28943805  93.03537  0.47471706
## Study Name
## [1] "HC"
## Effect Sizes
##                     t       df          d
## AGE           3.079921 369.9991  0.3202353
## EDUCATION    -3.136811 369.6668 -0.3262970
## group1        4.080417 377.5313  0.4200085
## month         1.542196 331.1162  0.1695037
## group1:month  1.740498 330.5958  0.1914498
## Study Name
## [1] "HU"
## Effect Sizes
##                      t       df           d
## AGE          -0.4027470 183.4344 -0.05947328
## EDUCATION    -1.5101059 183.1461 -0.22317143
## group1        4.3159069 183.4569  0.63728703
## month         2.1794527 209.2660  0.30132011
```

© 2018 Kennedy RE et al. *JAMA Network Open*.

```
## group1:month  0.3237497 200.4569  0.04573292
## Study Name
## [1] "LL"
## Effect Sizes
##                      t       df           d
## AGE          -0.1267571 380.0840 -0.01300356
## EDUCATION     0.4921702 373.3606  0.05094259
## group1        1.3173025 382.9875  0.13462422
## month         2.6260264 264.5239  0.32292124
## group1:month  0.1944765 264.5884  0.02391176
## Study Name
## [1] "NS"
## Effect Sizes
##                      t       df           d
## AGE          -1.8590646 336.2817 -0.20275560
## EDUCATION    -0.4575322 336.5388 -0.04988088
## group1        1.3404055 339.0903  0.14558228
## month         1.8718855 233.7528  0.24486734
## group1:month  2.8346195 235.0970  0.36974404
## Study Name
## [1] "PR"
## Effect Sizes
##                       t        df            d
## AGE          -0.59784614 127.01989 -0.106092227
## EDUCATION    -1.65410896 122.24971 -0.299205920
## group1       -0.05281457 127.73206 -0.009346172
## month         9.29378230 104.74108  1.816201245
## group1:month -1.38875250  92.53538 -0.288736008
## Study Name
## [1] "SL"
## Effect Sizes
##                       t       df            d
## AGE          -2.22468100 309.9802 -0.252714862
## EDUCATION     0.03760996 310.7230  0.004267231
## group1       -1.61347700 304.6155 -0.184891437
## month        21.67285055 187.9064  3.162097829
## group1:month  0.98908684 185.4270  0.145270687
## Study Name
## [1] "VN"
## Effect Sizes
##                      t       df           d
## AGE          -1.8327867 273.5500 -0.22162745
## EDUCATION    -0.4616327 271.3396 -0.05604927
## group1       -0.5559867 269.3575 -0.06775321
## month         2.6688896 161.1334  0.42050173
## group1:month -0.1792771 160.8840 -0.02826821
```

© 2018 Kennedy RE et al. *JAMA Network Open*.

Meta-Analysis with metafor

```
##
## Random-Effects Model (k = 10; tau^2 estimator: REML)
##
## tau^2 (estimated amount of total heterogeneity): 0.0078 (SE = 0.0248)
## tau (square root of estimated tau^2 value):      0.0883
## I^2 (total heterogeneity / total variability):   13.27%
## H^2 (total variability / sampling variability):  1.15
##
## Test for Heterogeneity:
## Q(df = 9) = 8.6382, p-val = 0.4713
##
## Model Results:
##
## estimate      se    zval    pval   ci.lb   ci.ub
##   0.1673  0.0771  2.1705  0.0300  0.0162  0.3184  *
##
## ---
## Signif. codes:  0 '***' 0.001 '**' 0.01 '*' 0.05 '.' 0.1 ' ' 1
```

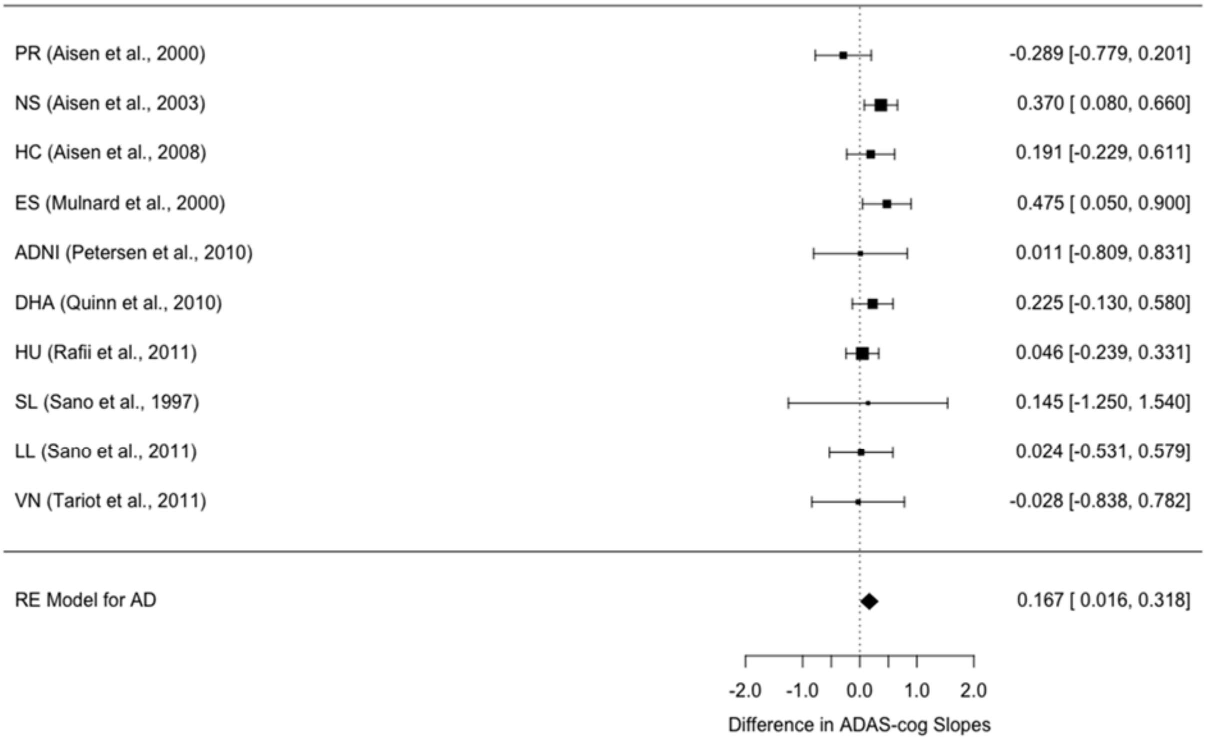

© 2018 Kennedy RE et al. *JAMA Network Open*.

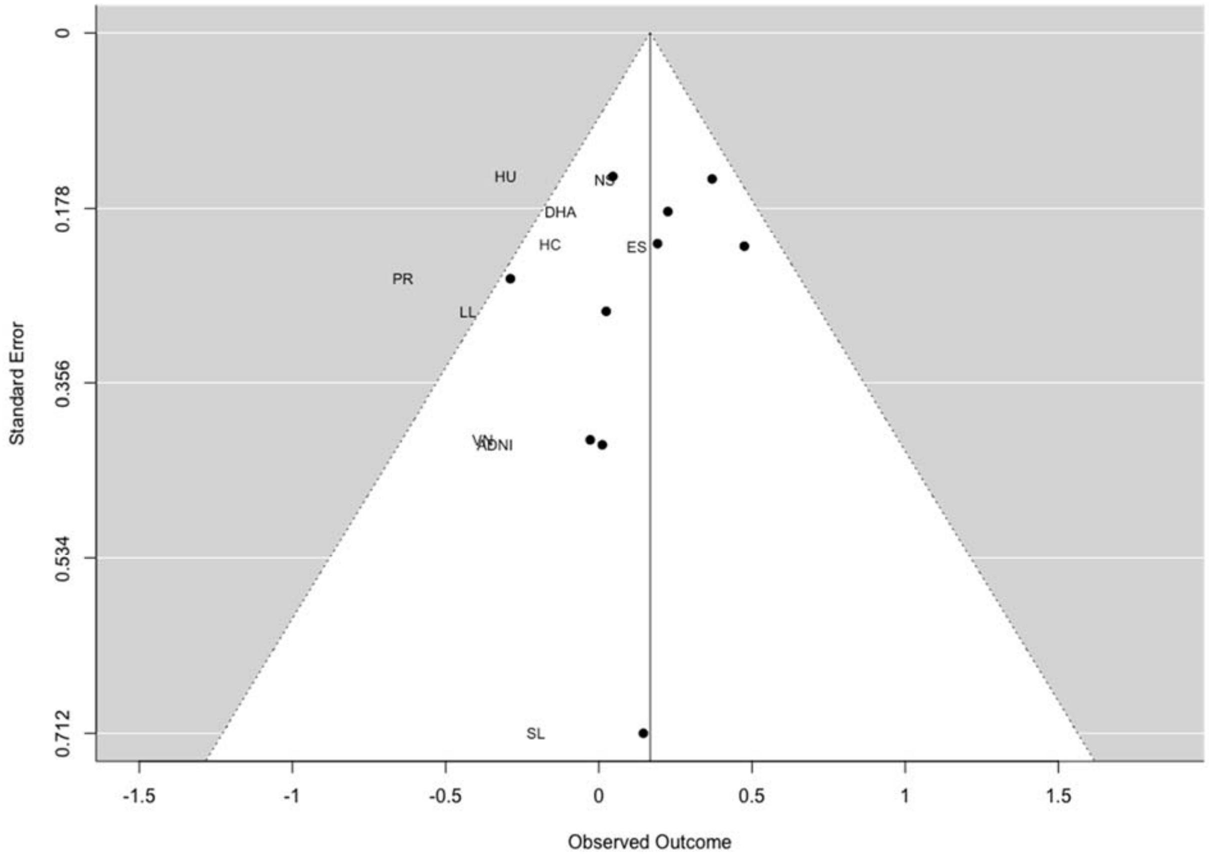

© 2018 Kennedy RE et al. *JAMA Network Open*.

Rates of decline for ChEIs vs. neither

Mixed Models

```
## Study Name
## [1] "ADNI"
## Full Mixed Effects Model
## Linear mixed model fit by maximum likelihood  ['lmerMod']
## Formula: ADAS ~ AGE + EDUCATION + group + month + group:month + (month |
##     UID)
##    Data: mixed.data
##
##      AIC      BIC   logLik deviance df.resid
##   1355.7   1389.5   -667.8   1335.7      207
##
## Scaled residuals:
##      Min       1Q   Median       3Q      Max
## -2.23216 -0.39292 -0.02574  0.36394  2.53793
##
## Random effects:
##  Groups   Name        Variance Std.Dev. Corr
##  UID      (Intercept) 30.229   5.498
##           month       14.023   3.745    0.49
##  Residual              9.804   3.131
## Number of obs: 217, groups:  UID, 65
##
## Fixed effects:
##             Estimate Std. Error t value
## (Intercept)  11.77585   10.17250   1.158
## AGE           0.05206    0.12220   0.426
## EDUCATION     0.16585    0.21942   0.756
## group1       -0.41759    2.63963  -0.158
## month         4.96049    2.07887   2.386
## group1:month -0.26826    2.17385  -0.123
##
## Correlation of Fixed Effects:
##             (Intr) AGE    EDUCAT group1 month
## AGE         -0.933
## EDUCATION   -0.276  0.015
## group1      -0.204  0.017 -0.158
## month        0.016  0.013  0.063 -0.182
## group1:mnth -0.020 -0.008 -0.061  0.190 -0.956
## P Value for Full vs. Reduced Model
## [1] 0.9019588
```

© 2018 Kennedy RE et al. *JAMA Network Open*.

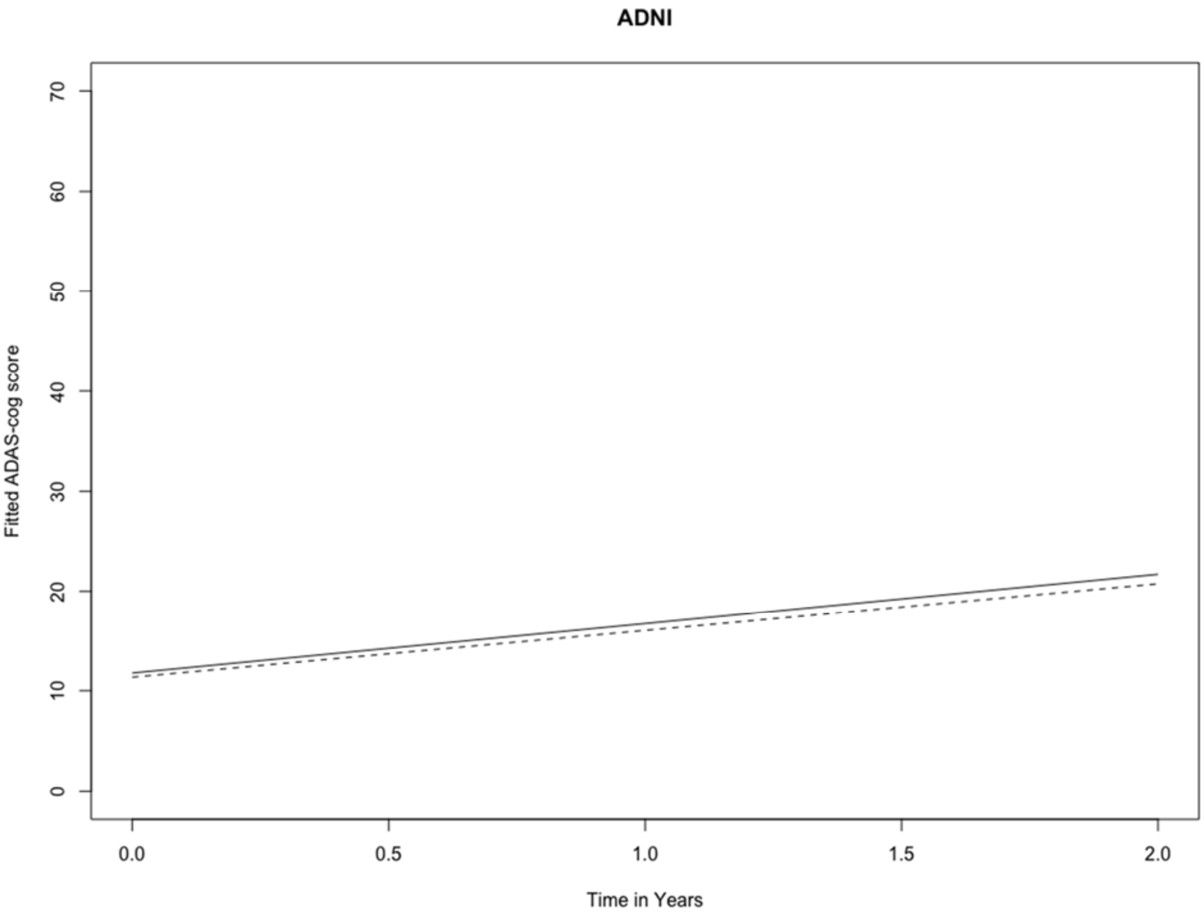


```
## Study Name
## [1] "DHA"
## Full Mixed Effects Model
## Linear mixed model fit by maximum likelihood  ['lmerMod']
## Formula: ADAS ~ AGE + EDUCATION + group + month + group:month + (month |
##     UID)
##    Data: mixed.data
##
##      AIC      BIC   logLik deviance df.resid
##   2872.3   2913.5  -1426.1   2852.3      445
##
## Scaled residuals:
##      Min       1Q   Median       3Q      Max
## -2.47423 -0.47238 -0.05433  0.44697  2.82137
##
## Random effects:
##  Groups   Name        Variance Std.Dev. Corr
##  UID      (Intercept) 39.49    6.284
##           month       20.82    4.563    0.64
##  Residual             11.14    3.338
```

© 2018 Kennedy RE et al. *JAMA Network Open*.

```
## Number of obs: 455, groups:  UID, 134
##
## Fixed effects:
##              Estimate Std. Error t value
## (Intercept)   5.91901    6.30850   0.938
## AGE           0.23081    0.06911   3.340
## EDUCATION    -0.46288    0.20682  -2.238
## group1        3.58044    1.39511   2.566
## month         3.20186    1.06703   3.001
## group1:month  0.76304    1.22349   0.624
##
## Correlation of Fixed Effects:
##             (Intr) AGE    EDUCAT group1 month
## AGE         -0.862
## EDUCATION   -0.476  0.008
## group1      -0.268  0.089  0.057
## month        0.055 -0.009  0.008 -0.230
## group1:mnth -0.040 -0.001 -0.006  0.264 -0.872
## P Value for Full vs. Reduced Model
## [1] 0.5351913
```

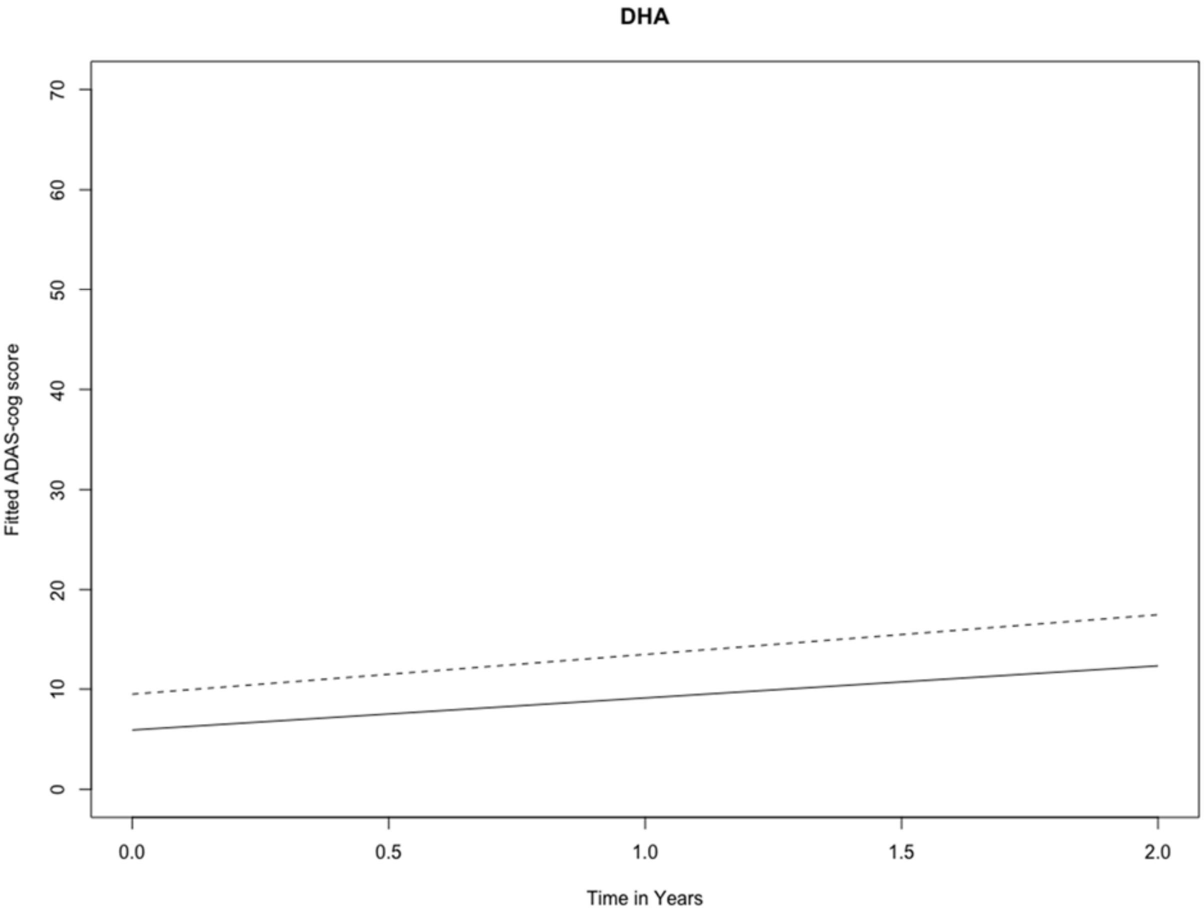

© 2018 Kennedy RE et al. *JAMA Network Open*.

```
## Study Name
## [1] "ES"
## Full Mixed Effects Model
## Linear mixed model fit by maximum likelihood  ['lmerMod']
## Formula: ADAS ~ AGE + EDUCATION + group + month + group:month + (month |
##     UID)
##    Data: mixed.data
##
##      AIC      BIC   logLik deviance df.resid
##   2004.6   2041.7   -992.3   1984.6      292
##
## Scaled residuals:
##      Min       1Q   Median       3Q      Max
## -2.47336 -0.52156 -0.01569  0.48441  2.62754
##
## Random effects:
##  Groups   Name        Variance Std.Dev. Corr
##  UID      (Intercept) 62.40    7.899
##           month       15.68    3.959    0.87
##  Residual             13.66    3.696
## Number of obs: 302, groups:  UID, 115
##
## Fixed effects:
##             Estimate Std. Error t value
## (Intercept)   14.9766     9.8512   1.520
## AGE            0.1429     0.1177   1.215
## EDUCATION     -0.1100     0.2496  -0.441
## group1        -0.1170     1.8339  -0.064
## month          4.8566     0.7858   6.181
## group1:month   3.4706     1.5006   2.313
##
## Correlation of Fixed Effects:
##             (Intr) AGE    EDUCAT group1 month
## AGE         -0.950
## EDUCATION   -0.442  0.155
## group1      -0.061  0.059 -0.135
## month        0.023 -0.002 -0.007 -0.106
## group1:mnth -0.018  0.004  0.012  0.208 -0.524
## P Value for Full vs. Reduced Model
## [1] 0.02347949
```

© 2018 Kennedy RE et al. *JAMA Network Open*.

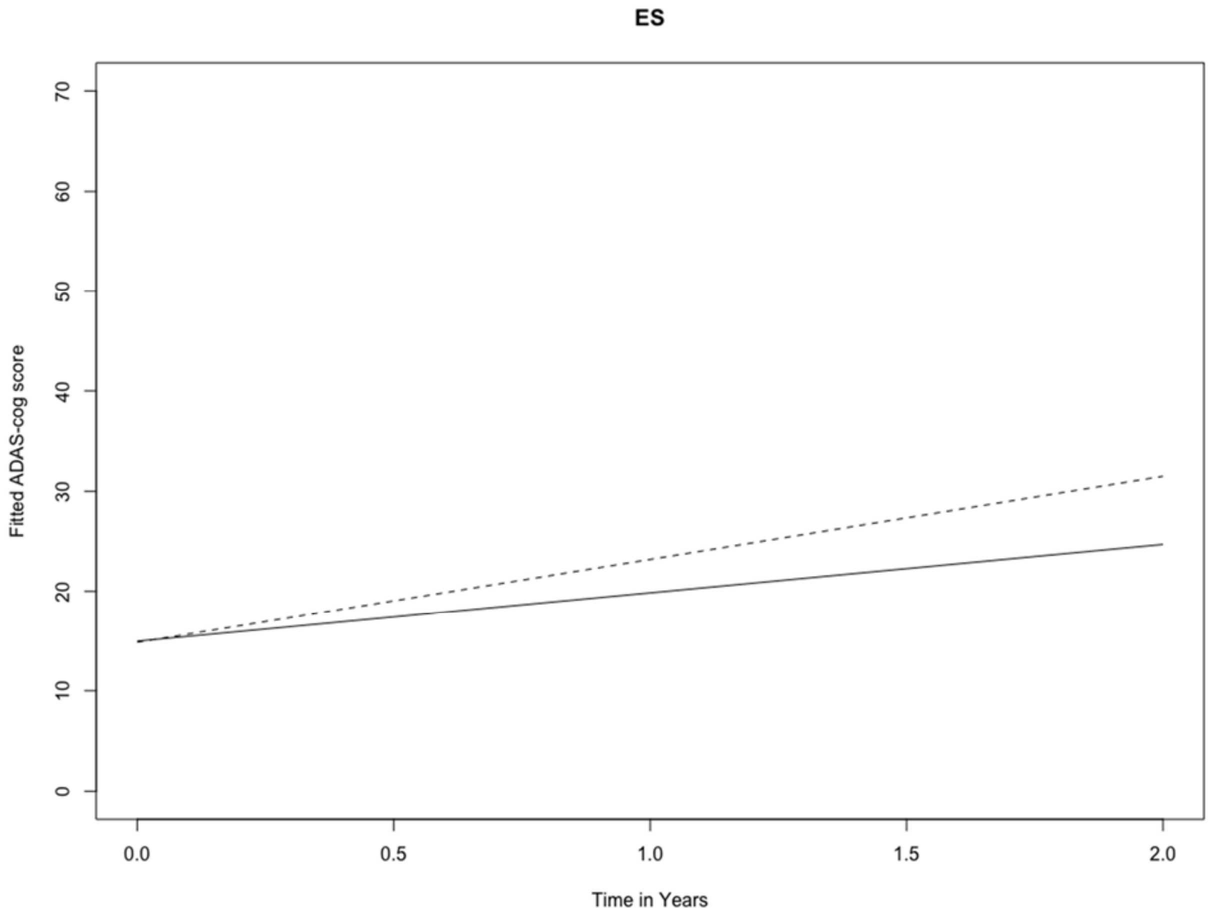


```
## Study Name
## [1] "HC"
## Full Mixed Effects Model
## Linear mixed model fit by maximum likelihood  ['lmerMod']
## Formula: ADAS ~ AGE + EDUCATION + group + month + group:month + (month |
##     UID)
##    Data: mixed.data
##
##      AIC      BIC   logLik deviance df.resid
##   4191.7   4236.8  -2085.9   4171.7      658
##
## Scaled residuals:
##     Min      1Q  Median      3Q     Max
## -3.2083 -0.5395 -0.0379  0.4835  2.7823
##
## Random effects:
##  Groups   Name        Variance Std.Dev. Corr
##  UID      (Intercept) 46.99    6.855
##           month       10.27    3.205    0.56
##  Residual             11.58    3.403
```

© 2018 Kennedy RE et al. *JAMA Network Open*.

```
## Number of obs: 668, groups:  UID, 193
##
## Fixed effects:
##              Estimate Std. Error t value
## (Intercept)   0.76595    6.15387   0.124
## AGE           0.22354    0.07229   3.092
## EDUCATION    -0.16638    0.17366  -0.958
## group1        4.99961    1.72001   2.907
## month         2.08521    1.12879   1.847
## group1:month  0.85686    1.18793   0.721
##
## Correlation of Fixed Effects:
##             (Intr) AGE    EDUCAT group1 month
## AGE         -0.893
## EDUCATION   -0.438  0.080
## group1      -0.068 -0.167 -0.073
## month        0.042 -0.015  0.005 -0.109
## group1:mnth -0.034  0.010 -0.009  0.119 -0.950
## P Value for Full vs. Reduced Model
## [1] 0.4708178
```

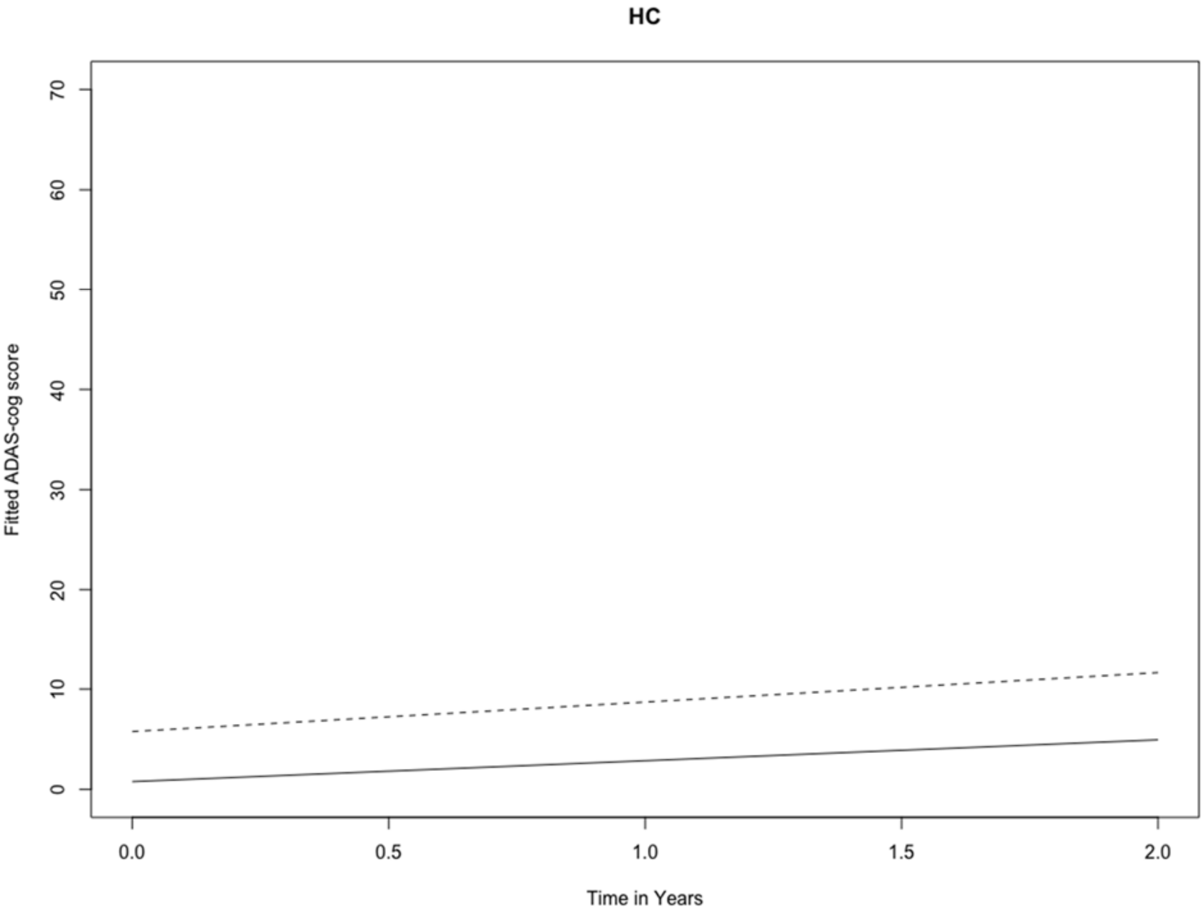

© 2018 Kennedy RE et al. *JAMA Network Open*.

```
## Study Name
## [1] "HU"
## Full Mixed Effects Model
## Linear mixed model fit by maximum likelihood  ['lmerMod']
## Formula: ADAS ~ AGE + EDUCATION + group + month + group:month + (month |
##     UID)
##    Data: mixed.data
##
##      AIC      BIC   logLik deviance df.resid
##   1548.8   1583.2   -764.4   1528.8      220
##
## Scaled residuals:
##      Min       1Q   Median       3Q      Max
## -1.93725 -0.53146  0.01131  0.45825  2.91594
##
## Random effects:
##  Groups   Name        Variance Std.Dev. Corr
##  UID      (Intercept) 77.459   8.801
##           month        5.217   2.284    1.00
##  Residual             14.922   3.863
## Number of obs: 230, groups:  UID, 94
##
## Fixed effects:
##             Estimate Std. Error t value
## (Intercept)  12.14502   10.27778   1.182
## AGE           0.14030    0.12028   1.166
## EDUCATION    -0.04411    0.31938  -0.138
## group1        0.78853    6.80071   0.116
## month         1.66161    0.70692   2.350
## group1:month  1.05060    3.49354   0.301
##
## Correlation of Fixed Effects:
##            (Intr) AGE    EDUCAT group1 month
## AGE        -0.910
## EDUCATION   -0.372 -0.036
## group1       0.025  0.021 -0.145
## month        0.040 -0.033 -0.001 -0.014
## group1:mnth -0.022  0.010  0.025  0.200 -0.203
## P Value for Full vs. Reduced Model
## [1] 0.7645879
```

© 2018 Kennedy RE et al. *JAMA Network Open*.

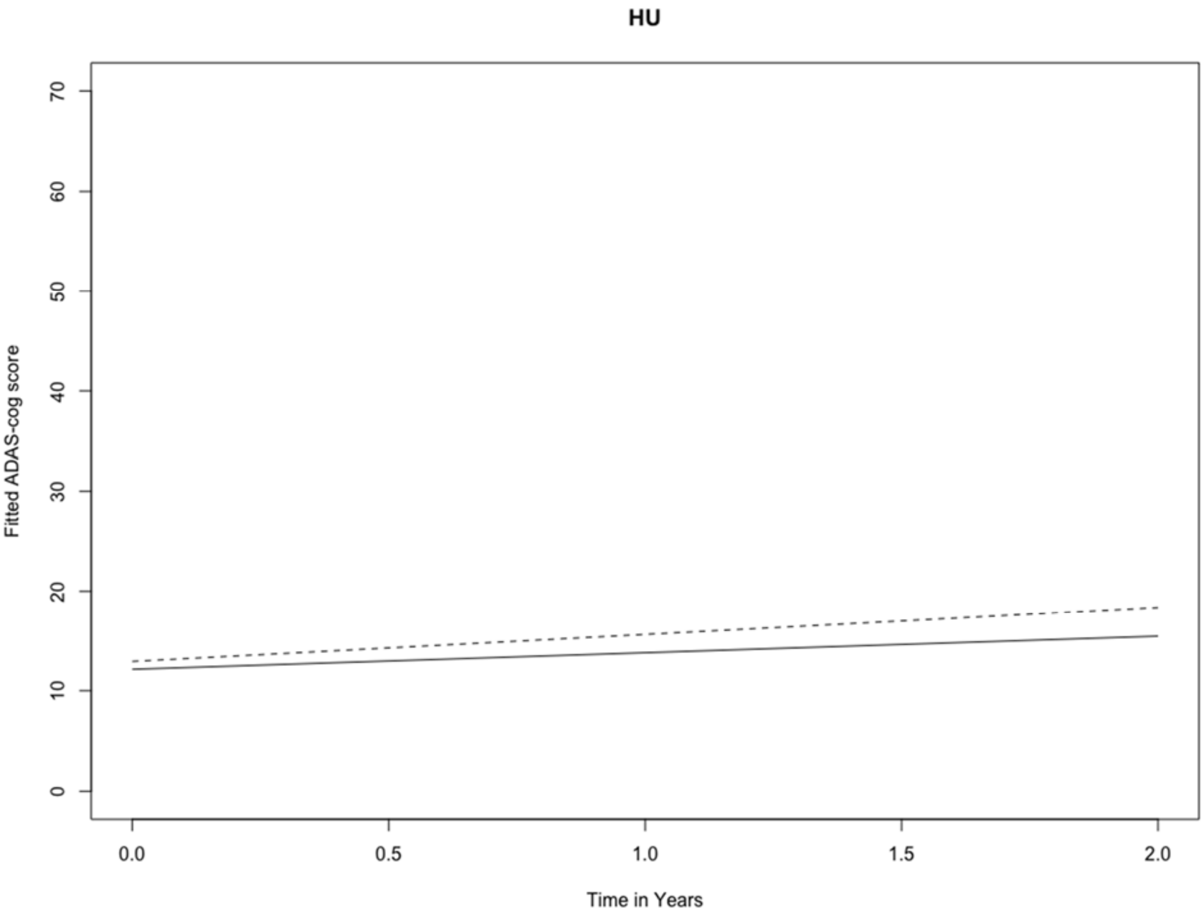


```
## Study Name
## [1] "LL"
## Full Mixed Effects Model
## Linear mixed model fit by maximum likelihood  ['lmerMod']
## Formula: ADAS ~ AGE + EDUCATION + group + month + group:month + (month |
##     UID)
##    Data: mixed.data
##
##      AIC      BIC   logLik deviance df.resid
##   3639.9   3683.3  -1810.0   3619.9      553
##
## Scaled residuals:
##     Min      1Q  Median      3Q     Max
## -2.4868 -0.4675 -0.0148  0.4783  3.2435
##
## Random effects:
##  Groups   Name        Variance Std.Dev. Corr
##  UID      (Intercept) 67.02    8.187
##           month       14.03    3.745    0.23
##  Residual             11.85    3.442
```

© 2018 Kennedy RE et al. *JAMA Network Open*.

```
## Number of obs: 563, groups:  UID, 180
##
## Fixed effects:
##              Estimate Std. Error t value
## (Intercept)  18.18100    7.07638   2.569
## AGE           0.01529    0.07846   0.195
## EDUCATION     0.06772    0.19688   0.344
## group1        0.89764    2.53179   0.355
## month         5.06810    1.68741   3.003
## group1:month -1.20203    1.74570  -0.689
##
## Correlation of Fixed Effects:
##             (Intr) AGE    EDUCAT group1 month
## AGE         -0.846
## EDUCATION   -0.479  0.087
## group1      -0.304 -0.066  0.071
## month       -0.004 -0.003 -0.001  0.017
## group1:mnth  0.003  0.003  0.001 -0.018 -0.967
## P Value for Full vs. Reduced Model
## [1] 0.4930244
```

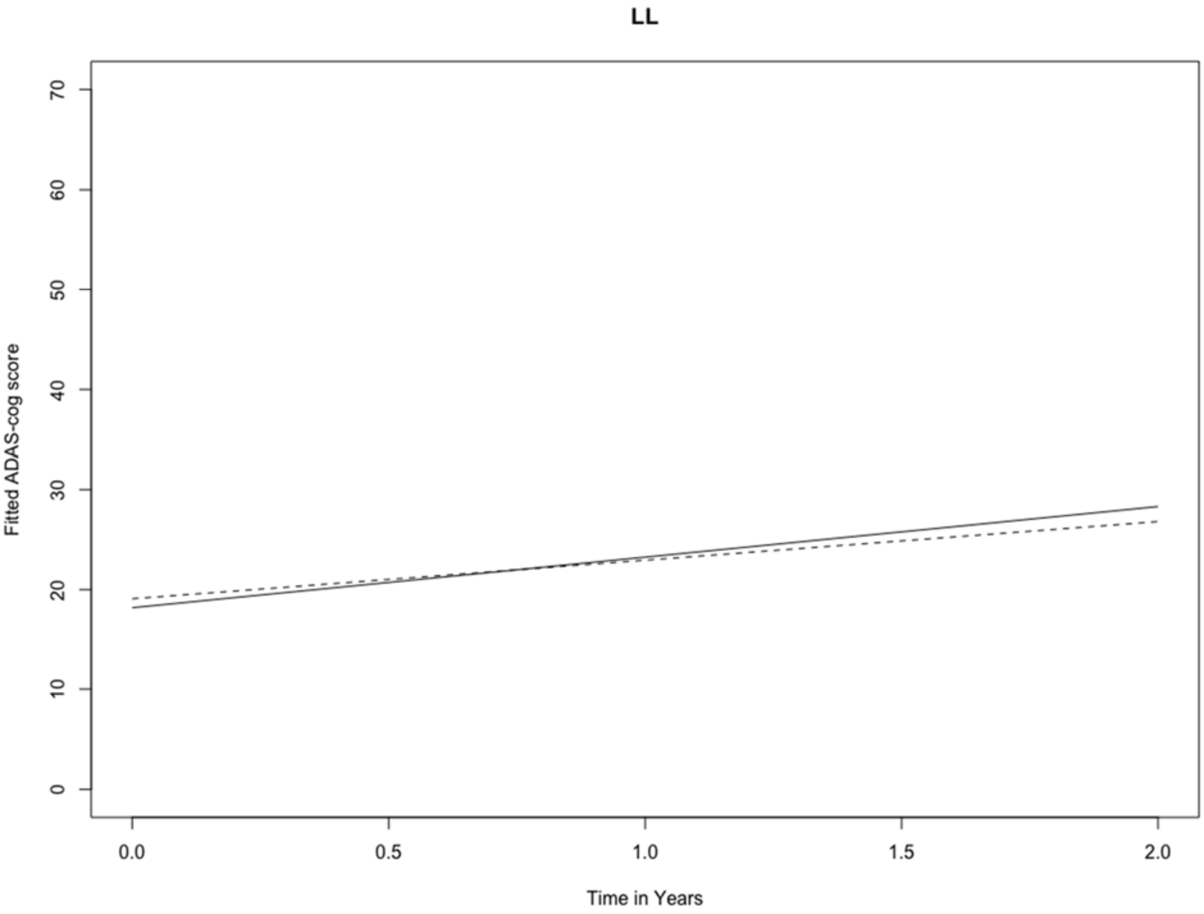

© 2018 Kennedy RE et al. *JAMA Network Open*.

```
## Study Name
## [1] "NS"
## Full Mixed Effects Model
## Linear mixed model fit by maximum likelihood  ['lmerMod']
## Formula: ADAS ~ AGE + EDUCATION + group + month + group:month + (month |
##     UID)
##    Data: mixed.data
##
##      AIC      BIC   logLik deviance df.resid
##   5706.5   5753.8  -2843.2   5686.5      833
##
## Scaled residuals:
##      Min       1Q   Median       3Q      Max
## -2.57400 -0.45745 -0.02595  0.44000  2.61174
##
## Random effects:
##  Groups   Name        Variance Std.Dev. Corr
##  UID      (Intercept) 79.28    8.904
##           month       22.27    4.719    0.48
##  Residual             14.05    3.748
## Number of obs: 843, groups:  UID, 343
##
## Fixed effects:
##              Estimate Std. Error t value
## (Intercept)  31.46520    6.02915   5.219
## AGE          -0.11060    0.06868  -1.610
## EDUCATION    -0.07899    0.15996  -0.494
## group1        1.90681    1.41642   1.346
## month         2.20864    1.17283   1.883
## group1:month  3.60857    1.27024   2.841
##
## Correlation of Fixed Effects:
##             (Intr) AGE    EDUCAT group1 month
## AGE         -0.912
## EDUCATION   -0.458  0.121
## group1      -0.280  0.129 -0.069
## month        0.009  0.004  0.003 -0.058
## group1:mnth -0.006 -0.005 -0.005  0.063 -0.923
## P Value for Full vs. Reduced Model
## [1] 0.004861028
```

© 2018 Kennedy RE et al. *JAMA Network Open*.

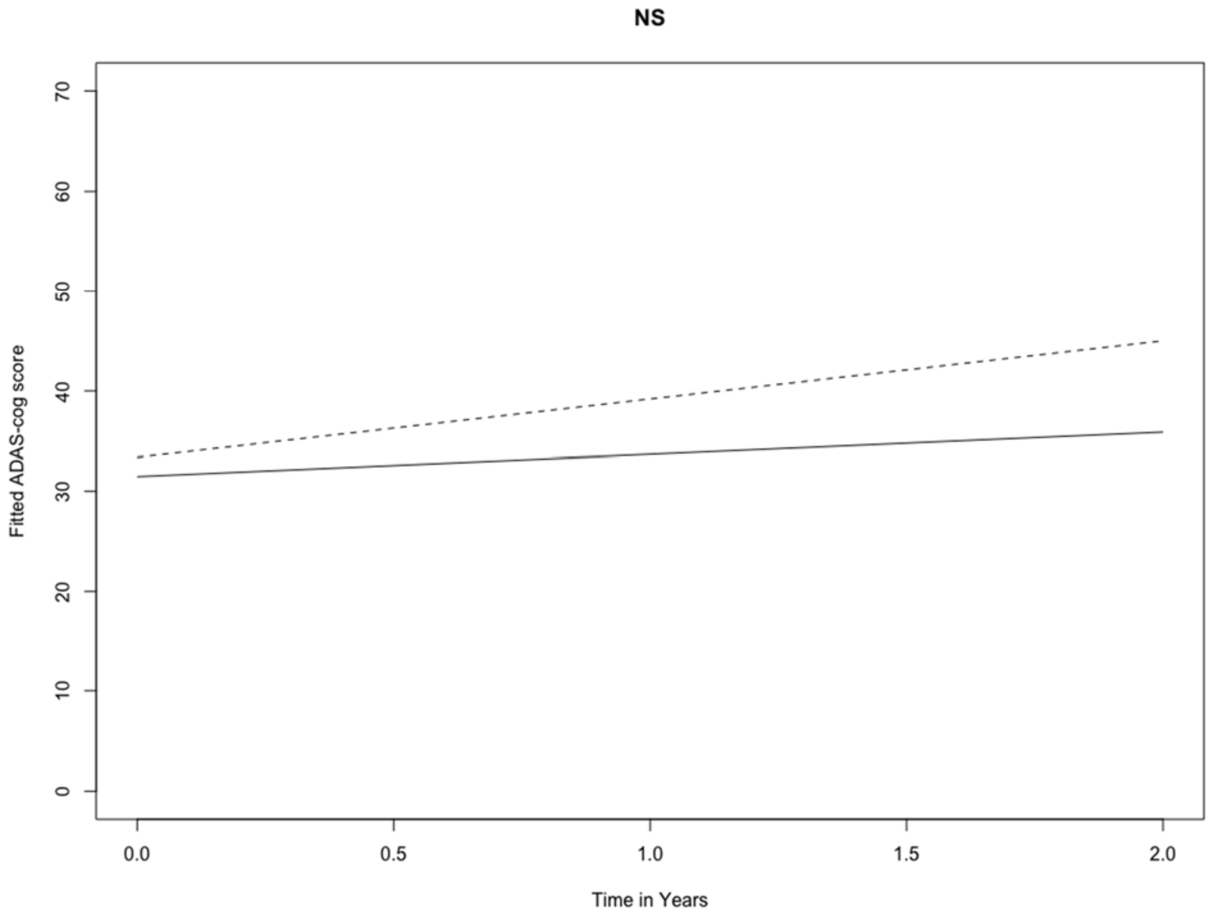


```
## Study Name
## [1] "PR"
## Full Mixed Effects Model
## Linear mixed model fit by maximum likelihood  ['lmerMod']
## Formula: ADAS ~ AGE + EDUCATION + group + month + group:month + (month |
##     UID)
##    Data: mixed.data
##
##      AIC      BIC   logLik deviance df.resid
##   2958.4   2999.2  -1469.2   2938.4      428
##
## Scaled residuals:
##      Min       1Q   Median       3Q      Max
## -2.76980 -0.50680 -0.02234  0.47104  2.93902
##
## Random effects:
##  Groups   Name        Variance Std.Dev. Corr
##  UID      (Intercept) 81.11    9.006
##           month       22.10    4.701    0.76
##  Residual             16.67    4.083
```

© 2018 Kennedy RE et al. *JAMA Network Open*.

```
## Number of obs: 438, groups:  UID, 135
##
## Fixed effects:
##              Estimate Std. Error t value
## (Intercept)  32.78688    9.01135   3.638
## AGE          -0.06482    0.10664  -0.608
## EDUCATION    -0.43416    0.25763  -1.685
## group1       -0.12742    2.39055  -0.053
## month         5.99618    0.63927   9.380
## group1:month -2.16187    1.54065  -1.403
##
## Correlation of Fixed Effects:
##             (Intr) AGE    EDUCAT group1 month
## AGE         -0.911
## EDUCATION   -0.521  0.137
## group1      -0.051 -0.008  0.049
## month        0.053 -0.014 -0.026 -0.117
## group1:mnth -0.032  0.014  0.017  0.356 -0.415
## P Value for Full vs. Reduced Model
## [1] 0.1620023
```

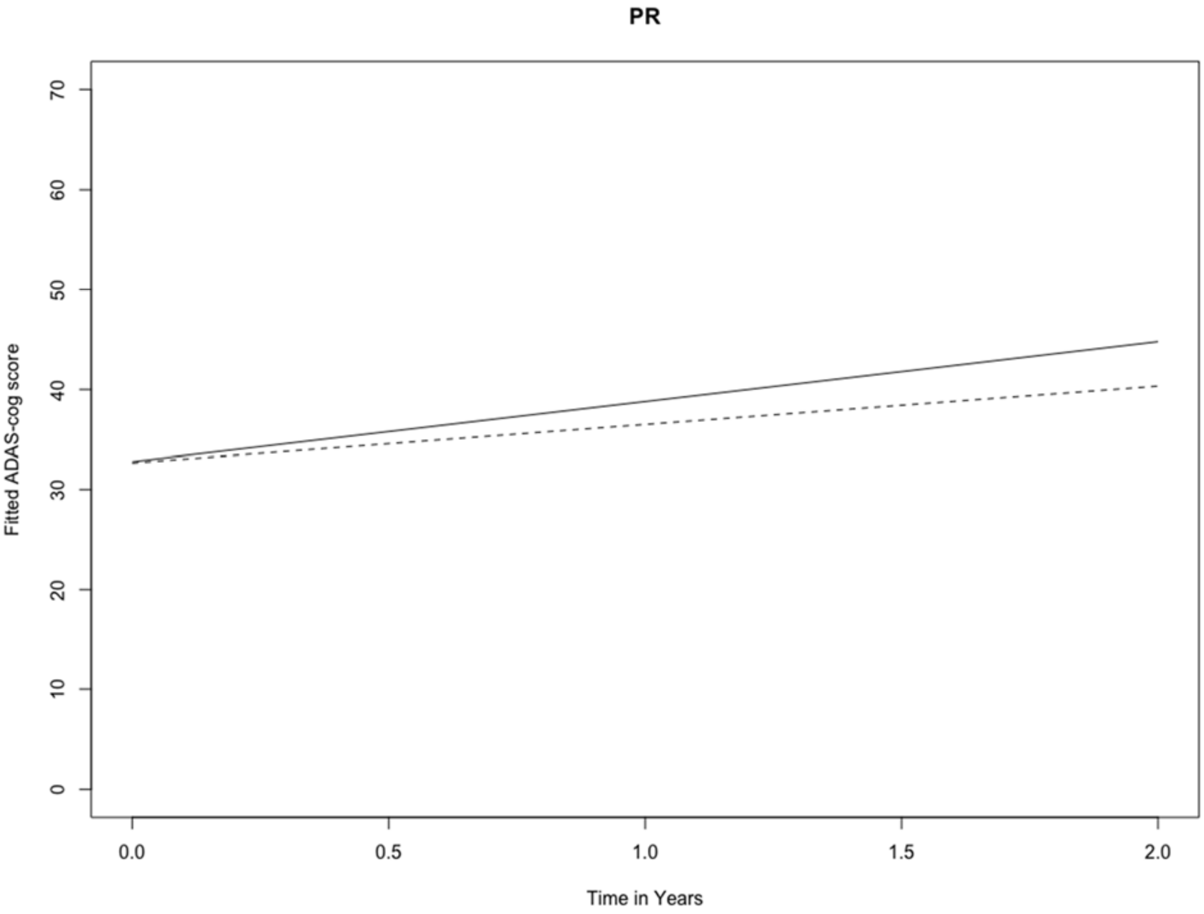

© 2018 Kennedy RE et al. *JAMA Network Open*.

```
## Study Name
## [1] "SL"
## Full Mixed Effects Model
## Linear mixed model fit by maximum likelihood  ['lmerMod']
## Formula: ADAS ~ AGE + EDUCATION + group + month + group:month + (month |
##     UID)
##    Data: mixed.data
##
##      AIC      BIC   logLik deviance df.resid
##   6467.8   6516.5  -3223.9   6447.8      951
##
## Scaled residuals:
##     Min      1Q  Median      3Q     Max
## -2.9713 -0.4627  0.0355  0.4802  3.1977
##
## Random effects:
##  Groups   Name        Variance Std.Dev. Corr
##  UID      (Intercept) 105.53   10.273
##           month        14.80    3.847   0.34
##  Residual              14.68    3.831
## Number of obs: 961, groups:  UID, 316
##
## Fixed effects:
##               Estimate Std. Error t value
## (Intercept)   51.372173   6.398754   8.028
## AGE           -0.172115   0.076926  -2.237
## EDUCATION      0.006988   0.187404   0.037
## group1       -12.686658   7.818356  -1.623
## month          7.769544   0.356626  21.786
## group1:month   3.711581   3.751239   0.989
##
## Correlation of Fixed Effects:
##             (Intr) AGE    EDUCAT group1 month
## AGE         -0.929
## EDUCATION   -0.488  0.145
## group1      -0.015  0.087 -0.191
## month        0.002  0.001  0.010 -0.007
## group1:mnth  0.001  0.000 -0.003  0.086 -0.095
## P Value for Full vs. Reduced Model
## [1] 0.3234868
```

© 2018 Kennedy RE et al. *JAMA Network Open*.

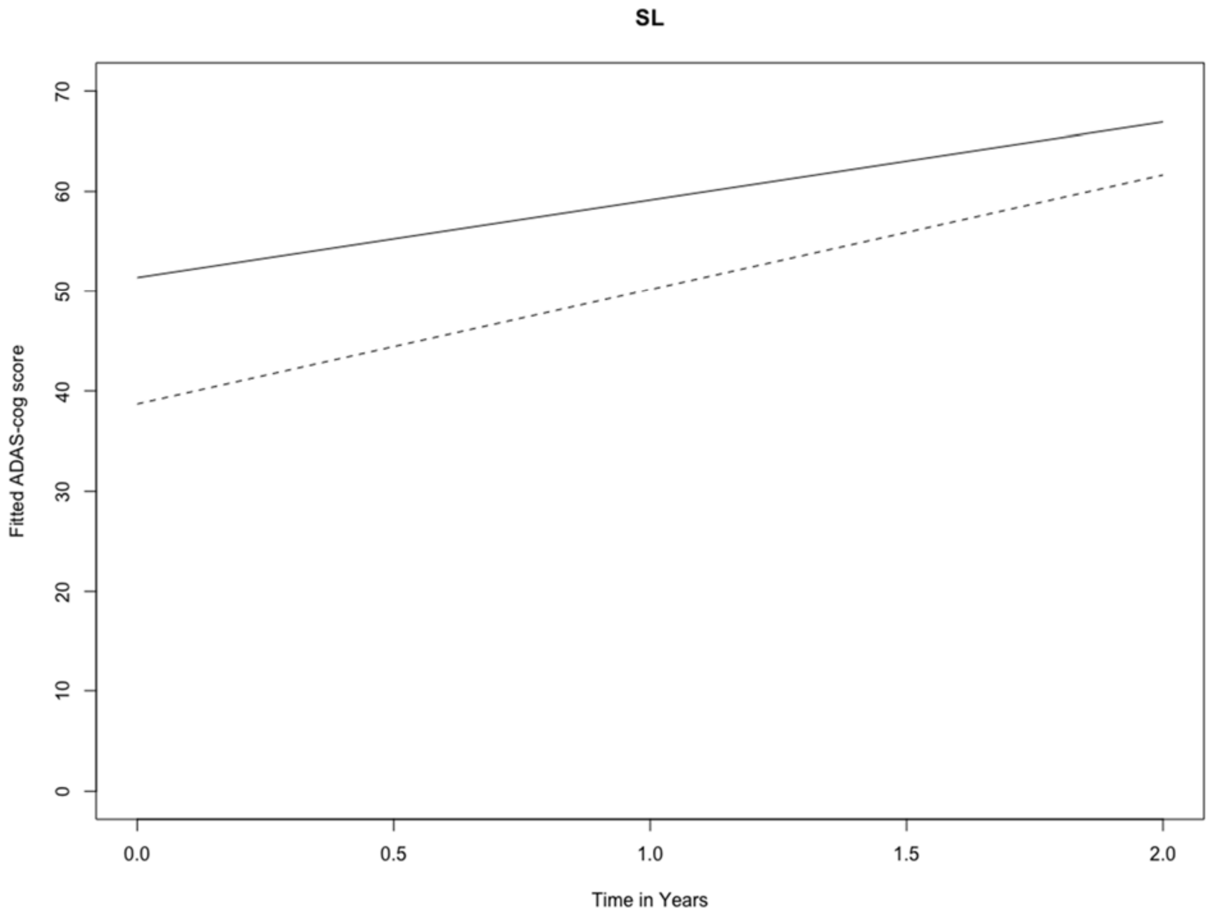


```
## Study Name
## [1] "VN"
## Full Mixed Effects Model
## Linear mixed model fit by maximum likelihood  ['lmerMod']
## Formula: ADAS ~ AGE + EDUCATION + group + month + group:month + (month |
##     UID)
##    Data: mixed.data
##
##      AIC      BIC   logLik deviance df.resid
##   1588.4   1623.1   -784.2   1568.4      227
##
## Scaled residuals:
##     Min      1Q  Median      3Q     Max
## -3.1131 -0.4569 -0.0712  0.4505  3.3913
##
## Random effects:
##  Groups   Name        Variance Std.Dev. Corr
##  UID      (Intercept) 58.55    7.652
##           month       26.75    5.172    0.39
##  Residual             15.17    3.895
```

© 2018 Kennedy RE et al. *JAMA Network Open*.

```
## Number of obs: 237, groups:  UID, 73
##
## Fixed effects:
##              Estimate Std. Error t value
## (Intercept)  23.36473   13.52038   1.728
## AGE           0.08565    0.15289   0.560
## EDUCATION     0.03185    0.26852   0.119
## group1       -3.97281    3.62194  -1.097
## month         7.02839    2.97828   2.360
## group1:month -1.57634    3.11021  -0.507
##
## Correlation of Fixed Effects:
##             (Intr) AGE    EDUCAT group1 month
## AGE         -0.920
## EDUCATION   -0.379  0.086
## group1      -0.348  0.063  0.180
## month        0.042 -0.021  0.001 -0.091
## group1:mnth -0.042  0.022 -0.003  0.096 -0.958
## P Value for Full vs. Reduced Model
## [1] 0.6131325
```

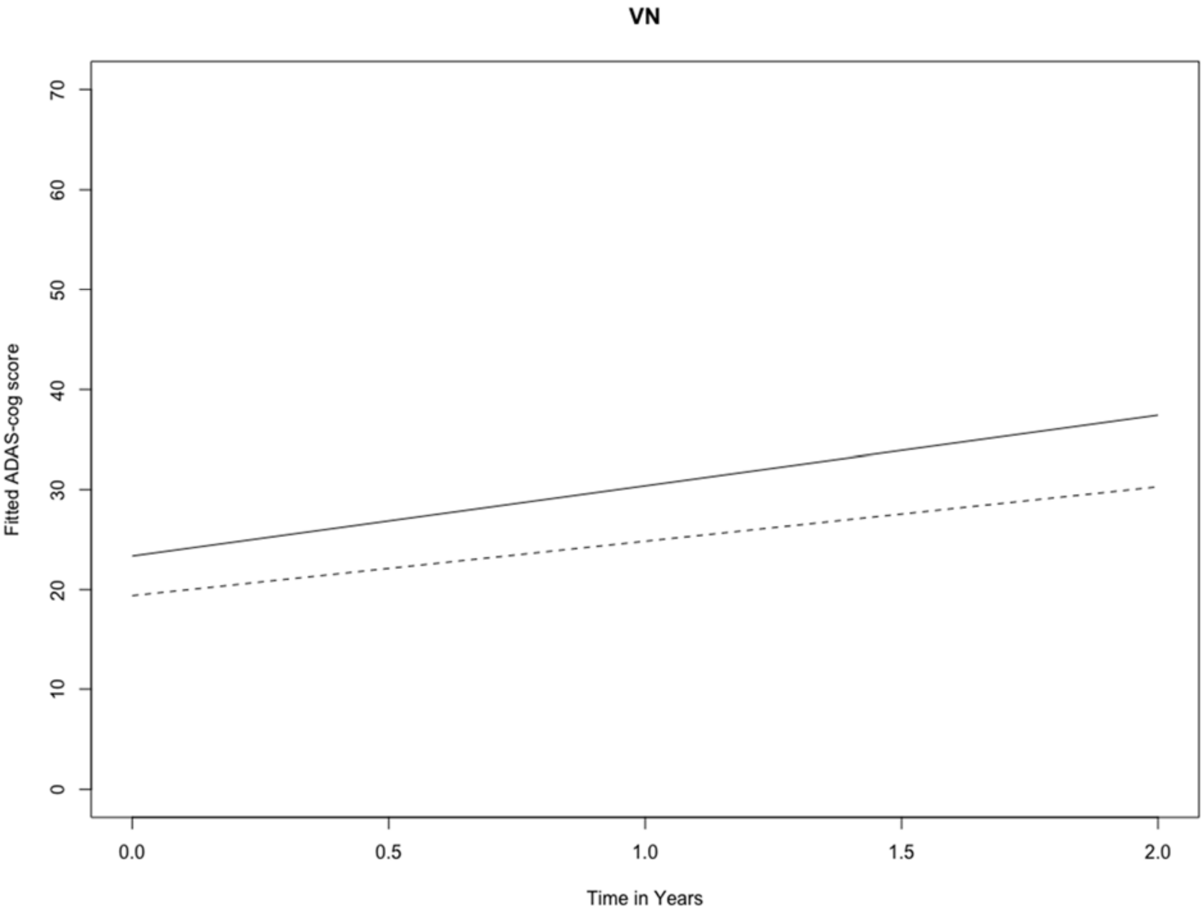

© 2018 Kennedy RE et al. *JAMA Network Open*.

```
##
## Random-Effects Model (k = 10; tau^2 estimator: REML)
##
## tau^2 (estimated amount of total heterogeneity): 2.1046 (SE = 2.3914)
## tau (square root of estimated tau^2 value):      1.4507
## I^2 (total heterogeneity / total variability):   42.37%
## H^2 (total variability / sampling variability):  1.74
##
## Test for Heterogeneity:
## Q(df = 9) = 14.3312, p-val = 0.1110
##
## Model Results:
##
## estimate      se    zval    pval    ci.lb   ci.ub
##   0.8889  0.7370  1.2061  0.2278  -0.5556  2.3334
##
## ---
## Signif. codes:  0 '***' 0.001 '**' 0.01 '*' 0.05 '.' 0.1 ' ' 1
```

Meta-Analysis with metafor

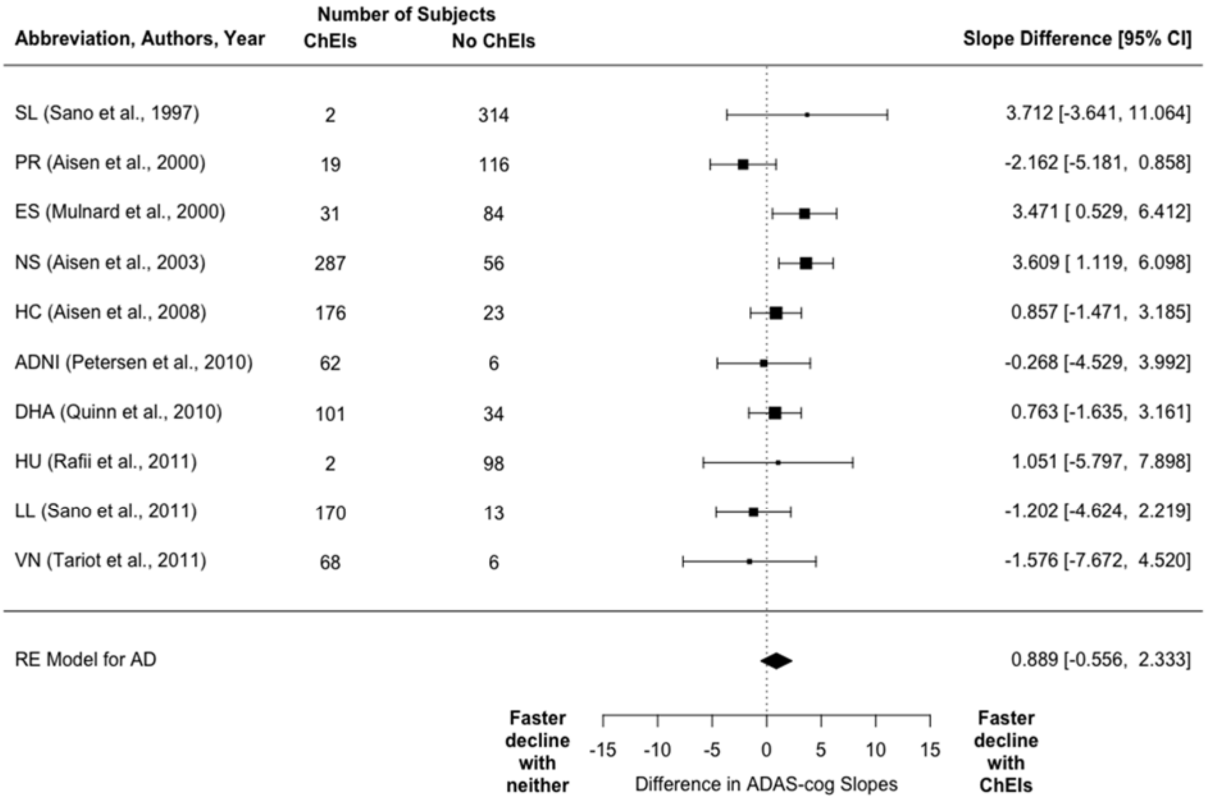

© 2018 Kennedy RE et al. *JAMA Network Open*.

Rates of decline for ChEIs vs. neither, with moderator of study year

Mixed Models

```
## Study Name
## [1] "ADNI"
## Full Mixed Effects Model
## Linear mixed model fit by maximum likelihood  ['lmerMod']
## Formula: ADAS ~ AGE + EDUCATION + group + month + group:month + (month |
##     UID)
##    Data: mixed.data
##
##      AIC      BIC   logLik deviance df.resid
##   1355.7   1389.5   -667.8   1335.7      207
##
## Scaled residuals:
##      Min       1Q   Median       3Q      Max
## -2.23216 -0.39292 -0.02574  0.36394  2.53793
##
## Random effects:
##  Groups   Name        Variance Std.Dev. Corr
##  UID      (Intercept) 30.229   5.498
##           month       14.023   3.745    0.49
##  Residual              9.804   3.131
## Number of obs: 217, groups:  UID, 65
##
## Fixed effects:
##             Estimate Std. Error t value
## (Intercept)  11.77585   10.17250   1.158
## AGE           0.05206    0.12220   0.426
## EDUCATION     0.16585    0.21942   0.756
## group1       -0.41759    2.63963  -0.158
## month         4.96049    2.07887   2.386
## group1:month -0.26826    2.17385  -0.123
##
## Correlation of Fixed Effects:
##            (Intr) AGE    EDUCAT group1 month
## AGE        -0.933
## EDUCATION  -0.276  0.015
## group1     -0.204  0.017 -0.158
## month       0.016  0.013  0.063 -0.182
## group1:mnth -0.020 -0.008 -0.061  0.190 -0.956
## P Value for Full vs. Reduced Model
## [1] 0.9019588
```

© 2018 Kennedy RE et al. *JAMA Network Open*.

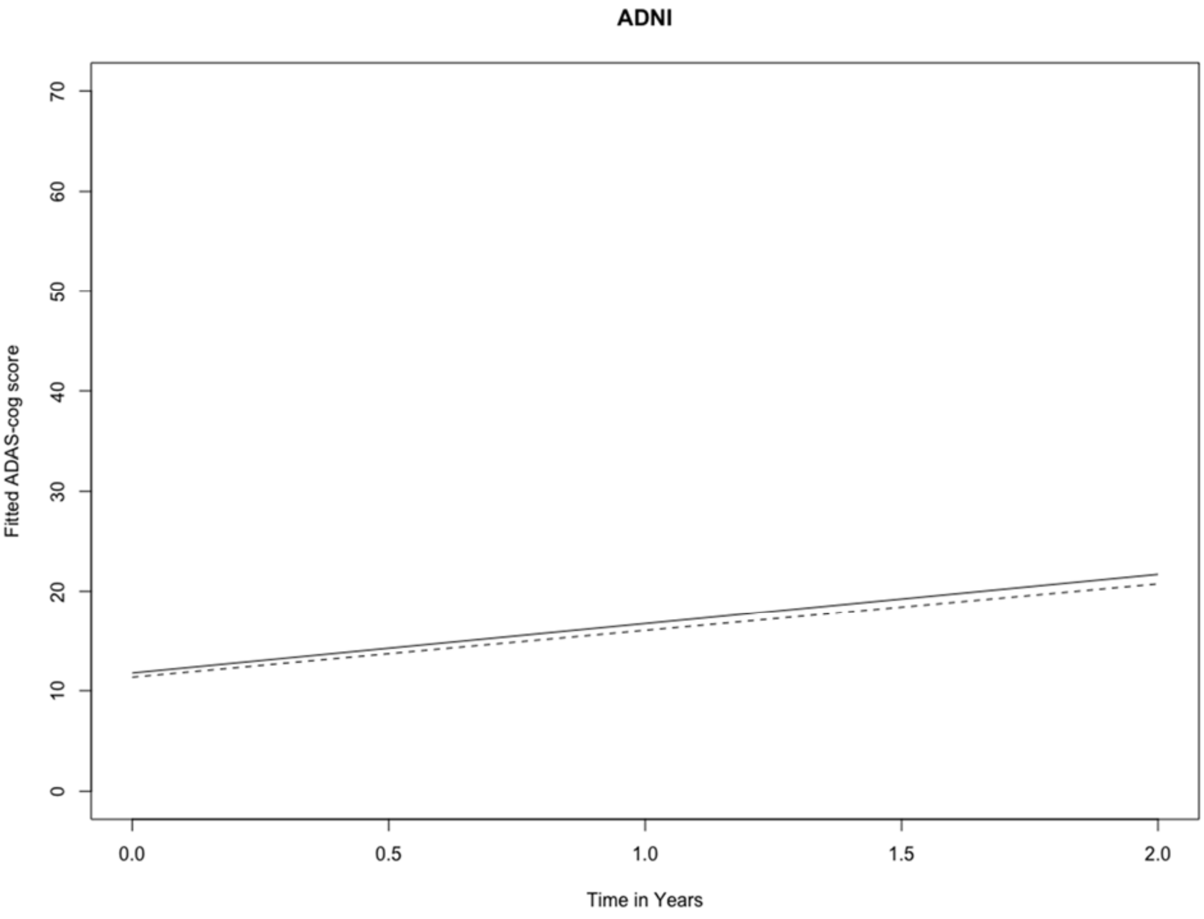


```
## Study Name
## [1] "DHA"
## Full Mixed Effects Model
## Linear mixed model fit by maximum likelihood  ['lmerMod']
## Formula: ADAS ~ AGE + EDUCATION + group + month + group:month + (month |
##     UID)
##    Data: mixed.data
##
##      AIC      BIC   logLik deviance df.resid
##   2872.3   2913.5  -1426.1   2852.3      445
##
## Scaled residuals:
##      Min       1Q   Median       3Q      Max
## -2.47423 -0.47238 -0.05433  0.44697  2.82137
##
## Random effects:
##  Groups   Name        Variance Std.Dev. Corr
##  UID      (Intercept) 39.49    6.284
##           month       20.82    4.563    0.64
##  Residual             11.14    3.338
```

© 2018 Kennedy RE et al. *JAMA Network Open*.

```
## Number of obs: 455, groups:  UID, 134
##
## Fixed effects:
##              Estimate Std. Error t value
## (Intercept)   5.91901    6.30850   0.938
## AGE           0.23081    0.06911   3.340
## EDUCATION    -0.46288    0.20682  -2.238
## group1        3.58044    1.39511   2.566
## month         3.20186    1.06703   3.001
## group1:month  0.76304    1.22349   0.624
##
## Correlation of Fixed Effects:
##             (Intr) AGE    EDUCAT group1 month
## AGE         -0.862
## EDUCATION   -0.476  0.008
## group1      -0.268  0.089  0.057
## month        0.055 -0.009  0.008 -0.230
## group1:mnth -0.040 -0.001 -0.006  0.264 -0.872
## P Value for Full vs. Reduced Model
## [1] 0.5351913
```

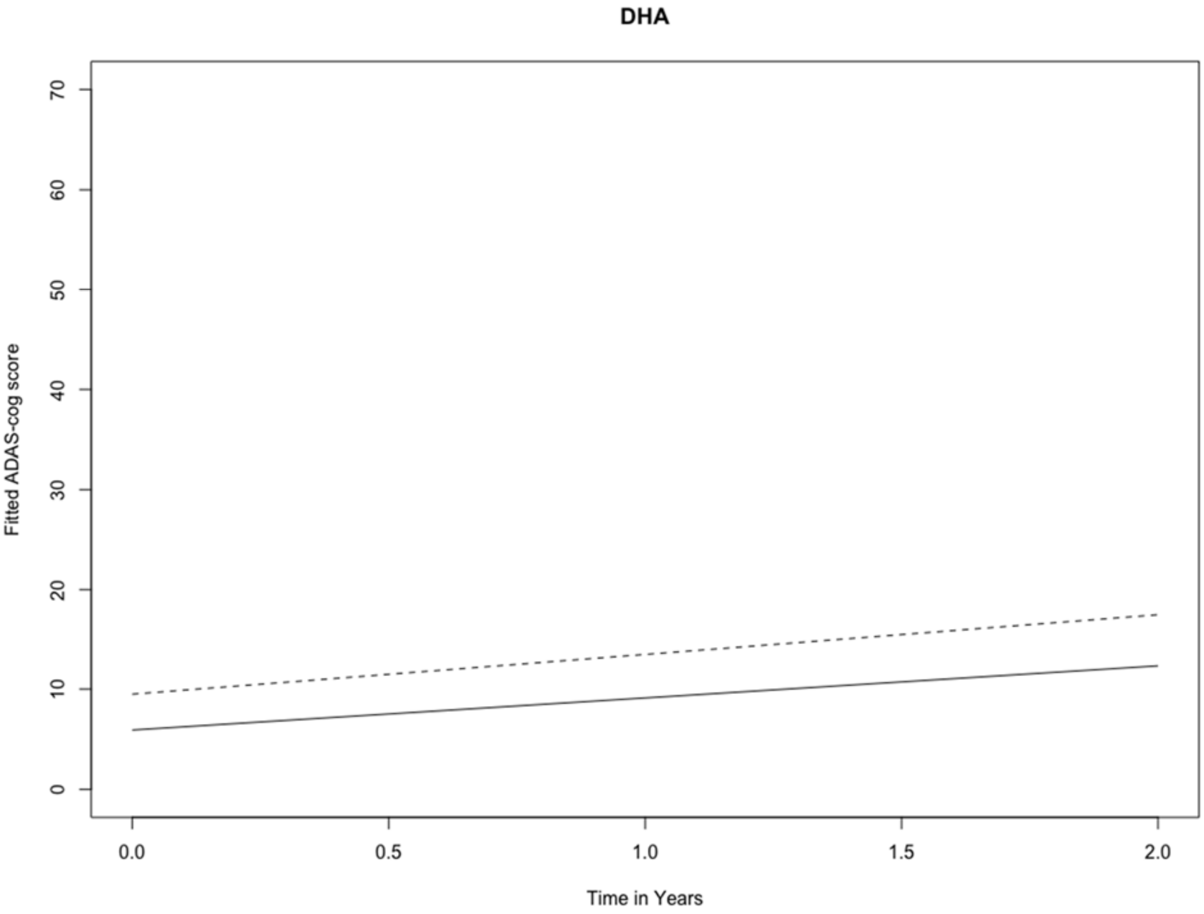

© 2018 Kennedy RE et al. *JAMA Network Open*.

```
## Study Name
## [1] "ES"
## Full Mixed Effects Model
## Linear mixed model fit by maximum likelihood  ['lmerMod']
## Formula: ADAS ~ AGE + EDUCATION + group + month + group:month + (month |
##     UID)
##    Data: mixed.data
##
##      AIC      BIC   logLik deviance df.resid
##   2004.6   2041.7   -992.3   1984.6      292
##
## Scaled residuals:
##      Min       1Q   Median       3Q      Max
## -2.47336 -0.52156 -0.01569  0.48441  2.62754
##
## Random effects:
##  Groups   Name        Variance Std.Dev. Corr
##  UID      (Intercept) 62.40    7.899
##           month       15.68    3.959    0.87
##  Residual             13.66    3.696
## Number of obs: 302, groups:  UID, 115
##
## Fixed effects:
##             Estimate Std. Error t value
## (Intercept)   14.9766     9.8512   1.520
## AGE            0.1429     0.1177   1.215
## EDUCATION     -0.1100     0.2496  -0.441
## group1        -0.1170     1.8339  -0.064
## month          4.8566     0.7858   6.181
## group1:month   3.4706     1.5006   2.313
##
## Correlation of Fixed Effects:
##             (Intr) AGE    EDUCAT group1 month
## AGE         -0.950
## EDUCATION   -0.442  0.155
## group1      -0.061  0.059 -0.135
## month        0.023 -0.002 -0.007 -0.106
## group1:mnth -0.018  0.004  0.012  0.208 -0.524
## P Value for Full vs. Reduced Model
## [1] 0.02347949
```

© 2018 Kennedy RE et al. *JAMA Network Open*.

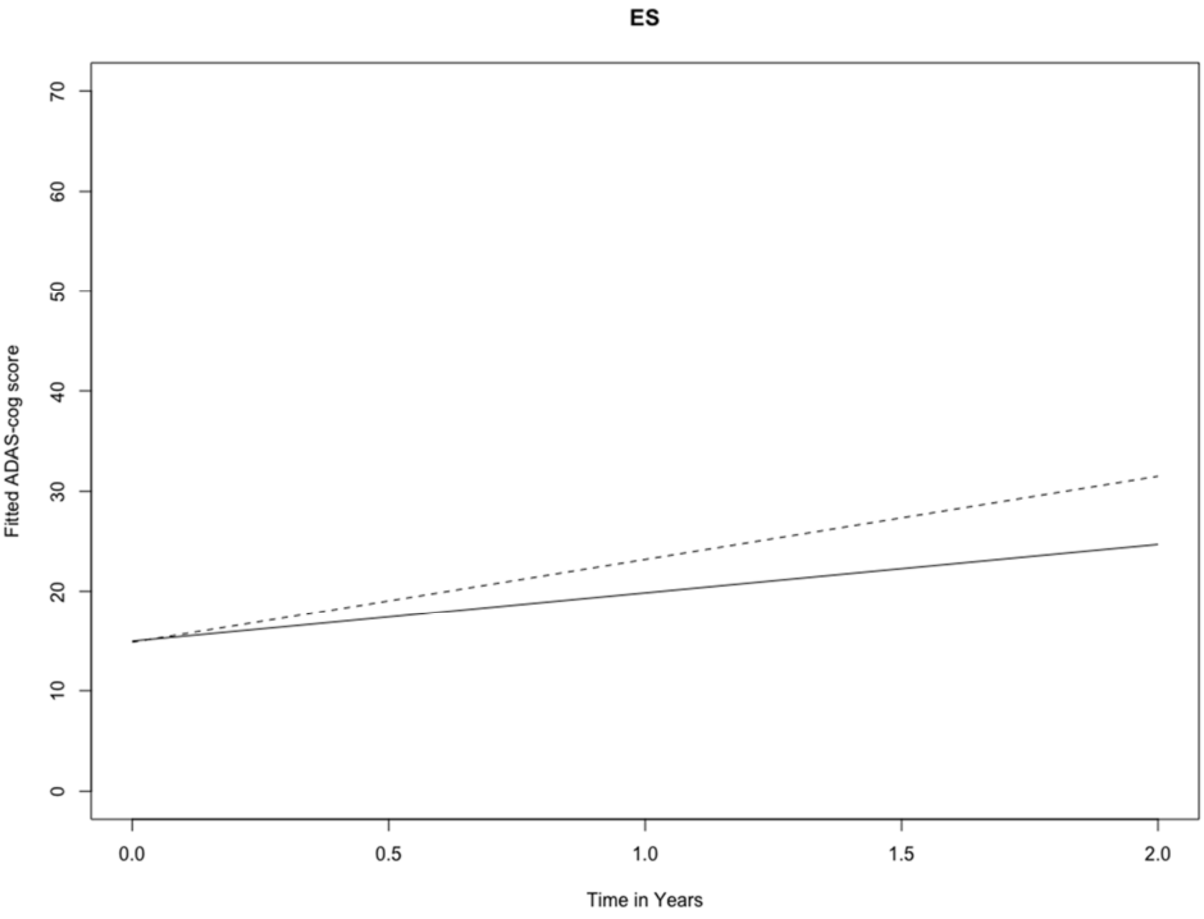


```
## Study Name
## [1] "HC"
## Full Mixed Effects Model
## Linear mixed model fit by maximum likelihood  ['lmerMod']
## Formula: ADAS ~ AGE + EDUCATION + group + month + group:month + (month |
##     UID)
##    Data: mixed.data
##
##      AIC      BIC   logLik deviance df.resid
##   4191.7   4236.8  -2085.9   4171.7      658
##
## Scaled residuals:
##     Min      1Q  Median      3Q     Max
## -3.2083 -0.5395 -0.0379  0.4835  2.7823
##
## Random effects:
##  Groups   Name        Variance Std.Dev. Corr
##  UID      (Intercept) 46.99    6.855
##           month       10.27    3.205    0.56
##  Residual             11.58    3.403
```

© 2018 Kennedy RE et al. *JAMA Network Open*.

```
## Number of obs: 668, groups:  UID, 193
##
## Fixed effects:
##              Estimate Std. Error t value
## (Intercept)   0.76595    6.15387   0.124
## AGE           0.22354    0.07229   3.092
## EDUCATION    -0.16638    0.17366  -0.958
## group1        4.99961    1.72001   2.907
## month         2.08521    1.12879   1.847
## group1:month  0.85686    1.18793   0.721
##
## Correlation of Fixed Effects:
##             (Intr) AGE    EDUCAT group1 month
## AGE         -0.893
## EDUCATION   -0.438  0.080
## group1      -0.068 -0.167 -0.073
## month        0.042 -0.015  0.005 -0.109
## group1:mnth -0.034  0.010 -0.009  0.119 -0.950
## P Value for Full vs. Reduced Model
## [1] 0.4708178
```

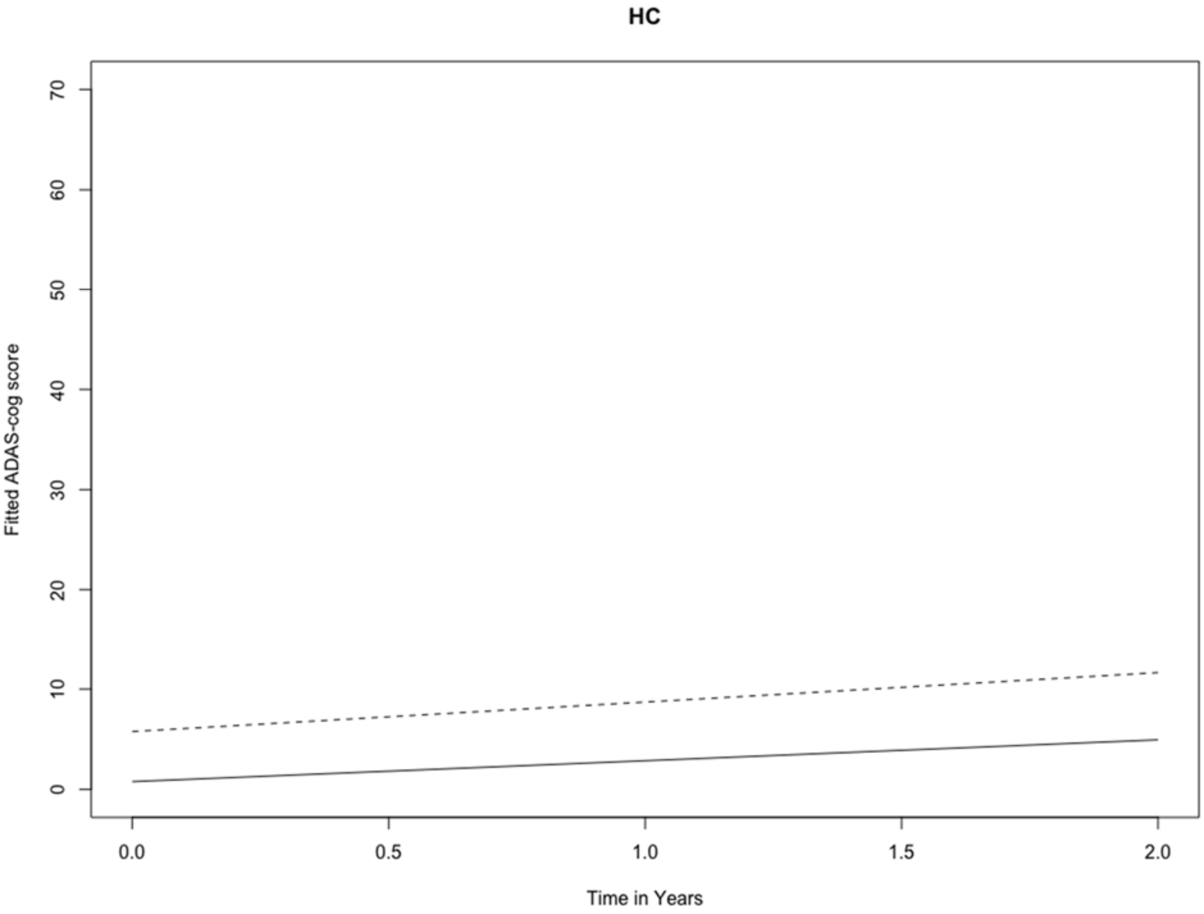

© 2018 Kennedy RE et al. *JAMA Network Open*.

```
## Study Name
## [1] "HU"
## Full Mixed Effects Model
## Linear mixed model fit by maximum likelihood  ['lmerMod']
## Formula: ADAS ~ AGE + EDUCATION + group + month + group:month + (month |
##     UID)
##    Data: mixed.data
##
##      AIC      BIC   logLik deviance df.resid
##   1548.8   1583.2   -764.4   1528.8      220
##
## Scaled residuals:
##      Min       1Q   Median       3Q      Max
## -1.93725 -0.53146  0.01131  0.45825  2.91594
##
## Random effects:
##  Groups   Name        Variance Std.Dev. Corr
##  UID      (Intercept) 77.459   8.801
##           month        5.217   2.284    1.00
##  Residual             14.922   3.863
## Number of obs: 230, groups:  UID, 94
##
## Fixed effects:
##             Estimate Std. Error t value
## (Intercept)  12.14502   10.27778   1.182
## AGE           0.14030    0.12028   1.166
## EDUCATION    -0.04411    0.31938  -0.138
## group1        0.78853    6.80071   0.116
## month         1.66161    0.70692   2.350
## group1:month  1.05060    3.49354   0.301
##
## Correlation of Fixed Effects:
##            (Intr) AGE    EDUCAT group1 month
## AGE        -0.910
## EDUCATION   -0.372 -0.036
## group1      0.025  0.021 -0.145
## month       0.040 -0.033 -0.001 -0.014
## group1:mnth -0.022  0.010  0.025  0.200 -0.203
## P Value for Full vs. Reduced Model
## [1] 0.7645879
```

© 2018 Kennedy RE et al. *JAMA Network Open*.

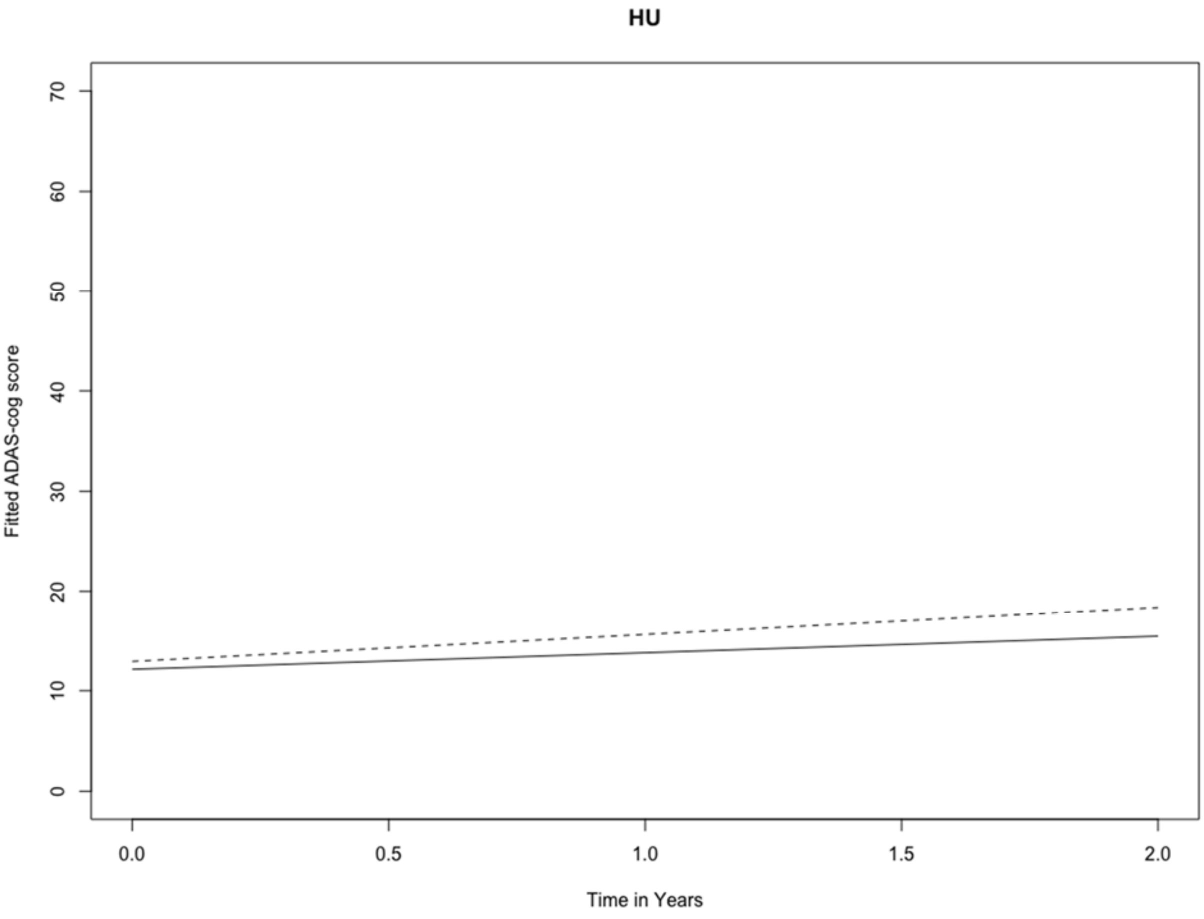


```
## Study Name
## [1] "LL"
## Full Mixed Effects Model
## Linear mixed model fit by maximum likelihood  ['lmerMod']
## Formula: ADAS ~ AGE + EDUCATION + group + month + group:month + (month |
##     UID)
##    Data: mixed.data
##
##      AIC      BIC   logLik deviance df.resid
##   3639.9   3683.3  -1810.0   3619.9      553
##
## Scaled residuals:
##     Min      1Q  Median      3Q     Max
## -2.4868 -0.4675 -0.0148  0.4783  3.2435
##
## Random effects:
##  Groups   Name        Variance Std.Dev. Corr
##  UID      (Intercept) 67.02    8.187
##           month       14.03    3.745    0.23
##  Residual             11.85    3.442
```

© 2018 Kennedy RE et al. *JAMA Network Open*.

```
## Number of obs: 563, groups:  UID, 180
##
## Fixed effects:
##             Estimate Std. Error t value
## (Intercept)  18.18100    7.07638   2.569
## AGE           0.01529    0.07846   0.195
## EDUCATION     0.06772    0.19688   0.344
## group1        0.89764    2.53179   0.355
## month         5.06810    1.68741   3.003
## group1:month -1.20203    1.74570  -0.689
##
## Correlation of Fixed Effects:
##            (Intr) AGE    EDUCAT group1 month
## AGE        -0.846
## EDUCATION  -0.479  0.087
## group1     -0.304 -0.066  0.071
## month      -0.004 -0.003 -0.001  0.017
## group1:mnth  0.003  0.003  0.001 -0.018 -0.967
## P Value for Full vs. Reduced Model
## [1] 0.4930244
```

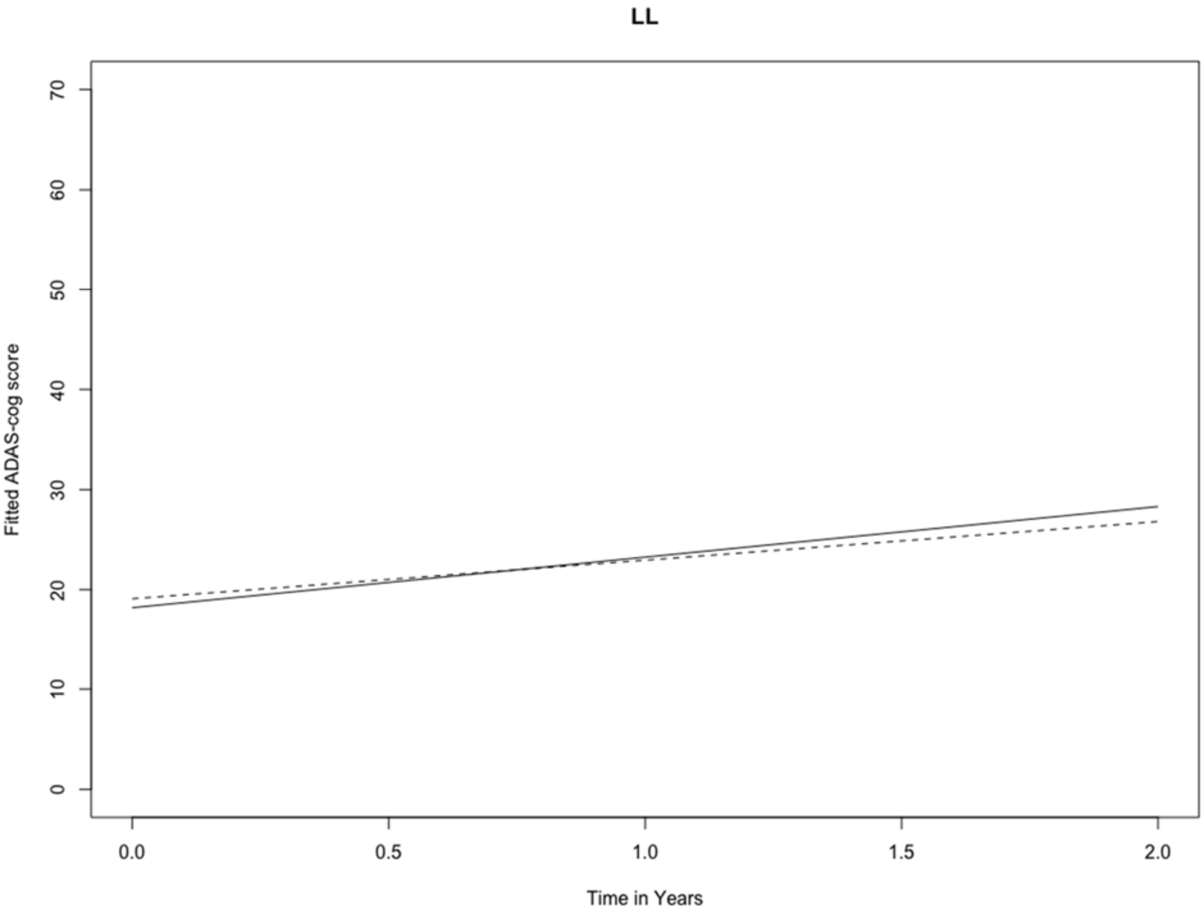

© 2018 Kennedy RE et al. *JAMA Network Open*.

```
## Study Name
## [1] "NS"
## Full Mixed Effects Model
## Linear mixed model fit by maximum likelihood  ['lmerMod']
## Formula: ADAS ~ AGE + EDUCATION + group + month + group:month + (month |
##     UID)
##    Data: mixed.data
##
##      AIC      BIC   logLik deviance df.resid
##   5706.5   5753.8  -2843.2   5686.5      833
##
## Scaled residuals:
##      Min       1Q   Median       3Q      Max
## -2.57400 -0.45745 -0.02595  0.44000  2.61174
##
## Random effects:
##  Groups   Name        Variance Std.Dev. Corr
##  UID      (Intercept) 79.28    8.904
##           month       22.27    4.719    0.48
##  Residual             14.05    3.748
## Number of obs: 843, groups:  UID, 343
##
## Fixed effects:
##              Estimate Std. Error t value
## (Intercept)  31.46520    6.02915   5.219
## AGE          -0.11060    0.06868  -1.610
## EDUCATION    -0.07899    0.15996  -0.494
## group1        1.90681    1.41642   1.346
## month         2.20864    1.17283   1.883
## group1:month  3.60857    1.27024   2.841
##
## Correlation of Fixed Effects:
##             (Intr) AGE    EDUCAT group1 month
## AGE         -0.912
## EDUCATION   -0.458  0.121
## group1      -0.280  0.129 -0.069
## month        0.009  0.004  0.003 -0.058
## group1:mnth -0.006 -0.005 -0.005  0.063 -0.923
## P Value for Full vs. Reduced Model
## [1] 0.004861028
```

© 2018 Kennedy RE et al. *JAMA Network Open*.

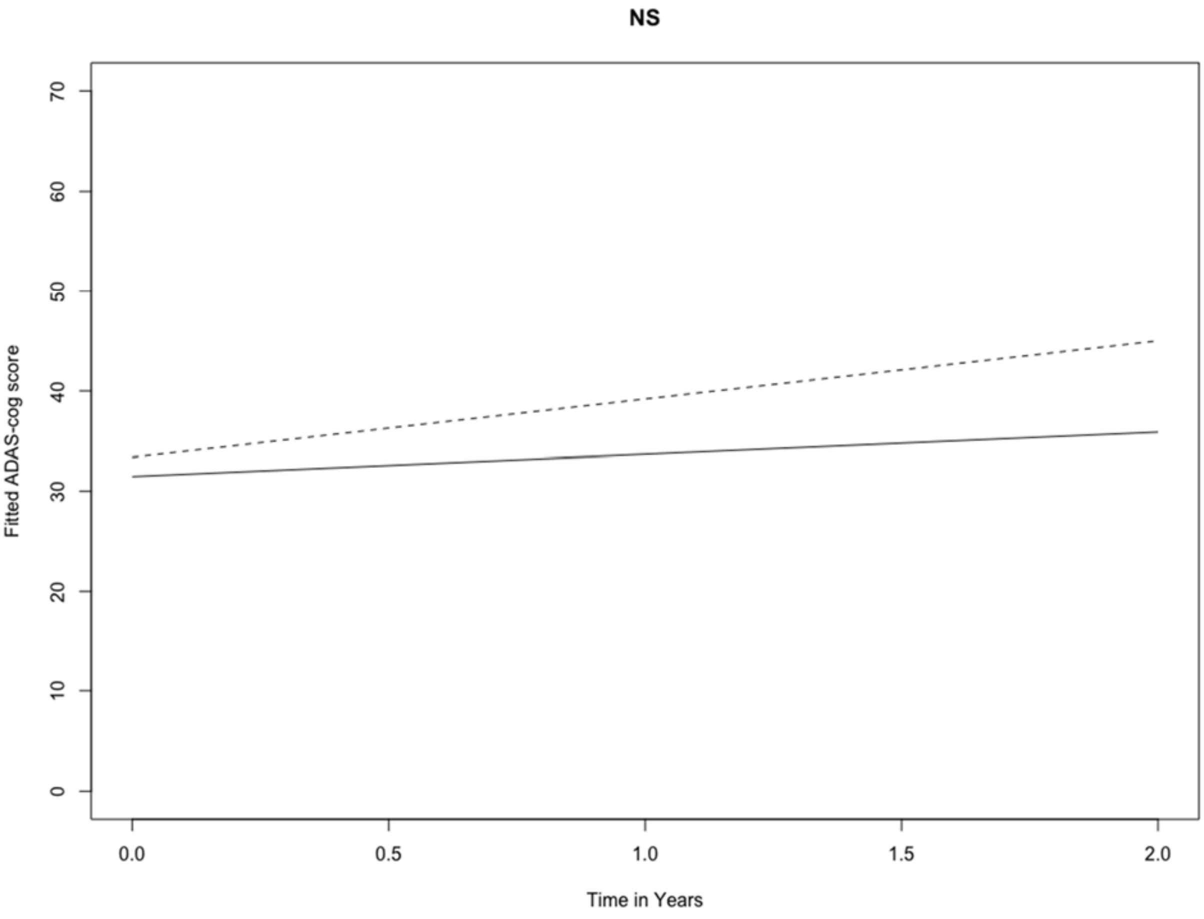


```
## Study Name
## [1] "PR"
## Full Mixed Effects Model
## Linear mixed model fit by maximum likelihood  ['lmerMod']
## Formula: ADAS ~ AGE + EDUCATION + group + month + group:month + (month |
##     UID)
##    Data: mixed.data
##
##      AIC      BIC   logLik deviance df.resid
##   2958.4   2999.2  -1469.2   2938.4      428
##
## Scaled residuals:
##      Min       1Q   Median       3Q      Max
## -2.76980 -0.50680 -0.02234  0.47104  2.93902
##
## Random effects:
##  Groups   Name        Variance Std.Dev. Corr
##  UID      (Intercept) 81.11    9.006
##           month       22.10    4.701    0.76
##  Residual             16.67    4.083
```

© 2018 Kennedy RE et al. *JAMA Network Open*.

```
## Number of obs: 438, groups:  UID, 135
##
## Fixed effects:
##              Estimate Std. Error t value
## (Intercept)  32.78688    9.01135   3.638
## AGE          -0.06482    0.10664  -0.608
## EDUCATION    -0.43416    0.25763  -1.685
## group1       -0.12742    2.39055  -0.053
## month         5.99618    0.63927   9.380
## group1:month -2.16187    1.54065  -1.403
##
## Correlation of Fixed Effects:
##             (Intr) AGE    EDUCAT group1 month
## AGE         -0.911
## EDUCATION   -0.521  0.137
## group1      -0.051 -0.008  0.049
## month        0.053 -0.014 -0.026 -0.117
## group1:mnth -0.032  0.014  0.017  0.356 -0.415
## P Value for Full vs. Reduced Model
## [1] 0.1620023
```

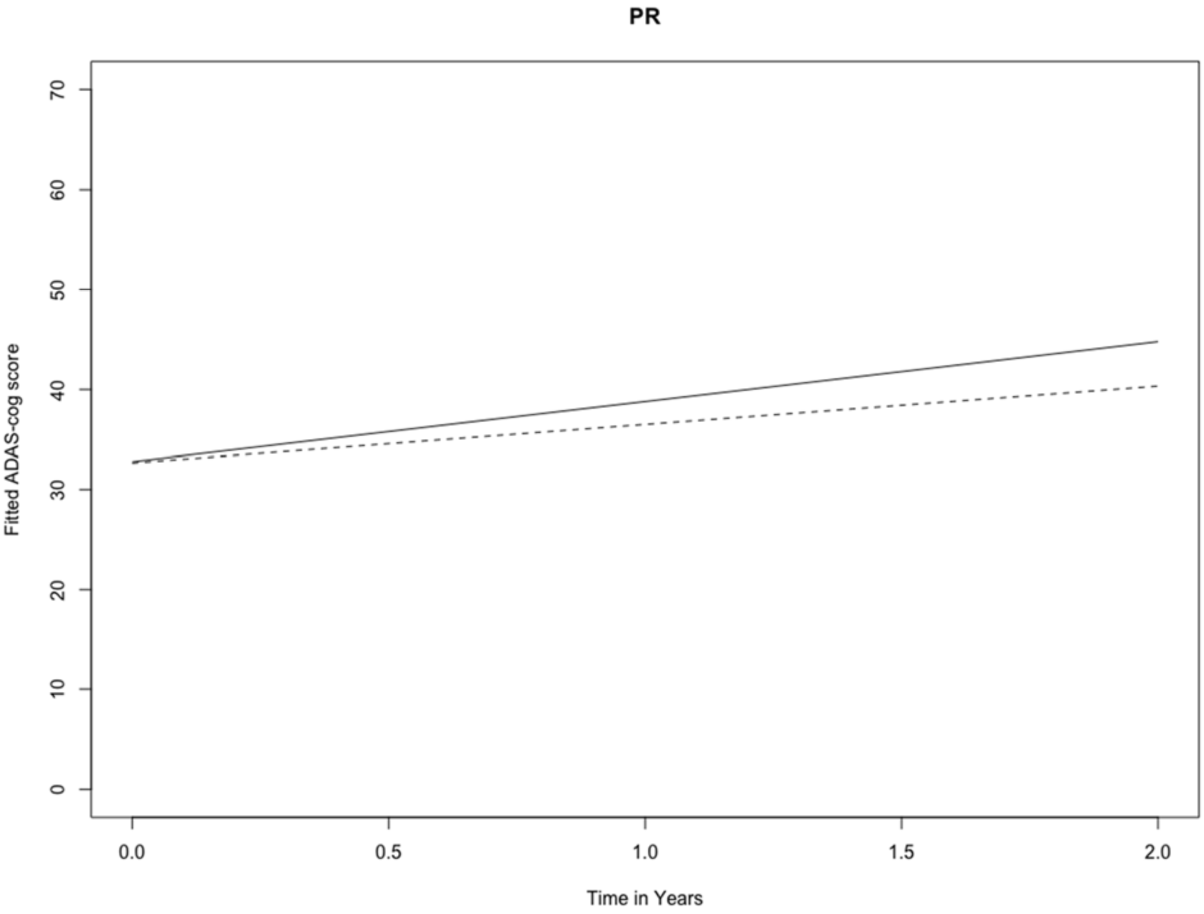

© 2018 Kennedy RE et al. *JAMA Network Open*.

```
## Study Name
## [1] "SL"
## Full Mixed Effects Model
## Linear mixed model fit by maximum likelihood  ['lmerMod']
## Formula: ADAS ~ AGE + EDUCATION + group + month + group:month + (month |
##     UID)
##    Data: mixed.data
##
##      AIC      BIC   logLik deviance df.resid
##   6467.8   6516.5  -3223.9   6447.8      951
##
## Scaled residuals:
##     Min      1Q  Median      3Q     Max
## -2.9713 -0.4627  0.0355  0.4802  3.1977
##
## Random effects:
##  Groups   Name        Variance Std.Dev. Corr
##  UID      (Intercept) 105.53   10.273
##           month        14.80    3.847   0.34
##  Residual              14.68    3.831
## Number of obs: 961, groups:  UID, 316
##
## Fixed effects:
##               Estimate Std. Error t value
## (Intercept)   51.372173   6.398754   8.028
## AGE           -0.172115   0.076926  -2.237
## EDUCATION      0.006988   0.187404   0.037
## group1       -12.686658   7.818356  -1.623
## month          7.769544   0.356626  21.786
## group1:month   3.711581   3.751239   0.989
##
## Correlation of Fixed Effects:
##             (Intr) AGE    EDUCAT group1 month
## AGE         -0.929
## EDUCATION   -0.488  0.145
## group1      -0.015  0.087 -0.191
## month        0.002  0.001  0.010 -0.007
## group1:mnth  0.001  0.000 -0.003  0.086 -0.095
## P Value for Full vs. Reduced Model
## [1] 0.3234868
```

© 2018 Kennedy RE et al. *JAMA Network Open*.

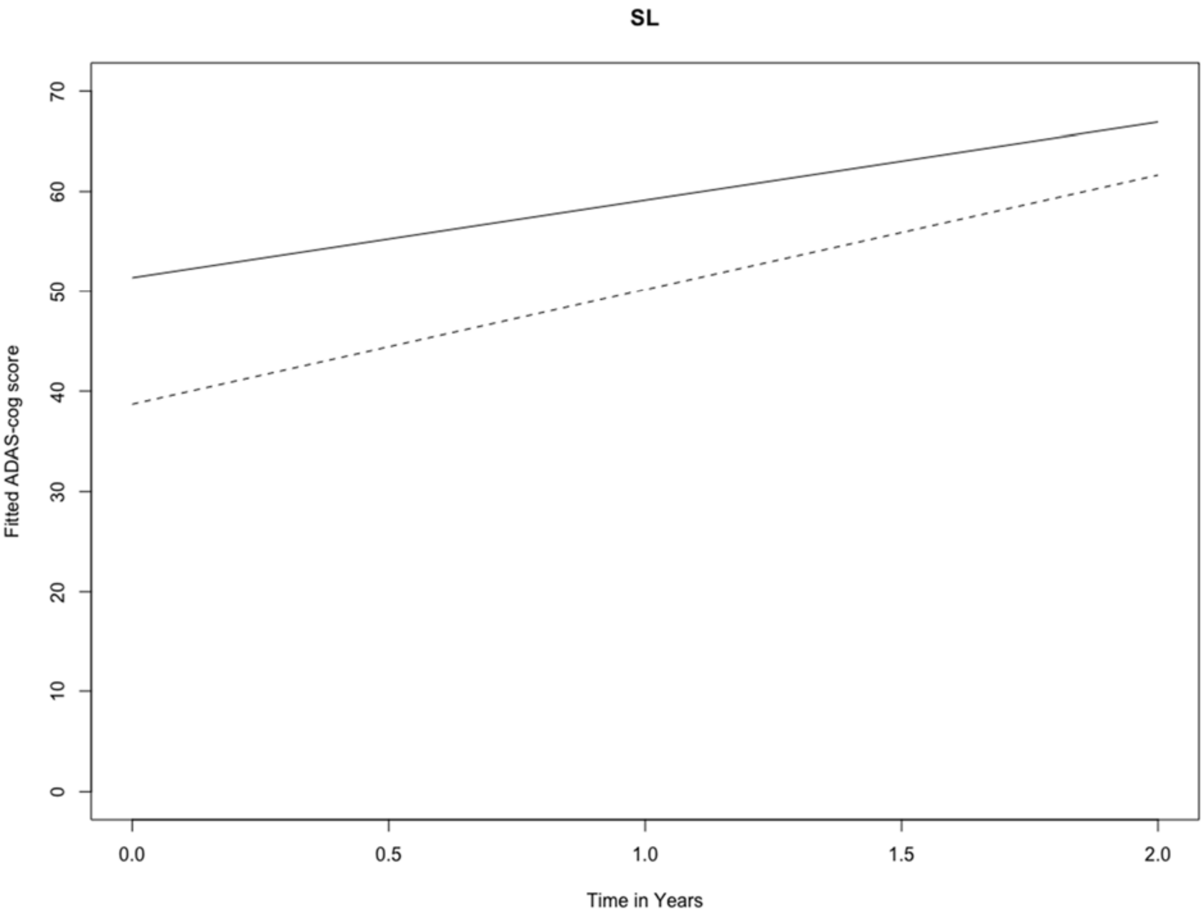


```
## Study Name
## [1] "VN"
## Full Mixed Effects Model
## Linear mixed model fit by maximum likelihood  ['lmerMod']
## Formula: ADAS ~ AGE + EDUCATION + group + month + group:month + (month |
##     UID)
##    Data: mixed.data
##
##      AIC      BIC   logLik deviance df.resid
##   1588.4   1623.1   -784.2   1568.4      227
##
## Scaled residuals:
##     Min      1Q  Median      3Q     Max
## -3.1131 -0.4569 -0.0712  0.4505  3.3913
##
## Random effects:
##  Groups   Name        Variance Std.Dev. Corr
##  UID      (Intercept) 58.55    7.652
##           month       26.75    5.172    0.39
##  Residual             15.17    3.895
```

© 2018 Kennedy RE et al. *JAMA Network Open*.

```
## Number of obs: 237, groups:  UID, 73
##
## Fixed effects:
##              Estimate Std. Error t value
## (Intercept)  23.36473   13.52038   1.728
## AGE           0.08565    0.15289   0.560
## EDUCATION     0.03185    0.26852   0.119
## group1       -3.97281    3.62194  -1.097
## month         7.02839    2.97828   2.360
## group1:month -1.57634    3.11021  -0.507
##
## Correlation of Fixed Effects:
##             (Intr) AGE    EDUCAT group1 month
## AGE         -0.920
## EDUCATION   -0.379  0.086
## group1      -0.348  0.063  0.180
## month        0.042 -0.021  0.001 -0.091
## group1:mnth -0.042  0.022 -0.003  0.096 -0.958
## P Value for Full vs. Reduced Model
## [1] 0.6131325
```

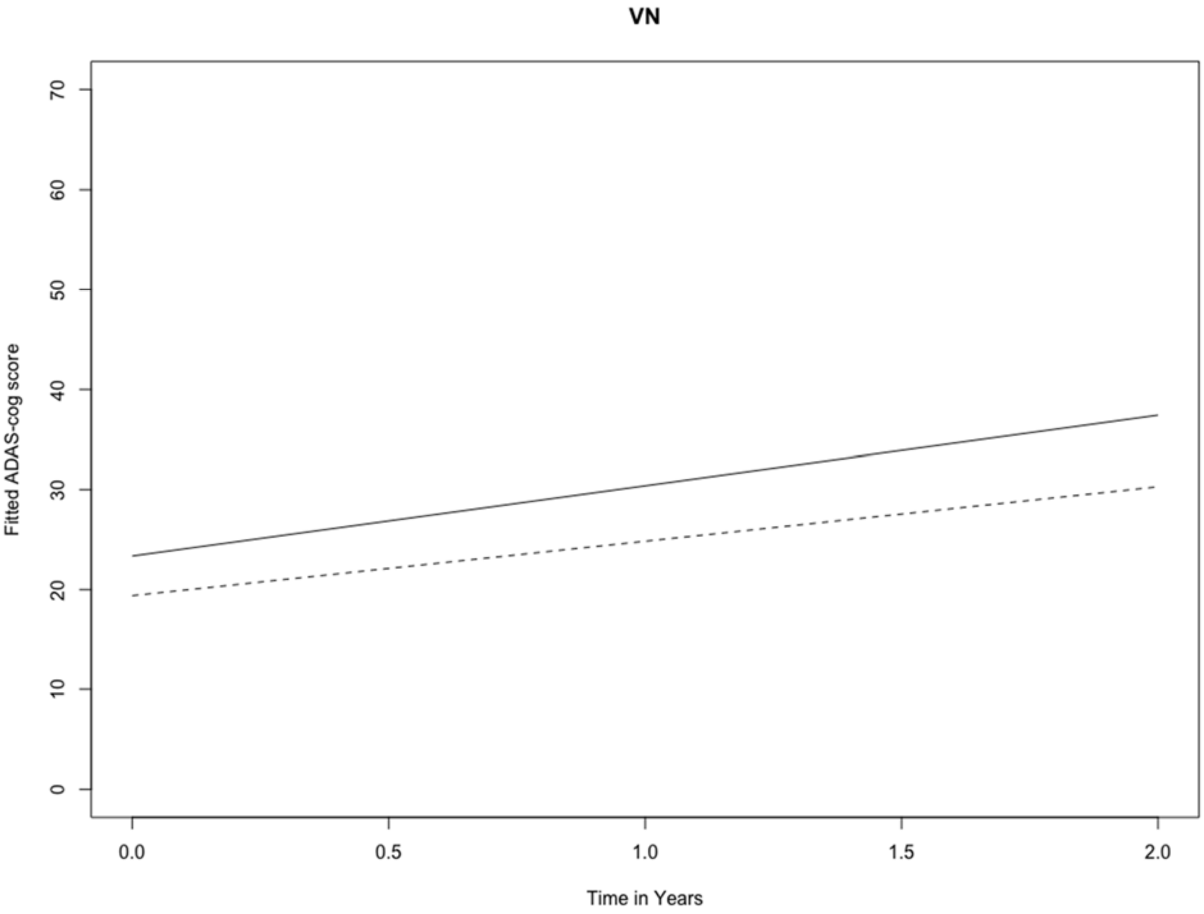

© 2018 Kennedy RE et al. *JAMA Network Open*.

Meta-Analysis with metafor

```
##
## Mixed-Effects Model (k = 10; tau^2 estimator: REML)
##
## tau^2 (estimated amount of residual heterogeneity):     2.1276 (SE = 2.5881)
## tau (square root of estimated tau^2 value):             1.4586
## I^2 (residual heterogeneity / unaccounted variability): 42.00%
## H^2 (unaccounted variability / sampling variability):   1.72
## R^2 (amount of heterogeneity accounted for):            0.00%
##
## Test for Residual Heterogeneity:
## QE(df = 8) = 12.3357, p-val = 0.1368
##
## Test of Moderators (coefficient(s) 2):
## QM(df = 1) = 1.2402, p-val = 0.2654
##
## Model Results:
##
##          estimate      se     zval    pval    ci.lb   ci.ub
## intrcpt    2.4624  1.5951   1.5438  0.1226  -0.6638  5.5887
## mods      -0.1757  0.1577  -1.1136  0.2654  -0.4848  0.1335
##
## ---
## Signif. codes:  0 '***' 0.001 '**' 0.01 '*' 0.05 '.' 0.1 ' ' 1
```

© 2018 Kennedy RE et al. *JAMA Network Open*.

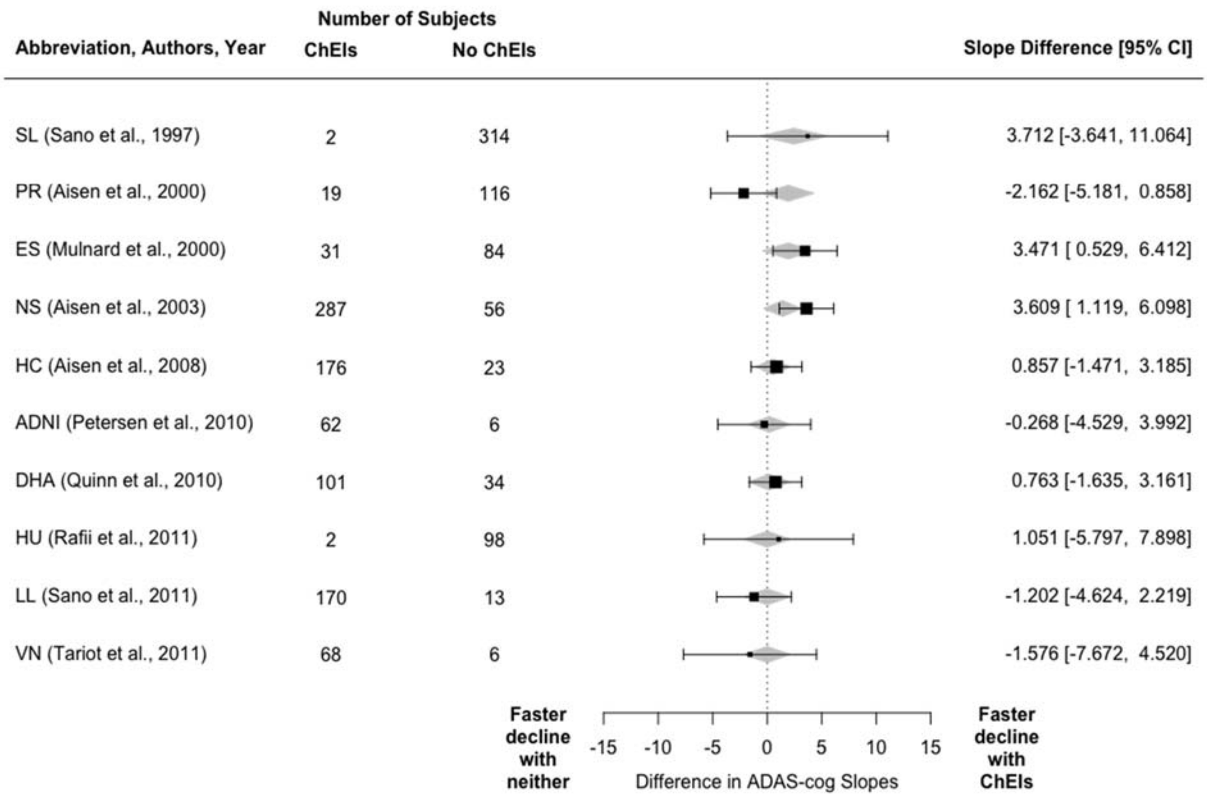

© 2018 Kennedy RE et al. *JAMA Network Open*.

Rates of decline for ChEIs vs. neither, with moderator of % taking meds

Mixed Models

```
## Study Name
## [1] "ADNI"
## Full Mixed Effects Model
## Linear mixed model fit by maximum likelihood  ['lmerMod']
## Formula: ADAS ~ AGE + EDUCATION + group + month + group:month + (month |
##     UID)
##    Data: mixed.data
##
##      AIC      BIC   logLik deviance df.resid
##   1355.7   1389.5   -667.8   1335.7      207
##
## Scaled residuals:
##      Min       1Q   Median       3Q      Max
## -2.23216 -0.39292 -0.02574  0.36394  2.53793
##
## Random effects:
##  Groups   Name        Variance Std.Dev. Corr
##  UID      (Intercept) 30.229   5.498
##           month       14.023   3.745    0.49
##  Residual              9.804   3.131
## Number of obs: 217, groups:  UID, 65
##
## Fixed effects:
##             Estimate Std. Error t value
## (Intercept)  11.77585   10.17250   1.158
## AGE           0.05206    0.12220   0.426
## EDUCATION     0.16585    0.21942   0.756
## group1       -0.41759    2.63963  -0.158
## month         4.96049    2.07887   2.386
## group1:month -0.26826    2.17385  -0.123
##
## Correlation of Fixed Effects:
##             (Intr) AGE    EDUCAT group1 month
## AGE         -0.933
## EDUCATION   -0.276  0.015
## group1      -0.204  0.017 -0.158
## month        0.016  0.013  0.063 -0.182
## group1:mnth -0.020 -0.008 -0.061  0.190 -0.956
## P Value for Full vs. Reduced Model
## [1] 0.9019588
```

© 2018 Kennedy RE et al. *JAMA Network Open*.

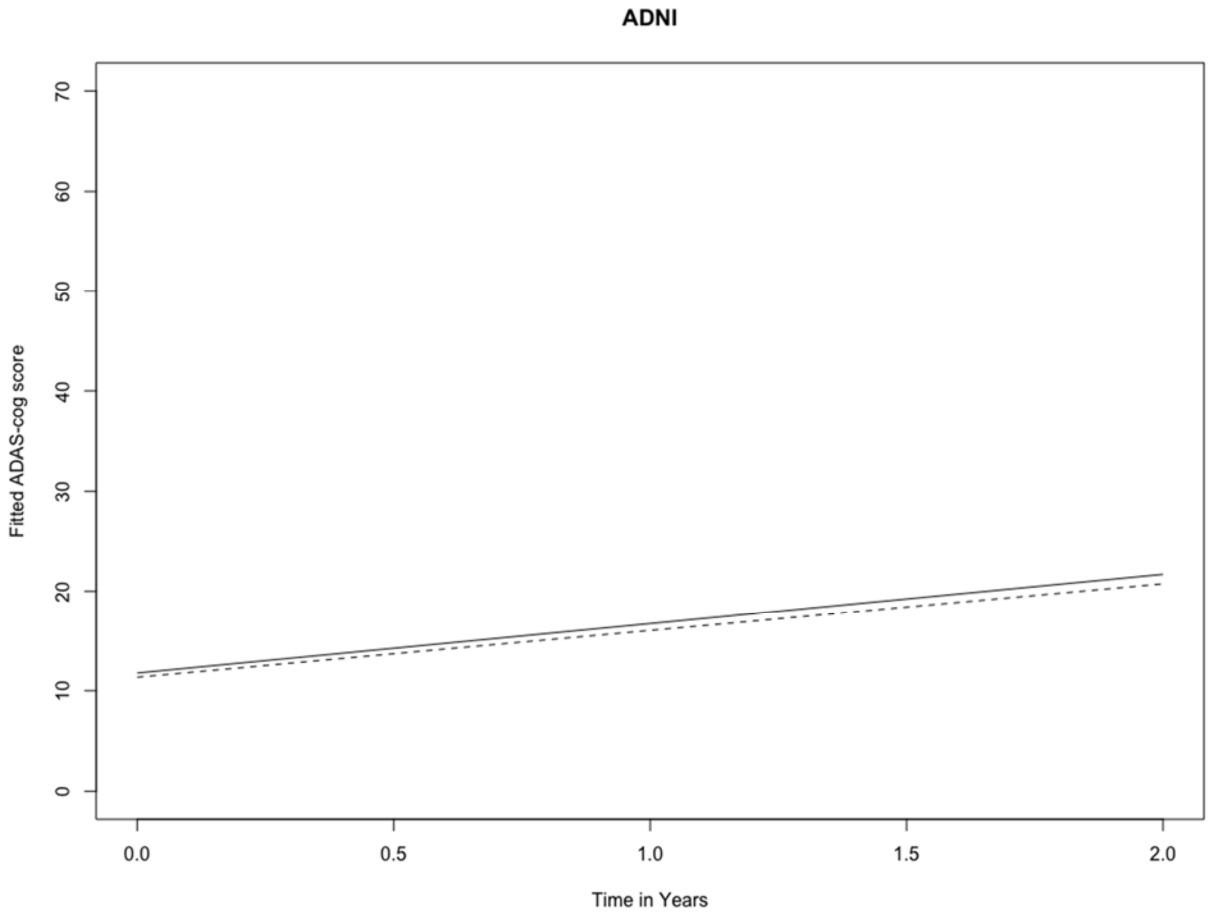


```
## Study Name
## [1] "DHA"
## Full Mixed Effects Model
## Linear mixed model fit by maximum likelihood  ['lmerMod']
## Formula: ADAS ~ AGE + EDUCATION + group + month + group:month + (month |
##     UID)
##    Data: mixed.data
##
##      AIC      BIC   logLik deviance df.resid
##   2872.3   2913.5  -1426.1   2852.3      445
##
## Scaled residuals:
##      Min       1Q   Median       3Q      Max
## -2.47423 -0.47238 -0.05433  0.44697  2.82137
##
## Random effects:
##  Groups   Name        Variance Std.Dev. Corr
##  UID      (Intercept) 39.49    6.284
##           month       20.82    4.563    0.64
##  Residual             11.14    3.338
```

© 2018 Kennedy RE et al. *JAMA Network Open*.

```
## Number of obs: 455, groups:  UID, 134
##
## Fixed effects:
##              Estimate Std. Error t value
## (Intercept)   5.91901    6.30850   0.938
## AGE           0.23081    0.06911   3.340
## EDUCATION    -0.46288    0.20682  -2.238
## group1        3.58044    1.39511   2.566
## month         3.20186    1.06703   3.001
## group1:month  0.76304    1.22349   0.624
##
## Correlation of Fixed Effects:
##             (Intr) AGE    EDUCAT group1 month
## AGE         -0.862
## EDUCATION   -0.476  0.008
## group1      -0.268  0.089  0.057
## month        0.055 -0.009  0.008 -0.230
## group1:mnth -0.040 -0.001 -0.006  0.264 -0.872
## P Value for Full vs. Reduced Model
## [1] 0.5351913
```

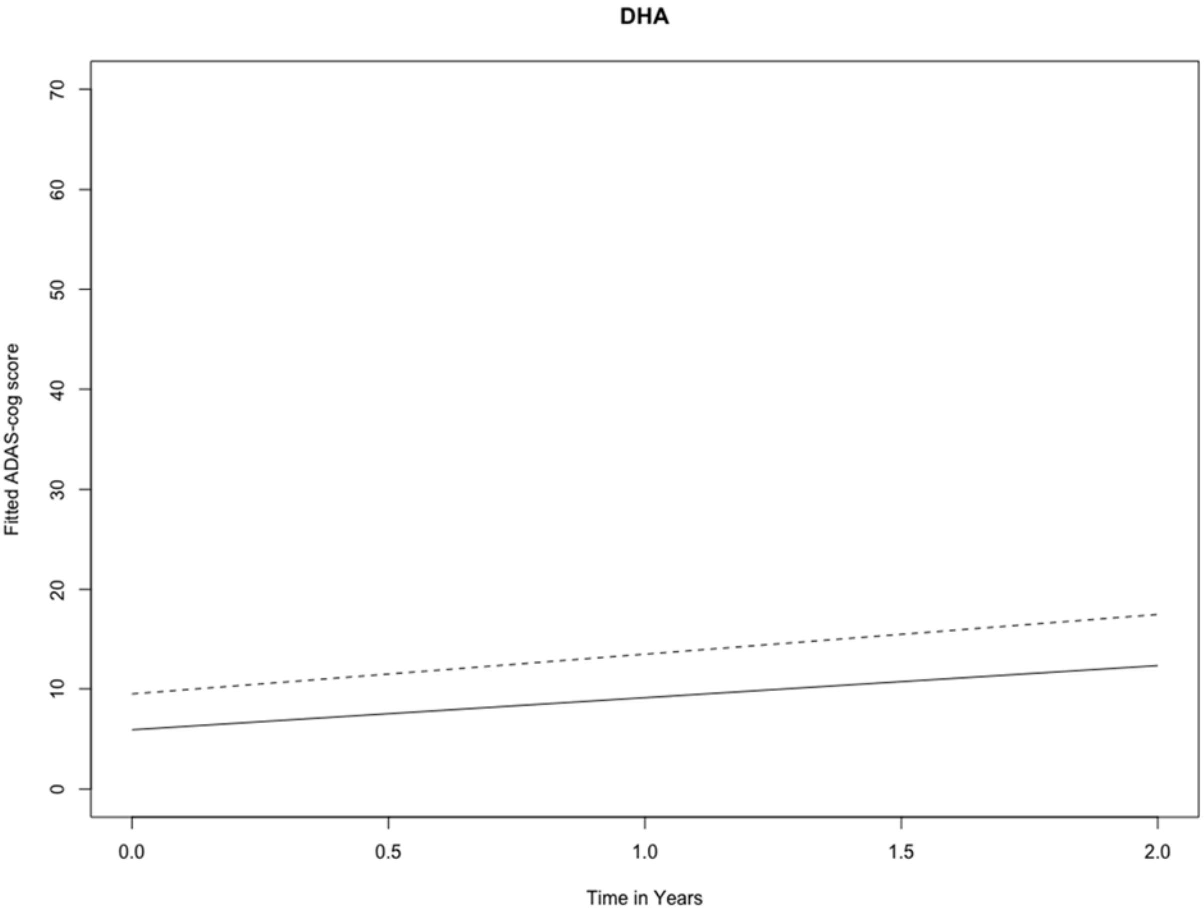

© 2018 Kennedy RE et al. *JAMA Network Open*.

```
## Study Name
## [1] "ES"
## Full Mixed Effects Model
## Linear mixed model fit by maximum likelihood  ['lmerMod']
## Formula: ADAS ~ AGE + EDUCATION + group + month + group:month + (month |
##     UID)
##    Data: mixed.data
##
##      AIC      BIC   logLik deviance df.resid
##   2004.6   2041.7   -992.3   1984.6      292
##
## Scaled residuals:
##      Min       1Q   Median       3Q      Max
## -2.47336 -0.52156 -0.01569  0.48441  2.62754
##
## Random effects:
##  Groups   Name        Variance Std.Dev. Corr
##  UID      (Intercept) 62.40    7.899
##           month       15.68    3.959    0.87
##  Residual             13.66    3.696
## Number of obs: 302, groups:  UID, 115
##
## Fixed effects:
##             Estimate Std. Error t value
## (Intercept)   14.9766     9.8512   1.520
## AGE            0.1429     0.1177   1.215
## EDUCATION     -0.1100     0.2496  -0.441
## group1        -0.1170     1.8339  -0.064
## month          4.8566     0.7858   6.181
## group1:month   3.4706     1.5006   2.313
##
## Correlation of Fixed Effects:
##            (Intr) AGE    EDUCAT group1 month
## AGE        -0.950
## EDUCATION   -0.442  0.155
## group1      -0.061  0.059 -0.135
## month        0.023 -0.002 -0.007 -0.106
## group1:mnth -0.018  0.004  0.012  0.208 -0.524
## P Value for Full vs. Reduced Model
## [1] 0.02347949
```

© 2018 Kennedy RE et al. *JAMA Network Open*.

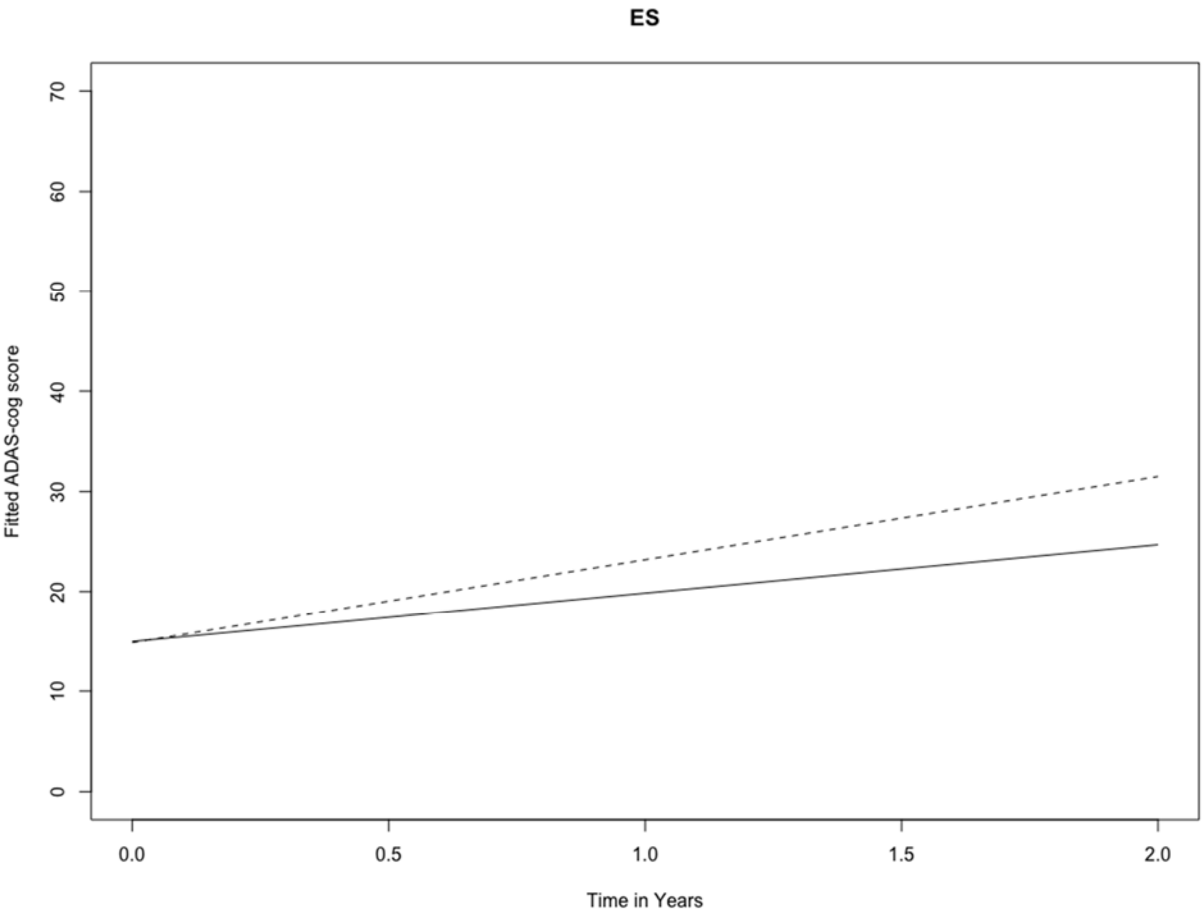


```
## Study Name
## [1] "HC"
## Full Mixed Effects Model
## Linear mixed model fit by maximum likelihood  ['lmerMod']
## Formula: ADAS ~ AGE + EDUCATION + group + month + group:month + (month |
##     UID)
##    Data: mixed.data
##
##      AIC      BIC   logLik deviance df.resid
##   4191.7   4236.8  -2085.9   4171.7      658
##
## Scaled residuals:
##     Min      1Q  Median      3Q     Max
## -3.2083 -0.5395 -0.0379  0.4835  2.7823
##
## Random effects:
##  Groups   Name        Variance Std.Dev. Corr
##  UID      (Intercept) 46.99    6.855
##           month       10.27    3.205    0.56
##  Residual             11.58    3.403
```

© 2018 Kennedy RE et al. *JAMA Network Open*.

```
## Number of obs: 668, groups:  UID, 193
##
## Fixed effects:
##               Estimate Std. Error t value
## (Intercept)   0.76595    6.15387   0.124
## AGE           0.22354    0.07229   3.092
## EDUCATION    -0.16638    0.17366  -0.958
## group1        4.99961    1.72001   2.907
## month         2.08521    1.12879   1.847
## group1:month  0.85686    1.18793   0.721
##
## Correlation of Fixed Effects:
##              (Intr) AGE    EDUCAT group1 month
## AGE          -0.893
## EDUCATION    -0.438  0.080
## group1       -0.068 -0.167 -0.073
## month         0.042 -0.015  0.005 -0.109
## group1:mnth -0.034  0.010 -0.009  0.119 -0.950
## P Value for Full vs. Reduced Model
## [1] 0.4708178
```

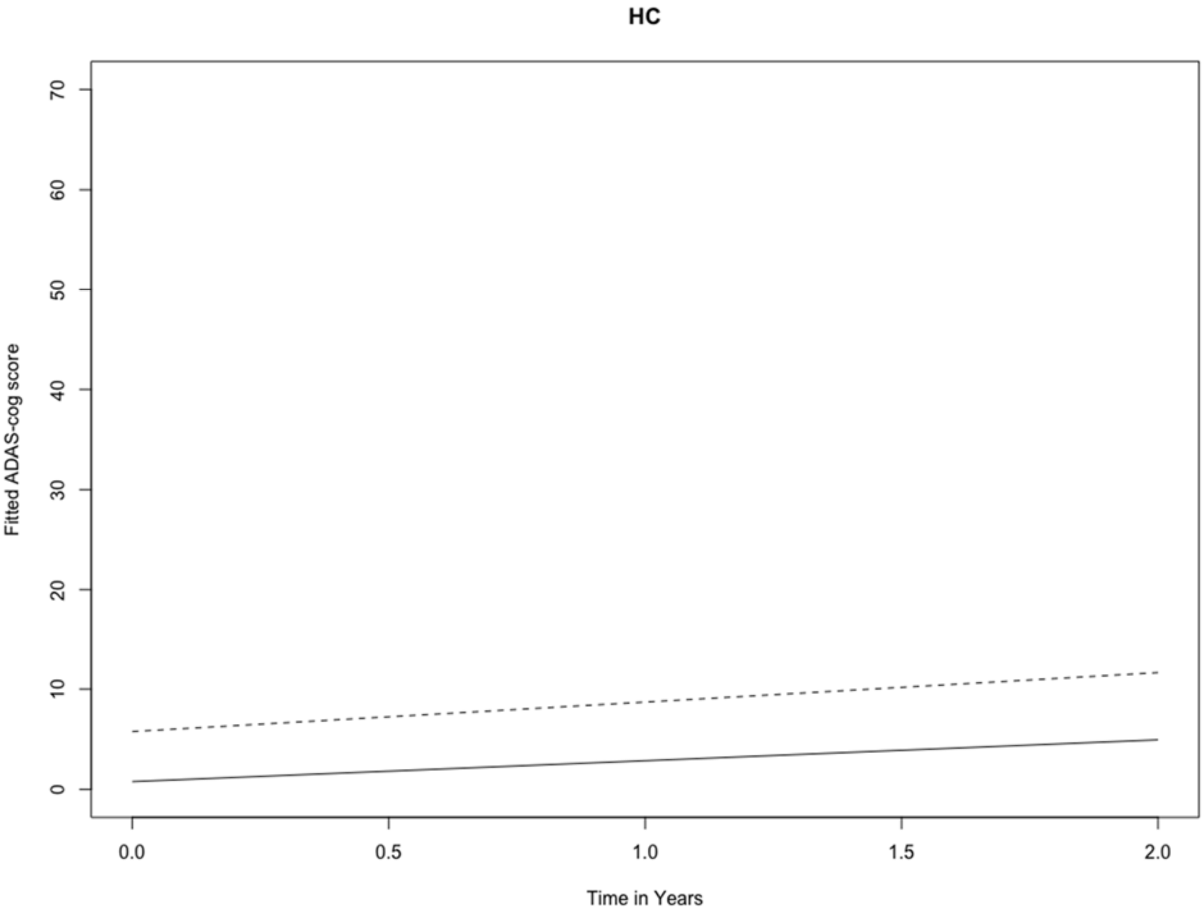

© 2018 Kennedy RE et al. *JAMA Network Open*.

```
## Study Name
## [1] "HU"
## Full Mixed Effects Model
## Linear mixed model fit by maximum likelihood  ['lmerMod']
## Formula: ADAS ~ AGE + EDUCATION + group + month + group:month + (month |
##     UID)
##    Data: mixed.data
##
##      AIC      BIC   logLik deviance df.resid
##   1548.8   1583.2   -764.4   1528.8      220
##
## Scaled residuals:
##      Min       1Q   Median       3Q      Max
## -1.93725 -0.53146  0.01131  0.45825  2.91594
##
## Random effects:
##  Groups   Name        Variance Std.Dev. Corr
##  UID      (Intercept) 77.459   8.801
##           month        5.217   2.284    1.00
##  Residual             14.922   3.863
## Number of obs: 230, groups:  UID, 94
##
## Fixed effects:
##             Estimate Std. Error t value
## (Intercept)  12.14502   10.27778   1.182
## AGE           0.14030    0.12028   1.166
## EDUCATION    -0.04411    0.31938  -0.138
## group1        0.78853    6.80071   0.116
## month         1.66161    0.70692   2.350
## group1:month  1.05060    3.49354   0.301
##
## Correlation of Fixed Effects:
##            (Intr) AGE    EDUCAT group1 month
## AGE        -0.910
## EDUCATION  -0.372 -0.036
## group1      0.025  0.021 -0.145
## month       0.040 -0.033 -0.001 -0.014
## group1:mnth -0.022  0.010  0.025  0.200 -0.203
## P Value for Full vs. Reduced Model
## [1] 0.7645879
```

© 2018 Kennedy RE et al. *JAMA Network Open*.

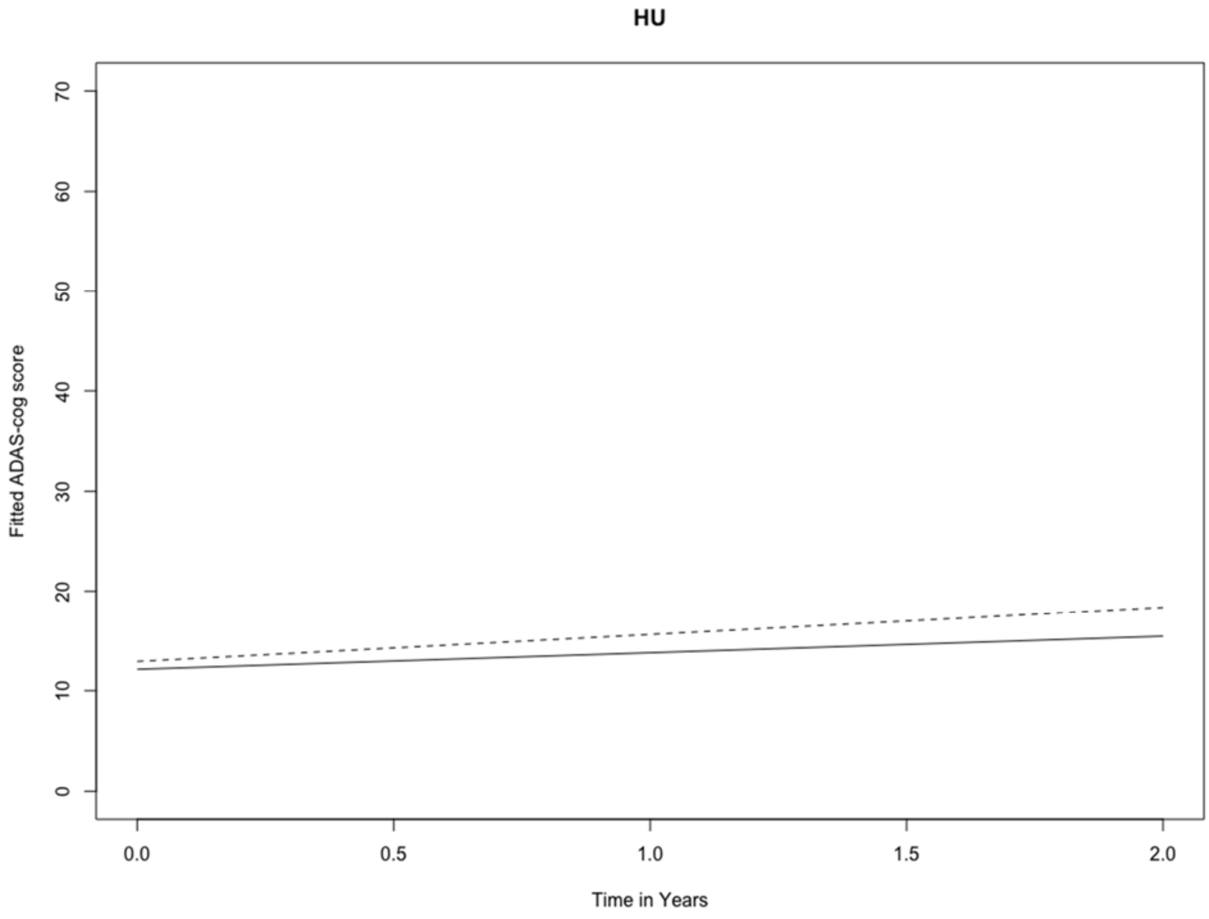


```
## Study Name
## [1] "LL"
## Full Mixed Effects Model
## Linear mixed model fit by maximum likelihood  ['lmerMod']
## Formula: ADAS ~ AGE + EDUCATION + group + month + group:month + (month |
##     UID)
##    Data: mixed.data
##
##      AIC      BIC   logLik deviance df.resid
##   3639.9   3683.3  -1810.0   3619.9      553
##
## Scaled residuals:
##     Min      1Q  Median      3Q     Max
## -2.4868 -0.4675 -0.0148  0.4783  3.2435
##
## Random effects:
##  Groups   Name        Variance Std.Dev. Corr
##  UID      (Intercept) 67.02    8.187
##           month       14.03    3.745    0.23
##  Residual             11.85    3.442
```

© 2018 Kennedy RE et al. *JAMA Network Open*.

```
## Number of obs: 563, groups:  UID, 180
##
## Fixed effects:
##              Estimate Std. Error t value
## (Intercept)  18.18100    7.07638   2.569
## AGE           0.01529    0.07846   0.195
## EDUCATION     0.06772    0.19688   0.344
## group1        0.89764    2.53179   0.355
## month         5.06810    1.68741   3.003
## group1:month -1.20203    1.74570  -0.689
##
## Correlation of Fixed Effects:
##             (Intr) AGE    EDUCAT group1 month
## AGE         -0.846
## EDUCATION   -0.479  0.087
## group1      -0.304 -0.066  0.071
## month       -0.004 -0.003 -0.001  0.017
## group1:mnth  0.003  0.003  0.001 -0.018 -0.967
## P Value for Full vs. Reduced Model
## [1] 0.4930244
```

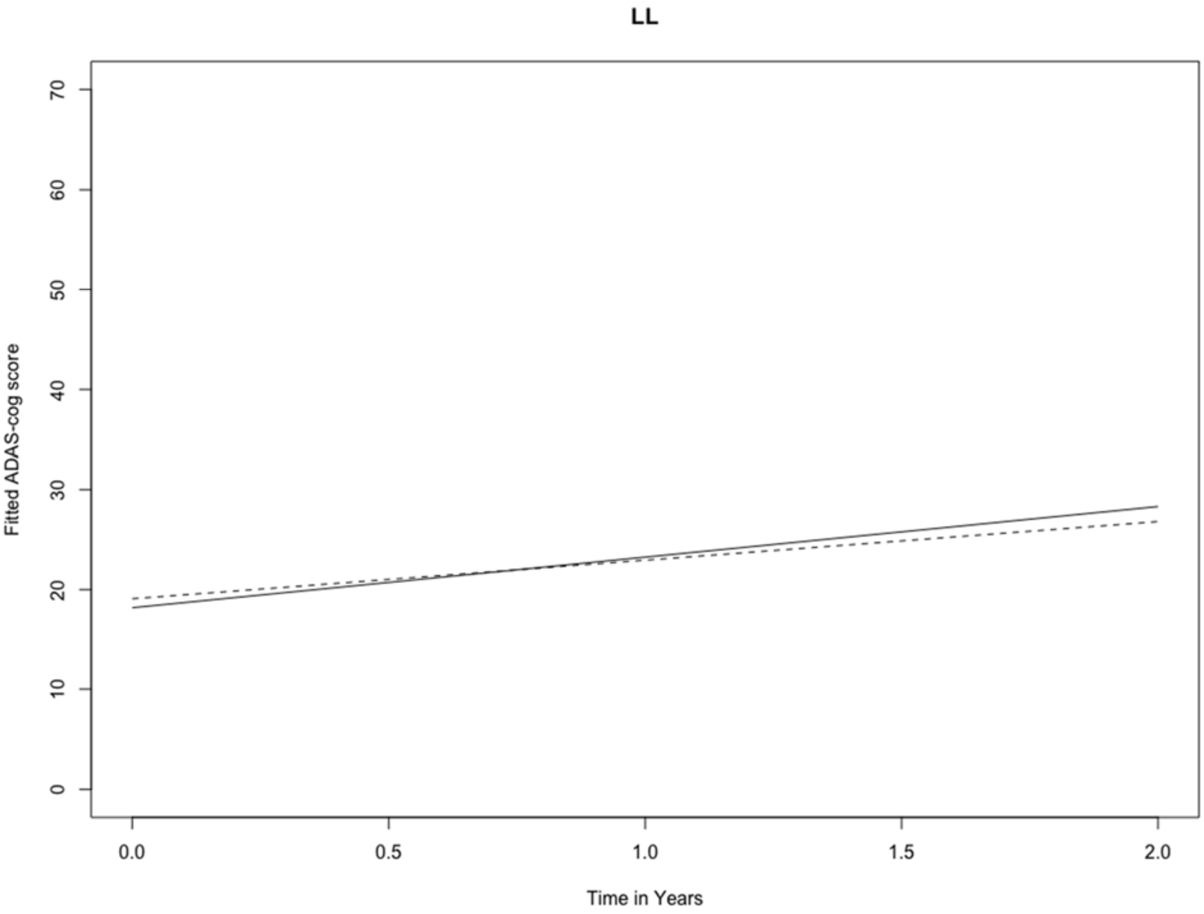

© 2018 Kennedy RE et al. *JAMA Network Open*.

```
## Study Name
## [1] "NS"
## Full Mixed Effects Model
## Linear mixed model fit by maximum likelihood  ['lmerMod']
## Formula: ADAS ~ AGE + EDUCATION + group + month + group:month + (month |
##     UID)
##    Data: mixed.data
##
##      AIC      BIC   logLik deviance df.resid
##   5706.5   5753.8  -2843.2   5686.5      833
##
## Scaled residuals:
##      Min       1Q   Median       3Q      Max
## -2.57400 -0.45745 -0.02595  0.44000  2.61174
##
## Random effects:
##  Groups   Name        Variance Std.Dev. Corr
##  UID      (Intercept) 79.28    8.904
##           month       22.27    4.719    0.48
##  Residual             14.05    3.748
## Number of obs: 843, groups:  UID, 343
##
## Fixed effects:
##             Estimate Std. Error t value
## (Intercept)  31.46520    6.02915   5.219
## AGE          -0.11060    0.06868  -1.610
## EDUCATION    -0.07899    0.15996  -0.494
## group1        1.90681    1.41642   1.346
## month         2.20864    1.17283   1.883
## group1:month  3.60857    1.27024   2.841
##
## Correlation of Fixed Effects:
##             (Intr) AGE    EDUCAT group1 month
## AGE         -0.912
## EDUCATION   -0.458  0.121
## group1      -0.280  0.129 -0.069
## month        0.009  0.004  0.003 -0.058
## group1:mnth -0.006 -0.005 -0.005  0.063 -0.923
## P Value for Full vs. Reduced Model
## [1] 0.004861028
```

© 2018 Kennedy RE et al. *JAMA Network Open*.

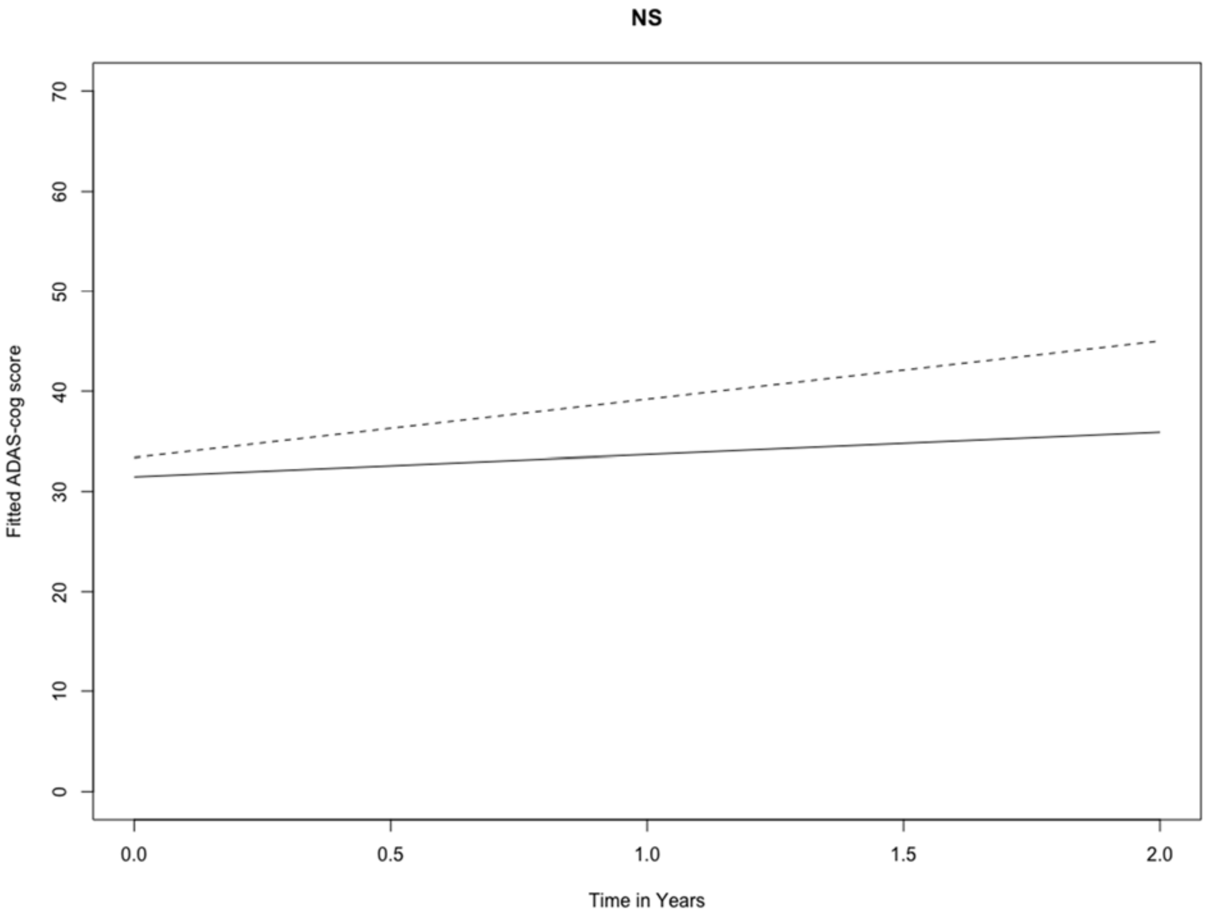


```
## Study Name
## [1] "PR"
## Full Mixed Effects Model
## Linear mixed model fit by maximum likelihood  ['lmerMod']
## Formula: ADAS ~ AGE + EDUCATION + group + month + group:month + (month |
##     UID)
##    Data: mixed.data
##
##      AIC      BIC   logLik deviance df.resid
##   2958.4   2999.2  -1469.2   2938.4      428
##
## Scaled residuals:
##      Min       1Q   Median       3Q      Max
## -2.76980 -0.50680 -0.02234  0.47104  2.93902
##
## Random effects:
##  Groups   Name        Variance Std.Dev. Corr
##  UID      (Intercept) 81.11    9.006
##           month       22.10    4.701    0.76
##  Residual             16.67    4.083
```

© 2018 Kennedy RE et al. *JAMA Network Open*.

```
## Number of obs: 438, groups:  UID, 135
##
## Fixed effects:
##              Estimate Std. Error t value
## (Intercept)  32.78688    9.01135   3.638
## AGE          -0.06482    0.10664  -0.608
## EDUCATION    -0.43416    0.25763  -1.685
## group1       -0.12742    2.39055  -0.053
## month         5.99618    0.63927   9.380
## group1:month -2.16187    1.54065  -1.403
##
## Correlation of Fixed Effects:
##             (Intr) AGE    EDUCAT group1 month
## AGE         -0.911
## EDUCATION   -0.521  0.137
## group1      -0.051 -0.008  0.049
## month        0.053 -0.014 -0.026 -0.117
## group1:mnth -0.032  0.014  0.017  0.356 -0.415
## P Value for Full vs. Reduced Model
## [1] 0.1620023
```

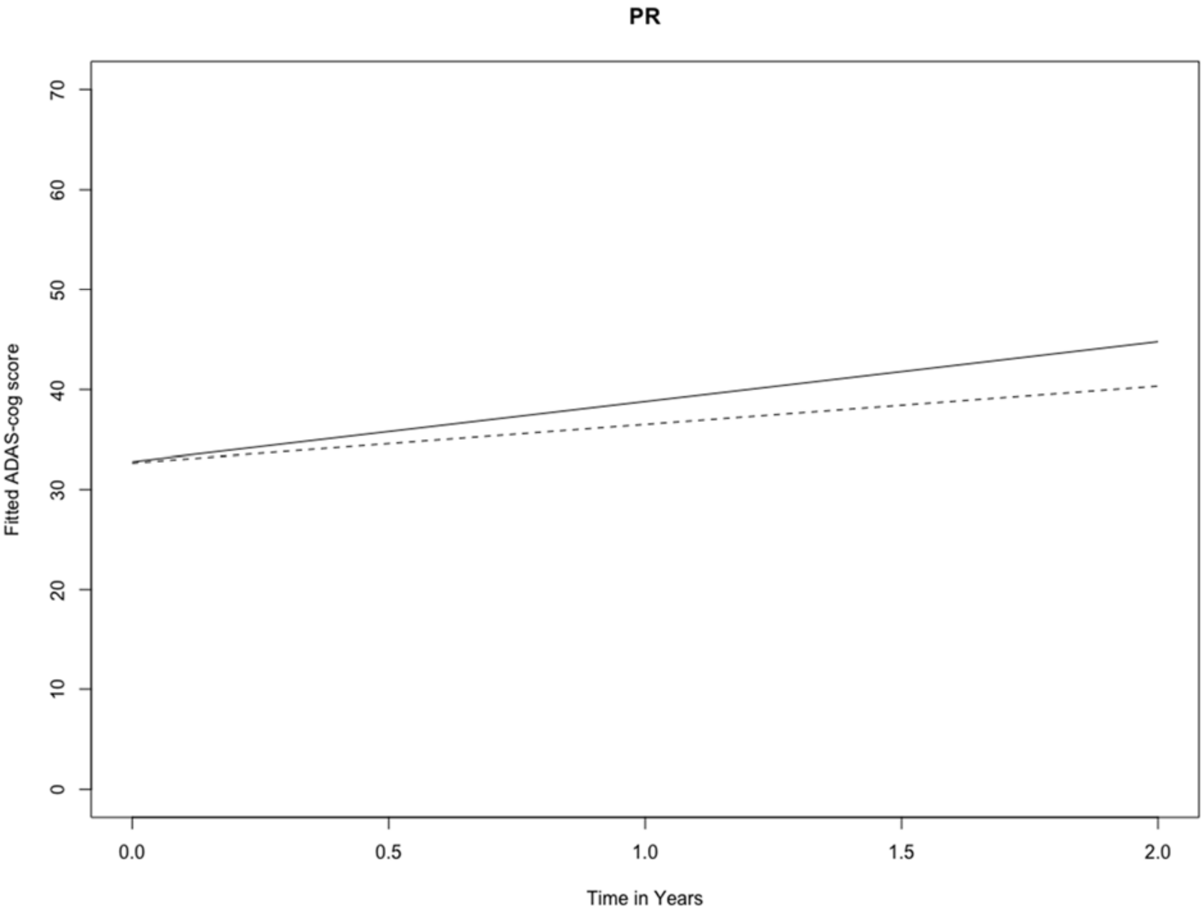

© 2018 Kennedy RE et al. *JAMA Network Open*.

```
## Study Name
## [1] "SL"
## Full Mixed Effects Model
## Linear mixed model fit by maximum likelihood  ['lmerMod']
## Formula: ADAS ~ AGE + EDUCATION + group + month + group:month + (month |
##     UID)
##    Data: mixed.data
##
##      AIC      BIC   logLik deviance df.resid
##   6467.8   6516.5  -3223.9   6447.8      951
##
## Scaled residuals:
##     Min      1Q  Median      3Q     Max
## -2.9713 -0.4627  0.0355  0.4802  3.1977
##
## Random effects:
##  Groups   Name        Variance Std.Dev. Corr
##  UID      (Intercept) 105.53   10.273
##           month        14.80    3.847   0.34
##  Residual              14.68    3.831
## Number of obs: 961, groups:  UID, 316
##
## Fixed effects:
##               Estimate Std. Error t value
## (Intercept)   51.372173   6.398754   8.028
## AGE           -0.172115   0.076926  -2.237
## EDUCATION      0.006988   0.187404   0.037
## group1       -12.686658   7.818356  -1.623
## month          7.769544   0.356626  21.786
## group1:month   3.711581   3.751239   0.989
##
## Correlation of Fixed Effects:
##             (Intr) AGE    EDUCAT group1 month
## AGE         -0.929
## EDUCATION   -0.488  0.145
## group1      -0.015  0.087 -0.191
## month        0.002  0.001  0.010 -0.007
## group1:mnth  0.001  0.000 -0.003  0.086 -0.095
## P Value for Full vs. Reduced Model
## [1] 0.3234868
```

© 2018 Kennedy RE et al. *JAMA Network Open*.

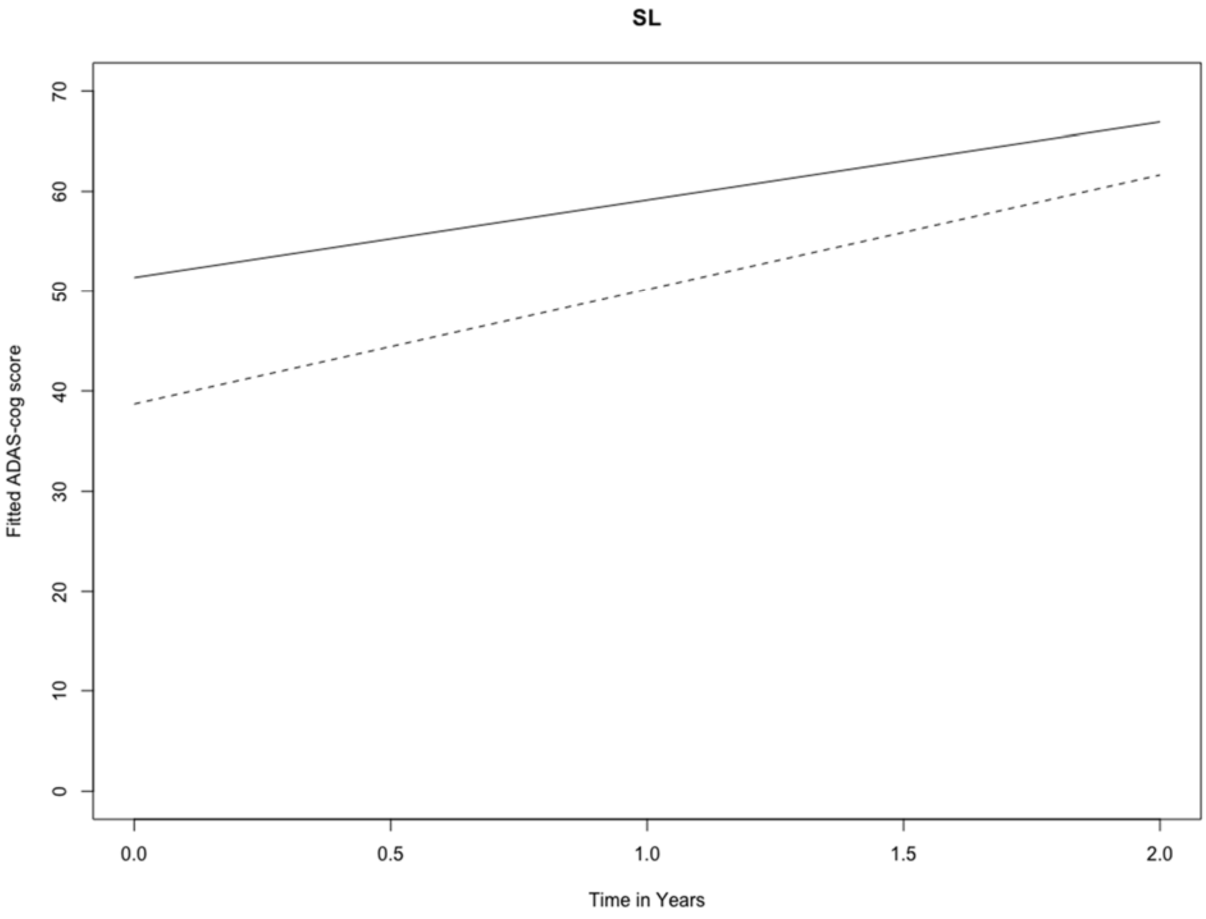


```
## Study Name
## [1] "VN"
## Full Mixed Effects Model
## Linear mixed model fit by maximum likelihood  ['lmerMod']
## Formula: ADAS ~ AGE + EDUCATION + group + month + group:month + (month |
##     UID)
##    Data: mixed.data
##
##      AIC      BIC   logLik deviance df.resid
##   1588.4   1623.1   -784.2   1568.4      227
##
## Scaled residuals:
##     Min      1Q  Median      3Q     Max
## -3.1131 -0.4569 -0.0712  0.4505  3.3913
##
## Random effects:
##  Groups   Name        Variance Std.Dev. Corr
##  UID      (Intercept) 58.55    7.652
##           month       26.75    5.172    0.39
##  Residual             15.17    3.895
```

© 2018 Kennedy RE et al. *JAMA Network Open*.

```
## Number of obs: 237, groups:  UID, 73
##
## Fixed effects:
##              Estimate Std. Error t value
## (Intercept)  23.36473   13.52038   1.728
## AGE           0.08565    0.15289   0.560
## EDUCATION     0.03185    0.26852   0.119
## group1       -3.97281    3.62194  -1.097
## month         7.02839    2.97828   2.360
## group1:month -1.57634    3.11021  -0.507
##
## Correlation of Fixed Effects:
##             (Intr) AGE    EDUCAT group1 month
## AGE         -0.920
## EDUCATION   -0.379  0.086
## group1      -0.348  0.063  0.180
## month        0.042 -0.021  0.001 -0.091
## group1:mnth -0.042  0.022 -0.003  0.096 -0.958
## P Value for Full vs. Reduced Model
## [1] 0.6131325
```

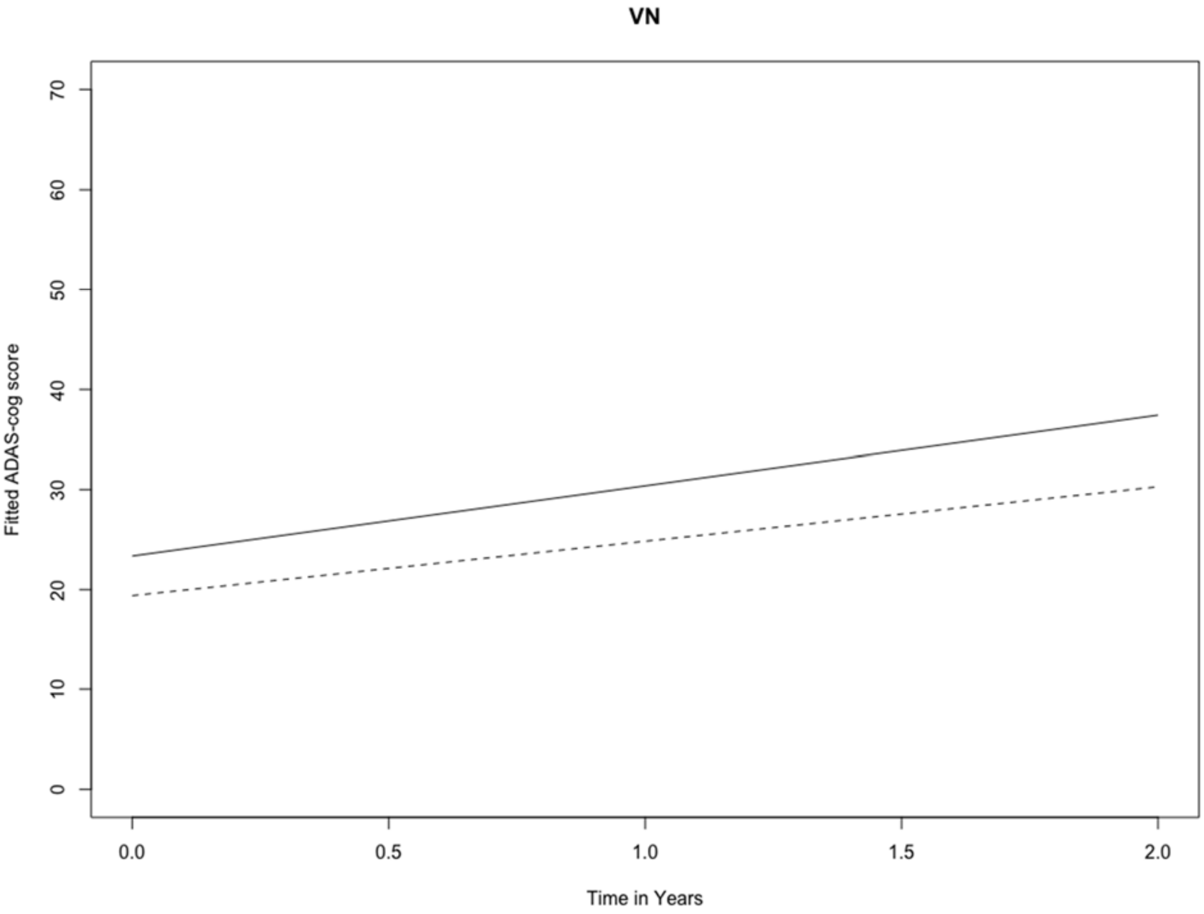

© 2018 Kennedy RE et al. *JAMA Network Open*.

Meta-Analysis with metafor

```
##
## Mixed-Effects Model (k = 10; tau^2 estimator: REML)
##
## tau^2 (estimated amount of residual heterogeneity):     2.7097 (SE = 2.9241)
## tau (square root of estimated tau^2 value):             1.6461
## I^2 (residual heterogeneity / unaccounted variability): 48.20%
## H^2 (unaccounted variability / sampling variability):   1.93
## R^2 (amount of heterogeneity accounted for):            0.00%
##
## Test for Residual Heterogeneity:
## QE(df = 8) = 14.3309, p-val = 0.0735
##
## Test of Moderators (coefficient(s) 2):
## QM(df = 1) = 0.0549, p-val = 0.8148
##
## Model Results:
##
##          estimate      se     zval    pval    ci.lb   ci.ub
## intrcpt    1.2192  1.6763   0.7273  0.4670  -2.0662  4.5046
## mods      -0.0053  0.0224  -0.2343  0.8148  -0.0492  0.0387
##
## ---
## Signif. codes:  0 '***' 0.001 '**' 0.01 '*' 0.05 '.' 0.1 ' ' 1
```

© 2018 Kennedy RE et al. *JAMA Network Open*.

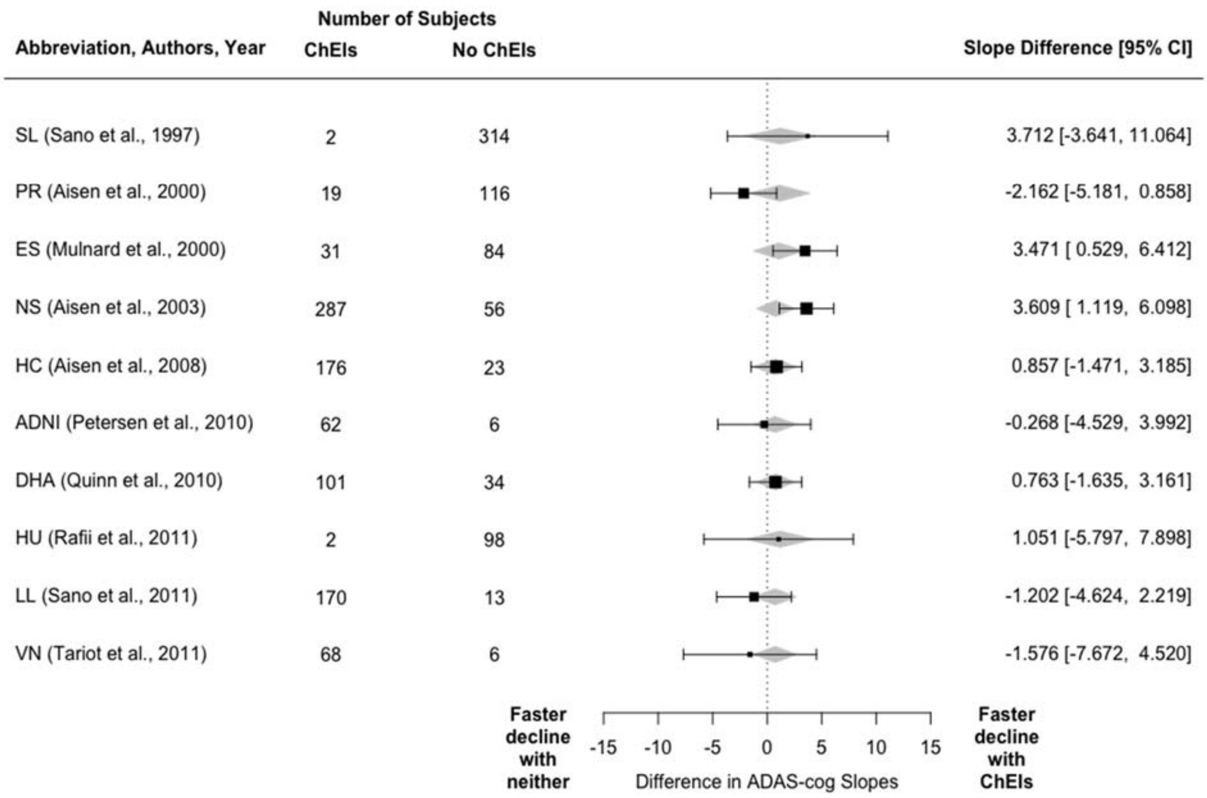

© 2018 Kennedy RE et al. *JAMA Network Open*.

Rates of decline for memantine (+/- ChEIs) vs. ChEIs only

Mixed Models

```
## Study Name
## [1] "ADNI"
## Full Mixed Effects Model
## Linear mixed model fit by maximum likelihood  ['lmerMod']
## Formula: ADAS ~ AGE + EDUCATION + group + month + group:month + (month |
##     UID)
##    Data: mixed.data
##
##      AIC      BIC   logLik deviance df.resid
##   3825.8   3869.9  -1902.9   3805.8      597
##
## Scaled residuals:
##      Min       1Q   Median       3Q      Max
## -2.26251 -0.45376 -0.02347  0.41798  2.59506
##
## Random effects:
##  Groups   Name        Variance Std.Dev. Corr
##  UID      (Intercept) 30.34    5.508
##           month       20.24    4.498    0.48
##  Residual             10.79    3.284
## Number of obs: 607, groups:  UID, 173
##
## Fixed effects:
##              Estimate Std. Error t value
## (Intercept)  13.43112    5.21594   2.575
## AGE           0.06334    0.06045   1.048
## EDUCATION    -0.04356    0.15123  -0.288
## group1        1.50850    1.00789   1.497
## month         4.72455    0.73490   6.429
## group1:month  0.50066    0.89188   0.561
##
## Correlation of Fixed Effects:
##             (Intr) AGE    EDUCAT group1 month
## AGE         -0.900
## EDUCATION   -0.426  0.019
## group1      -0.202  0.162 -0.155
## month        0.021  0.008 -0.002 -0.141
## group1:mnth -0.013 -0.009 -0.005  0.182 -0.824
## P Value for Full vs. Reduced Model
## [1] 0.5752262
```

© 2018 Kennedy RE et al. *JAMA Network Open*.

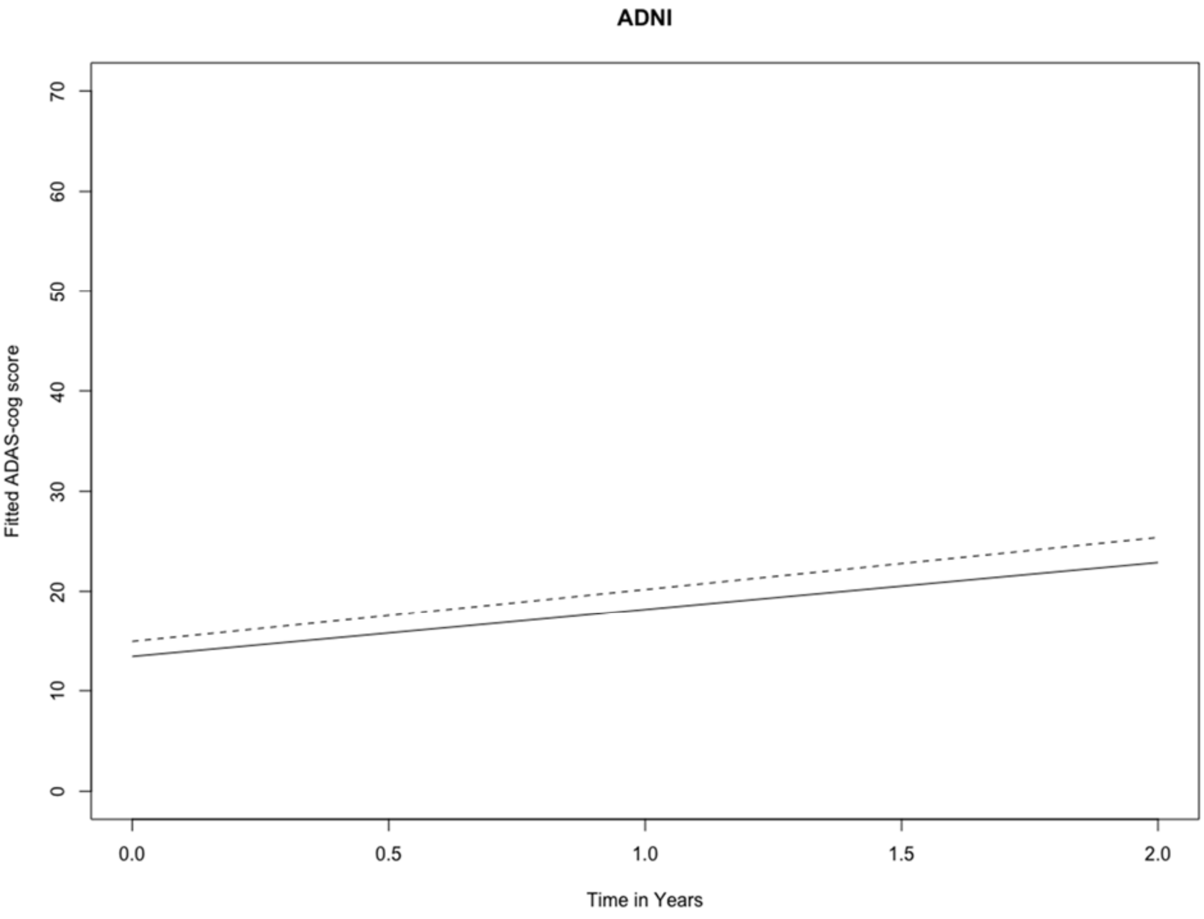


```
## Study Name
## [1] "DHA"
## Full Mixed Effects Model
## Linear mixed model fit by maximum likelihood  ['lmerMod']
## Formula: ADAS ~ AGE + EDUCATION + group + month + group:month + (month |
##     UID)
##    Data: mixed.data
##
##      AIC      BIC   logLik deviance df.resid
##   7545.2   7595.9  -3762.6   7525.2     1170
##
## Scaled residuals:
##      Min       1Q   Median       3Q      Max
## -2.61384 -0.47988  0.00245  0.47883  2.57241
##
## Random effects:
##  Groups   Name        Variance Std.Dev. Corr
##  UID      (Intercept) 57.56    7.587
##           month       22.30    4.722    0.57
##  Residual             10.91    3.302
```

© 2018 Kennedy RE et al. *JAMA Network Open*.

```
## Number of obs: 1180, groups:  UID, 353
##
## Fixed effects:
##              Estimate Std. Error t value
## (Intercept)   15.4286     4.7024   3.281
## AGE            0.1166     0.0496   2.350
## EDUCATION     -0.2601     0.1509  -1.723
## group1         4.9019     0.9590   5.111
## month          3.9729     0.6114   6.498
## group1:month   2.0387     0.7250   2.812
##
## Correlation of Fixed Effects:
##             (Intr) AGE    EDUCAT group1 month
## AGE         -0.881
## EDUCATION   -0.579  0.163
## group1      -0.151  0.048 -0.071
## month        0.054 -0.011  0.000 -0.223
## group1:mnth -0.036  0.003 -0.009  0.260 -0.843
## P Value for Full vs. Reduced Model
## [1] 0.005062576
```

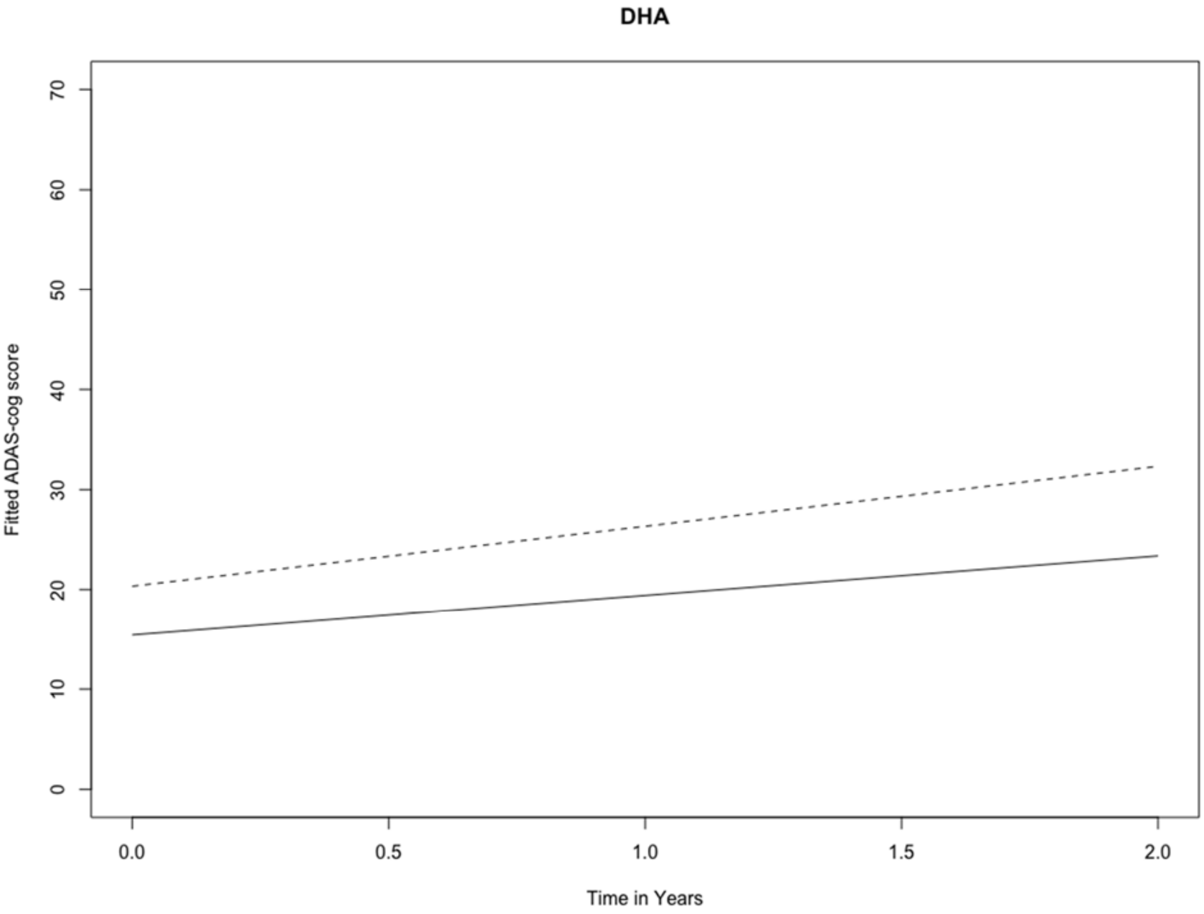

© 2018 Kennedy RE et al. *JAMA Network Open*.

```
## Study Name
## [1] "HC"
## Full Mixed Effects Model
## Linear mixed model fit by maximum likelihood  ['lmerMod']
## Formula: ADAS ~ AGE + EDUCATION + group + month + group:month + (month |
##     UID)
##    Data: mixed.data
##
##      AIC      BIC   logLik deviance df.resid
##   8260.1   8311.6  -4120.0   8240.1     1273
##
## Scaled residuals:
##     Min      1Q  Median      3Q     Max
## -3.1929 -0.5029 -0.0233  0.4700  4.0560
##
## Random effects:
##  Groups   Name        Variance Std.Dev. Corr
##  UID      (Intercept) 54.89    7.409
##           month       16.41    4.051    0.42
##  Residual             13.12    3.622
## Number of obs: 1283, groups:  UID, 361
##
## Fixed effects:
##             Estimate Std. Error t value
## (Intercept)  15.01354    4.88852   3.071
## AGE           0.16131    0.05513   2.926
## EDUCATION    -0.49051    0.14001  -3.503
## group1        4.01666    0.85470   4.699
## month         2.95427    0.43377   6.811
## group1:month  2.76470    0.59132   4.675
##
## Correlation of Fixed Effects:
##             (Intr) AGE    EDUCAT group1 month
## AGE         -0.912
## EDUCATION   -0.480  0.101
## group1      -0.138  0.093 -0.086
## month        0.023 -0.009 -0.008 -0.066
## group1:mnth -0.016  0.005  0.010  0.089 -0.734
## P Value for Full vs. Reduced Model
## [1] 4.473328e-06
```

© 2018 Kennedy RE et al. *JAMA Network Open*.

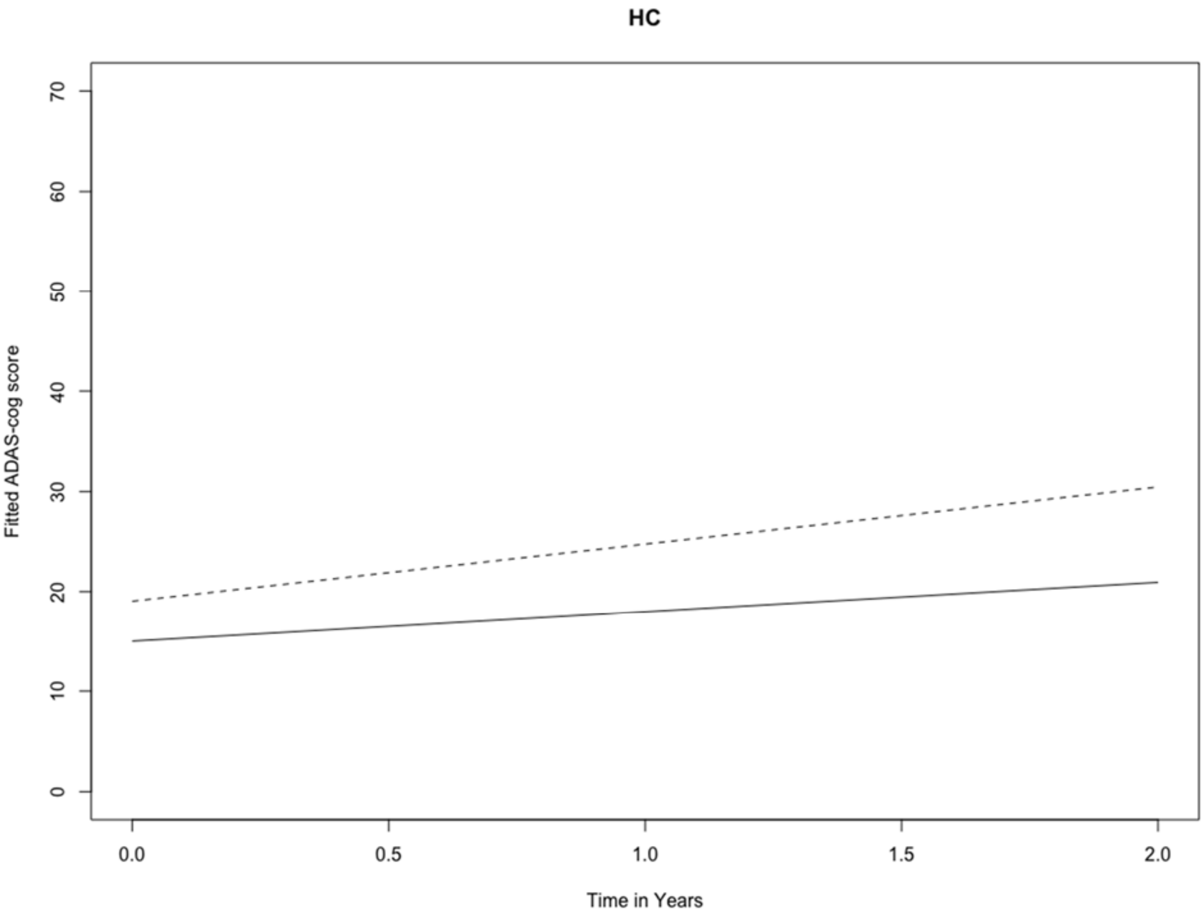


```
## Study Name
## [1] "HU"
## Full Mixed Effects Model
## Linear mixed model fit by maximum likelihood  ['lmerMod']
## Formula: ADAS ~ AGE + EDUCATION + group + month + group:month + (month |
##     UID)
##    Data: mixed.data
##
##      AIC      BIC   logLik deviance df.resid
##   1681.8   1716.9   -830.9   1661.8      236
##
## Scaled residuals:
##      Min       1Q   Median       3Q      Max
## -2.16585 -0.47352 -0.01075  0.48386  2.31685
##
## Random effects:
##  Groups   Name        Variance Std.Dev. Corr
##  UID      (Intercept) 107.423  10.364
##           month         2.608   1.615   0.75
##  Residual              15.910   3.989
```

© 2018 Kennedy RE et al. *JAMA Network Open*.

```
## Number of obs: 246, groups:  UID, 97
##
## Fixed effects:
##               Estimate Std. Error t value
## (Intercept)   52.9841    15.5889   3.399
## AGE           -0.2617     0.1536  -1.703
## EDUCATION     -0.5873     0.3212  -1.828
## group1         4.7030     7.8414   0.600
## month          2.6606     3.3151   0.803
## group1:month  -1.0131     3.3770  -0.300
##
## Correlation of Fixed Effects:
##             (Intr) AGE    EDUCAT group1 month
## AGE         -0.804
## EDUCATION   -0.413  0.107
## group1      -0.532  0.015  0.097
## month        0.006  0.004  0.011 -0.024
## group1:mnth -0.006 -0.004 -0.010  0.024 -0.982
## P Value for Full vs. Reduced Model
## [1] 0.7669275
```

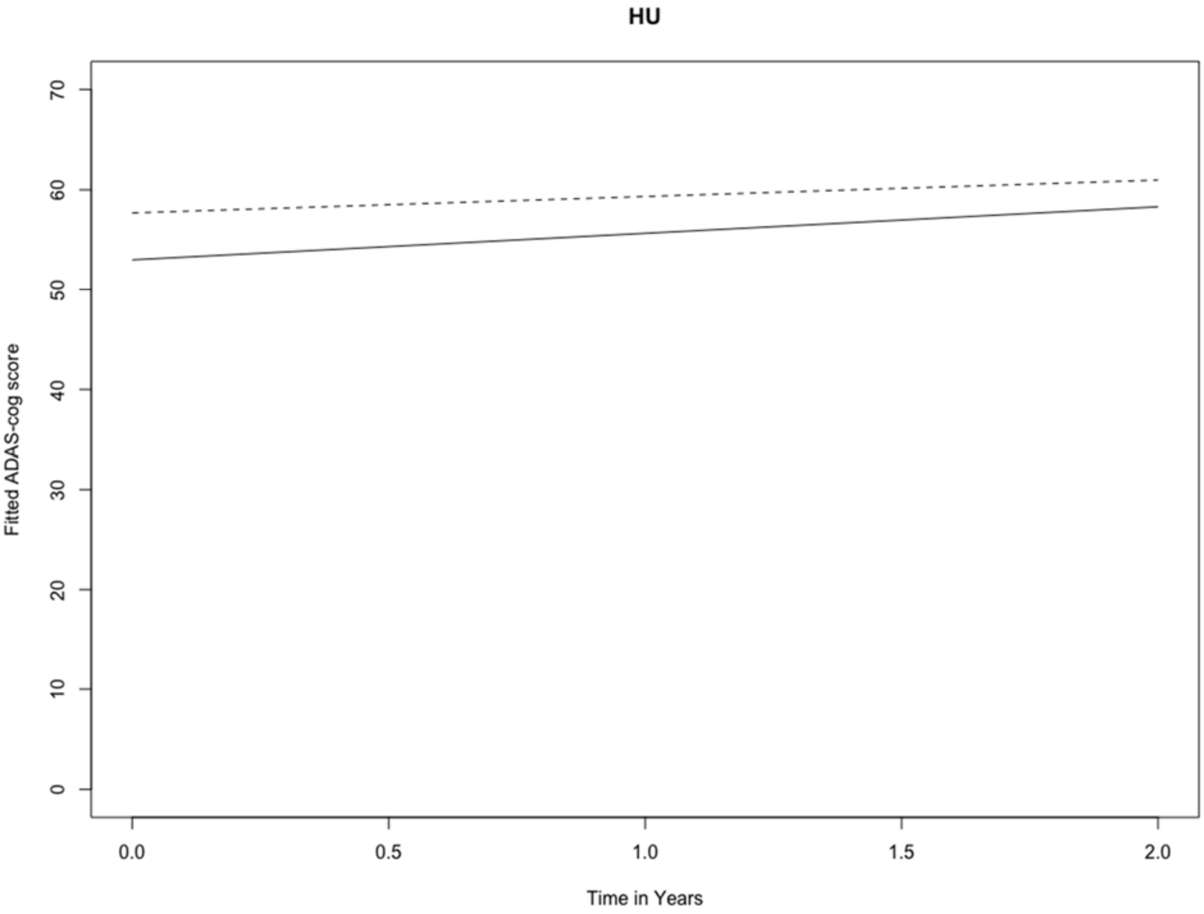

© 2018 Kennedy RE et al. *JAMA Network Open*.

```
## Study Name
## [1] "LL"
## Full Mixed Effects Model
## Linear mixed model fit by maximum likelihood  ['lmerMod']
## Formula: ADAS ~ AGE + EDUCATION + group + month + group:month + (month |
##     UID)
##    Data: mixed.data
##
##      AIC      BIC   logLik deviance df.resid
##   8060.9   8111.9  -4020.4   8040.9     1209
##
## Scaled residuals:
##      Min       1Q   Median       3Q      Max
## -2.45904 -0.48528 -0.00988  0.45778  2.97316
##
## Random effects:
##  Groups   Name        Variance Std.Dev. Corr
##  UID      (Intercept) 82.58    9.087
##           month       18.82    4.339    0.34
##  Residual             13.63    3.692
## Number of obs: 1219, groups:  UID, 373
##
## Fixed effects:
##              Estimate Std. Error t value
## (Intercept)  20.488283   4.829999   4.242
## AGE           0.006948   0.054431   0.128
## EDUCATION     0.010258   0.159901   0.064
## group1        5.056426   1.026693   4.925
## month         3.977156   0.500995   7.939
## group1:month  2.422437   0.657554   3.684
##
## Correlation of Fixed Effects:
##             (Intr) AGE    EDUCAT group1 month
## AGE         -0.878
## EDUCATION   -0.505  0.060
## group1      -0.144  0.123 -0.160
## month        0.014 -0.001 -0.006 -0.049
## group1:mnth -0.010  0.000  0.005  0.066 -0.762
## P Value for Full vs. Reduced Model
## [1] 0.000262101
```

© 2018 Kennedy RE et al. *JAMA Network Open*.

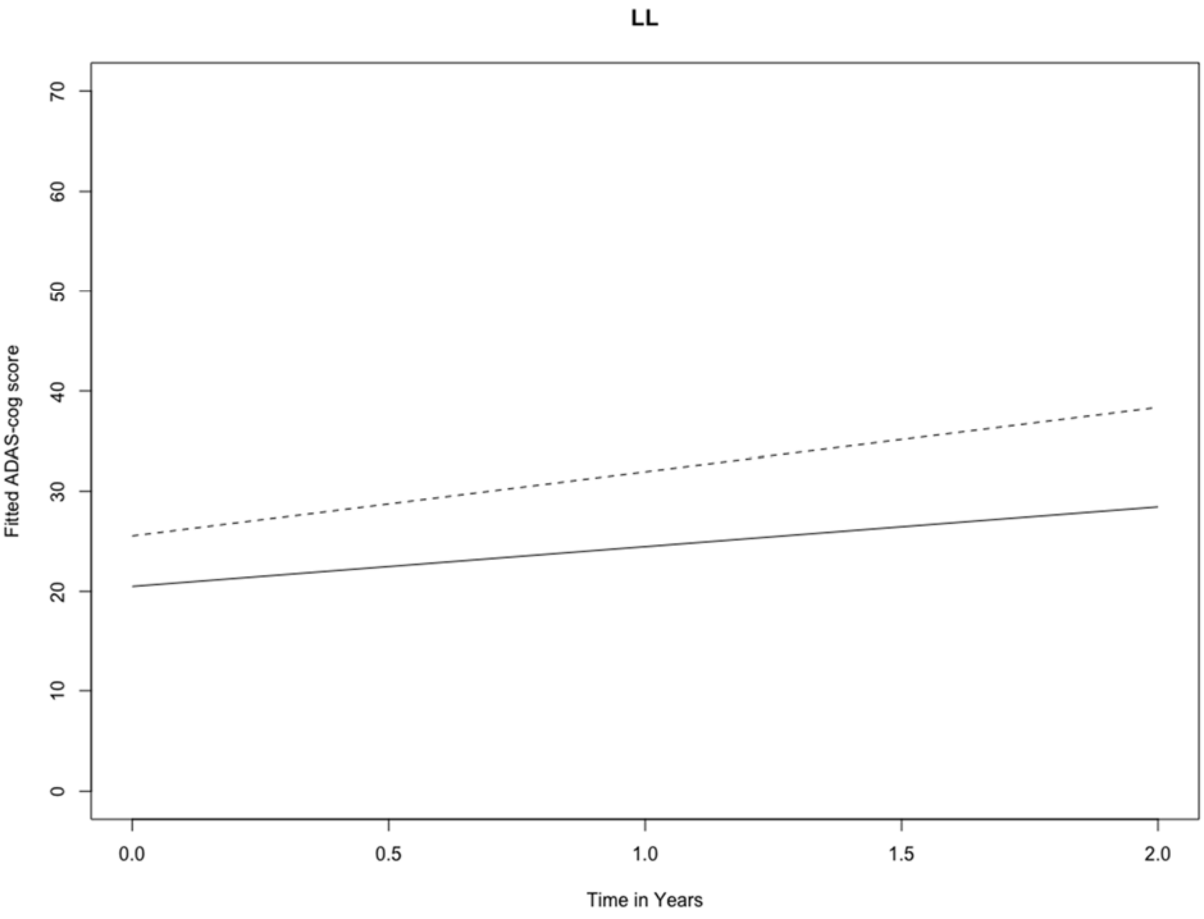


```
## Study Name
## [1] "VN"
## Full Mixed Effects Model
## Linear mixed model fit by maximum likelihood  ['lmerMod']
## Formula: ADAS ~ AGE + EDUCATION + group + month + group:month + (month |
##     UID)
##    Data: mixed.data
##
##      AIC      BIC   logLik deviance df.resid
##   5633.5   5681.0  -2806.8   5613.5      840
##
## Scaled residuals:
##     Min      1Q  Median      3Q     Max
## -2.9715 -0.4960 -0.0157  0.4295  3.9849
##
## Random effects:
##  Groups   Name        Variance Std.Dev. Corr
##  UID      (Intercept) 63.44    7.965
##           month       13.59    3.686    0.32
##  Residual             16.11    4.014
```

© 2018 Kennedy RE et al. *JAMA Network Open*.

```
## Number of obs: 850, groups:  UID, 275
##
## Fixed effects:
##              Estimate Std. Error t value
## (Intercept)  35.33160    5.90774   5.981
## AGE          -0.09639    0.06730  -1.432
## EDUCATION    -0.09911    0.15953  -0.621
## group1        3.32177    1.25067   2.656
## month         5.06683    0.72732   6.966
## group1:month  1.43710    0.84547   1.700
##
## Correlation of Fixed Effects:
##             (Intr) AGE    EDUCAT group1 month
## AGE         -0.922
## EDUCATION   -0.433  0.098
## group1      -0.234  0.149 -0.146
## month        0.002  0.002 -0.003 -0.010
## group1:mnth -0.002 -0.002  0.006  0.009 -0.860
## P Value for Full vs. Reduced Model
## [1] 0.0932488
```

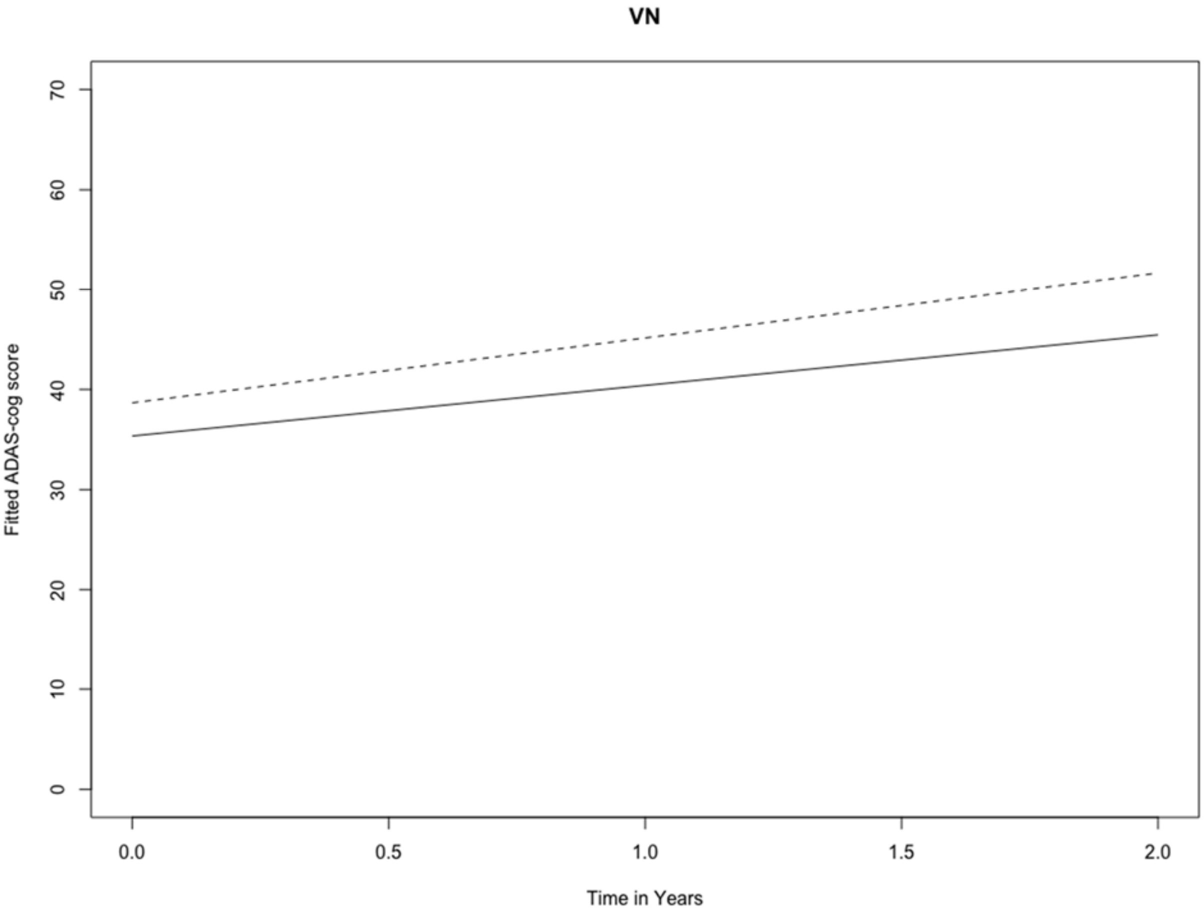

© 2018 Kennedy RE et al. *JAMA Network Open*.

Meta-analysis with metafor

```
##
## Random-Effects Model (k = 6; tau^2 estimator: REML)
##
## tau^2 (estimated amount of total heterogeneity): 0.1538 (SE = 0.4763)
## tau (square root of estimated tau^2 value):      0.3922
## I^2 (total heterogeneity / total variability):   19.17%
## H^2 (total variability / sampling variability):  1.24
##
## Test for Heterogeneity:
## Q(df = 5) = 6.1442, p-val = 0.2924
##
## Model Results:
##
## estimate      se    zval    pval   ci.lb   ci.ub
##   1.9743  0.3662  5.3907  <.0001  1.2565  2.6921  ***
##
## ---
## Signif. codes:  0 '***' 0.001 '**' 0.01 '*' 0.05 '.' 0.1 ' ' 1
```

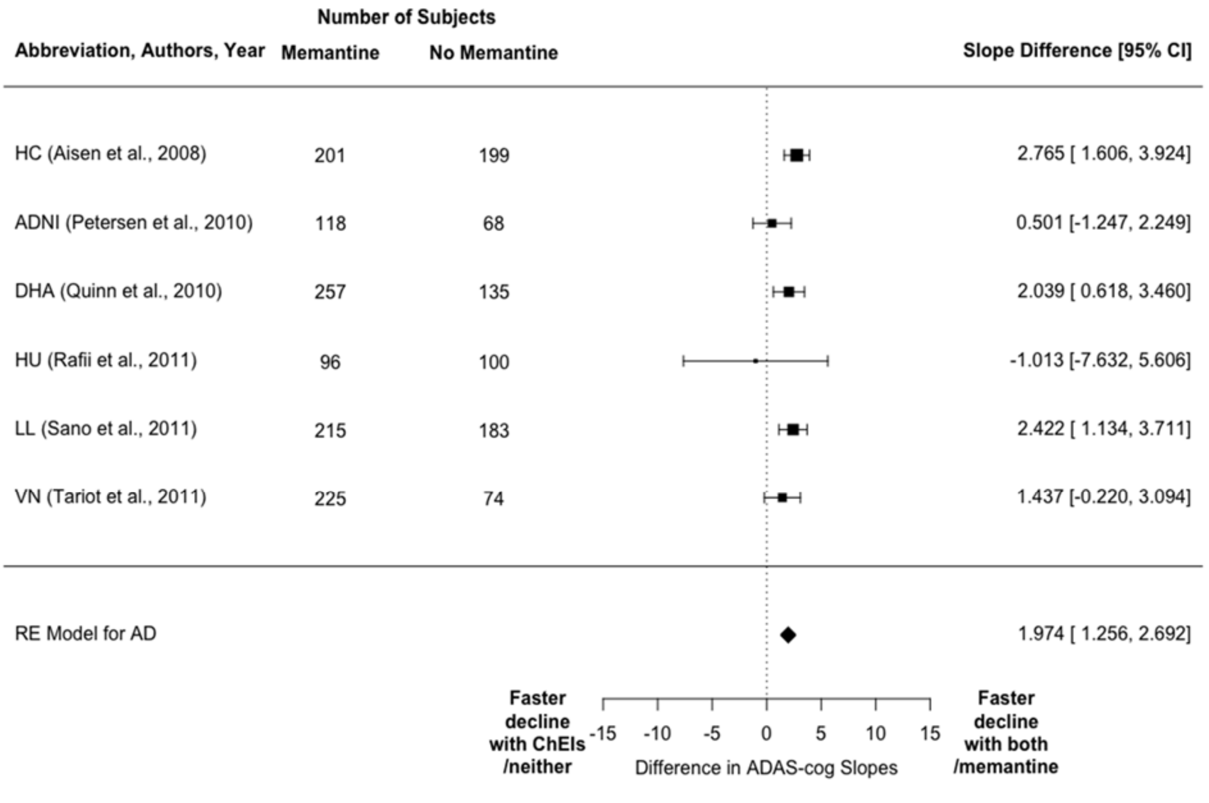

© 2018 Kennedy RE et al. *JAMA Network Open*.

© 2018 Kennedy RE et al. *JAMA Network Open*.

Rates of decline for memantine (+/- ChEIs) vs. ChEIs only, with moderator of study year

Mixed Models

```
## Study Name
## [1] "ADNI"
## Full Mixed Effects Model
## Linear mixed model fit by maximum likelihood  ['lmerMod']
## Formula: ADAS ~ AGE + EDUCATION + group + month + group:month + (month |
##     UID)
##    Data: mixed.data
##
##      AIC      BIC   logLik deviance df.resid
##   3825.8   3869.9  -1902.9   3805.8      597
##
## Scaled residuals:
##      Min       1Q   Median       3Q      Max
## -2.26251 -0.45376 -0.02347  0.41798  2.59506
##
## Random effects:
##  Groups   Name        Variance Std.Dev. Corr
##  UID      (Intercept) 30.34    5.508
##           month       20.24    4.498    0.48
##  Residual             10.79    3.284
## Number of obs: 607, groups:  UID, 173
##
## Fixed effects:
##             Estimate Std. Error t value
## (Intercept)  13.43112    5.21594   2.575
## AGE           0.06334    0.06045   1.048
## EDUCATION    -0.04356    0.15123  -0.288
## group1        1.50850    1.00789   1.497
## month         4.72455    0.73490   6.429
## group1:month  0.50066    0.89188   0.561
##
## Correlation of Fixed Effects:
##             (Intr) AGE    EDUCAT group1 month
## AGE         -0.900
## EDUCATION   -0.426  0.019
## group1      -0.202  0.162 -0.155
## month        0.021  0.008 -0.002 -0.141
## group1:mnth -0.013 -0.009 -0.005  0.182 -0.824
## P Value for Full vs. Reduced Model
## [1] 0.5752262
```

© 2018 Kennedy RE et al. *JAMA Network Open*.

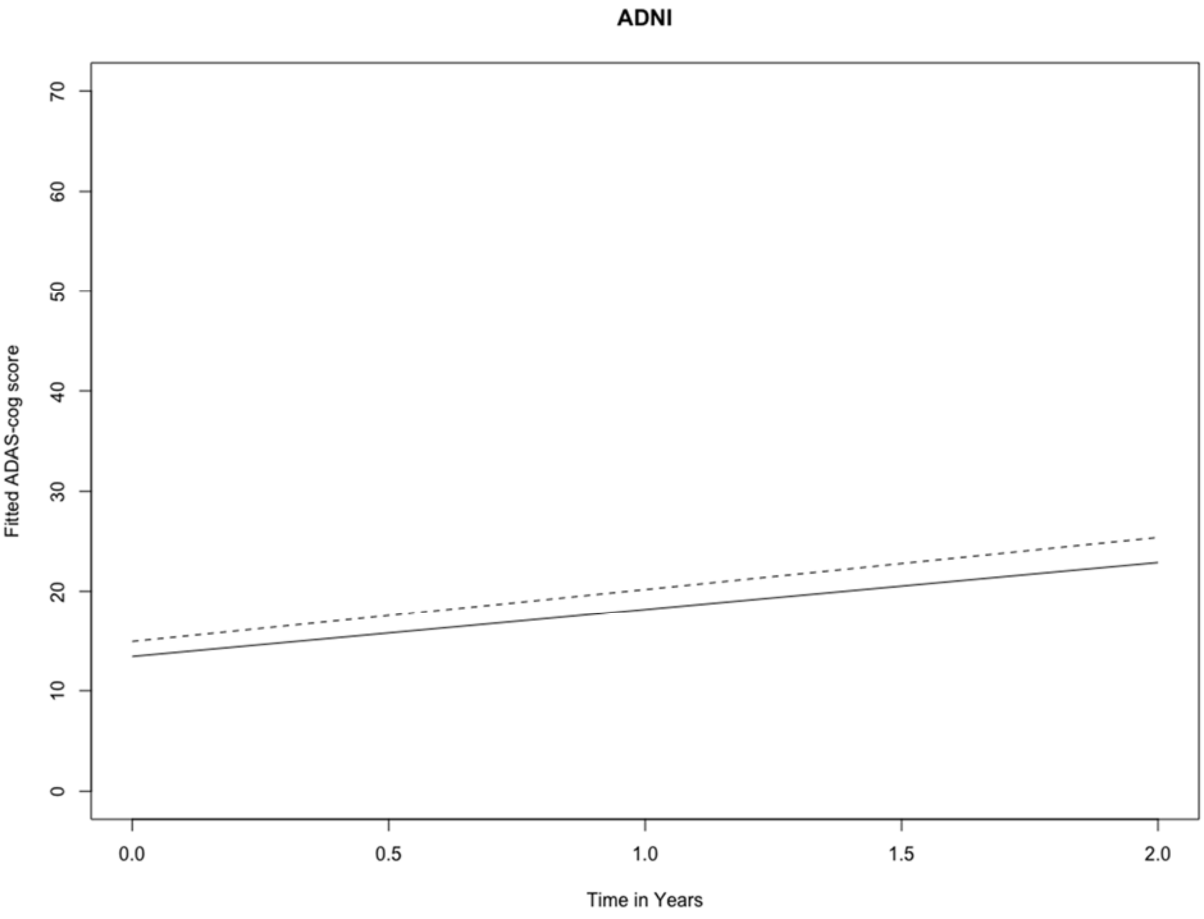


```
## Study Name
## [1] "DHA"
## Full Mixed Effects Model
## Linear mixed model fit by maximum likelihood  ['lmerMod']
## Formula: ADAS ~ AGE + EDUCATION + group + month + group:month + (month |
##     UID)
##    Data: mixed.data
##
##      AIC      BIC   logLik deviance df.resid
##   7545.2   7595.9  -3762.6   7525.2     1170
##
## Scaled residuals:
##      Min       1Q   Median       3Q      Max
## -2.61384 -0.47988  0.00245  0.47883  2.57241
##
## Random effects:
##  Groups   Name        Variance Std.Dev. Corr
##  UID      (Intercept) 57.56    7.587
##           month       22.30    4.722    0.57
##  Residual             10.91    3.302
```

© 2018 Kennedy RE et al. *JAMA Network Open*.

```
## Number of obs: 1180, groups:  UID, 353
##
## Fixed effects:
##               Estimate Std. Error t value
## (Intercept)   15.4286     4.7024   3.281
## AGE            0.1166     0.0496   2.350
## EDUCATION     -0.2601     0.1509  -1.723
## group1         4.9019     0.9590   5.111
## month          3.9729     0.6114   6.498
## group1:month   2.0387     0.7250   2.812
##
## Correlation of Fixed Effects:
##              (Intr) AGE    EDUCAT group1 month
## AGE          -0.881
## EDUCATION    -0.579  0.163
## group1       -0.151  0.048 -0.071
## month         0.054 -0.011  0.000 -0.223
## group1:mnth -0.036  0.003 -0.009  0.260 -0.843
## P Value for Full vs. Reduced Model
## [1] 0.005062576
```

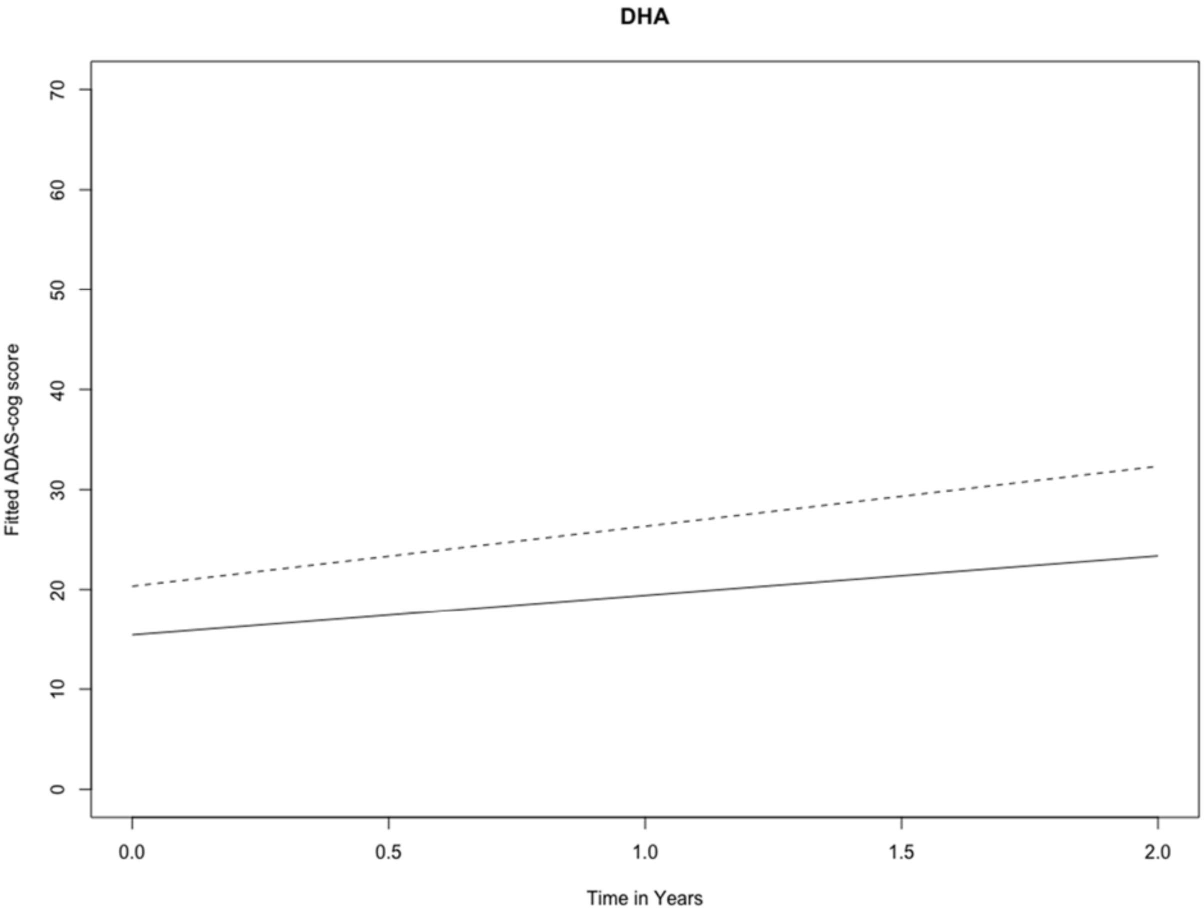

© 2018 Kennedy RE et al. *JAMA Network Open*.

```
## Study Name
## [1] "HC"
## Full Mixed Effects Model
## Linear mixed model fit by maximum likelihood  ['lmerMod']
## Formula: ADAS ~ AGE + EDUCATION + group + month + group:month + (month |
##     UID)
##    Data: mixed.data
##
##      AIC      BIC   logLik deviance df.resid
##   8260.1   8311.6  -4120.0   8240.1     1273
##
## Scaled residuals:
##     Min      1Q  Median      3Q     Max
## -3.1929 -0.5029 -0.0233  0.4700  4.0560
##
## Random effects:
##  Groups   Name        Variance Std.Dev. Corr
##  UID      (Intercept) 54.89    7.409
##           month       16.41    4.051    0.42
##  Residual             13.12    3.622
## Number of obs: 1283, groups:  UID, 361
##
## Fixed effects:
##             Estimate Std. Error t value
## (Intercept)  15.01354    4.88852   3.071
## AGE           0.16131    0.05513   2.926
## EDUCATION    -0.49051    0.14001  -3.503
## group1        4.01666    0.85470   4.699
## month         2.95427    0.43377   6.811
## group1:month  2.76470    0.59132   4.675
##
## Correlation of Fixed Effects:
##             (Intr) AGE    EDUCAT group1 month
## AGE         -0.912
## EDUCATION   -0.480  0.101
## group1      -0.138  0.093 -0.086
## month        0.023 -0.009 -0.008 -0.066
## group1:mnth -0.016  0.005  0.010  0.089 -0.734
## P Value for Full vs. Reduced Model
## [1] 4.473328e-06
```

© 2018 Kennedy RE et al. *JAMA Network Open*.

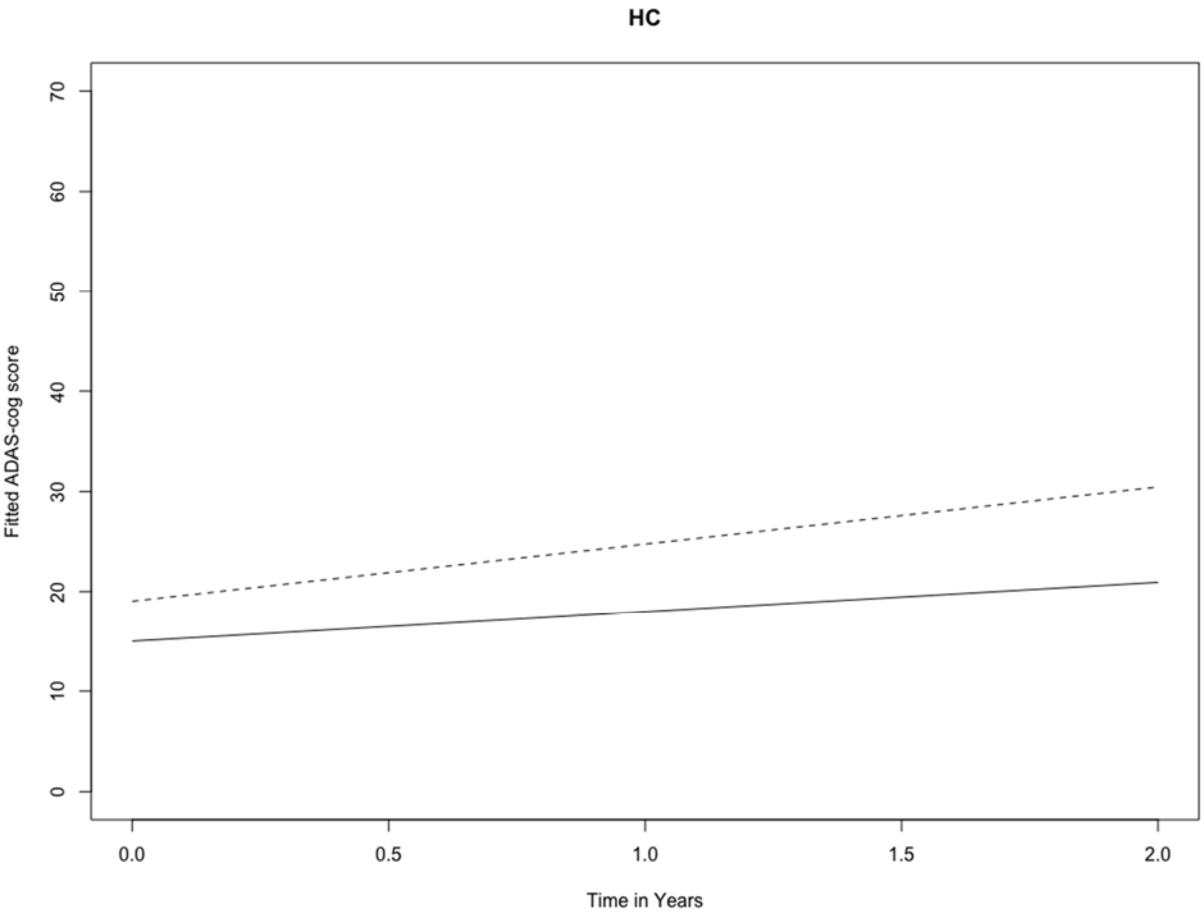


```
## Study Name
## [1] "HU"
## Full Mixed Effects Model
## Linear mixed model fit by maximum likelihood  ['lmerMod']
## Formula: ADAS ~ AGE + EDUCATION + group + month + group:month + (month |
##     UID)
##    Data: mixed.data
##
##      AIC      BIC   logLik deviance df.resid
##   1681.8   1716.9   -830.9   1661.8      236
##
## Scaled residuals:
##      Min       1Q   Median       3Q      Max
## -2.16585 -0.47352 -0.01075  0.48386  2.31685
##
## Random effects:
##  Groups   Name        Variance Std.Dev. Corr
##  UID      (Intercept) 107.423  10.364
##           month         2.608   1.615   0.75
##  Residual              15.910   3.989
```

© 2018 Kennedy RE et al. *JAMA Network Open*.

```
## Number of obs: 246, groups:  UID, 97
##
## Fixed effects:
##              Estimate Std. Error t value
## (Intercept)   52.9841    15.5889   3.399
## AGE           -0.2617     0.1536  -1.703
## EDUCATION     -0.5873     0.3212  -1.828
## group1         4.7030     7.8414   0.600
## month          2.6606     3.3151   0.803
## group1:month  -1.0131     3.3770  -0.300
##
## Correlation of Fixed Effects:
##             (Intr) AGE    EDUCAT group1 month
## AGE         -0.804
## EDUCATION   -0.413  0.107
## group1      -0.532  0.015  0.097
## month        0.006  0.004  0.011 -0.024
## group1:mnth -0.006 -0.004 -0.010  0.024 -0.982
## P Value for Full vs. Reduced Model
## [1] 0.7669275
```

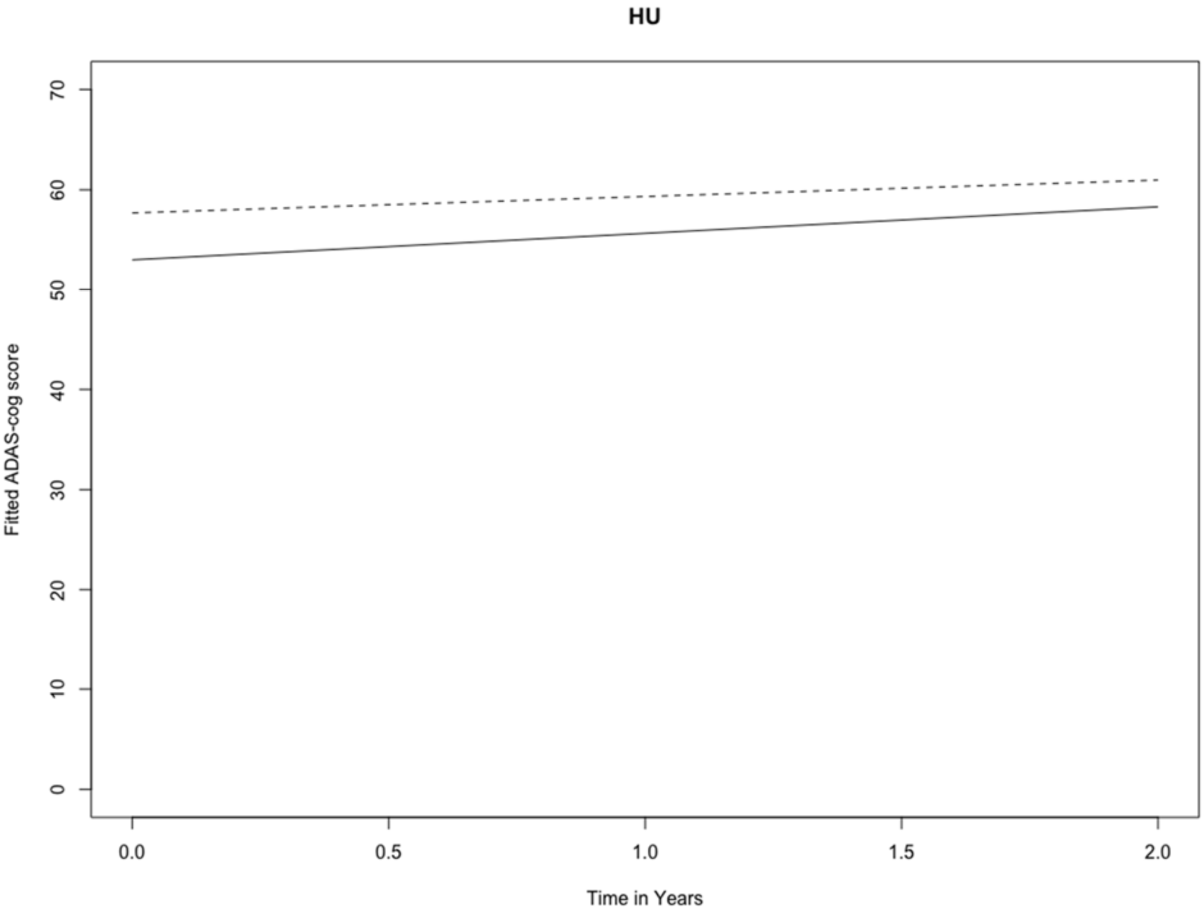

© 2018 Kennedy RE et al. *JAMA Network Open*.

```
## Study Name
## [1] "LL"
## Full Mixed Effects Model
## Linear mixed model fit by maximum likelihood  ['lmerMod']
## Formula: ADAS ~ AGE + EDUCATION + group + month + group:month + (month |
##     UID)
##    Data: mixed.data
##
##      AIC      BIC   logLik deviance df.resid
##   8060.9   8111.9  -4020.4   8040.9     1209
##
## Scaled residuals:
##      Min       1Q   Median       3Q      Max
## -2.45904 -0.48528 -0.00988  0.45778  2.97316
##
## Random effects:
##  Groups   Name        Variance Std.Dev. Corr
##  UID      (Intercept) 82.58    9.087
##           month       18.82    4.339    0.34
##  Residual             13.63    3.692
## Number of obs: 1219, groups:  UID, 373
##
## Fixed effects:
##              Estimate Std. Error t value
## (Intercept)  20.488283   4.829999   4.242
## AGE           0.006948   0.054431   0.128
## EDUCATION     0.010258   0.159901   0.064
## group1        5.056426   1.026693   4.925
## month         3.977156   0.500995   7.939
## group1:month  2.422437   0.657554   3.684
##
## Correlation of Fixed Effects:
##             (Intr) AGE    EDUCAT group1 month
## AGE         -0.878
## EDUCATION   -0.505  0.060
## group1      -0.144  0.123 -0.160
## month        0.014 -0.001 -0.006 -0.049
## group1:mnth -0.010  0.000  0.005  0.066 -0.762
## P Value for Full vs. Reduced Model
## [1] 0.000262101
```

© 2018 Kennedy RE et al. *JAMA Network Open*.

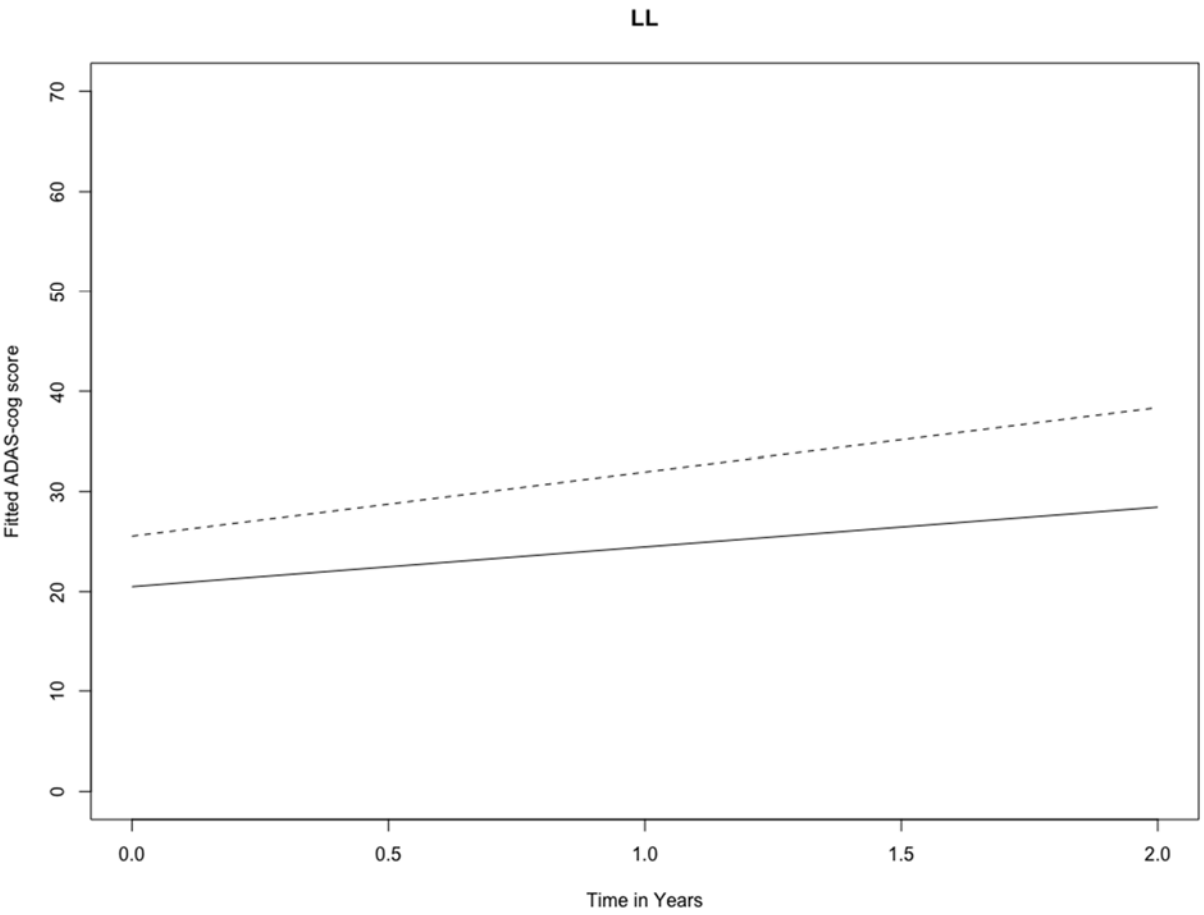


```
## Study Name
## [1] "VN"
## Full Mixed Effects Model
## Linear mixed model fit by maximum likelihood  ['lmerMod']
## Formula: ADAS ~ AGE + EDUCATION + group + month + group:month + (month |
##     UID)
##    Data: mixed.data
##
##      AIC      BIC   logLik deviance df.resid
##   5633.5   5681.0  -2806.8   5613.5      840
##
## Scaled residuals:
##     Min      1Q  Median      3Q     Max
## -2.9715 -0.4960 -0.0157  0.4295  3.9849
##
## Random effects:
##  Groups   Name        Variance Std.Dev. Corr
##  UID      (Intercept) 63.44    7.965
##           month       13.59    3.686    0.32
##  Residual             16.11    4.014
```

© 2018 Kennedy RE et al. *JAMA Network Open*.

```
## Number of obs: 850, groups:  UID, 275
##
## Fixed effects:
##              Estimate Std. Error t value
## (Intercept)  35.33160    5.90774   5.981
## AGE          -0.09639    0.06730  -1.432
## EDUCATION    -0.09911    0.15953  -0.621
## group1        3.32177    1.25067   2.656
## month         5.06683    0.72732   6.966
## group1:month  1.43710    0.84547   1.700
##
## Correlation of Fixed Effects:
##             (Intr) AGE    EDUCAT group1 month
## AGE         -0.922
## EDUCATION   -0.433  0.098
## group1      -0.234  0.149 -0.146
## month        0.002  0.002 -0.003 -0.010
## group1:mnth -0.002 -0.002  0.006  0.009 -0.860
## P Value for Full vs. Reduced Model
## [1] 0.0932488
```

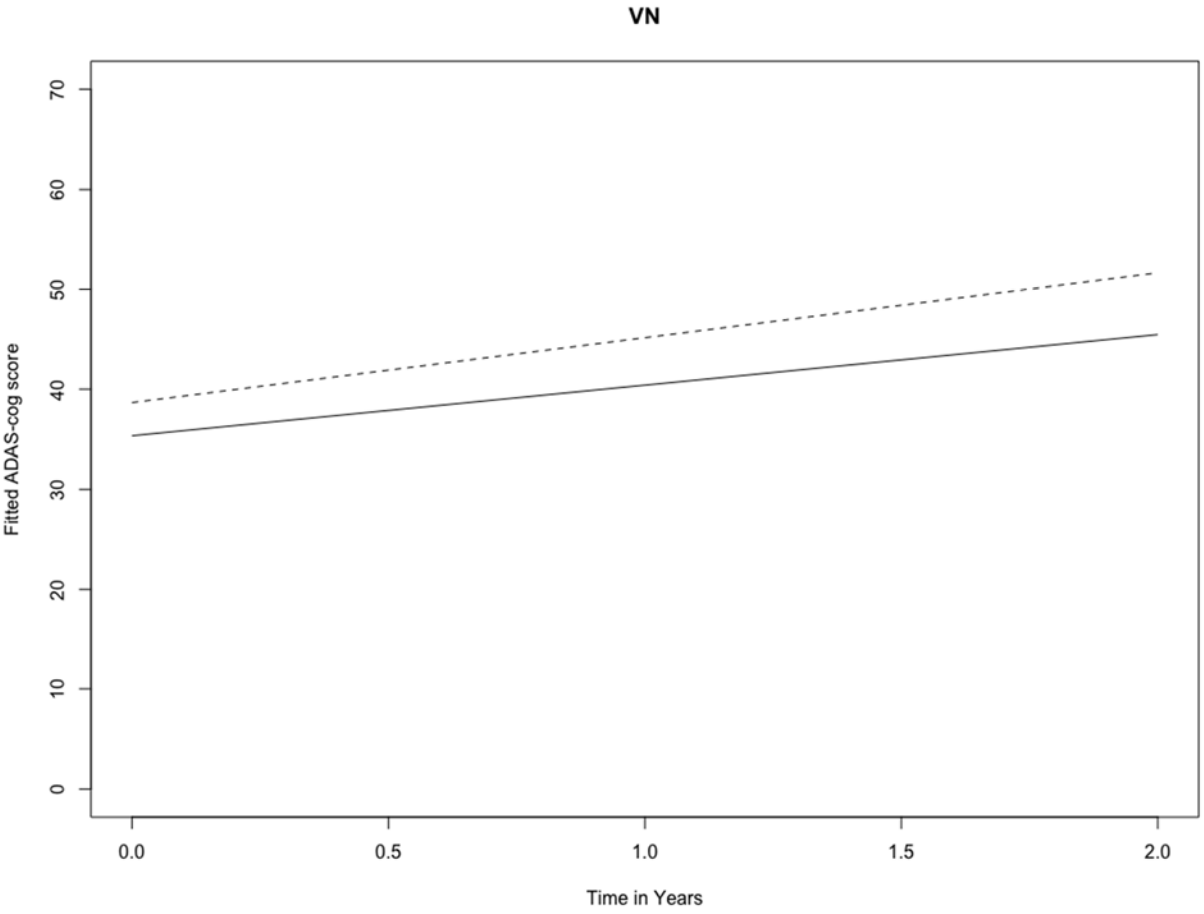

© 2018 Kennedy RE et al. *JAMA Network Open*.

```
##
## Mixed-Effects Model (k = 6; tau^2 estimator: REML)
##
## tau^2 (estimated amount of residual heterogeneity):     0.2143 (SE = 0.6541)
## tau (square root of estimated tau^2 value):             0.4629
## I^2 (residual heterogeneity / unaccounted variability): 21.80%
## H^2 (unaccounted variability / sampling variability):   1.28
## R^2 (amount of heterogeneity accounted for):            0.00%
##
## Test for Residual Heterogeneity:
## QE(df = 4) = 4.8477, p-val = 0.3033
##
## Test of Moderators (coefficient(s) 2):
## QM(df = 1) = 0.9030, p-val = 0.3420
##
## Model Results:
##
##          estimate      se     zval    pval    ci.lb    ci.ub
## intrcpt    5.8868  4.1525   1.4177  0.1563  -2.2518  14.0255
## mods      -0.3051  0.3211  -0.9503  0.3420  -0.9344   0.3242
##
## ---
## Signif. codes:  0 '***' 0.001 '**' 0.01 '*' 0.05 '.' 0.1 ' ' 1
```

© 2018 Kennedy RE et al. *JAMA Network Open*.

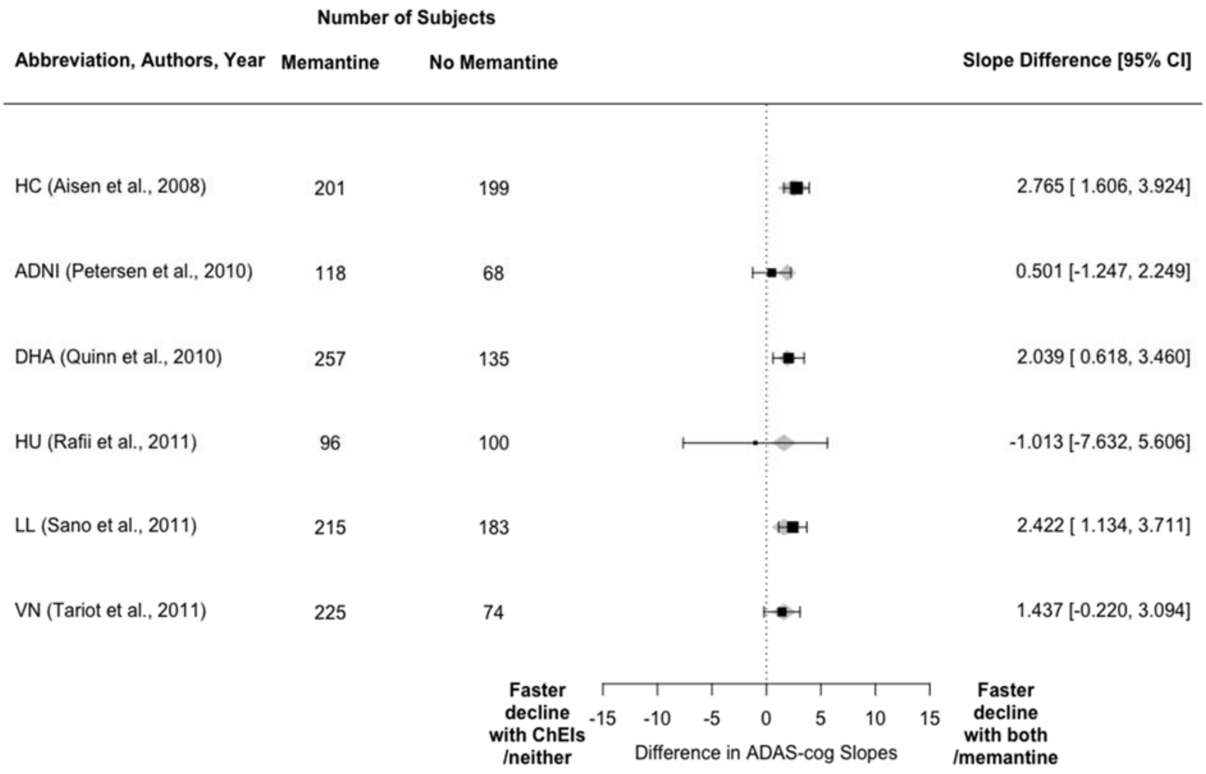

© 2018 Kennedy RE et al. *JAMA Network Open*.

© 2018 Kennedy RE et al. *JAMA Network Open*.

Rates of decline for memantine (+/- ChEIs) vs. ChEIs only, with moderator of % taking meds

```
## Study Name
## [1] "ADNI"
## Full Mixed Effects Model
## Linear mixed model fit by maximum likelihood  ['lmerMod']
## Formula: ADAS ~ AGE + EDUCATION + group + month + group:month + (month |
##     UID)
##    Data: mixed.data
##
##      AIC      BIC   logLik deviance df.resid
##   3825.8   3869.9  -1902.9   3805.8      597
##
## Scaled residuals:
##      Min       1Q   Median       3Q      Max
## -2.26251 -0.45376 -0.02347  0.41798  2.59506
##
## Random effects:
##  Groups   Name        Variance Std.Dev. Corr
##  UID      (Intercept) 30.34    5.508
##           month       20.24    4.498    0.48
##  Residual             10.79    3.284
## Number of obs: 607, groups:  UID, 173
##
## Fixed effects:
##              Estimate Std. Error t value
## (Intercept)  13.43112    5.21594   2.575
## AGE           0.06334    0.06045   1.048
## EDUCATION    -0.04356    0.15123  -0.288
## group1        1.50850    1.00789   1.497
## month         4.72455    0.73490   6.429
## group1:month  0.50066    0.89188   0.561
##
## Correlation of Fixed Effects:
##             (Intr) AGE    EDUCAT group1 month
## AGE         -0.900
## EDUCATION   -0.426  0.019
## group1      -0.202  0.162 -0.155
## month        0.021  0.008 -0.002 -0.141
## group1:mnth -0.013 -0.009 -0.005  0.182 -0.824
## P Value for Full vs. Reduced Model
## [1] 0.5752262
```

© 2018 Kennedy RE et al. *JAMA Network Open*.

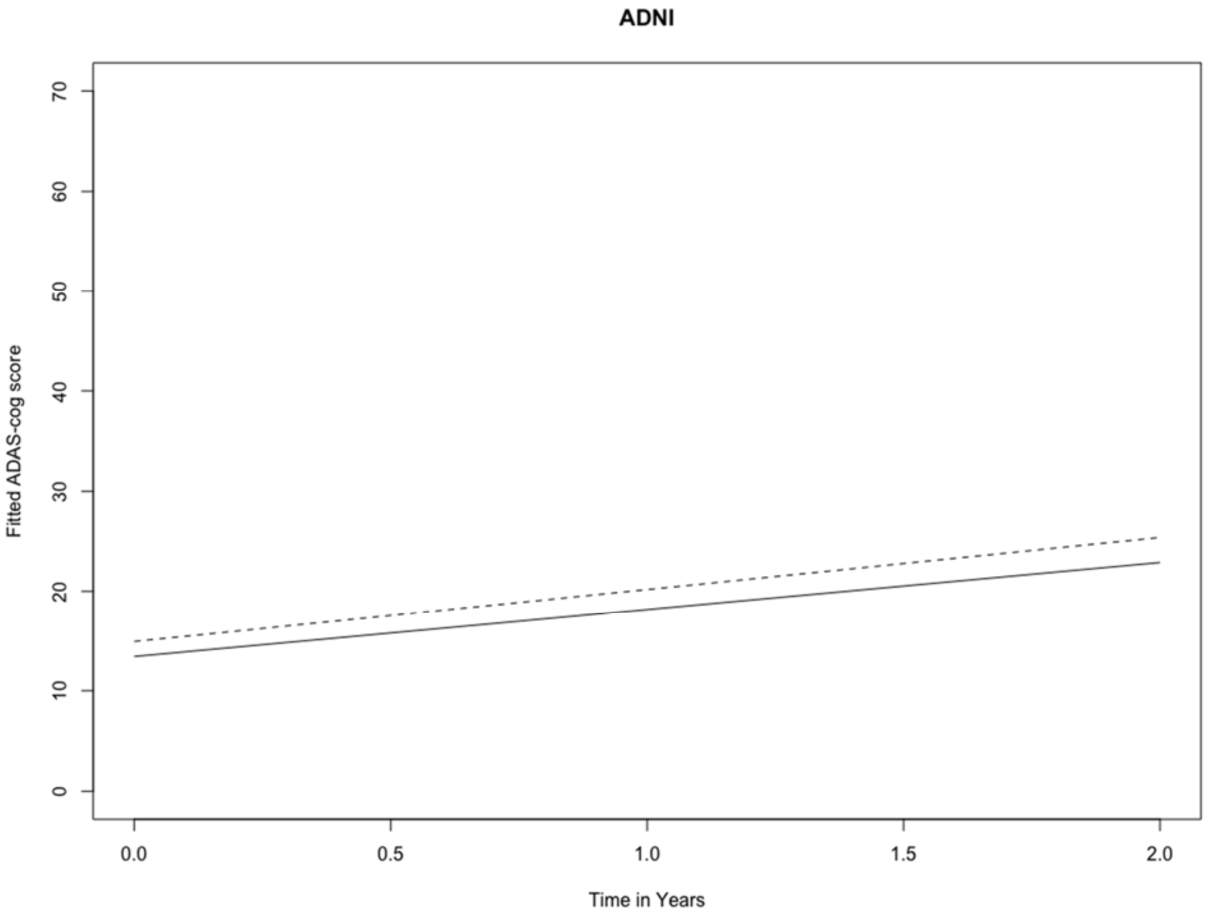


```
## Study Name
## [1] "DHA"
## Full Mixed Effects Model
## Linear mixed model fit by maximum likelihood  ['lmerMod']
## Formula: ADAS ~ AGE + EDUCATION + group + month + group:month + (month |
##     UID)
##    Data: mixed.data
##
##      AIC      BIC   logLik deviance df.resid
##   7545.2   7595.9  -3762.6   7525.2     1170
##
## Scaled residuals:
##      Min       1Q   Median       3Q      Max
## -2.61384 -0.47988  0.00245  0.47883  2.57241
##
## Random effects:
##  Groups   Name        Variance Std.Dev. Corr
##  UID      (Intercept) 57.56    7.587
##           month       22.30    4.722    0.57
##  Residual             10.91    3.302
```

© 2018 Kennedy RE et al. *JAMA Network Open*.

```
## Number of obs: 1180, groups:  UID, 353
##
## Fixed effects:
##               Estimate Std. Error t value
## (Intercept)   15.4286     4.7024   3.281
## AGE            0.1166     0.0496   2.350
## EDUCATION     -0.2601     0.1509  -1.723
## group1         4.9019     0.9590   5.111
## month          3.9729     0.6114   6.498
## group1:month   2.0387     0.7250   2.812
##
## Correlation of Fixed Effects:
##              (Intr) AGE    EDUCAT group1 month
## AGE          -0.881
## EDUCATION    -0.579  0.163
## group1       -0.151  0.048 -0.071
## month         0.054 -0.011  0.000 -0.223
## group1:mnth -0.036  0.003 -0.009  0.260 -0.843
## P Value for Full vs. Reduced Model
## [1] 0.005062576
```

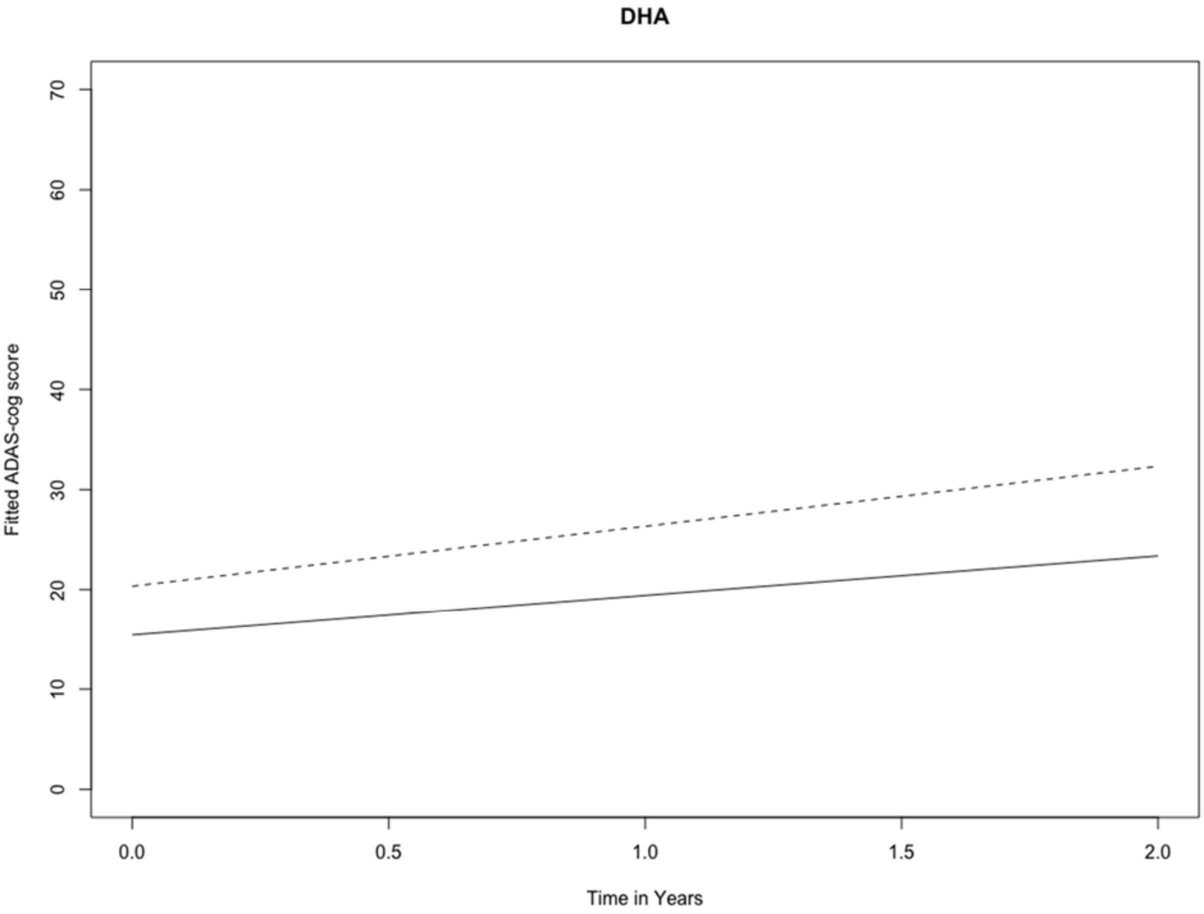

© 2018 Kennedy RE et al. *JAMA Network Open*.

```
## Study Name
## [1] "HC"
## Full Mixed Effects Model
## Linear mixed model fit by maximum likelihood  ['lmerMod']
## Formula: ADAS ~ AGE + EDUCATION + group + month + group:month + (month |
##     UID)
##    Data: mixed.data
##
##      AIC      BIC   logLik deviance df.resid
##   8260.1   8311.6  -4120.0   8240.1     1273
##
## Scaled residuals:
##     Min      1Q  Median      3Q     Max
## -3.1929 -0.5029 -0.0233  0.4700  4.0560
##
## Random effects:
##  Groups   Name        Variance Std.Dev. Corr
##  UID      (Intercept) 54.89    7.409
##           month       16.41    4.051    0.42
##  Residual             13.12    3.622
## Number of obs: 1283, groups:  UID, 361
##
## Fixed effects:
##             Estimate Std. Error t value
## (Intercept)  15.01354    4.88852   3.071
## AGE          0.16131    0.05513   2.926
## EDUCATION    -0.49051    0.14001  -3.503
## group1       4.01666    0.85470   4.699
## month        2.95427    0.43377   6.811
## group1:month  2.76470    0.59132   4.675
##
## Correlation of Fixed Effects:
##            (Intr) AGE    EDUCAT group1 month
## AGE        -0.912
## EDUCATION   -0.480  0.101
## group1      -0.138  0.093 -0.086
## month        0.023 -0.009 -0.008 -0.066
## group1:mnth -0.016  0.005  0.010  0.089 -0.734
## P Value for Full vs. Reduced Model
## [1] 4.473328e-06
```

© 2018 Kennedy RE et al. *JAMA Network Open*.

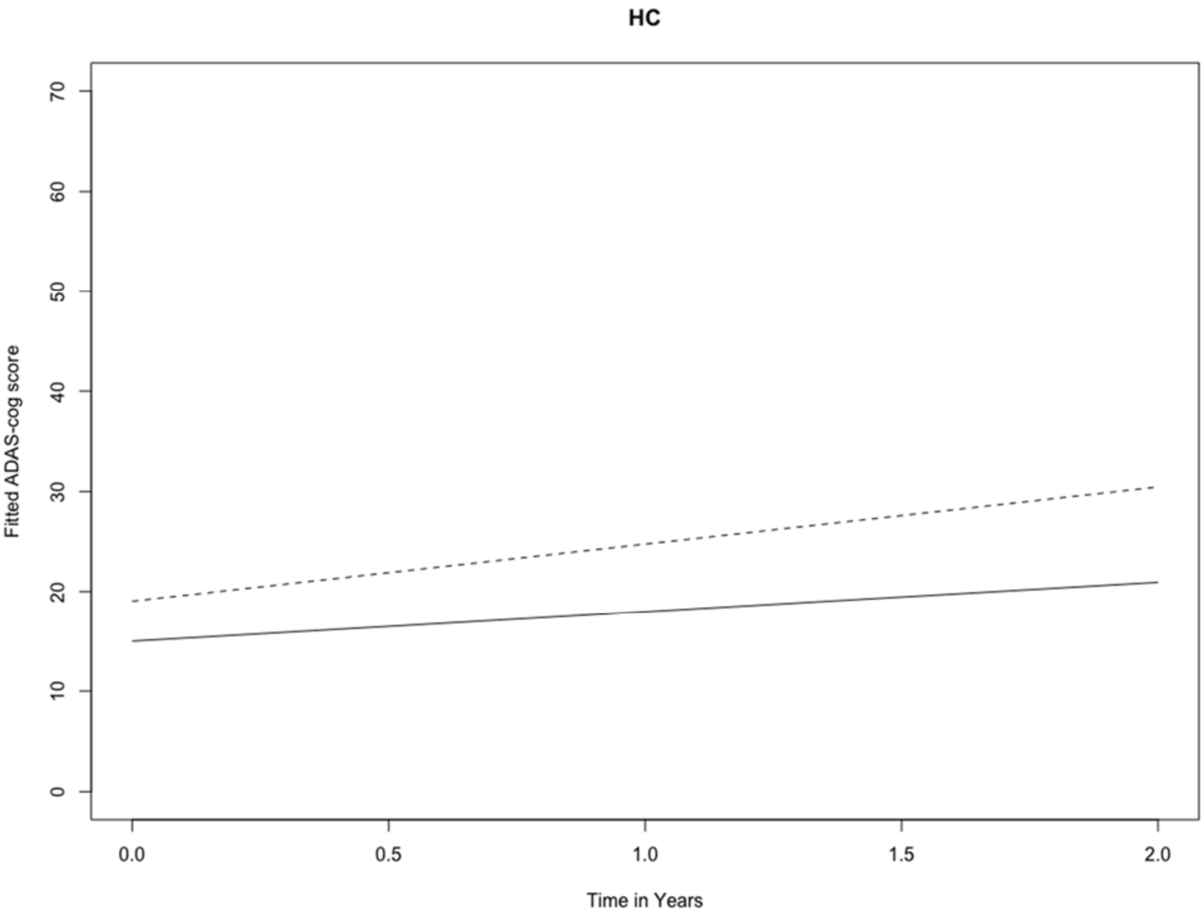


```
## Study Name
## [1] "HU"
## Full Mixed Effects Model
## Linear mixed model fit by maximum likelihood  ['lmerMod']
## Formula: ADAS ~ AGE + EDUCATION + group + month + group:month + (month |
##     UID)
##    Data: mixed.data
##
##      AIC      BIC   logLik deviance df.resid
##   1681.8   1716.9   -830.9   1661.8      236
##
## Scaled residuals:
##      Min       1Q   Median       3Q      Max
## -2.16585 -0.47352 -0.01075  0.48386  2.31685
##
## Random effects:
##  Groups   Name        Variance Std.Dev. Corr
##  UID      (Intercept) 107.423  10.364
##           month         2.608   1.615   0.75
##  Residual              15.910   3.989
```

© 2018 Kennedy RE et al. *JAMA Network Open*.

```
## Number of obs: 246, groups:  UID, 97
##
## Fixed effects:
##              Estimate Std. Error t value
## (Intercept)   52.9841    15.5889   3.399
## AGE           -0.2617     0.1536  -1.703
## EDUCATION     -0.5873     0.3212  -1.828
## group1         4.7030     7.8414   0.600
## month          2.6606     3.3151   0.803
## group1:month  -1.0131     3.3770  -0.300
##
## Correlation of Fixed Effects:
##             (Intr) AGE    EDUCAT group1 month
## AGE         -0.804
## EDUCATION   -0.413  0.107
## group1      -0.532  0.015  0.097
## month        0.006  0.004  0.011 -0.024
## group1:mnth -0.006 -0.004 -0.010  0.024 -0.982
## P Value for Full vs. Reduced Model
## [1] 0.7669275
```

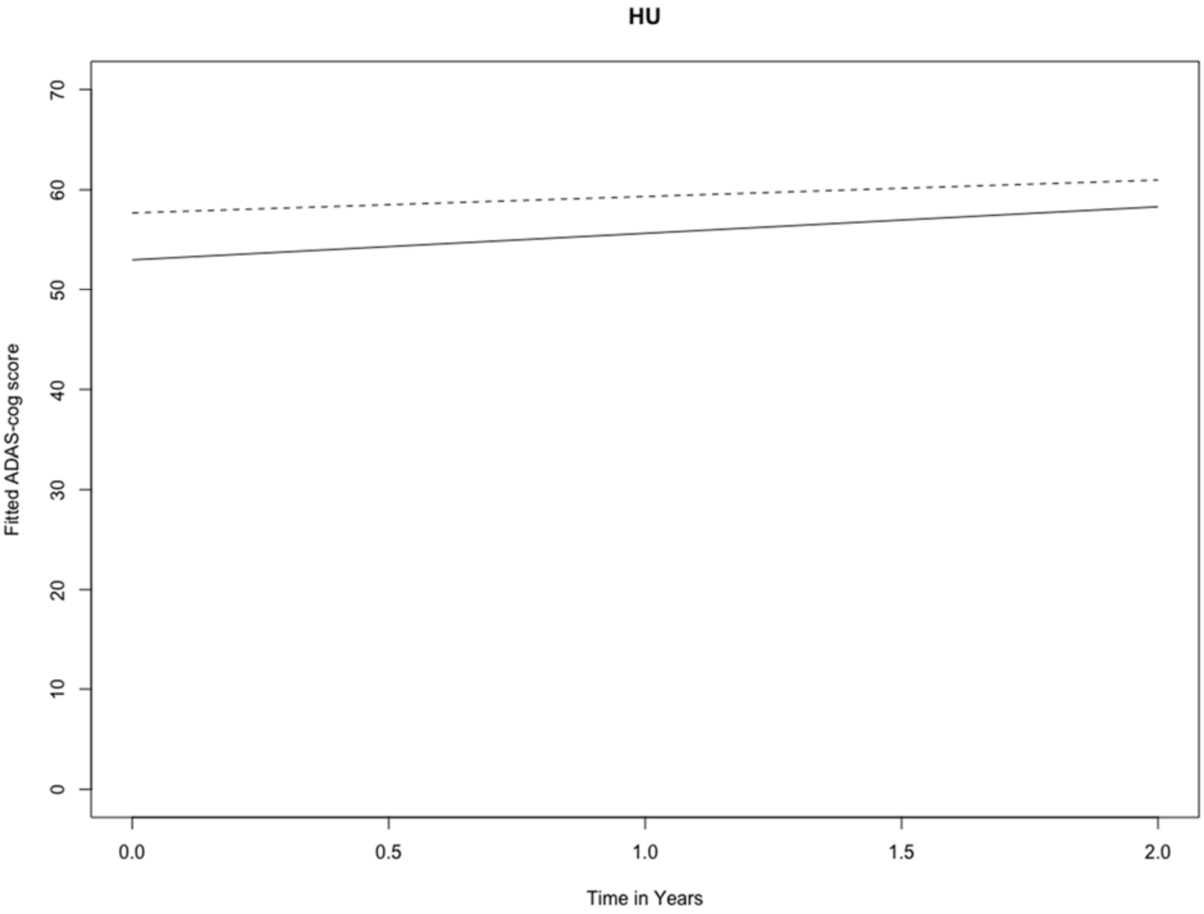

© 2018 Kennedy RE et al. *JAMA Network Open*.

```
## Study Name
## [1] "LL"
## Full Mixed Effects Model
## Linear mixed model fit by maximum likelihood  ['lmerMod']
## Formula: ADAS ~ AGE + EDUCATION + group + month + group:month + (month |
##     UID)
##    Data: mixed.data
##
##      AIC      BIC   logLik deviance df.resid
##   8060.9   8111.9  -4020.4   8040.9     1209
##
## Scaled residuals:
##      Min       1Q   Median       3Q      Max
## -2.45904 -0.48528 -0.00988  0.45778  2.97316
##
## Random effects:
##  Groups   Name        Variance Std.Dev. Corr
##  UID      (Intercept) 82.58    9.087
##           month       18.82    4.339    0.34
##  Residual             13.63    3.692
## Number of obs: 1219, groups:  UID, 373
##
## Fixed effects:
##              Estimate Std. Error t value
## (Intercept)  20.488283   4.829999   4.242
## AGE           0.006948   0.054431   0.128
## EDUCATION     0.010258   0.159901   0.064
## group1        5.056426   1.026693   4.925
## month         3.977156   0.500995   7.939
## group1:month  2.422437   0.657554   3.684
##
## Correlation of Fixed Effects:
##             (Intr) AGE    EDUCAT group1 month
## AGE         -0.878
## EDUCATION   -0.505  0.060
## group1      -0.144  0.123 -0.160
## month        0.014 -0.001 -0.006 -0.049
## group1:mnth -0.010  0.000  0.005  0.066 -0.762
## P Value for Full vs. Reduced Model
## [1] 0.000262101
```

© 2018 Kennedy RE et al. *JAMA Network Open*.

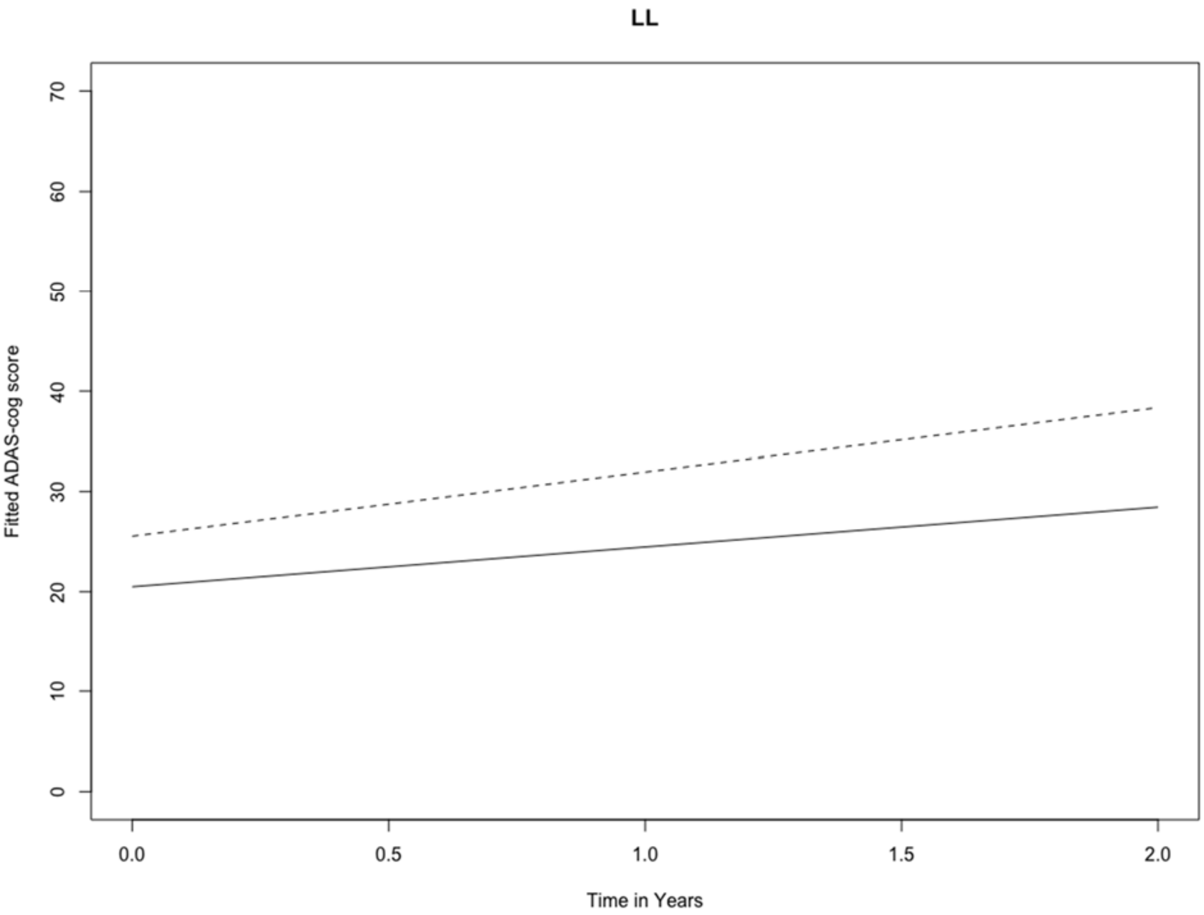


```
## Study Name
## [1] "VN"
## Full Mixed Effects Model
## Linear mixed model fit by maximum likelihood  ['lmerMod']
## Formula: ADAS ~ AGE + EDUCATION + group + month + group:month + (month |
##     UID)
##    Data: mixed.data
##
##      AIC      BIC   logLik deviance df.resid
##   5633.5   5681.0  -2806.8   5613.5      840
##
## Scaled residuals:
##     Min      1Q  Median      3Q     Max
## -2.9715 -0.4960 -0.0157  0.4295  3.9849
##
## Random effects:
##  Groups   Name        Variance Std.Dev. Corr
##  UID      (Intercept) 63.44    7.965
##           month       13.59    3.686    0.32
##  Residual             16.11    4.014
```

© 2018 Kennedy RE et al. *JAMA Network Open*.

```
## Number of obs: 850, groups:  UID, 275
##
## Fixed effects:
##              Estimate Std. Error t value
## (Intercept)  35.33160    5.90774   5.981
## AGE          -0.09639    0.06730  -1.432
## EDUCATION    -0.09911    0.15953  -0.621
## group1        3.32177    1.25067   2.656
## month         5.06683    0.72732   6.966
## group1:month  1.43710    0.84547   1.700
##
## Correlation of Fixed Effects:
##            (Intr) AGE    EDUCAT group1 month
## AGE        -0.922
## EDUCATION  -0.433  0.098
## group1     -0.234  0.149 -0.146
## month       0.002  0.002 -0.003 -0.010
## group1:mnth -0.002 -0.002  0.006  0.009 -0.860
## P Value for Full vs. Reduced Model
## [1] 0.0932488
```

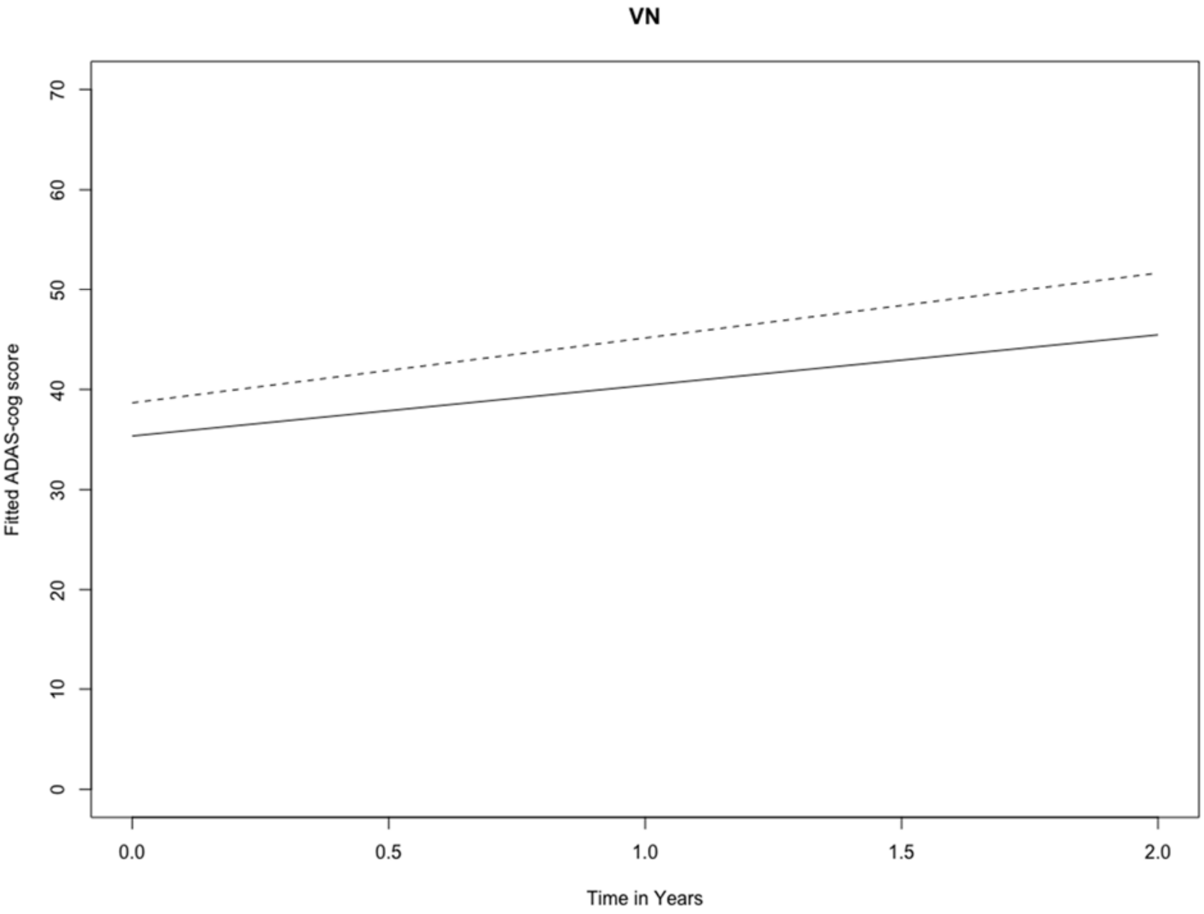

© 2018 Kennedy RE et al. *JAMA Network Open*.

```
##
## Mixed-Effects Model (k = 6; tau^2 estimator: REML)
##
## tau^2 (estimated amount of residual heterogeneity):     0.0000 (SE = 0.4241)
## tau (square root of estimated tau^2 value):             0.0008
## I^2 (residual heterogeneity / unaccounted variability): 0.00%
## H^2 (unaccounted variability / sampling variability):   1.00
## R^2 (amount of heterogeneity accounted for):            100.00%
##
## Test for Residual Heterogeneity:
## QE(df = 4) = 3.5783, p-val = 0.4661
##
## Test of Moderators (coefficient(s) 2):
## QM(df = 1) = 2.5658, p-val = 0.1092
##
## Model Results:
##
##          estimate      se     zval    pval    ci.lb   ci.ub
## intrcpt    5.4700  2.1707   2.5199  0.0117   1.2155  9.7245  *
## mods      -0.0585  0.0365  -1.6018  0.1092  -0.1300  0.0131
##
## ---
## Signif. codes:  0 '***' 0.001 '**' 0.01 '*' 0.05 '.' 0.1 ' ' 1
```

© 2018 Kennedy RE et al. *JAMA Network Open*.

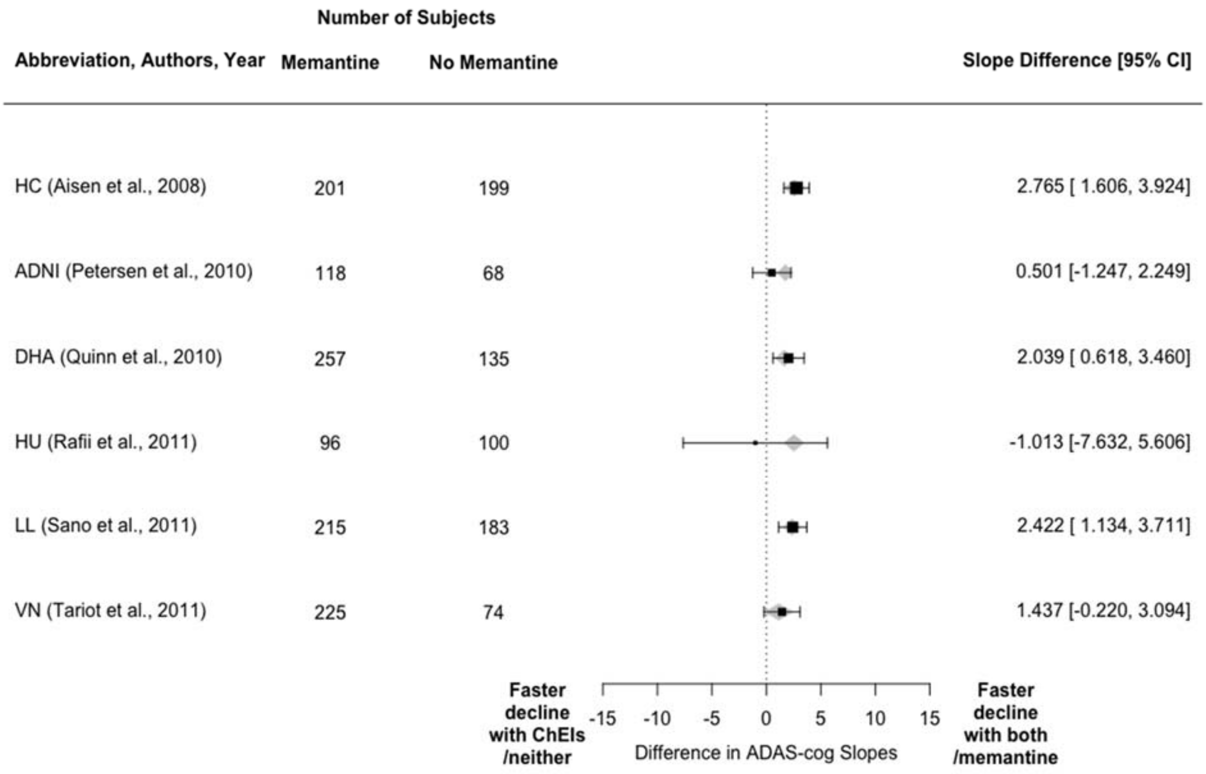

© 2018 Kennedy RE et al. *JAMA Network Open*.

Source Code

```
library(knitr)
opts_chunk$set(fig.path = 'figure/', message=FALSE, warning=FALSE, dev=c('png','post
script'), fig.width=11, fig.height=8.5)
library(knitcitations); cleanbib()
cite_options(citation_format = "pandoc", check.entries=FALSE)
library(bibtex)
# load libraries required for analysis
library(compareGroups)
library(car)
library(metafor)
library(lme4)
library(EMAtools)
library(calibrate)
library(Hmisc)

cohensd.ci <- function(d, n1, n2, ci = 0.95) {
  library(compute.es)
  t <- d * sqrt((n1 * n2)/(n1 + n2))
  capture.output(
    fit <- compute.es::tes(t = t, n.1 = n1, n.2 = n2, level = 100 * ci),
    file = "NUL"
  )
  c(lower.ci = fit$l.d, upper.ci = fit$u.d)
}

setwd("/Users/richardkennedy/Documents/ADCS")
adasna.strings=c('-4.0','-1.0',", '-1','-2','-3', '-4','-5','NA',")

study.names <- c("ADNI", "AP", "DHA", "EIN", "ES", "HC", "HU", "LL", "MCI", "ML
", "NL", "NS", "PI", "PIWEB", "PR", "SIN", "SL", "VN", "VP")
study.authors <- c("Petersen et al.", "Teri et al.", "Quinn et al.", "Ferris et al.", "Mulnard
et al.", "Aisen et al.", "Rafii et al.","Sano et al.", "Petersen et al.", "Singer et al.", "Normal
Controls", "Aisen et al.", "Ferris et al.", "Walsh et al.", "Aisen et al.", "Sano et al.", "Sano
et al.", "Tariot et al.", "Tariot et al.")
study.refno <- c("2", "", "15", "", "12", "14", "16", "17", "", "", "", "13", "", "", "11", "", "
10", "18", "")
names(study.authors) <- study.names
names(study.refno) <- study.names
# by year of publication
#study.years <- c("2010", "2000", "2010", "1997", "2000", "2008", "2011", "2011", "200
5", "2003", "in progress", "2003", "2006", "2006", "2000", "1997a", "1997", "2011", "20
05")
# by year of enrollment
study.years <- c("2005", "1994", "2006", "1993", "1995", "2003", "2004", "2003", "1999
", "1997", "1999", "1999", "2002", "2002", "1995", "1993", "1993", "2003", "2000")
```

© 2018 Kennedy RE et al. *JAMA Network Open*.

```
names(study.years) <- study.names
study.pubyears <- c(2010, 2000, 2010, 1997, 2000, 2008, 2011, 2011, 2005, 2003, 2016,
2003, 2006, 2006, 2000, 1997, 1997, 2011, 2005)
names(study.pubyears) <- study.names
study.relyears <- study.pubyears - min(study.pubyears)
names(study.relyears) <- study.names

codes <-  c("sc", "bl", "m01", "m02", "m03", "m06", "m09", "m12", "m15", "m18", "m20
", "m21", "m24", "m30", "m36", "m48", "sc", "w04", "w06", "w08", "w11", "w12", "w16
", "w20", "w24", "w28", "w36", "w44", "w48", "w52", "w60", "w68", "w72")
new.codes <- c("sc", "bl", "m01", "m02", "m03", "m06", "m09", "m12", "m15", "m18", "
m20", "m21", "m24", "m30", "m36", "m48", "sc", "m01", "m01", "m02", "m03", "m03",
"m04", "m05", "m06", "m06", "m09", "m11", "m12", "m12", "m15", "m18", "m18")
months <- c(0, 0, 1, 2, 3, 6, 9, 12, 15, 18, 20, 21, 24, 30, 36, 48, 0, 1, 2, 2, 3, 3, 4, 5, 6, 7,
9, 11, 12, 12, 15, 17, 18)
names(months) <- codes
placebo.codes <- c("1", "2", "3", "4", "5", "6", "7", "8", "9", "Behavior", "Clinic", "Home
", "Placebo", "Web")

drug.codes <-  c("0.625mg", "1.25mg", "Active", "Alpha-Tocopherol", "DHA", "Donepe
zil", "Haloperidol", "HU200ug", "HU400ug", "ML 10", "ML 2.5SR", "Naproxen", "PR",
"Rofecoxib", "Selegiline", "Selegiline_and_Alpha-Tocopherol", "Trazodone", "Valproate
", "Vitamin B", "Vitamin E")

drug.studies <- c("SL", "AP", "PR", "ES", "ML", "MCI", "NS", "VP", "LL", "HC", "VN"
, "HU", "AX", "DHA")

adas.studies <- c("DHA", "ES", "HC", "HU", "LL", "MCI", "ML", "NL", "NS", "PR", "S
L", "VN", "ADNI")

adas.ad.studies <- c("DHA", "ES", "HC", "HU", "LL", "ML", "NS", "PR", "SL", "VN", "
ADNI")

chei.studies <- c("DHA", "ES", "HC", "HU", "LL", "ML", "NS", "PR", "SL", "VN", "AD
NI")

memantine.studies <- c("DHA", "HC", "HU", "LL", "VN", "ADNI")

adasscores <- read.table(file="adcs-adasscores-data-2013-09-24.csv", sep=",", header=T
RUE, stringsAsFactors=FALSE, na.strings=adasna.strings)
demog <- read.table(file="adcs-demog-data-2012-06-12.csv", sep=",", header=TRUE, st
ringsAsFactors=TRUE, na.strings=adasna.strings)
arm <- read.table(file="adcs-arm-data-2012-05-30.csv", sep=",", header=TRUE, strings
AsFactors=FALSE, na.strings=adasna.strings)
cdr <- read.table(file="adcs-cdr-data-2012-06-11.csv", sep=",", header=TRUE, stringsA
sFactors=FALSE, na.strings=adasna.strings)
```

© 2018 Kennedy RE et al. *JAMA Network Open*.

```r
setwd("/Users/richardkennedy/Documents/Schneider/ChEI")
# medications
medhist <- read.csv("adcs-conmeds-data-revised-2012-11-21modified.csv", na.strings="-
1", stringsAsFactors=FALSE, allowEscapes=TRUE)
temp <- ifelse(medhist$CMMED=="", medhist$GNAME, medhist$CMMED)
temp <- toupper(temp)
temp <- replace(temp, temp=="ARICEFT", "DONEPEZIL")
temp <- replace(temp, temp=="ARICET", "DONEPEZIL")
temp <- replace(temp, temp=="ARICIEPT", "DONEPEZIL")
temp <- replace(temp, temp=="ARICIPT", "DONEPEZIL")
temp <- replace(temp, temp=="ARICPET", "DONEPEZIL")
temp <- replace(temp, temp=="ARICEPT", "DONEPEZIL")
temp <- replace(temp, temp=="ARICEPT (DONEPEZIL)", "DONEPEZIL")
temp <- replace(temp, temp=="ARICEPT 10", "DONEPEZIL")
temp <- replace(temp, temp=="ARICEPT 10MG", "DONEPEZIL")
temp <- replace(temp, temp=="ARICEPT 5MG", "DONEPEZIL")
temp <- replace(temp, temp=="DONEPEZIL", "DONEPEZIL")
temp <- replace(temp, temp=="DONEPEZIL (ARICEPT)", "DONEPEZIL")
temp <- replace(temp, temp=="DONEPEZIL HCL", "DONEPEZIL")
temp <- replace(temp, temp=="DONEPEZIL (DONEPEZIL)", "DONEPEZIL")
temp <- replace(temp, temp=="DONEPEZIL 10", "DONEPEZIL")
temp <- replace(temp, temp=="DONEPEZIL 10MG", "DONEPEZIL")
temp <- replace(temp, temp=="DONEPEZIL 5MG", "DONEPEZIL")
temp <- replace(temp, temp=="DONEPEZIL HCL", "DONEPEZIL")
temp <- replace(temp, temp=="DONEPEZIL", "DONEPEZIL")
temp <- replace(temp, temp=="DONEPEZIL (DONEPZIL)", "DONEPEZIL")
temp <- replace(temp, temp=="DONEPEZIL HCI", "DONEPEZIL")
temp <- replace(temp, temp=="DONEPEZIL HCI (DONEPZIL)", "DONEPEZIL")
temp <- replace(temp, temp=="DONEPEZIL HCL", "DONEPEZIL")
temp <- replace(temp, temp=="DONEPEZIL HCL (DONEPZIL)", "DONEPEZIL")
temp <- replace(temp, temp=="DONEPEZIL HYDROCHLORIDE", "DONEPEZIL")
temp <- replace(temp, temp=="DONEPEZL HCL", "DONEPEZIL")
temp <- replace(temp, temp=="DONEPIZIL", "DONEPEZIL")
temp <- replace(temp, temp=="DONEPIZIL HCL", "DONEPEZIL")
temp <- replace(temp, temp=="DONEPZIL", "DONEPEZIL")
temp <- replace(temp, temp=="DONEPZIL (DONEPEZIL)", "DONEPEZIL")
temp <- replace(temp, temp=="DONEPZIL 10", "DONEPEZIL")
temp <- replace(temp, temp=="DONEPZIL 10MG", "DONEPEZIL")
temp <- replace(temp, temp=="DONEPZIL 5MG", "DONEPEZIL")
temp <- replace(temp, temp=="DONEZEPIL", "DONEPEZIL")
temp <- replace(temp, temp=="DOREPZEIL HCL", "DONEPEZIL")
temp <- replace(temp, temp=="E2020", "TACRINE")
temp <- replace(temp, temp=="EXELON", "RIVASTIGMINE")
temp <- replace(temp, temp=="EXELON - TRANSDERMAL PATCH", "RIVASTIGMI
NE")
```

© 2018 Kennedy RE et al. *JAMA Network Open*.

```
temp <- replace(temp, temp=="EXELON (RIVASTIGMINE)", "RIVASTIGMINE")
temp <- replace(temp, temp=="EXELON 10CM2", "RIVASTIGMINE")
temp <- replace(temp, temp=="EXELON 6MG", "RIVASTIGMINE")
temp <- replace(temp, temp=="EXELON PARCH", "RIVASTIGMINE")
temp <- replace(temp, temp=="EXELON PATCH", "RIVASTIGMINE")
temp <- replace(temp, temp=="GALANTAMINE", "GALANTAMINE")
temp <- replace(temp, temp=="GALANTAMINE ER", "GALANTAMINE")
temp <- replace(temp, temp=="GALANTAMINE HYDROBROMIDE", "GALANTAM
INE")
temp <- replace(temp, temp=="GALANTAMINE", "GALANTAMINE")
temp <- replace(temp, temp=="GALANTAMINE (GALANTAMINEE)", "GALANTA
MINE")
temp <- replace(temp, temp=="GALANTAMINE 8MG", "GALANTAMINE")
temp <- replace(temp, temp=="GALANTAMINE ER", "GALANTAMINE")
temp <- replace(temp, temp=="GALANTAMINE SR", "GALANTAMINE")
temp <- replace(temp, temp=="GALANTAMINEE", "GALANTAMINE")
temp <- replace(temp, temp=="GALANTAMINEE ER", "GALANTAMINE")
temp <- replace(temp, temp=="GALANTAMINEE GALANTAMINE", "GALANTAMI
NE")
temp <- replace(temp, temp=="GALANTAMINEE SR", "GALANTAMINE")
temp <- replace(temp, temp=="GALANTAMINEE/GALANTAMINE", "GALANTAMI
NE")
temp <- replace(temp, temp=="GALANTAMINEEL", "GALANTAMINE")
temp <- replace(temp, temp=="MEMANTIME", "MEMANTINE")
temp <- replace(temp, temp=="MEMANTINE", "MEMANTINE")
temp <- replace(temp, temp=="MEMANTINE (NAMENDA)", "MEMANTINE")
temp <- replace(temp, temp=="MEMANTINE HCL", "MEMANTINE")
temp <- replace(temp, temp=="MEMANTINE HCL (NAMENDA)", "MEMANTINE")
temp <- replace(temp, temp=="MEMANTINE HYDROCHLORIDE", "MEMANTINE"
)
temp <- replace(temp, temp=="MEMATINE", "MEMANTINE")
temp <- replace(temp, temp=="MEMENTINE", "MEMANTINE")
temp <- replace(temp, temp=="MEMONTINE", "MEMANTINE")
temp <- replace(temp, temp=="MENANTINE", "MEMANTINE")
temp <- replace(temp, temp=="MENMANTINE", "MEMANTINE")
temp <- replace(temp, temp=="METRIFONATE", "METRIFONATE")
temp <- replace(temp, temp=="NAMEDA", "MEMANTINE")
temp <- replace(temp, temp=="NAMENDA", "MEMANTINE")
temp <- replace(temp, temp=="NAMENDA (MEMANTINE)", "MEMANTINE")
temp <- replace(temp, temp=="NAMENDA 10", "MEMANTINE")
temp <- replace(temp, temp=="NAMENDA HCL", "MEMANTINE")
temp <- replace(temp, temp=="NAMENDA STARTER KIT", "MEMANTINE")
temp <- replace(temp, temp=="NAMERDA", "MEMANTINE")
temp <- replace(temp, temp=="NEMANDA", "MEMANTINE")
temp <- replace(temp, temp=="NEMENDA", "MEMANTINE")
temp <- replace(temp, temp=="NOMENDA", "MEMANTINE")
```

© 2018 Kennedy RE et al. *JAMA Network Open*.

```
temp <- replace(temp, temp=="PRICEPT", "DONEPEZIL")
temp <- replace(temp, temp=="RAZADINE", "GALANTAMINE")
temp <- replace(temp, temp=="RAZADYN", "GALANTAMINE")
temp <- replace(temp, temp=="RAZADYN ER", "GALANTAMINE")
temp <- replace(temp, temp=="RAZADYNE", "GALANTAMINE")
temp <- replace(temp, temp=="RAZADYNE ER", "GALANTAMINE")
temp <- replace(temp, temp=="RAZADYNE REMINYL", "GALANTAMINE")
temp <- replace(temp, temp=="RAZADYNE SR", "GALANTAMINE")
temp <- replace(temp, temp=="RAZADYNE/REMINYL", "GALANTAMINE")
temp <- replace(temp, temp=="RAZADYNEL", "GALANTAMINE")
temp <- replace(temp, temp=="RAZDYNE", "GALANTAMINE")
temp <- replace(temp, temp=="RAZDYNE SR", "GALANTAMINE")
temp <- replace(temp, temp=="RAZYDYNE", "GALANTAMINE")
temp <- replace(temp, temp=="RAZYDYNE ER", "GALANTAMINE")
temp <- replace(temp, temp=="REMENYL", "GALANTAMINE")
temp <- replace(temp, temp=="REMERYL", "GALANTAMINE")
temp <- replace(temp, temp=="REMINOL", "GALANTAMINE")
temp <- replace(temp, temp=="REMINYL", "GALANTAMINE")
temp <- replace(temp, temp=="REMINYL (RAZADYNE)", "GALANTAMINE")
temp <- replace(temp, temp=="REMINYL 8MG", "GALANTAMINE")
temp <- replace(temp, temp=="REMINYL ER", "GALANTAMINE")
temp <- replace(temp, temp=="RENEDIL", "GALANTAMINE")
temp <- replace(temp, temp=="REVISTAGMINE", "RIVASTIGMINE")
temp <- replace(temp, temp=="REZADYNE", "GALANTAMINE")
temp <- replace(temp, temp=="RIVASTIGINE", "RIVASTIGMINE")
temp <- replace(temp, temp=="RIVASTIGMINE", "RIVASTIGMINE")
temp <- replace(temp, temp=="RIVASTIGMINE", "RIVASTIGMINE")
temp <- replace(temp, temp=="RIVASTIGMINE HYDROGEN TARTRATE", "RIVAS
TIGMINE")
temp <- replace(temp, temp=="ROZADYNE", "GALANTAMINE")
memantine <- ifelse(temp=="MEMANTINE", 1, 0)
memantine <- tapply(memantine, medhist$UID, max)
#idx <- unique(medhist$UID)
memantine <- memantine[medhist$UID]
#memantine <- rep(memantine, times=table(medhist$UID))
medhist$memantine <- memantine
chei <- ifelse(temp=="DONEPEZIL" | temp=="GALANTAMINE" | temp=="RIVASTI
GMINE" | temp=="METRIFONATE" | temp=="TACRINE", 1, 0)
chei <- tapply(chei, medhist$UID, max)
#idx <- sort(unique(medhist$UID))
chei <- chei[medhist$UID]
#chei <- rep(chei, times=table(medhist$UID))
medhist$chei <- chei

donepezil <- ifelse(temp=="DONEPEZIL", 1, 0)
donepezil <- tapply(donepezil, medhist$UID, max)
```

© 2018 Kennedy RE et al. *JAMA Network Open*.

```
donepezil <- donepezil[medhist$UID]
medhist$donepezil <- donepezil

galantamine <- ifelse(temp=="GALANTAMINE", 1, 0)
galantamine <- tapply(galantamine, medhist$UID, max)
galantamine <- galantamine[medhist$UID]
medhist$galantamine <- galantamine

rivastigmine <- ifelse(temp=="RIVASTIGMINE", 1, 0)
rivastigmine <- tapply(rivastigmine, medhist$UID, max)
rivastigmine <- rivastigmine[medhist$UID]
medhist$rivastigmine <- rivastigmine

metrifonate <- ifelse(temp=="METRIFONATE", 1, 0)
metrifonate <- tapply(metrifonate, medhist$UID, max)
metrifonate <- metrifonate[medhist$UID]
medhist$metrifonate <- metrifonate

tacrine <- ifelse(temp=="TACRINE", 1, 0)
tacrine <- tapply(tacrine, medhist$UID, max)
tacrine <- tacrine[medhist$UID]
medhist$tacrine <- tacrine

medhist$group4 <- ifelse(medhist$memantine==1, ifelse(medhist$chei==1,"Both", "Me
mantine"), ifelse(medhist$chei==1, "ChEI", "Neither"))
medhist$group <- NA
idx <- medhist$group4=="Neither"
idx[is.na(idx)] <- FALSE
medhist$group[idx] <- 0
idx <- medhist$group4=="Both" | medhist$group4=="Memantine" | medhist$group4=="
ChEI"
idx[is.na(idx)] <- FALSE
medhist$group[idx] <- 1

all.data <- merge(adasscores, arm, by=c("UID", "METAID", "STUDY", "CENTNO", "R
ID"), all.x=TRUE, all.y=TRUE, suffixes=c("",".arm"))
all.data <- merge(all.data, demog, by=c("UID", "METAID", "STUDY", "CENTNO", "R
ID"), all.x=TRUE, all.y=TRUE, suffixes=c("",".demog"))

data.cols <- c("UID", "memantine", "chei", "donepezil", "galantamine", "rivastigmine", "
metrifonate", "tacrine", "group4", "group")
temp <- medhist[,data.cols]
idx <- !duplicated(temp$UID)
temp <- temp[idx,]
all.data <- merge(all.data, temp, by="UID", all.x=TRUE, all.y=FALSE)
```

© 2018 Kennedy RE et al. *JAMA Network Open*.

```
all.data$TX <- NA
idx <- all.data$ARM %in% placebo.codes
all.data$TX[idx] <- "Placebo"
idx <- all.data$ARM %in% drug.codes
all.data$TX[idx] <- "Drug"

all.data$SEVERITY <- NA

idx <- all.data$ARM=="1" | all.data$ARM=="4" | all.data$ARM=="7" | all.data$STUD
Y=="NL"
all.data$SEVERITY[idx] <- "NL"
idx <- cdr$STUDY=="EIN" & cdr$CDGLOBAL==0 & cdr$VISCODE=="sc"
temp <- unique(cdr[idx,"UID"])
idx <- all.data$UID %in% temp
all.data$SEVERITY[idx] <- "NL"
idx <- cdr$STUDY=="SIN" & cdr$CDGLOBAL==0 & cdr$VISCODE=="sc"
temp <- unique(cdr[idx,"UID"])
idx <- all.data$UID %in% temp
all.data$SEVERITY[idx] <- "NL"
idx <- cdr$STUDY=="PI" & cdr$CDGLOBAL==0 & cdr$VISCODE=="sc"
temp <- unique(cdr[idx,"UID"])
idx <- all.data$UID %in% temp
all.data$SEVERITY[idx] <- "NL"
idx <- cdr$STUDY=="PIWEB" & cdr$CDGLOBAL==0 & cdr$VISCODE=="sc"
temp <- unique(cdr[idx,"UID"])
idx <- all.data$UID %in% temp
all.data$SEVERITY[idx] <- "NL"
idx <-  all.data$ARM=="2" | all.data$ARM=="5" | all.data$ARM=="8" | all.data$STUD
Y=="MCI" | all.data$STUDY=="SIN"
all.data$SEVERITY[idx] <- "MCI"
idx <- cdr$STUDY=="PI" & cdr$CDGLOBAL==0.5 & cdr$VISCODE=="sc"
temp <- unique(cdr[idx,"UID"])
idx <- all.data$UID %in% temp
all.data$SEVERITY[idx] <- "MCI"
idx <- cdr$STUDY=="PIWEB" & cdr$CDGLOBAL==0.5 & cdr$VISCODE=="sc"
temp <- unique(cdr[idx,"UID"])
idx <- all.data$UID %in% temp
all.data$SEVERITY[idx] <- "MCI"
idx <- all.data$ARM=="3" | all.data$ARM=="6" | all.data$ARM=="9" | all.data$STUD
Y=="SL" | all.data$STUDY=="AP" | all.data$STUDY=="PR" | all.data$STUDY=="ES"
| all.data$STUDY=="ML" | all.data$STUDY=="NS" | all.data$STUDY=="VP" | all.data
$STUDY=="LL" | all.data$STUDY=="HC" | all.data$STUDY=="VN" | all.data$STUD
Y=="HU" | all.data$STUDY=="AX" | all.data$STUDY=="DHA"
all.data$SEVERITY[idx] <- "AD"
idx <- cdr$STUDY=="EIN" & cdr$CDGLOBAL >= 1 & cdr$VISCODE=="sc"
temp <- unique(cdr[idx,"UID"])
```

© 2018 Kennedy RE et al. *JAMA Network Open*.

```
idx <- all.data$UID %in% temp
all.data$SEVERITY[idx] <- "AD"
idx <- cdr$STUDY=="SIN" & cdr$CDGLOBAL >= 1 & cdr$VISCODE=="sc"
temp <- unique(cdr[idx,"UID"])
idx <- all.data$UID %in% temp
all.data$SEVERITY[idx] <- "AD"

all.data$group1 <- paste(all.data$STUDY, all.data$SEVERITY, sep="-")
all.data$group1 <- as.factor(all.data$group1)
all.data$group2 <- paste(all.data$STUDY, all.data$SEVERITY, all.data$TX, sep="-")
all.data$group2 <- as.factor(all.data$group2)

all.data$MARITAL <- replace(all.data$MARITAL, all.data$MARITAL != 1, 2)
all.data$RACE <- replace(all.data$RACE, all.data$RACE != 5, 2)
all.data$RACE <- replace(all.data$RACE, all.data$RACE == 5, 1)
all.data$ETHNIC <- replace(all.data$ETHNIC, all.data$ETHNIC != 1, 2)

all.data$MONTH <- months[as.character(all.data[,"VISCODE"])]
all.data$MONTH <- as.factor(all.data$MONTH)
#all.data$STUDY <- as.factor(all.data$STUDY)
all.data$EDCAT <- cut(all.data$EDUCATION, c(0, 12, 16, 25), right=FALSE, labels=c(
"Less than High School", "High School graduate", "College graduate"))
all.data$ETHNIC <- factor(all.data$ETHNIC, labels=c("Hispanic", "Not Hispanic"))
all.data$MARITAL <- factor(all.data$MARITAL, labels=c("Married", "Not Married"))
all.data$RACE <- factor(all.data$RACE, labels=c("White", "Nonwhite"))
all.data$SEX <- factor(all.data$SEX, labels=c("Male","Female"))
all.data$TX <- factor(all.data$TX, levels=c("Placebo", "Drug"))

idx <- is.na(all.data$SEVERITY)
idx[is.na(idx)] <- FALSE
all.data <- all.data[!idx,]

data.cols <- c("UID", "METAID", "STUDY", "CENTNO", "RID", "SITEID", "TOTAL1
1", "VISCODE", "ARM", "AGE", "BIRTHDATE", "EDCAT", "EDUCATION", "ETHN
IC", "EXAMDATE", "MARITAL", "RACE", "SEX", "TX", "SEVERITY", "group", "gr
oup1", "group2", "group4", "donepezil", "galantamine", "rivastigmine", "metrifonate", "ta
crine")
all.data <- all.data[,data.cols]
data.cols <- c("UID", "METAID", "STUDY", "CENTNO", "RID", "SITEID", "ADAS", "
VISCODE", "ARM", "AGE", "BIRTHDATE", "EDCAT", "EDUCATION", "ETHNIC",
"EXAMDATE", "MARITAL", "RACE", "SEX", "TX", "SEVERITY", "group", "group1
", "group2", "group4", "donepezil", "galantamine", "rivastigmine", "metrifonate", "tacrine
")
colnames(all.data) <- data.cols
temp <- all.data$VISCODE
for (i in 1:length(codes)) {
```

© 2018 Kennedy RE et al. *JAMA Network Open*.

```
    idx <- temp==codes[i]
    temp[idx] <- new.codes[i]
}
all.data$VISCODE <- temp
#study.months <- c("sc", "bl", "m06", "m12", "m18", "m24", "w28", "w52")
study.months <- c("sc", "bl", "m01", "m02", "m03", "m06", "m09", "m11", "m12", "m15
", "m17", "m18", "m20", "m21", "m24", "m30", "m36", "m48")
#idx <- all.data[,"VISCODE"] %in% study.months
#all.data <- all.data[idx,]
#all.data$VISCODE <- as.factor(all.data$VISCODE)
#print(length(unique(all.data$UID)))

all.data.long <- all.data
temp <- reshape(all.data, v.names="ADAS", timevar="VISCODE", idvar="UID", directi
on="wide", sep="_")
idx <- is.na(temp$ADAS_sc)
idx[is.na(idx)] <- FALSE
temp$ADAS_sc[idx] <- temp$ADAS_bl[idx]
idx <- is.na(temp$ADAS_bl)
idx[is.na(idx)] <- FALSE
temp$ADAS_bl[idx] <- temp$ADAS_sc[idx]

idx <- c("UID", "METAID", "STUDY", "CENTNO", "RID", "SITEID", "ARM", "AGE"
, "BIRTHDATE", "EDCAT", "EDUCATION", "ETHNIC", "EXAMDATE", "MARITA
L", "RACE", "SEX", "TX", "SEVERITY", "group", "group1", "group2", "group4", "done
pezil", "galantamine", "rivastigmine", "metrifonate", "tacrine", "ADAS_bl", "ADAS_m0
6", "ADAS_m12", "ADAS_m18", "ADAS_m24")
temp <- temp[,idx]
all.data <- temp
all.data[,"EDCAT"] <- cut(all.data[,"EDUCATION"], c(0, 12, 16, 25), right=FALSE, lab
els=c("Less than HS", "HS graduate", "College graduate"))

idx <- all.data$SEVERITY=="AD"
all.ad <- all.data[idx,]
idx <- all.data$SEVERITY=="MCI"
all.mci <- all.data[idx,]
idx <- all.data$SEVERITY=="NL"
all.nl <- all.data[idx,]
idx <- all.data$SEVERITY=="AD"
all.ad <- all.data[idx,]
idx <- !duplicated(all.ad$UID)
all.ad <- all.ad[idx,]
idx <- !is.na(all.ad$STUDY)
all.ad <- all.ad[idx,]
idx <- !is.na(all.ad$ADAS_bl)
all.ad <- all.ad[idx,]
```

© 2018 Kennedy RE et al. *JAMA Network Open*.

```
data.cols <- c("UID", "STUDY", "AGE", "EDUCATION", "ADAS_bl", "ADAS_m06",
"ADAS_m12", "ADAS_m18", "ADAS_m24", "group", "group4")
temp <- reshape(all.ad[,data.cols], idvar = "UID", varying = c("ADAS_bl", "ADAS_m0
6", "ADAS_m12", "ADAS_m18", "ADAS_m24"), direction = "long", sep="_")
temp$month <- recode(temp$time, "'bl'=0;'m06'=6;'m12'=12;'m18'=18;'m24'=24")

idx <- !is.na(temp$group)
temp <- temp[idx,]

idx <- order(temp$UID, temp$month)
temp <- temp[idx,]

idx <- !is.na(temp$ADAS)
temp <- temp[idx,]

sample.mixed <- temp
results <- NULL
study.ids <- NULL
for (i in unique(sample.mixed$STUDY)) {
  study.name <- i
  writeLines("Study Name")
  print(study.name)
  idx <- sample.mixed$STUDY==study.name
  mixed.data <- sample.mixed[idx,]

  mixed.data$month <- as.numeric(mixed.data$month)
  mixed.data$group <- as.factor(mixed.data$group)
  mixed.data$month <- mixed.data$month / 12
  idx <- order(mixed.data$UID, mixed.data$month)
  mixed.data <- mixed.data[idx,]
  mixed.full <- try(lmer(ADAS ~ AGE + EDUCATION + group + month + group:month
+ (month|UID), REML=FALSE, data=mixed.data))
  mixed.reduced <- try(lmer(ADAS ~ AGE + EDUCATION + group + month + (month|
UID), REML=FALSE, data=mixed.data))

  if ((class(mixed.full) == "try-error") | (class(mixed.reduced) == "try-error")) {
    result <- NA
  } else {
    study.ids <- c(study.ids, as.character(model.frame(mixed.full)$UID))
    mixed.p.val <- anova(mixed.full, mixed.reduced)$"Pr(>Chisq)"[2]
    mixed.int <- fixef(mixed.full)["group1"]
    mixed.slope <- fixef(mixed.full)["group1:month"]

    writeLines("Full Mixed Effects Model")
    print(summary(mixed.full))
    writeLines("P Value for Full vs. Reduced Model")
```

© 2018 Kennedy RE et al. *JAMA Network Open*.

```
   print(mixed.p.val)
   result <- summary(mixed.full)$coefficients["group1:month",1:2]
   sample.times <- c(0, 6, 12, 18, 24) /12
   med.values <- fixef(mixed.full)["(Intercept)"] + fixef(mixed.full)["group1"] + sample.t
imes * fixef(mixed.full)["month"] + sample.times * fixef(mixed.full)["group1:month"]
   nomed.values <- fixef(mixed.full)["(Intercept)"] + sample.times * fixef(mixed.full)["m
onth"]
   plot(sample.times, nomed.values, main=study.name, xlab="Time in Years", ylab="Fitt
ed ADAS-cog score", col="black", lty=1, type="l", xaxt="n", ylim=c(0,70))
   lines(sample.times, med.values, col="black", lty=2, type="l", xaxt="n")
   axis(1, at = sample.times)
  }
  results <- rbind(results, result)
}
rownames(results) <- unique(sample.mixed$STUDY)
study.ids <- unique(study.ids)
idx <- all.ad$UID %in% study.ids
N <- table(all.ad$STUDY[idx], all.ad$group[idx])
idx <- N[,1] != 0
N <- N[idx,]
results <- as.data.frame(results)
results$medN <- N[,2]
results$nomedN <- N[,1]
results$author <- study.authors[rownames(results)]
results$year <- study.pubyears[rownames(results)]
results$refno <- study.refno[rownames(results)]
idx <- order(results$year, results$author)
results <- results[idx,]
meta <- rma(yi=results[,"Estimate"], sei=results[,"Std. Error"], slab=paste(rownames(re
sults), " (", results$author, ", ", results$year, ") [", results$refno, "]", sep=""))
print(meta)
results$severity <- "AD"
all.results <- results
rate.ad <- meta
#pdf("AAICRateForestAD080818.pdf", width=11, height=8.5)
forest(meta, ilab=cbind(results$medN, results$nomedN), ilab.xpos = c(-40, -25), digits=
c(3,0), cex=1.0, xlab="Difference in ADAS-cog Slopes", cex.lab=1, at=c(-15, -10,-5, 0, 5
, 10, 15), xlim=c(-70, 40), mlab="RE Model for AD") #, main="Comparison of Memanti
ne + ChEIs vs. Neither")
op <- par(cex = 1.0, font = 2)
text(-70, 11.5, "Abbreviation, Authors, Year", pos = 4, cex=1.0)
text(40.1, 11.5, "Slope Difference [95% CI]", pos = 2, cex=1.0)
text(c(-40, -25), 11.5, c("AD Meds", "No AD Meds"))
text(-33, 12, "Number of Subjects")
par(op, xpd=TRUE)
text(-21, -2.8, "Faster\ndecline\nwith\nneither")
```

© 2018 Kennedy RE et al. *JAMA Network Open*.

```
text(22, -2.8, "Faster\ndecline\nwith ChEIs\n/memantine")
#dev.off()
#pdf("AAICRateFunnelAD080818.pdf", width=11, height=8.5)
funnel(meta)
#text(meta$yi-c(1.5,1.5,1.6,1.5,1.5,1.8,1.6,1.5,1.5,1.5),sqrt(meta$vi)+c(0,0,0,0,0,0,0,0,0,0
.03),rownames(results),cex=.8)
text(meta$yi-c(0.35,0.35,0.35,0.35,0.35,0.35,0.35,0.35,0.45,0.35),sqrt(meta$vi),rowna
mes(results),cex=.8)
#dev.off()
colnames(results) <- c("Estimate", "StdError", "medN", "nomedN", "author", "year")
failsafe <- fsn(yi=Estimate, sei=StdError, data=results, type="Rosenberg")
print(failsafe)
#idx <- is.na(all.ad$ADAS_bl) & is.na(all.ad$ADAS_m06) & is.na(all.ad$ADAS_m12)
#all.ad <- all.ad[!idx,]

#idx <- all.ad$STUDY %in% adas.ad.studies
#all.ad <- all.ad[idx,]
# Hmisc::label(all.ad$AGE) <- "Age, years"
# Hmisc::label(all.ad$RACE) <- "Race"
# Hmisc::label(all.ad$ETHNIC) <- "Ethnicity"
# Hmisc::label(all.ad$SEX) <- "Sex"
# Hmisc::label(all.ad$MARITAL) <- "Married"
# Hmisc::label(all.ad$TX) <- "Treatment Arm"
# Hmisc::label(all.ad$EDCAT) <- "Education"
# Hmisc::label(all.ad$ADAS_bl) <- "ADAS-cog, Baseline"
# Hmisc::label(all.ad$ADAS_m06) <- "ADAS-cog, 6 months"
# Hmisc::label(all.ad$ADAS_m12) <- "ADAS-cog, 12 months"
# Hmisc::label(all.ad$ADAS_m18) <- "ADAS-cog, 18 months"
# Hmisc::label(all.ad$ADAS_m24) <- "ADAS-cog, 24 months"

label.names <- c("All", "   P Value", "Trend Test", "Odds Ratio")
names(label.names) <- c("all", "p.overall", "p.trend", "p.ratio")
temp <- compareGroups(group4 ~ AGE + EDCAT + RACE + ETHNIC + SEX + MAR
ITAL + TX + ADAS_bl + ADAS_m06 + ADAS_m12 + ADAS_m18 + ADAS_m24, dat
a=all.ad, subset=STUDY %in% adas.ad.studies & UID %in% study.ids)
tab <- createTable(temp, type = 2, hide=c(NA, NA, 2, 2, 1, 2, 2, NA, NA, NA, NA, NA)
, show.n=TRUE)
export2md(tab, caption = "Baseline Characteristics for All Studies", header.labels=label.
names, nmax=TRUE)

temp <- compareGroups(group4 ~ AGE + EDCAT + RACE + ETHNIC + SEX + MAR
ITAL + TX + ADAS_bl + ADAS_m06 + ADAS_m12 + ADAS_m18, data=all.ad, subset
=STUDY=="DHA" & UID %in% study.ids)
tab <- createTable(temp, type = 2, hide=c(NA, NA, 2, 2, 1, 2, 2, NA, NA, NA, NA, NA)
, show.n=TRUE)
export2md(tab, caption = "Baseline Characteristics for DHA", header.labels=label.name
```

© 2018 Kennedy RE et al. *JAMA Network Open*.

```
s, nmax=TRUE)

temp <- compareGroups(group4 ~ AGE + EDCAT + RACE + ETHNIC + SEX + MAR
ITAL + TX + ADAS_bl + ADAS_m06 + ADAS_m12, data=all.ad, subset=STUDY=="E
S" & UID %in% study.ids)
tab <- createTable(temp, type = 2, hide=c(NA, NA, 2, 2, 1, 2, 2, NA, NA, NA, NA, NA)
, show.n=TRUE)
export2md(tab, caption = "Baseline Characteristics for ES", header.labels=label.names,
nmax=TRUE)

temp <- compareGroups(group4 ~ AGE + EDCAT + RACE + ETHNIC + SEX + MAR
ITAL + TX + ADAS_bl + ADAS_m06 + ADAS_m12 + ADAS_m18, data=all.ad, subset
=STUDY=="HC" & UID %in% study.ids)
tab <- createTable(temp, type = 2, hide=c(NA, NA, 2, 2, 1, 2, 2, NA, NA, NA, NA, NA)
, show.n=TRUE)
export2md(tab, caption = "Baseline Characteristics for HC", header.labels=label.names,
nmax=TRUE)

temp <- compareGroups(group4 ~ AGE + EDCAT + RACE + ETHNIC + SEX + MAR
ITAL + TX + ADAS_bl + ADAS_m06 + ADAS_m12 + ADAS_m18, data=all.ad, subset
=STUDY=="HU" & UID %in% study.ids)
tab <- createTable(temp, type = 2, hide=c(NA, NA, 2, 2, 1, 2, 2, NA, NA, NA, NA, NA)
, show.n=TRUE)
export2md(tab, caption = "Baseline Characteristics for Study HU", header.labels=label.n
ames, nmax=TRUE)

temp <- compareGroups(group4 ~ AGE + EDCAT + RACE + ETHNIC + SEX + MAR
ITAL + TX + ADAS_bl + ADAS_m06 + ADAS_m12 + ADAS_m18, data=all.ad, subset
=STUDY=="LL" & UID %in% study.ids)
tab <- createTable(temp, type = 2, hide=c(NA, NA, 2, 2, 1, 2, 2, NA, NA, NA, NA, NA)
, show.n=TRUE)
export2md(tab, caption = "Baseline Characteristics for LL", header.labels=label.names,
nmax=TRUE)

temp <- compareGroups(group4 ~ AGE + EDCAT + RACE + ETHNIC + SEX + MAR
ITAL + TX + ADAS_bl + ADAS_m06 + ADAS_m12, data=all.ad, subset=STUDY=="N
S" & UID %in% study.ids)
tab <- createTable(temp, type = 2, hide=c(NA, NA, 2, 2, 1, 2, 2, NA, NA, NA, NA, NA)
, show.n=TRUE)
export2md(tab, caption = "Baseline Characteristics for NS", header.labels=label.names,
nmax=TRUE)

temp <- compareGroups(group4 ~ AGE + EDCAT + RACE + ETHNIC + SEX + MAR
ITAL + TX + ADAS_bl + ADAS_m06 + ADAS_m12 + ADAS_m18, data=all.ad, subset
=STUDY=="PR" & UID %in% study.ids)
tab <- createTable(temp, type = 2, hide=c(NA, NA, 2, 2, 1, 2, 2, NA, NA, NA, NA, NA)
```

© 2018 Kennedy RE et al. *JAMA Network Open*.

```
, show.n=TRUE)
export2md(tab, caption = "Baseline Characteristics for PR", header.labels=label.names,
nmax=TRUE)

temp <- compareGroups(group4 ~ AGE + EDCAT + RACE + ETHNIC + SEX + MAR
ITAL + TX + ADAS_bl + ADAS_m06 + ADAS_m12 + ADAS_m18 + ADAS_m24, dat
a=all.ad, subset=STUDY=="SL" & UID %in% study.ids)
tab <- createTable(temp, type = 2, hide=c(NA, NA, 2, 2, 1, 2, 2, NA, NA, NA, NA, NA)
, show.n=TRUE)
export2md(tab, caption = "Baseline Characteristics for SL", header.labels=label.names,
nmax=TRUE)

temp <- compareGroups(group4 ~ AGE + EDCAT + RACE + ETHNIC + SEX + MAR
ITAL + TX + ADAS_bl + ADAS_m06 + ADAS_m12 + ADAS_m18 + ADAS_m24, dat
a=all.ad, subset=STUDY=="VN" & UID %in% study.ids)
tab <- createTable(temp, type = 2, hide=c(NA, NA, 2, 2, 1, 2, 2, NA, NA, NA, NA, NA)
, show.n=TRUE)
export2md(tab, caption = "Baseline Characteristics for VN", header.labels=label.names,
nmax=TRUE)

temp <- compareGroups(group4 ~ AGE + EDCAT + RACE + ETHNIC + SEX + MAR
ITAL + TX + ADAS_bl + ADAS_m06 + ADAS_m12 + ADAS_m24, data=all.ad, subset
=STUDY=="ADNI" & UID %in% study.ids)
tab <- createTable(temp, type = 2, hide=c(NA, NA, 2, 2, 1, 2, 2, NA, NA, NA, NA, NA)
, show.n=TRUE)
export2md(tab, caption = "Baseline Characteristics for ADNI", header.labels=label.nam
es, nmax=TRUE)
idx <- all.ad$UID %in% study.ids
chei.pct <- tapply(ifelse(all.ad$group4[idx]=="ChEI" | all.ad$group4[idx]=="Both", 1, 0
), all.ad$STUDY[idx], mean, na.rm=TRUE)*100
memantine.pct <- tapply(ifelse(all.ad$group4[idx]=="Memantine" | all.ad$group4[idx]=
="Both", 1, 0), all.ad$STUDY[idx], mean, na.rm=TRUE)*100

#pdf("ChEIYear080818.pdf", width=11, height=8.5)
plot(study.years[names(chei.pct)], chei.pct, type="p", pch=16, col="blue", xlim=c(1992,
2007), ylim=c(0,100), xlab="Study Year", ylab="Percent of Participants Taking ChEIs")
#, main="Percent Taking ChEI by Publication Year")
textxy(2005, 90.4580152671756, labs="ADNI", cex=1)
textxy(2006, 84.2412451361868, labs="DHA", cex=1)
textxy(1995, 23.6641221374046, labs="ES", cex=1)
textxy(2003, 92.4939467312349-3, labs="HC", cex=1)
textxy(2004, 4.8780487804878, labs="HU", cex=1)
textxy(2003, 93.9903846153846+1, labs="LL", cex=1)
textxy(1999, 83.8068181818182, labs="NS", cex=1)
textxy(1995, 13.9705882352941, labs="PR", cex=1)
textxy(1993, 0.595238095238095+0.1, labs="SL", cex=1)
```

© 2018 Kennedy RE et al. *JAMA Network Open*.

```
textxy(2003, 93.0362116991644-1, labs="VN", cex=1)
text(1993, 80, "Tacrine\nApproved\n1993")
arrows(1993, 75, 1993, 70, length=0.1)
text(1996, 80, "Donepezil\nApproved\n1996")
arrows(1996, 75, 1996, 70, length=0.1)
text(2000, 99, "Rivastigmine\nApproved\n2000")
arrows(2000, 94, 2000, 89, length=0.1)
text(2001, 90, "Galantamine\nApproved\n2001")
arrows(2001, 85, 2001, 80, length=0.1)
#dev.off()
idx <- all.ad$UID %in% study.ids
chei.count <- sum(all.ad$group4[idx]=="ChEI" | all.ad$group4[idx]=="Both")

print(chei.count)

donepezil.total <- sum(all.ad$donepezil[idx], na.rm=TRUE)
galantamine.total <- sum(all.ad$galantamine[idx], na.rm=TRUE)
rivastigmine.total <- sum(all.ad$rivastigmine[idx], na.rm=TRUE)
metrifonate.total <- sum(all.ad$metrifonate[idx], na.rm=TRUE)
tacrine.total <- sum(all.ad$tacrine[idx], na.rm=TRUE)

each.chei.total <- matrix(c(donepezil.total, galantamine.total, rivastigmine.total, metrifon
ate.total, tacrine.total), ncol=1)
rownames(each.chei.total)=c("donepezil", "galantamine", "rivastigmine", "metrifonate",
"tacrine")
print(each.chei.total)

each.chei.pct <- each.chei.total / chei.count * 100
rownames(each.chei.pct)=c("donepezil", "galantamine", "rivastigmine", "metrifonate", "t
acrine")
print(each.chei.pct)

each.chei.count <- all.ad$donepezil[idx] + all.ad$galantamine[idx] + all.ad$rivastigmine[
idx] + all.ad$metrifonate[idx] + all.ad$tacrine[idx]
each.chei.count <- each.chei.count[each.chei.count != 0]
print(table(each.chei.count))
print(table(each.chei.count) / sum(table(each.chei.count)))

#pdf("EachChEI080818.pdf", width=11, height=8.5)
barplot(t(each.chei.pct), col="darkgreen", ylim=c(0,100), ylab="Percent of Subjects Tak
ing ChEIs", names.arg=c("Donepezil", "Galantamine", "Rivastigmine", "Metrifonate", "T
acrine")) #, main="Rates for Individual ChEIs")
#dev.off()
#pdf("MemantineYear080818.pdf", width=11, height=8.5)
plot(study.years[names(memantine.pct)], memantine.pct, type="p", pch=16, col="red", x
lim=c(1993, 2007), ylim=c(0,100), xlab="Study Year", ylab="Percent of Participants Tak
```

© 2018 Kennedy RE et al. *JAMA Network Open*.

```
ing Memantine") #, main="Percent Taking Memantine by Publication Year")
textxy(2005, 57.6335877862595, labs="ADNI",cex=1)
textxy(2006, 64.591439688716, labs="DHA", cex=1)
textxy(1995, 0-0.1, labs="ES", cex=1)
textxy(2003, 50.6053268765133, labs="HC", cex=1)
textxy(2004, 50.2439024390244, labs="HU", cex=1)
textxy(2003, 54.3269230769231, labs="LL", cex=1)
textxy(1999, 0.284090909090909, labs="NS", cex=1)
textxy(1995, 0, labs="PR", cex=1)
textxy(1993, 0-0.1, labs="SL", cex=1)
textxy(2003, 76.3231197771588, labs="VN", cex=1)
text(2003, 1, "Memantine\nApproved\n2003")
arrows(2003, 6, 2003, 11, length=0.1)
#dev.off()
idx <- all.ad$UID %in% study.ids
all.count <- table(all.ad$group4[idx])
print(all.count)
all.pct <- all.count / sum(all.count)*100
print(all.pct)

#pdf("ChEIMemantineYear080818.pdf", width=11, height=8.5)
plot(study.years[names(chei.pct)], chei.pct, type="p", pch=16, col="blue", xlim=c(1993,
2007), ylim=c(0,100), xlab="Study Year", ylab="Percent of Participants Taking ChEIs/M
emantine") #, main="Percent Taking ChEI by Publication Year")
textxy(2005, 90.4580152671756, labs="ADNI", cex=1)
textxy(2006, 84.2412451361868, labs="DHA", cex=1)
textxy(1995, 23.6641221374046, labs="ES", cex=1)
textxy(2003, 92.4939467312349-3, labs="HC", cex=1)
textxy(2004, 4.8780487804878, labs="HU", cex=1)
textxy(2003, 93.9903846153846+1, labs="LL", cex=1)
textxy(1999, 83.8068181818182, labs="NS", cex=1)
textxy(1995, 13.9705882352941, labs="PR", cex=1)
textxy(1993, 0.595238095238095+0.1, labs="SL", cex=1)
textxy(2003, 93.0362116991644-1, labs="VN", cex=1)
points(study.years[names(memantine.pct)], memantine.pct, type="p", pch=16, col="red"
) #, main="Percent Taking Memantine by Publication Year")
textxy(2005, 57.6335877862595, labs="ADNI",cex=1)
textxy(2006, 64.591439688716, labs="DHA", cex=1)
textxy(1995, 0-0.1, labs="ES", cex=1)
textxy(2003, 50.6053268765133, labs="HC", cex=1)
textxy(2004, 50.2439024390244, labs="HU", cex=1)
textxy(2003, 54.3269230769231, labs="LL", cex=1)
textxy(1999, 0.284090909090909, labs="NS", cex=1)
textxy(1995, 0, labs="PR", cex=1)
textxy(1993, 0-0.1, labs="SL", cex=1)
textxy(2003, 76.3231197771588, labs="VN", cex=1)
```

© 2018 Kennedy RE et al. *JAMA Network Open*.

```
text(1993, 80, "Tacrine\nApproved\n1993")
arrows(1993, 75, 1993, 70, length=0.1)
text(1996, 80, "Donepezil\nApproved\n1996")
arrows(1996, 75, 1996, 70, length=0.1)
text(2000, 99, "Rivastigmine\nApproved\n2000")
arrows(2000, 94, 2000, 89, length=0.1)
text(2001, 90, "Galantamine\nApproved\n2001")
arrows(2001, 85, 2001, 80, length=0.1)
text(2003, 1, "Memantine\nApproved\n2003")
arrows(2003, 6, 2003, 11, length=0.1)
legend("topleft", legend=c("ChEIs", "Memantine"), fill=c("blue", "red"))
#dev.off()
temp <- reshape(all.ad[,data.cols], idvar = "UID", varying = c("ADAS_bl", "ADAS_m0
6", "ADAS_m12", "ADAS_m18", "ADAS_m24"), direction = "long", sep="_")
temp$month <- recode(temp$time, "'bl'=0;'m06'=6;'m12'=12;'m18'=18;'m24'=24")

idx <- !is.na(temp$group)
temp <- temp[idx,]

idx <- order(temp$UID, temp$month)
temp <- temp[idx,]

idx <- !is.na(temp$ADAS)
temp <- temp[idx,]

sample.mixed <- temp
results <- NULL
study.ids <- NULL
for (i in c("DHA", "ES", "HC", "HU", "LL", "NS", "PR", "SL", "VN")) {
  study.name <- i
  idx <- sample.mixed$STUDY==study.name
  mixed.data <- sample.mixed[idx,]

  mixed.data$month <- as.numeric(mixed.data$month)
  mixed.data$group <- as.factor(mixed.data$group)
  mixed.data$month <- mixed.data$month / 12
  idx <- order(mixed.data$UID, mixed.data$month)
  mixed.data <- mixed.data[idx,]
  mixed.full <- try(lmer(ADAS ~ AGE + EDUCATION + group + month + group:month
+ (month|UID), REML=FALSE, data=mixed.data))
  mixed.reduced <- try(lmer(ADAS ~ AGE + EDUCATION + group + month + (month|
UID), REML=FALSE, data=mixed.data))

  if ((class(mixed.full) == "try-error") | (class(mixed.reduced) == "try-error")) {
    result <- NA
  } else {
```

© 2018 Kennedy RE et al. *JAMA Network Open*.

```
    study.ids <- c(study.ids, as.character(model.frame(mixed.full)$UID))
    mixed.p.val <- anova(mixed.full, mixed.reduced)$"Pr(>Chisq)"[2]
    mixed.int <- fixef(mixed.full)["group1"]
    mixed.slope <- fixef(mixed.full)["group1:month"]

#    print(summary(mixed.full))
#    print(mixed.p.val)
    result <- summary(mixed.full)$coefficients["group1:month",1:2]
    sample.times <- c(0, 6, 12, 18, 24) /12
    med.values <- fixef(mixed.full)["(Intercept)"] + fixef(mixed.full)["group1"] + sample.t
imes * fixef(mixed.full)["month"] + sample.times * fixef(mixed.full)["group1:month"]
    nomed.values <- fixef(mixed.full)["(Intercept)"] + sample.times * fixef(mixed.full)["m
onth"]
#    plot(sample.times, nomed.values, main=study.name, xlab="Time in Years", ylab="Fi
tted ADAS-cog score", col="black", lty=1, type="l", xaxt="n", ylim=c(0,70))
#    lines(sample.times, med.values, col="black", lty=2, type="l", xaxt="n")
#    axis(1, at = sample.times)
  }
  results <- rbind(results, result)
}
rownames(results) <- c("DHA", "ES", "HC", "HU", "LL", "NS", "PR", "SL", "VN")
study.ids <- unique(study.ids)
idx <- all.ad$UID %in% study.ids
N <- table(all.ad$STUDY[idx], all.ad$group[idx])
idx <- N[,1] != 0
N <- N[idx,]
results <- as.data.frame(results)
results$medN <- N[,2]
results$nomedN <- N[,1]
results$author <- study.authors[rownames(results)]
results$year <- study.pubyears[rownames(results)]
idx <- order(results$year, results$author)
results <- results[idx,]
meta <- rma(yi=results[,"Estimate"], sei=results[,"Std. Error"], slab=paste(rownames(re
sults), " (", results$author, ", ", results$year, ")", sep=""))
results$severity <- "AD"
all.results <- results
rate.ad <- meta
#pdf("AAICRateForestNoADNI080818.pdf", width=11, height=8.5)
forest(meta, ilab=cbind(results$medN, results$nomedN), ilab.xpos = c(-40, -25), digits=
c(3,0), cex=1.0, xlab="Difference in ADAS-cog Slopes", cex.lab=1, at=c(-15, -10,-5, 0, 5
, 10, 15), xlim=c(-70, 40), mlab="RE Model for AD") #, main="Comparison of Memanti
ne + ChEIs vs. Neither")
op <- par(cex = 1.0, font = 2)
text(-70, 11.5, "Abbreviation, Authors, Year", pos = 4, cex=1.0)
text(40.1, 11.5, "Slope Difference [95% CI]", pos = 2, cex=1.0)
```

© 2018 Kennedy RE et al. *JAMA Network Open*.

```
text(c(-40, -25), 11.5, c("AD Meds", "No AD Meds"))
text(-33, 12, "Number of Subjects")
par(op, xpd=TRUE)
text(-21, -2.8, "Faster\ndecline\nwith\nneither")
text(22, -2.8, "Faster\ndecline\nwith ChEIs\n/memantine")
#dev.off()
#pdf("AAICRateFunnelNoADNI080818.pdf", width=11, height=8.5)
funnel(meta)
#text(meta$yi-c(1.5,1.5,1.6,1.5,1.5,1.8,1.6,1.5,1.5,1.5),sqrt(meta$vi)+c(0,0,0,0,0,0,0,0,0,0
.03),rownames(results),cex=.8)
text(meta$yi-c(0.35,0.35,0.35,0.35,0.35,0.35,0.35,0.35,0.45,0.35),sqrt(meta$vi),rowna
mes(results),cex=.8)
#dev.off()
temp <- reshape(all.ad, idvar = "UID", varying = c("ADAS_bl", "ADAS_m06", "ADAS
_m12", "ADAS_m18", "ADAS_m24"), direction = "long", sep="_")
temp$month <- recode(temp$time, "'bl'=0;'m06'=6;'m12'=12;'m18'=18;'m24'=24")

idx <- !is.na(temp$group)
temp <- temp[idx,]

idx <- order(temp$UID, temp$month)
temp <- temp[idx,]

idx <- !is.na(temp$ADAS)
temp <- temp[idx,]

sample.mixed <- temp
results <- NULL
study.ids <- NULL
for (i in unique(sample.mixed$STUDY)) {
  study.name <- i
  writeLines("Study Name")
  print(study.name)
  idx <- sample.mixed$STUDY==study.name
  mixed.data <- sample.mixed[idx,]

  mixed.data$month <- as.numeric(mixed.data$month)
  mixed.data$group <- as.factor(mixed.data$group)
  mixed.data$month <- mixed.data$month / 12
  idx <- order(mixed.data$UID, mixed.data$month)
  mixed.data <- mixed.data[idx,]
  mixed.full <- try(lmer(ADAS ~ AGE + EDUCATION + group + month + group:month
+ (month|UID), REML=FALSE, data=mixed.data))
  mixed.reduced <- try(lmer(ADAS ~ AGE + EDUCATION + group + month + (month|
UID), REML=FALSE, data=mixed.data))
```

© 2018 Kennedy RE et al. *JAMA Network Open*.

```
  if ((class(mixed.full) == "try-error") | (class(mixed.reduced) == "try-error")) {
    result <- NA
  } else {
    study.ids <- c(study.ids, as.character(model.frame(mixed.full)$UID))
    mixed.p.val <- anova(mixed.full, mixed.reduced)$"Pr(>Chisq)"[2]
    mixed.int <- fixef(mixed.full)["group1"]
    mixed.slope <- fixef(mixed.full)["group1:month"]

    dscore <- lme.dscore(mixed.full, data=mixed.data, type="lme4")
    writeLines("Effect Sizes")
    print(dscore)
    d <- dscore["group1:month","d"]
    idx <- !duplicated(mixed.data$UID)
    N <- table(mixed.data$group[idx])
    ci <- cohensd.ci(d=d, n1=N[1], n2=N[2])
    se <- (ci[2] - ci[1]) / (2 * 1.96)
    result <- data.frame(d, se)
  }
  results <- rbind(results, result)
}
rownames(results) <- unique(sample.mixed$STUDY)
N <- table(all.ad$STUDY, all.ad$group)
idx <- N[,1] != 0
N <- N[idx,]
results <- as.data.frame(results)
results$medN <- N[,2]
results$nomedN <- N[,1]
results$author <- study.authors[rownames(results)]
results$year <- study.pubyears[rownames(results)]
idx <- order(results$author, results$year)
results <- results[idx,]
meta <- rma(yi=results[,"d"], sei=results[,"se"], slab=paste(rownames(results), " (", resu
lts$author, ", ", results$year, ")", sep=""))
print(meta)
all.results <- results
#pdf("AAICRateForestES080818.pdf", width=11, height=8.5)
forest(meta, ilab=cbind(results$medN, results$nomedN), ilab.xpos = c(-40, -25), digits=
c(3,1), cex=1.0, xlab="Difference in ADAS-cog Slopes", cex.lab=1, at=c(-2, -1,-0.5, 0, 0.
5, 1, 2), xlim=c(-15, 6), mlab="RE Model for AD") #, main="Comparison of Memantine
+ ChEIs vs. Neither")
op <- par(cex = 1.0, font = 2)
text(-70, 11.5, "Abbreviation, Authors, Year", pos = 4, cex=1.0)
text(40.1, 11.5, "Slope Difference [95% CI]", pos = 2, cex=1.0)
text(c(-40, -25), 11.5, c("AD Meds", "No AD Meds"))
text(-33, 12, "Number of Subjects")
par(op, xpd=TRUE)
```

© 2018 Kennedy RE et al. *JAMA Network Open*.

```
text(-21, -2.8, "Faster\ndecline\nwith\nneither")
text(22, -2.8, "Faster\ndecline\nwith ChEIs\n/memantine")
#dev.off()
#pdf("AAICRateFunnelES080818.pdf", width=11, height=8.5)
funnel(meta)
#text(meta$yi-c(1.5,1.5,1.6,1.5,1.5,1.8,1.6,1.5,1.5,1.5),sqrt(meta$vi)+c(0,0,0,0,0,0,0,0,0,0
.03),rownames(results),cex=.8)
text(meta$yi-c(0.35,0.35,0.35,0.35,0.35,0.35,0.35,0.35,0.45,0.35),sqrt(meta$vi),rowna
mes(results),cex=.8)
#dev.off()
data.cols <- c("UID", "STUDY", "AGE", "EDUCATION", "ADAS_bl", "ADAS_m06",
"ADAS_m12", "ADAS_m18", "ADAS_m24", "group", "group4")
idx <- all.data$SEVERITY=="AD"
all.ad <- all.data[idx,data.cols]
idx <- !duplicated(all.ad$UID)
all.ad <- all.ad[idx,]
idx <- !is.na(all.ad$STUDY)
all.ad <- all.ad[idx,]
idx <- !is.na(all.ad$ADAS_bl)
all.ad <- all.ad[idx,]
idx <- all.ad$STUDY %in% chei.studies
all.ad <- all.ad[idx,]
temp <- reshape(all.ad, idvar = "UID", varying = c("ADAS_bl", "ADAS_m06", "ADAS
_m12", "ADAS_m18", "ADAS_m24"), direction = "long", sep="_")
temp$month <- recode(temp$time, "'bl'=0;'m06'=6;'m12'=12;'m18'=18;'m24'=24")

temp$group <- ifelse(temp$group4=="ChEI", 1, ifelse(temp$group4=="Neither", 0, NA)
)
#  ifelse($memantine==1, ifelse(medhist$chei==1,"Both", "Memantine"), ifelse(medhist$
chei==1, "ChEI", "Neither"))

idx <- !is.na(temp$group)
temp <- temp[idx,]

idx <- order(temp$UID, temp$month)
temp <- temp[idx,]

idx <- !is.na(temp$ADAS)
temp <- temp[idx,]

sample.mixed <- temp
results <- NULL
for (i in unique(sample.mixed$STUDY)) {
  study.name <- i
  writeLines("Study Name")
  print(study.name)
```

© 2018 Kennedy RE et al. *JAMA Network Open*.

```
  idx <- sample.mixed$STUDY==study.name
  mixed.data <- sample.mixed[idx,]

  mixed.data$month <- as.numeric(mixed.data$month)
  mixed.data$group <- as.factor(mixed.data$group)
  mixed.data$month <- mixed.data$month / 12
  idx <- order(mixed.data$UID, mixed.data$month)
  mixed.data <- mixed.data[idx,]
  mixed.full <- try(lmer(ADAS ~ AGE + EDUCATION + group + month + group:month
+ (month|UID), REML=FALSE, data=mixed.data))
  mixed.reduced <- try(lmer(ADAS ~ AGE + EDUCATION + group + month + (month|
UID), REML=FALSE, data=mixed.data))

  if ((class(mixed.full) == "try-error") | (class(mixed.reduced) == "try-error")) {
    result <- NA
  } else {
    mixed.p.val <- anova(mixed.full, mixed.reduced)$"Pr(>Chisq)"[2]
    mixed.int <- fixef(mixed.full)["group1"]
    mixed.slope <- fixef(mixed.full)["group1:month"]

    writeLines("Full Mixed Effects Model")
    print(summary(mixed.full))
    writeLines("P Value for Full vs. Reduced Model")
    print(mixed.p.val)
    result <- summary(mixed.full)$coefficients["group1:month",1:2]
    sample.times <- c(0, 6, 12, 18, 24) /12
    med.values <- fixef(mixed.full)["(Intercept)"] + fixef(mixed.full)["group1"] + sample.t
imes * fixef(mixed.full)["month"] + sample.times * fixef(mixed.full)["group1:month"]
    nomed.values <- fixef(mixed.full)["(Intercept)"] + sample.times * fixef(mixed.full)["m
onth"]
    plot(sample.times, nomed.values, main=study.name, xlab="Time in Years", ylab="Fitt
ed ADAS-cog score", col="black", lty=1, type="l", xaxt="n", ylim=c(0,70))
    lines(sample.times, med.values, col="black", lty=2, type="l", xaxt="n")
    axis(1, at = sample.times)
  }
  results <- rbind(results, result)
}
rownames(results) <- unique(sample.mixed$STUDY)
N <- table(all.ad$STUDY, all.ad$group4)
idx <- N[,"ChEI"] != 0
N <- N[idx,]
results <- as.data.frame(results)
results$medN <- N[,"ChEI"]
results$nomedN <- N[,"Neither"]
results$author <- study.authors[rownames(results)]
results$year <- study.pubyears[rownames(results)]
```

© 2018 Kennedy RE et al. *JAMA Network Open*.

```
idx <- order(results$year, results$author)
results <- results[idx,]
meta <- rma(yi=results[,"Estimate"], sei=results[,"Std. Error"])
print(meta)
results$severity <- "AD"
all.results <- results
rate.ad <- meta
#pdf("AAICRateForestChEI080818.pdf", width=11, height=8.5)
forest(meta, slab=paste(rownames(results), " (", results$author, ", ", results$year, ")", se
p=""), ilab=cbind(results$medN, results$nomedN), ilab.xpos = c(-40, -25), digits=c(3,0),
cex=1.0, xlab="Difference in ADAS-cog Slopes", cex.lab=1, at=c(-15, -10,-5, 0, 5, 10, 1
5), xlim=c(-70, 40), mlab="RE Model for AD") #, main="Comparison of ChEIs vs. Neith
er")
op <- par(cex = 1.0, font = 2)
text(-70, 11.5, "Abbreviation, Authors, Year", pos = 4, cex=1.0)
text(40.1, 11.5, "Slope Difference [95% CI]", pos = 2, cex=1.0)
text(c(-40, -25), 11.5, c("ChEIs", "No ChEIs"))
text(-33, 12, "Number of Subjects")
par(op, xpd=TRUE)
text(-21, -2.8, "Faster\ndecline\nwith\nneither")
text(22, -2.8, "Faster\ndecline\nwith\nChEIs")
#dev.off()
data.cols <- c("UID", "STUDY", "AGE", "EDUCATION", "ADAS_bl", "ADAS_m06",
"ADAS_m12", "ADAS_m18", "ADAS_m24", "group", "group4")
idx <- all.data$SEVERITY=="AD"
all.ad <- all.data[idx,data.cols]
idx <- !duplicated(all.ad$UID)
all.ad <- all.ad[idx,]
idx <- !is.na(all.ad$STUDY)
all.ad <- all.ad[idx,]
idx <- !is.na(all.ad$ADAS_bl)
all.ad <- all.ad[idx,]
idx <- all.ad$STUDY %in% chei.studies
all.ad <- all.ad[idx,]
temp <- reshape(all.ad, idvar = "UID", varying = c("ADAS_bl", "ADAS_m06", "ADAS
_m12", "ADAS_m18", "ADAS_m24"), direction = "long", sep="_")
temp$month <- recode(temp$time, "'bl'=0;'m06'=6;'m12'=12;'m18'=18;'m24'=24")

temp$group <- ifelse(temp$group4=="ChEI", 1, ifelse(temp$group4=="Neither", 0, NA)
)
#  ifelse($memantine==1, ifelse(medhist$chei==1,"Both", "Memantine"), ifelse(medhist$
chei==1, "ChEI", "Neither"))

idx <- !is.na(temp$group)
temp <- temp[idx,]
```

© 2018 Kennedy RE et al. *JAMA Network Open*.

```r
idx <- order(temp$UID, temp$month)
temp <- temp[idx,]

idx <- !is.na(temp$ADAS)
temp <- temp[idx,]

sample.mixed <- temp
results <- NULL
for (i in unique(sample.mixed$STUDY)) {
  study.name <- i
  writeLines("Study Name")
  print(study.name)
  idx <- sample.mixed$STUDY==study.name
  mixed.data <- sample.mixed[idx,]

  mixed.data$month <- as.numeric(mixed.data$month)
  mixed.data$group <- as.factor(mixed.data$group)
  mixed.data$month <- mixed.data$month / 12
  idx <- order(mixed.data$UID, mixed.data$month)
  mixed.data <- mixed.data[idx,]
  mixed.full <- try(lmer(ADAS ~ AGE + EDUCATION + group + month + group:month
+ (month|UID), REML=FALSE, data=mixed.data))
  mixed.reduced <- try(lmer(ADAS ~ AGE + EDUCATION + group + month + (month|
UID), REML=FALSE, data=mixed.data))

  if ((class(mixed.full) == "try-error") | (class(mixed.reduced) == "try-error")) {
    result <- NA
  } else {
    mixed.p.val <- anova(mixed.full, mixed.reduced)$"Pr(>Chisq)"[2]
    mixed.int <- fixef(mixed.full)["group1"]
    mixed.slope <- fixef(mixed.full)["group1:month"]

    writeLines("Full Mixed Effects Model")
    print(summary(mixed.full))
    writeLines("P Value for Full vs. Reduced Model")
    print(mixed.p.val)
    result <- summary(mixed.full)$coefficients["group1:month",1:2]
    sample.times <- c(0, 6, 12, 18, 24) /12
    med.values <- fixef(mixed.full)["(Intercept)"] + fixef(mixed.full)["group1"] + sample.t
imes * fixef(mixed.full)["month"] + sample.times * fixef(mixed.full)["group1:month"]
    nomed.values <- fixef(mixed.full)["(Intercept)"] + sample.times * fixef(mixed.full)["m
onth"]
    plot(sample.times, nomed.values, main=study.name, xlab="Time in Years", ylab="Fitt
ed ADAS-cog score", col="black", lty=1, type="l", xaxt="n", ylim=c(0,70))
    lines(sample.times, med.values, col="black", lty=2, type="l", xaxt="n")
    axis(1, at = sample.times)
```

© 2018 Kennedy RE et al. *JAMA Network Open*.

```
  }
  results <- rbind(results, result)
}
rownames(results) <- unique(sample.mixed$STUDY)
N <- table(all.ad$STUDY, all.ad$group4)
idx <- N[,"ChEI"] != 0
N <- N[idx,]
results <- as.data.frame(results)
results$medN <- N[,"ChEI"]
results$nomedN <- N[,"Neither"]
results$author <- study.authors[rownames(results)]
results$year <- study.pubyears[rownames(results)]

idx <- order(results$year, results$author)
results <- results[idx,]
meta <- rma(yi=results[,"Estimate"], sei=results[,"Std. Error"], mods=study.relyears[row
names(results)])
print(meta)
results$severity <- "AD"
all.results <- results
rate.ad <- meta
#pdf("AAICRateForestChEIModerator080818.pdf", width=11, height=8.5)
forest(meta, slab=paste(rownames(results), " (", results$author, ", ", results$year, ")", se
p=""), ilab=cbind(results$medN, results$nomedN), ilab.xpos = c(-40, -25), digits=c(3,0),
cex=1.0, xlab="Difference in ADAS-cog Slopes", cex.lab=1, at=c(-15, -10,-5, 0, 5, 10, 1
5), xlim=c(-70, 40), mlab="RE Model for AD") #, main="Comparison of ChEIs vs. Neith
er")
op <- par(cex = 1.0, font = 2)
text(-70, 11.5, "Abbreviation, Authors, Year", pos = 4, cex=1.0)
text(40.1, 11.5, "Slope Difference [95% CI]", pos = 2, cex=1.0)
text(c(-40, -25), 11.5, c("ChEIs", "No ChEIs"))
text(-33, 12, "Number of Subjects")
par(op, xpd=TRUE)
text(-21, -0.6, "Faster\ndecline\nwith\nneither")
text(22, -0.6, "Faster\ndecline\nwith\nChEIs")
#dev.off()
data.cols <- c("UID", "STUDY", "AGE", "EDUCATION", "ADAS_bl", "ADAS_m06",
"ADAS_m12", "ADAS_m18", "ADAS_m24", "group", "group4")
idx <- all.data$SEVERITY=="AD"
all.ad <- all.data[idx,data.cols]
idx <- !duplicated(all.ad$UID)
all.ad <- all.ad[idx,]
idx <- !is.na(all.ad$STUDY)
all.ad <- all.ad[idx,]
idx <- !is.na(all.ad$ADAS_bl)
all.ad <- all.ad[idx,]
```

© 2018 Kennedy RE et al. *JAMA Network Open*.

```
idx <- all.ad$STUDY %in% chei.studies
all.ad <- all.ad[idx,]
temp <- reshape(all.ad, idvar = "UID", varying = c("ADAS_bl", "ADAS_m06", "ADAS
_m12", "ADAS_m18", "ADAS_m24"), direction = "long", sep="_")
temp$month <- recode(temp$time, "'bl'=0;'m06'=6;'m12'=12;'m18'=18;'m24'=24")

temp$group <- ifelse(temp$group4=="ChEI", 1, ifelse(temp$group4=="Neither", 0, NA)
)
#  ifelse($memantine==1, ifelse(medhist$chei==1,"Both", "Memantine"), ifelse(medhist$
chei==1, "ChEI", "Neither"))

idx <- !is.na(temp$group)
temp <- temp[idx,]

idx <- order(temp$UID, temp$month)
temp <- temp[idx,]

idx <- !is.na(temp$ADAS)
temp <- temp[idx,]

sample.mixed <- temp
results <- NULL
for (i in unique(sample.mixed$STUDY)) {
  study.name <- i
  writeLines("Study Name")
  print(study.name)
  idx <- sample.mixed$STUDY==study.name
  mixed.data <- sample.mixed[idx,]

  mixed.data$month <- as.numeric(mixed.data$month)
  mixed.data$group <- as.factor(mixed.data$group)
  mixed.data$month <- mixed.data$month / 12
  idx <- order(mixed.data$UID, mixed.data$month)
  mixed.data <- mixed.data[idx,]
  mixed.full <- try(lmer(ADAS ~ AGE + EDUCATION + group + month + group:month
+ (month|UID), REML=FALSE, data=mixed.data))
  mixed.reduced <- try(lmer(ADAS ~ AGE + EDUCATION + group + month + (month|
UID), REML=FALSE, data=mixed.data))

  if ((class(mixed.full) == "try-error") | (class(mixed.reduced) == "try-error")) {
    result <- NA
  } else {
    mixed.p.val <- anova(mixed.full, mixed.reduced)$"Pr(>Chisq)"[2]
    mixed.int <- fixef(mixed.full)["group1"]
    mixed.slope <- fixef(mixed.full)["group1:month"]
```

© 2018 Kennedy RE et al. *JAMA Network Open*.

```
    writeLines("Full Mixed Effects Model")
    print(summary(mixed.full))
    writeLines("P Value for Full vs. Reduced Model")
    print(mixed.p.val)
    result <- summary(mixed.full)$coefficients["group1:month",1:2]
    sample.times <- c(0, 6, 12, 18, 24) /12
    med.values <- fixef(mixed.full)["(Intercept)"] + fixef(mixed.full)["group1"] + sample.t
imes * fixef(mixed.full)["month"] + sample.times * fixef(mixed.full)["group1:month"]
    nomed.values <- fixef(mixed.full)["(Intercept)"] + sample.times * fixef(mixed.full)["m
onth"]
    plot(sample.times, nomed.values, main=study.name, xlab="Time in Years", ylab="Fitt
ed ADAS-cog score", col="black", lty=1, type="l", xaxt="n", ylim=c(0,70))
    lines(sample.times, med.values, col="black", lty=2, type="l", xaxt="n")
    axis(1, at = sample.times)
  }
  results <- rbind(results, result)
}
rownames(results) <- unique(sample.mixed$STUDY)
N <- table(all.ad$STUDY, all.ad$group4)
idx <- N[,"ChEI"] != 0
N <- N[idx,]
results <- as.data.frame(results)
results$medN <- N[,"ChEI"]
results$nomedN <- N[,"Neither"]
results$author <- study.authors[rownames(results)]
results$year <- study.pubyears[rownames(results)]

idx <- order(results$year, results$author)
results <- results[idx,]
meta <- rma(yi=results[,"Estimate"], sei=results[,"Std. Error"], mods=chei.pct[rowname
s(results)])
print(meta)
results$severity <- "AD"
all.results <- results
rate.ad <- meta
#pdf("AAICRateForestChEIPct080818.pdf", width=11, height=8.5)
forest(meta, slab=paste(rownames(results), " (", results$author, ", ", results$year, ")", se
p=""), ilab=cbind(results$medN, results$nomedN), ilab.xpos = c(-40, -25), digits=c(3,0),
cex=1.0, xlab="Difference in ADAS-cog Slopes", cex.lab=1, at=c(-15, -10,-5, 0, 5, 10, 1
5), xlim=c(-70, 40), mlab="RE Model for AD") #, main="Comparison of ChEIs vs. Neith
er")
op <- par(cex = 1.0, font = 2)
text(-70, 11.5, "Abbreviation, Authors, Year", pos = 4, cex=1.0)
text(40.1, 11.5, "Slope Difference [95% CI]", pos = 2, cex=1.0)
text(c(-40, -25), 11.5, c("ChEIs", "No ChEIs"))
text(-33, 12, "Number of Subjects")
```

© 2018 Kennedy RE et al. *JAMA Network Open*.

```
par(op, xpd=TRUE)
text(-21, -0.6, "Faster\ndecline\nwith\nneither")
text(22, -0.6, "Faster\ndecline\nwith\nChEIs")
#dev.off()
data.cols <- c("UID", "STUDY", "AGE", "EDUCATION", "ADAS_bl", "ADAS_m06",
"ADAS_m12", "ADAS_m18", "ADAS_m24", "group", "group4")
idx <- all.data$SEVERITY=="AD"
all.ad <- all.data[idx,data.cols]
idx <- !duplicated(all.ad$UID)
all.ad <- all.ad[idx,]
idx <- !is.na(all.ad$STUDY)
all.ad <- all.ad[idx,]
idx <- !is.na(all.ad$ADAS_bl)
all.ad <- all.ad[idx,]
idx <- all.ad$STUDY %in% memantine.studies
all.ad <- all.ad[idx,]
temp <- reshape(all.ad, idvar = "UID", varying = c("ADAS_bl", "ADAS_m06", "ADAS
_m12", "ADAS_m18", "ADAS_m24"), direction = "long", sep="_")
temp$month <- recode(temp$time, "'bl'=0;'m06'=6;'m12'=12;'m18'=18;'m24'=24")

temp$group <- ifelse(temp$group4=="Memantine" | temp$group4=="Both", 1, ifelse(te
mp$group4=="ChEI", 0, NA))

idx <- !is.na(temp$group)
temp <- temp[idx,]

idx <- order(temp$UID, temp$month)
temp <- temp[idx,]

idx <- !is.na(temp$ADAS)
temp <- temp[idx,]

sample.mixed <- temp
results <- NULL
for (i in unique(sample.mixed$STUDY)) {
  study.name <- i
  writeLines("Study Name")
  print(study.name)
  idx <- sample.mixed$STUDY==study.name
  mixed.data <- sample.mixed[idx,]

  mixed.data$month <- as.numeric(mixed.data$month)
  mixed.data$group <- as.factor(mixed.data$group)
  mixed.data$month <- mixed.data$month / 12
  idx <- order(mixed.data$UID, mixed.data$month)
  mixed.data <- mixed.data[idx,]
```

© 2018 Kennedy RE et al. *JAMA Network Open*.

```
  mixed.full <- try(lmer(ADAS ~ AGE + EDUCATION + group + month + group:month + (month|UID), REML=FALSE, data=mixed.data))
  mixed.reduced <- try(lmer(ADAS ~ AGE + EDUCATION + group + month + (month|UID), REML=FALSE, data=mixed.data))

  if ((class(mixed.full) == "try-error") | (class(mixed.reduced) == "try-error")) {
    result <- NA
  } else {
    mixed.p.val <- anova(mixed.full, mixed.reduced)$"Pr(>Chisq)"[2]
    mixed.int <- fixef(mixed.full)["group1"]
    mixed.slope <- fixef(mixed.full)["group1:month"]

    writeLines("Full Mixed Effects Model")
    print(summary(mixed.full))
    writeLines("P Value for Full vs. Reduced Model")
    print(mixed.p.val)
    result <- summary(mixed.full)$coefficients["group1:month",1:2]
    sample.times <- c(0, 6, 12, 18, 24) /12
    med.values <- fixef(mixed.full)["(Intercept)"] + fixef(mixed.full)["group1"] + sample.times * fixef(mixed.full)["month"] + sample.times * fixef(mixed.full)["group1:month"]
    nomed.values <- fixef(mixed.full)["(Intercept)"] + sample.times * fixef(mixed.full)["month"]
    plot(sample.times, nomed.values, main=study.name, xlab="Time in Years", ylab="Fitted ADAS-cog score", col="black", lty=1, type="l", xaxt="n", ylim=c(0,70))
    lines(sample.times, med.values, col="black", lty=2, type="l", xaxt="n")
    axis(1, at = sample.times)
  }
  results <- rbind(results, result)
}
rownames(results) <- unique(sample.mixed$STUDY)
N <- table(all.ad$STUDY, all.ad$group4)
idx <- N[,1] != 0
N <- N[idx,]
results <- as.data.frame(results)
results$medN <- N[,"Memantine"] + N[,"Both"]
results$nomedN <- N[,"Neither"] + N[,"ChEI"]
results$author <- study.authors[rownames(results)]
results$year <- study.pubyears[rownames(results)]

idx <- order(results$year, results$author)
results <- results[idx,]
meta <- rma(yi=results[,"Estimate"], sei=results[,"Std. Error"])
print(meta)
results$severity <- "AD"
all.results <- results
rate.ad <- meta
```

© 2018 Kennedy RE et al. *JAMA Network Open*.

```
#pdf("AAICRateForestMemantine080818.pdf", width=11, height=8.5)
forest(meta, slab=paste(rownames(results), " (", results$author, ", ", results$year, ")", se
p=""), ilab=cbind(results$medN, results$nomedN), ilab.xpos = c(-40, -25), digits=c(3,0),
cex=1.0, xlab="Difference in ADAS-cog Slopes", cex.lab=1, at=c(-15, -10,-5, 0, 5, 10, 1
5), xlim=c(-70, 40), mlab="RE Model for AD") #, main="Comparison of Memantine + B
oth vs. ChEIs + Neither")
op <- par(cex = 1.0, font = 2)
text(-70, 7.5, "Abbreviation, Authors, Year", pos = 4, cex=1.0)
text(40.1, 7.5, "Slope Difference [95% CI]", pos = 2, cex=1.0)
text(c(-40, -25), 7.5, c("Memantine", "No Memantine"))
text(-33, 8, "Number of Subjects")
par(op, xpd=TRUE)
text(-21, -2.4, "Faster\ndecline\nwith ChEIs\n/neither")
text(22, -2.4, "Faster\ndecline\nwith both\n/memantine")
#dev.off()
# Rates for memantine vs. ChEIs, with moderator of study year
data.cols <- c("UID", "STUDY", "AGE", "EDUCATION", "ADAS_bl", "ADAS_m06",
"ADAS_m12", "ADAS_m18", "ADAS_m24", "group", "group4")
idx <- all.data$SEVERITY=="AD"
all.ad <- all.data[idx,data.cols]
idx <- !duplicated(all.ad$UID)
all.ad <- all.ad[idx,]
idx <- !is.na(all.ad$STUDY)
all.ad <- all.ad[idx,]
idx <- !is.na(all.ad$ADAS_bl)
all.ad <- all.ad[idx,]
idx <- all.ad$STUDY %in% memantine.studies
all.ad <- all.ad[idx,]
temp <- reshape(all.ad, idvar = "UID", varying = c("ADAS_bl", "ADAS_m06", "ADAS
_m12", "ADAS_m18", "ADAS_m24"), direction = "long", sep="_")
temp$month <- recode(temp$time, "'bl'=0;'m06'=6;'m12'=12;'m18'=18;'m24'=24")

temp$group <- ifelse(temp$group4=="Memantine" | temp$group4=="Both", 1, ifelse(te
mp$group4=="ChEI", 0, NA))

idx <- !is.na(temp$group)
temp <- temp[idx,]

idx <- order(temp$UID, temp$month)
temp <- temp[idx,]

idx <- !is.na(temp$ADAS)
temp <- temp[idx,]

sample.mixed <- temp
results <- NULL
```

© 2018 Kennedy RE et al. *JAMA Network Open*.

```
for (i in unique(sample.mixed$STUDY)) {
  study.name <- i
  writeLines("Study Name")
  print(study.name)
  idx <- sample.mixed$STUDY==study.name
  mixed.data <- sample.mixed[idx,]

  mixed.data$month <- as.numeric(mixed.data$month)
  mixed.data$group <- as.factor(mixed.data$group)
  mixed.data$month <- mixed.data$month / 12
  idx <- order(mixed.data$UID, mixed.data$month)
  mixed.data <- mixed.data[idx,]
  mixed.full <- try(lmer(ADAS ~ AGE + EDUCATION + group + month + group:month + (month|UID), REML=FALSE, data=mixed.data))
  mixed.reduced <- try(lmer(ADAS ~ AGE + EDUCATION + group + month + (month|UID), REML=FALSE, data=mixed.data))

  if ((class(mixed.full) == "try-error") | (class(mixed.reduced) == "try-error")) {
    result <- NA
  } else {
    mixed.p.val <- anova(mixed.full, mixed.reduced)$"Pr(>Chisq)"[2]
    mixed.int <- fixef(mixed.full)["group1"]
    mixed.slope <- fixef(mixed.full)["group1:month"]

    writeLines("Full Mixed Effects Model")
    print(summary(mixed.full))
    writeLines("P Value for Full vs. Reduced Model")
    print(mixed.p.val)
    result <- summary(mixed.full)$coefficients["group1:month",1:2]
    sample.times <- c(0, 6, 12, 18, 24) /12
    med.values <- fixef(mixed.full)["(Intercept)"] + fixef(mixed.full)["group1"] + sample.times * fixef(mixed.full)["month"] + sample.times * fixef(mixed.full)["group1:month"]
    nomed.values <- fixef(mixed.full)["(Intercept)"] + sample.times * fixef(mixed.full)["month"]
    plot(sample.times, nomed.values, main=study.name, xlab="Time in Years", ylab="Fitted ADAS-cog score", col="black", lty=1, type="l", xaxt="n", ylim=c(0,70))
    lines(sample.times, med.values, col="black", lty=2, type="l", xaxt="n")
    axis(1, at = sample.times)
  }
  results <- rbind(results, result)
}
rownames(results) <- unique(sample.mixed$STUDY)
N <- table(all.ad$STUDY, all.ad$group4)
idx <- N[,1] != 0
N <- N[idx,]
results <- as.data.frame(results)
```

© 2018 Kennedy RE et al. *JAMA Network Open*.

```
results$medN <- N[,"Memantine"] + N[,"Both"]
results$nomedN <- N[,"Neither"] + N[,"ChEI"]
results$author <- study.authors[rownames(results)]
results$year <- study.pubyears[rownames(results)]

idx <- order(results$year, results$author)
results <- results[idx,]
meta <- rma(yi=results[,"Estimate"], sei=results[,"Std. Error"], mods=study.relyears[row
names(results)])
print(meta)
results$severity <- "AD"
all.results <- results
rate.ad <- meta
#pdf("AAICRateForestMemantineModerator080818.pdf", width=11, height=8.5)
forest(meta, slab=paste(rownames(results), " (", results$author, ", ", results$year, ")", se
p=""), ilab=cbind(results$medN, results$nomedN), ilab.xpos = c(-40, -25), digits=c(3,0),
cex=1.0, xlab="Difference in ADAS-cog Slopes", cex.lab=1, at=c(-15, -10,-5, 0, 5, 10, 1
5), xlim=c(-70, 40), mlab="RE Model for AD") #, main="Comparison of Memantine + B
oth vs. ChEIs + Neither")
op <- par(cex = 1.0, font = 2)
text(-70, 7.5, "Abbreviation, Authors, Year", pos = 4, cex=1.0)
text(40.1, 7.5, "Slope Difference [95% CI]", pos = 2, cex=1.0)
text(c(-40, -25), 7.5, c("Memantine", "No Memantine"))
text(-33, 8, "Number of Subjects")
par(op, xpd=TRUE)
text(-21, -0.3, "Faster\ndecline\nwith ChEIs\n/neither")
text(22, -0.3, "Faster\ndecline\nwith both\n/memantine")
#dev.off()
data.cols <- c("UID", "STUDY", "AGE", "EDUCATION", "ADAS_bl", "ADAS_m06",
"ADAS_m12", "ADAS_m18", "ADAS_m24", "group", "group4")
idx <- all.data$SEVERITY=="AD"
all.ad <- all.data[idx,data.cols]
idx <- !duplicated(all.ad$UID)
all.ad <- all.ad[idx,]
idx <- !is.na(all.ad$STUDY)
all.ad <- all.ad[idx,]
idx <- !is.na(all.ad$ADAS_bl)
all.ad <- all.ad[idx,]
idx <- all.ad$STUDY %in% memantine.studies
all.ad <- all.ad[idx,]
temp <- reshape(all.ad, idvar = "UID", varying = c("ADAS_bl", "ADAS_m06", "ADAS
_m12", "ADAS_m18", "ADAS_m24"), direction = "long", sep="_")
temp$month <- recode(temp$time, "'bl'=0;'m06'=6;'m12'=12;'m18'=18;'m24'=24")

temp$group <- ifelse(temp$group4=="Memantine" | temp$group4=="Both", 1, ifelse(te
mp$group4=="ChEI", 0, NA))
```

© 2018 Kennedy RE et al. *JAMA Network Open*.

```
idx <- !is.na(temp$group)
temp <- temp[idx,]

idx <- order(temp$UID, temp$month)
temp <- temp[idx,]

idx <- !is.na(temp$ADAS)
temp <- temp[idx,]

sample.mixed <- temp
results <- NULL
for (i in unique(sample.mixed$STUDY)) {
  study.name <- i
  writeLines("Study Name")
  print(study.name)
  idx <- sample.mixed$STUDY==study.name
  mixed.data <- sample.mixed[idx,]

  mixed.data$month <- as.numeric(mixed.data$month)
  mixed.data$group <- as.factor(mixed.data$group)
  mixed.data$month <- mixed.data$month / 12
  idx <- order(mixed.data$UID, mixed.data$month)
  mixed.data <- mixed.data[idx,]
  mixed.full <- try(lmer(ADAS ~ AGE + EDUCATION + group + month + group:month
+ (month|UID), REML=FALSE, data=mixed.data))
  mixed.reduced <- try(lmer(ADAS ~ AGE + EDUCATION + group + month + (month|
UID), REML=FALSE, data=mixed.data))

  if ((class(mixed.full) == "try-error") | (class(mixed.reduced) == "try-error")) {
    result <- NA
  } else {
    mixed.p.val <- anova(mixed.full, mixed.reduced)$"Pr(>Chisq)"[2]
    mixed.int <- fixef(mixed.full)["group1"]
    mixed.slope <- fixef(mixed.full)["group1:month"]

    writeLines("Full Mixed Effects Model")
    print(summary(mixed.full))
    writeLines("P Value for Full vs. Reduced Model")
    print(mixed.p.val)
    result <- summary(mixed.full)$coefficients["group1:month",1:2]
    sample.times <- c(0, 6, 12, 18, 24) /12
    med.values <- fixef(mixed.full)["(Intercept)"] + fixef(mixed.full)["group1"] + sample.t
imes * fixef(mixed.full)["month"] + sample.times * fixef(mixed.full)["group1:month"]
    nomed.values <- fixef(mixed.full)["(Intercept)"] + sample.times * fixef(mixed.full)["m
onth"]
```

© 2018 Kennedy RE et al. *JAMA Network Open*.

```
    plot(sample.times, nomed.values, main=study.name, xlab="Time in Years", ylab="Fitted ADAS-cog score", col="black", lty=1, type="l", xaxt="n", ylim=c(0,70))
    lines(sample.times, med.values, col="black", lty=2, type="l", xaxt="n")
    axis(1, at = sample.times)
  }
  results <- rbind(results, result)
}
rownames(results) <- unique(sample.mixed$STUDY)
N <- table(all.ad$STUDY, all.ad$group4)
idx <- N[,1] != 0
N <- N[idx,]
results <- as.data.frame(results)
results$medN <- N[,"Memantine"] + N[,"Both"]
results$nomedN <- N[,"Neither"] + N[,"ChEI"]
results$author <- study.authors[rownames(results)]
results$year <- study.pubyears[rownames(results)]

idx <- order(results$year, results$author)
results <- results[idx,]
meta <- rma(yi=results[,"Estimate"], sei=results[,"Std. Error"], mods=memantine.pct[rownames(results)])
print(meta)
results$severity <- "AD"
all.results <- results
rate.ad <- meta
#pdf("AAICRateForestMemantineModerator080818.pdf", width=11, height=8.5)
forest(meta, slab=paste(rownames(results), " (", results$author, ", ", results$year, ")", sep=""), ilab=cbind(results$medN, results$nomedN), ilab.xpos = c(-40, -25), digits=c(3,0), cex=1.0, xlab="Difference in ADAS-cog Slopes", cex.lab=1, at=c(-15, -10,-5, 0, 5, 10, 15), xlim=c(-70, 40), mlab="RE Model for AD") #, main="Comparison of Memantine + Both vs. ChEIs + Neither")
op <- par(cex = 1.0, font = 2)
text(-70, 7.5, "Abbreviation, Authors, Year", pos = 4, cex=1.0)
text(40.1, 7.5, "Slope Difference [95% CI]", pos = 2, cex=1.0)
text(c(-40, -25), 7.5, c("Memantine", "No Memantine"))
text(-33, 8, "Number of Subjects")
par(op, xpd=TRUE)
text(-21, -0.3, "Faster\ndecline\nwith ChEIs\n/neither")
text(22, -0.3, "Faster\ndecline\nwith both\n/memantine")
#dev.off()
render_markdown()
sessionInfo()
```

© 2018 Kennedy RE et al. *JAMA Network Open*.

Computing Environment

```
## R version 3.5.0 (2018-04-23)
## Platform: x86_64-apple-darwin15.6.0 (64-bit)
## Running under: macOS High Sierra 10.13.6
##
## Matrix products: default
## BLAS: /Library/Frameworks/R.framework/Versions/3.5/Resources/lib/libRblas.0.dyli
b
## LAPACK: /Library/Frameworks/R.framework/Versions/3.5/Resources/lib/libRlapack.
dylib
##
## locale:
## [1] en_US.UTF-8/en_US.UTF-8/en_US.UTF-8/C/en_US.UTF-8/en_US.UTF-8
##
## attached base packages:
## [1] parallel  stats     graphics  grDevices utils     datasets  methods
## [8] base
##
## other attached packages:
##  [1] compute.es_0.2-4    calibrate_1.7.2     MASS_7.3-50
##  [4] EMAtools_0.1.3      lme4_1.1-17         metafor_2.0-0
##  [7] Matrix_1.2-14       car_3.0-0           carData_3.0-1
## [10] compareGroups_3.4.0 SNPassoc_1.9-2      mvtnorm_1.0-7
## [13] haplo.stats_1.7.9   xtable_1.8-2        gdata_2.18.0
## [16] Hmisc_4.1-1         ggplot2_2.2.1       Formula_1.2-3
## [19] survival_2.42-3     lattice_0.20-35     bibtex_0.4.2
## [22] knitcitations_1.0.8 knitr_1.20
##
## loaded via a namespace (and not attached):
##   [1] TH.data_1.0-8       minqa_1.2.4         colorspace_1.3-2
##   [4] modeltools_0.2-22   rio_0.5.10          ggridges_0.5.0
##   [7] sjlabelled_1.0.12   rprojroot_1.3-2     snakecase_0.9.1
##  [10] estimability_1.3    htmlTable_1.12      base64enc_0.1-3
##  [13] rstudioapi_0.7      mice_3.0.0          glmmTMB_0.2.2.0
##  [16] MatrixModels_0.4-1  lubridate_1.7.4     coin_1.2-2
##  [19] RefManageR_1.2.0    xml2_1.2.0          codetools_0.2-15
##  [22] splines_3.5.0       sjmisc_2.7.4        bayesplot_1.6.0
##  [25] jsonlite_1.5        nloptr_1.0.4        broom_0.5.0
##  [28] cluster_2.0.7-1     compiler_3.5.0      httr_1.3.1
##  [31] emmeans_1.2.3       sjstats_0.16.0      backports_1.1.2
##  [34] assertthat_0.2.0    lazyeval_0.2.1      acepack_1.4.1
##  [37] htmltools_0.3.6     quantreg_5.36       tools_3.5.0
##  [40] lmerTest_3.0-1      bindrcpp_0.2.2      coda_0.19-1
##  [43] gtable_0.2.0        glue_1.2.0          dplyr_0.7.5
##  [46] Rcpp_0.12.17        cellranger_1.1.0    nlme_3.1-137
##  [49] lmtest_0.9-36       stringr_1.3.1       openxlsx_4.1.0
```

© 2018 Kennedy RE et al. *JAMA Network Open*.

```
## [52] gtools_3.5.0       stringdist_0.9.5.1  polspline_1.1.12
## [55] pan_1.4            zoo_1.8-1          scales_0.5.0
## [58] hms_0.4.2          sandwich_2.4-0     SparseM_1.77
## [61] pwr_1.2-2          TMB_1.7.14         RColorBrewer_1.1-2
## [64] HardyWeinberg_1.5.9 yaml_2.1.19        curl_3.2
## [67] gridExtra_2.3      epitools_0.5-10    rms_5.1-2
## [70] rpart_4.1-13       latticeExtra_0.6-28 stringi_1.2.2
## [73] highr_0.6          checkmate_1.8.5    zip_1.0.0
## [76] truncnorm_1.0-8    rlang_0.2.0        pkgconfig_2.0.1
## [79] Rsolnp_1.16        DataCombine_0.2.21  evaluate_0.10.1
## [82] prediction_0.3.6   purrr_0.2.5        bindr_0.1.1
## [85] htmlwidgets_1.2    tidyselect_0.2.4   plyr_1.8.4
## [88] magrittr_1.5       R6_2.2.2           mitml_0.3-5
## [91] multcomp_1.4-8     pillar_1.2.3       haven_1.1.2
## [94] foreign_0.8-70     abind_1.4-5        nnet_7.3-12
## [97] tibble_1.4.2       modelr_0.1.2       crayon_1.3.4
## [100] jomo_2.6-2        rmarkdown_1.9      grid_3.5.0
## [103] readxl_1.1.0      data.table_1.11.4   forcats_0.3.0
## [106] digest_0.6.15     numDeriv_2016.8-1   tidyr_0.8.1
## [109] stats4_3.5.0      munsell_0.4.3
```

© 2018 Kennedy RE et al. *JAMA Network Open*.
